# 4-Trifluoromethyl-*p*-quinols as dielectrophiles: three-component, double nucleophilic addition/aromatization reactions

**DOI:** 10.1038/srep26957

**Published:** 2016-06-01

**Authors:** Jinhuan Dong, Lou Shi, Ling Pan, Xianxiu Xu, Qun Liu

**Affiliations:** 1 Department of Chemistry and Jilin Province Key Laboratory of Organic Functional Molecular Design & Synthesis, Northeast Normal University, Changchun 130024, China

## Abstract

In recent years, numerous methods have emerged for the synthesis of trifluoromethylated arenes based on the late-stage introduction of a trifluoromethyl group onto an aryl ring. In sharp comparison, the synthesis of trifluoromethylated arenes based on the pre-introduction of a trifluoromethyl group onto an “aromatic to be” carbon has rarely been addressed. It has been found that 4-trifluoromethyl-*p*-quinol silyl ethers, the readily available and relatively stable compounds, can act as dielectrophiles to be applied to multi-component reactions for the synthesis of various trifluoromethylated arenes. Catalyzed by In(OTf)_3_, 4-trifluoromethyl-*p*-quinol silyl ethers react with C-, N-, and S-nucleophiles, respectively, in a regiospecific 1,2-addition manner to generate the corresponding highly reactive electrophilic intermediates. Further reaction of the *in-situ* generated electrophiles with a C-nucleophile followed by spontaneous aromatization enables the construction of functionalized trifluoromethyl arenes. This three-component, double nucleophilic addition/aromatization reaction based on the pre-introduction of a trifluoromethyl group onto an “aromatic to be” carbon provides a divergent strategy for the synthesis of trifluoromethylated arenes under mild reaction conditions in a single operation.

In the last decade, the introduction of fluorine-containing groups^1^,^2^,^3^,^4^,^5^,^6^,^7^,^8^,^9^,^10^,^11^,^12^,^13^,^14^,^15^,^16^,^17^,^18^,^19^,^20^,^21^,^22^,^23^ into organic molecules has become a major research focus. Trifluoromethyl containing motifs in an aromatic system are common pharmacophores (Fig. 1)^1^,^2^,^24^,^25^,^26^,^27^,^28^,^29^,^30^ and there is a great current interest in the discovery of trifluoromethylation methods upon electrophilic and radical trifluoromethylations of arenes and heteroarenes as a consequence of advances in catalysis^3^,^4^,^5^,^6^,^7^,^8^,^9^, and new trifluoromethylating reagents and methods^6^,^7^,^8^,^9^,^10^,^11^,^12^,^13^. Such growth based on the late-stage introduction of a trifluoromethyl group onto a aryl ring is in stark contrast to the synthetic applications of trifluoromethyltrimethylsilane (TMSCF_3_, also known as Ruppert−Prakash reagent)^14^,^15^, as notably less toxic, relatively cheaper, and widely accepted nucleophilc trifluoromethylating reagent, in the synthesis of trifluoromethylated arenes based on the pre-introduction of a trifluoromethyl group onto a “aromatic to be” substrate^1^,^2^,^3^,^4^,^5^,^6^,^7^,^8^,^9^,^10^,^11^,^12^,^13^,^14^,^15^,^16^,^17^,^18^,^19^.

Due to the high electronegativity of fluorine, the nucleophilic CF_3_ species are considered as hard nucleophiles, which usually undergo 1,2-addition reactions with α,β-unsaturated carbonyl compounds^16^,^17^,^18^,^19^,^20^,^31^,^32^, including divinyl ketones^20^ and *p*-quinones^31^,^32^,^33^. In 1989, Stahly and Bell described the monotrifluoromethylation of *p*-quinones with Et_3_SiCF_3_ and the further transformation to otherwise hardly accessible (trifluoromethyl)phenols by treatment of the adducts, 4-(trifluoromethyl)-*p*-quinol silyl ethers, (or the corresponding alcohols, 4-(trifluoromethyl)-*p*-quinols) by dissolving metal reduction (Fig. 2a)^31^. Stahly’s method provides the first example for the synthesis of trifluoromethylated arenes based on nucleophilic trifluoromethylation of non-aromatic precursors. Although Stahly’s method is limited to a few of simple trifluoromethylated alkyl phenols (3 examples) and uses two equivalents of zinc as the reductant, this method opened a route for the synthesis of trifluoromethylated arenes from simple precursors via the bond formation between the CF_3_ group and the “aromatic to be” carbon^11^,^12^,^31^,^32^,^33^,^34^,^35^.

In our recent research on the synthesis of trifluoromethylated arenes using the readily available 4-(trifluoromethyl)-*p*-quinones as non-aromatic precursors, a new reaction, the 1,3-carbothiolation/aromatization of 4-(trifluoromethyl)-*p*-quinols, has been developed^32^. This reaction enables two different nucleophiles, a thiol and a carbonucleophile generated *in-situ* from ketene dithioacetals^36^,^37^, to be introduced on “aromatic to be” carbons^31^,^32^,^33^,^34^,^35^ in the ***ortho*** and ***para*** positions of the CF_3_ group of 4-(trifluoromethyl)-*p*-quinols via a novel *meta*-double functionalization fashion (Fig. 2b)^32^. Encouraged by the advantage of the 1,3-carbothiolation/aromatization reaction, such as readily available substrates^31^,^32^,^33^,^38^,^39^, operational simplicity, and double functionalization on the “aromatic to be” carbons in a single operation^32^,^33^,^34^,^35^, we pursued the three-component, double nucleophilic addition/aromatization reaction of 4-(trifluoromethyl)-*p*-quinol silyl ethers as dielectrophiles with two nucleophiles, named **Nu1** and **Nu2** (Fig. 2c). In the double nucleophilic addition/aromatization reactions, **Nu1** can be a S-, N-, or C-nucleophile that attacks, in a regiospecific 1,2-addition manner, at the carbonyl carbon of a 4-(trifluoromethyl)-*p*-quinol silyl ether to form a highly reactive electrophilic intermediate as the crucial step. As a result, the subsequent nucleophilic addition of **Nu2** to the *in-situ* generated electrophilic intermediate followed by spontaneous aromatization can lead to a functionalized trifluoromethyl arene. Herein we present these three-component, double nucleophilic addition/aromatization reactions using 4-(trifluoromethyl)-*p*-quinol silyl ethers as the versatile dielectrophilic “aromatic to be” precursors. These approaches allow a variety of functional groups, including an alkylthio, an amino, an aryl group or various carbonyl methyl groups to be introduced onto the “aromatic ring” in a single operation under mild reaction conditions (Fig. 2c).

## Results and Discussion

### Three-component, double nucleophilic 1,3-carbothiolation/aromatization reactions using active methylenes as C-nucleophiles

4-(Trifluoromethyl)-*p*-quinol silyl ethers **1** can be prepared in high yields with the readily available *p*-quinones^38^,^39^ as electrophiles and TMSCF_3_ as the nucleophile^16^,^17^,^18^,^19^,^31^,^32^. In the present research, the three-component reactions of 4-(trifluoromethyl)-*p*-quinol silyl ether **1a** as double electrophile^32^, 1-dodecanethiol as S-nucleophile (**Nu1**)^25^,^26^,^27^,^40^ and acetone **2a** as C-nucleophile (**Nu2**) were first examined (Fig. 3). As a result, the desired product, a trifluoromethyl arene **3aa**, was obtained in moderate yield (Fig. 3, entry 1) under identical reaction conditions as the previous work, catalyzed by indium(III) trifluoromethanesulfonate (In(OTf)_3_) in the solvent, 1,2-dichloroethane (DCE) in the presence of trimethylsilyl chloride (TMSCl) as additive at 70 °C^32^. Under similar reaction conditions as above but in the absence of TMSCl, **3aa** was obtained in lower yield along with arylsulfide **10a** as the minor product formed through 1,3-dithiolation/aromatization (Fig. 3, entry 2), showing the beneficial effect of TMSCl on the formation of **3aa**.

Whereas, under identical conditions as in Fig. 3, entry 1 but at room temperature, **3aa** was produced in high yield (Fig. 3, entry 3). Similar result was obtained by using trimethylsilyl trifluoromethanesulfonate (TMSOTf) as the additive (Fig. 3, entry 5). Lowing the loading of In(OTf)_3_ (Fig. 3, entry 6) or the reaction temperature (Fig. 3, entry 4), resulted in the decrease of the yields of **3aa**. Among the solvents tested, DCE gave the highest yield of **3aa** (Fig. 3, entry 3) in comparison with dichloromethane (DCM, Fig. 3, entry 7), acetonitrile (Fig. 3, entry 8) or THF (Fig. 3, entry 9). With the optimal conditions (Fig. 3, entry 3) in hand, the scope of the three-component, double nucleophilic 1,3-carbothiolation/aromatization reaction of 4-(trifluoromethyl)-*p*-quinol silyl ether **1a** with 1-dodecanethiol as S-nucleophile (**Nu1**) and active methylenes **2** as C-nucleophiles (**Nu2**) were next examined and the results are summarized in Fig. 4. As shown in Fig. 4, various acyclic aliphatic ketones **2a**–**g** can be applied as the C-nucleophiles to give the desired functionalized trifluoromethyl arenes (Fig. 4, entries 1–7), despite the yield of **3ae** was low due to the steric hindrance of 3,3-dimethylbutan-2-one **2e** as the C-nucleophile (Fig. 4, entry 5). In comparison, the less hindered 3-methylbutan-2-one **2d** can react smoothly to enable the formation of **3ad** in high yield (Fig. 4, entry 4) and has an excellent regioselectivity with preferred C−C bond formation at the more substituted carbon of aliphatic ketones **2** (see Fig. 4, entries 2, 4, 6 and 7) via a double nucleophilic 1,3-carbothiolation/aromatization sequence.

**Figure 1:**
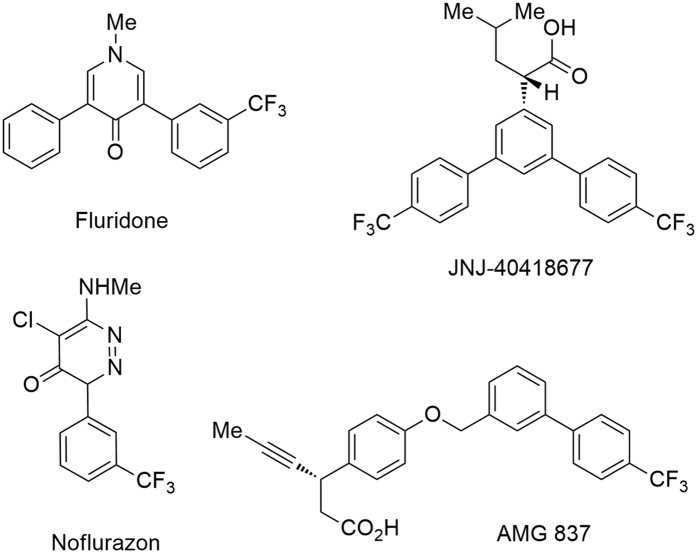
Examples of bioactive trifluoromethylated aromatic compounds.

In the cases of cyclic aliphatic ketones **2h**–**l** as the C-nucleophiles, the desired product **3al**, was obtained in high yield by using cycloheptanone **2l** as the **Nu2** component in the presence of 50 mol% of In(OTf)_3_ (Fig. 4, entry 12). Whereas, the corresponding **3ah**–**3aj** and **3ak**/**3ak′** were produced in low to moderate yields under identical conditions (Fig. 4, entries 8–11) because cyclohexanone is structurally more rigid than either cycloheptanone and acyclic aliphatic ketones, which makes cyclohexanone less reactive towards the C−C bond formation^41^,^42^,^43^. The three-component reaction mentioned above provides a convenient access to α-aryl ketones^41^,^42^,^44^,^45^,^46^,^47^,^48^,^49^ having a trifluoromethyl group on the aryl ring (Fig. 1)^24^,^27^ in a single operation^50^,^51^,^52^,^53^,^54^,^55^. Various methyl aryl ketones including acetophenone **2m**, methyl aryl ketones bearing either electron-donating (**2n** and **2o**) and electron-withdrawing groups (**2p** and **2q**), 1-(thiophen-2-yl)ethanone **2r**, and 2-chloro-1-phenylethanone **2s** were proven the suitable C-nucleophiles for the three-component reaction to deliver the desired products **2m**–**2s** in good to high yields in most cases (Fig. 4, entries 13–19). In addition, trifluoromethylated 2-aryl-1-phenylpropan-1-ones **3at**–**3av** (Fig. 4, entries 20–22) were prepared in good yields by using propiophenone **2t** and propiophenones **2u** and **2v** possessing either an electron-rich (**2u**) and an electron-poor aryl group (**2v**) as the C-nucleophiles, respectively. Furthermore, the corresponding formal α-arylation products **3aw**–**3aa2** of 1-arylpropan-2-ones (**2w** and **2x** as C-nucleophiles) and a variety of β-dicarbonyl compounds (acetoacetone **2y**, ethyl acetoacetate **2z**, ethyl 3-oxopentanoate **2a1**, and 3-methyl acetoacetone **2a2** as C-nucleophiles) were obtained in moderate to excellent yields, respectively (Fig. 4, entries 23–28).

The above three-component reactions (Fig. 4) showed the generality of the active methylene components as the C-nucleophiles (**Nu2**) for their reactions with **1a** as the 1,3-dielectrophile and 1-dodecanethiol as the S-nucleophile (**Nu1**). It was proved that phenylmethanethiol is also an efficient S-nucleophile for the above reaction (Fig. 4, entries 29 and 30). As an extension of the 4-(trifluoromethyl)-*p*-quinol silyl ether components **1**, the desired trifluoromethylated arene products, such as trifluoromethylated naphthalene **3ba** and **3bc**, trifluoromethylated 2-aryl-pentan-3-one **3cc** and **3dc** bearing 3-^*t*^Bu and 3-methyl group respectively on the benzene ring were prepared in good to high yields under similar reaction conditions using 4-(trifluoromethyl)-*p*-quinol silyl ethers **1b, 1c** and **1d** as the 1,3-dielectrophiles, respectively (Fig. 4, entries 31–34). In addition, pentafluoroethylated 2-aryl-pentan-3-one **3ec** was also prepared in high yield from the reaction of 4-(pentafluoroethyl)-*p*-quinol silyl ether **1e** as the 1,3-dielectrophile with 1-dodecanethiol and pentan-3-one **2c** (Fig. 4, entry 35).

### Three-component, double nucleophilic carbothiolation/aromatization reactions using electron-rich arenes as C-nucleophiles

The regioselective double nucleophilic 1,3-addition/aromatization reaction mentioned above provides an easy access to a broad range of α-(*ortho*-trifluoromethyl/pentafluoroethyl-aryl) carbonyl compounds **3** using various active methylene compounds as C-nucleophiles (Fig. 4). Fortunately, when the double nucleophilic addition/aromatization reaction was performed using electron-rich aromatic compounds **4** as the C-nucleophiles (π-nucleophiles), trifluoromethylated biaryls^28^,^29^ were obtained under similar reaction conditions for the synthesis of **3**, whereas at elevated temperatures (Fig. 5). Although numerous trifluoromethylated aromatic compounds have been prepared^1^,^2^,^3^,^4^,^5^,^6^,^7^,^8^,^9^,^10^,^11^,^12^,^13^,^24^,^25^,^26^,^28^,^29^,^30^, few of them are trifluoromethylated biaryls (Fig. 1)^3^,^11^,^12^,^28^,^29^,^30^,^56^,^57^,^58^,^59^,^60^,^61^, which were usually synthesized, for example, by cross-coupling of the corresponding biarylhalides^3^,^11^,^12^,^56^ or biaryl boronic acids^57^ with related trifluoromethylated species, Suzuki–Miyaura coupling of trifluoromethylphenylboronic acid with aryl bromides^58^, and direct arylation of trifluoromethyl benzene with aryl bromides to give a mixture of *para*- and *meta*-products^59^,^60^, respectively.

**Figure 3:**
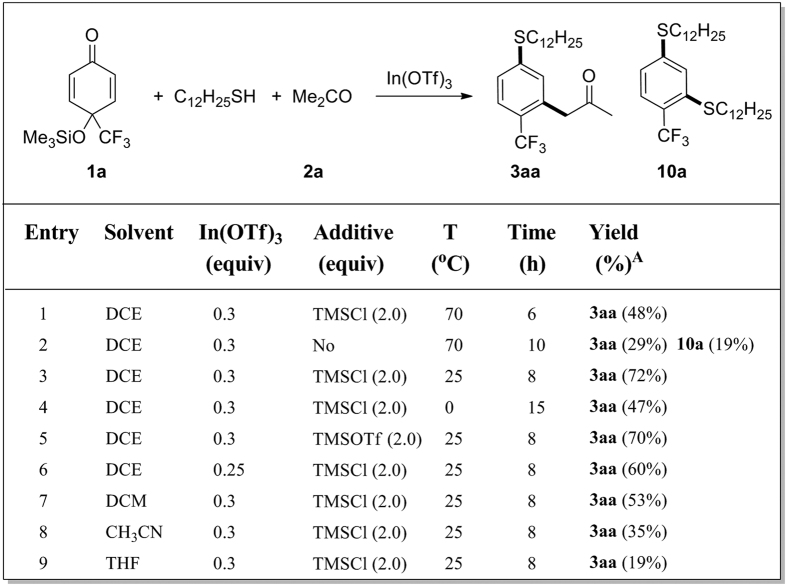
Optimization of reaction conditions. Reaction conditions: 4-(trifluoromethyl)-*p*-quinol silyl ether **1a** (0.6 mmol), 1-dodecanethiol (0.5 mmol), acetone **2a** (1.5 mmol). (A) Isolated yields.

It was found that, under the optimal conditions (Fig. 3, entry 3) but at 60 °C, a mixture of trifluoromethylated biaryls **5aa** and **5aa′** was produced in excellent overall yields by the three-component reaction of **1a**, 1-dodecanethiol as the S-nucleophile and 1,3,5-trimethoxybenzene **4a** as the C-nucleophile via double nucleophilic additions at the 1,3- and 1,2-positions of **1a**, respectively (Fig. 5, entry 1). Similar results were obtained by using phenylmethanethiol as the S-nucleophile (Fig. 5, entry 2)^61^. Under identical conditions as above, the desired trifluoromethylated biaryl compounds **5ba**/**5ba′**, **5ab**/**5ab′**–**5ae**/**5ae′** and **5bf**/**5bf′** were also prepared in moderate to high yields (Fig. 5, entries 3–8). The structure of **5ad**/**5ad′** was confirmed by Nuclear Overhauser Enhancement Spectroscopy (for details, please see the supplementary information). In comparison, trifluoromethylated biaryls **5ag** was produced in moderate yield by using mesitylene (20 equiv) **4g** as the C-nucleophile, (Fig. 5, entry 9). In this case, no the corresponding regioisomer **5ag′**, could be observed. The above results (Fig. 5) showed that the readily available 4-(trifluoromethyl)-*p*-quinol silyl ethers **1** can also act as the “aromatic to be” precursors of trifluoromethylated biaryl compounds.

### Pseudo three-component, double nucleophilic addition/aromatization reactions using electron-rich arenes as C-nucleophiles

In the case of using 1,3-dimethoxybenzene **4i** as the C-nucleophile and performing the reaction of **1a** with **4i** (6 equiv) at 80 °C for 5 h in the absence of a thiol, the double nucleophilic addition/aromatization products, *m*-terphenyl compound, **6** and **6′** were obtained in good overall yield as a 1:1 mixture (Fig. 6)^62^. This pseudo-three component reaction provides an efficient route to trifluoromethylated *m*-terphenyl and *o*-terphenyl compounds, respectively (Fig. 1).

### Three-component, double nucleophilic 1,3-carboamination/aromatization reactions

**Figure 4:**
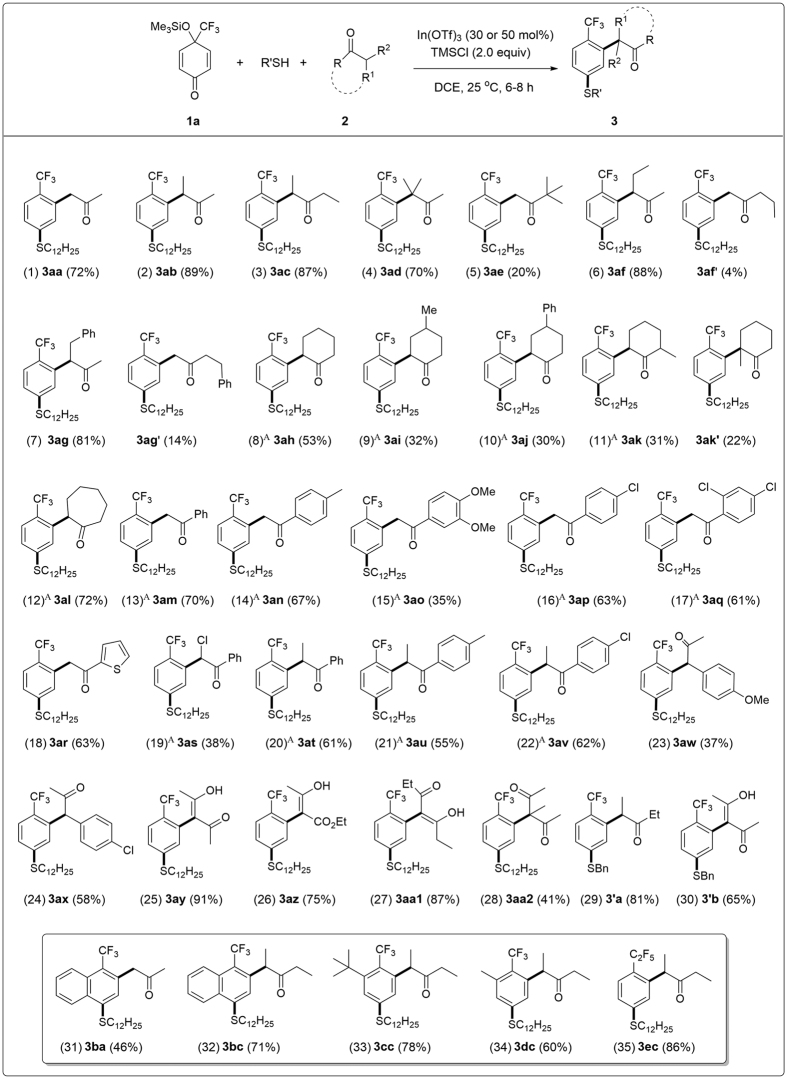
The scope of active methylenes as C-nucleophiles. Reaction conditions: 4-(trifluoromethyl)-*p*-quinol silyl ether **1a** (0.6 mmol), RSH (0.5 mmol), **3** (1.5 mmol), In(OTf)_3_ (0.15 mmol), TMSCl (1.0 mmol), DCE (3 mL), 25 °C, 6–8 h. (A) 0.25 mmol of In(OTf)_3_ was used.

**Figure 5:**
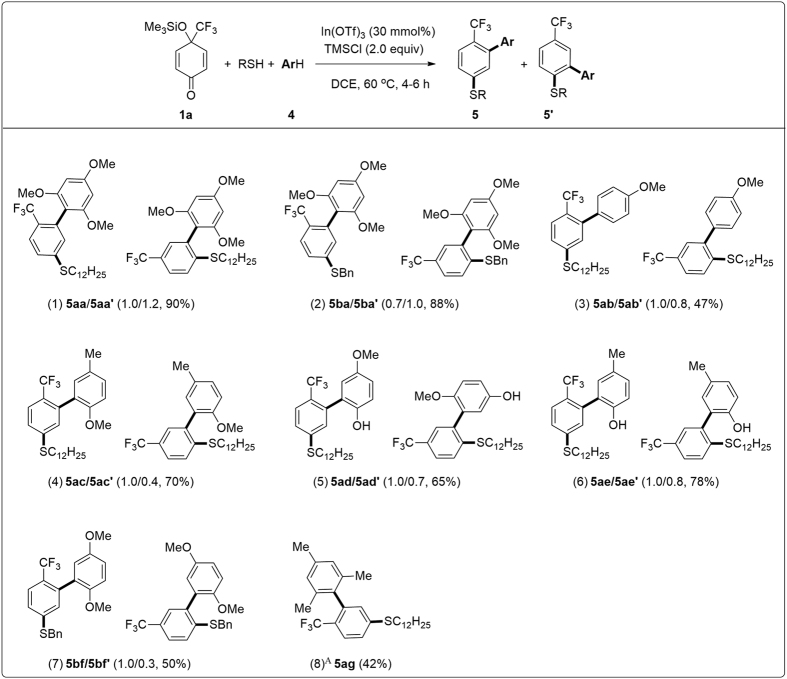
Synthesis of trifluoromethylated biaryl compounds. Reaction conditions: 4-(trifluoromethyl)-*p*-quinol silyl ether **1a** (0.6 mmol), RSH (0.5 mmol), **4** (1.5 mmol), In(OTf)_3_ (0.15 mmol), TMSCl (1.0 mmol), DCE (3 mL), 60 °C, 4–6 h. (A) 20 equiv of mesitylene **4h** was used.

**Figure 6:**
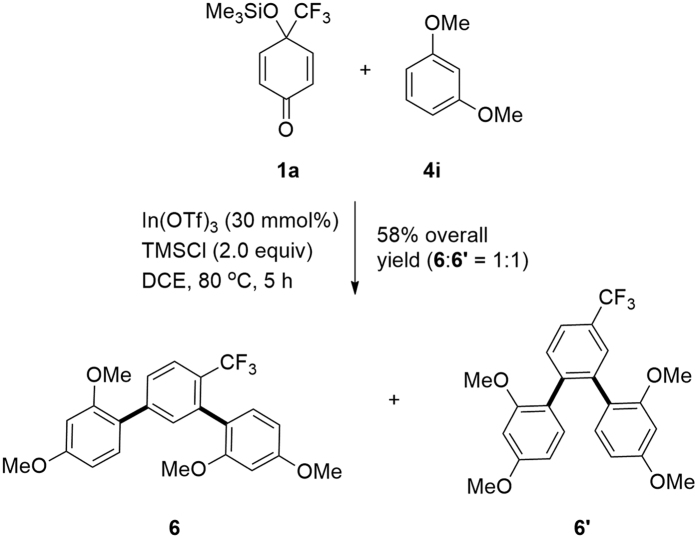
Synthesis of trifluoromethylated terphenyls.

**Figure 7:**
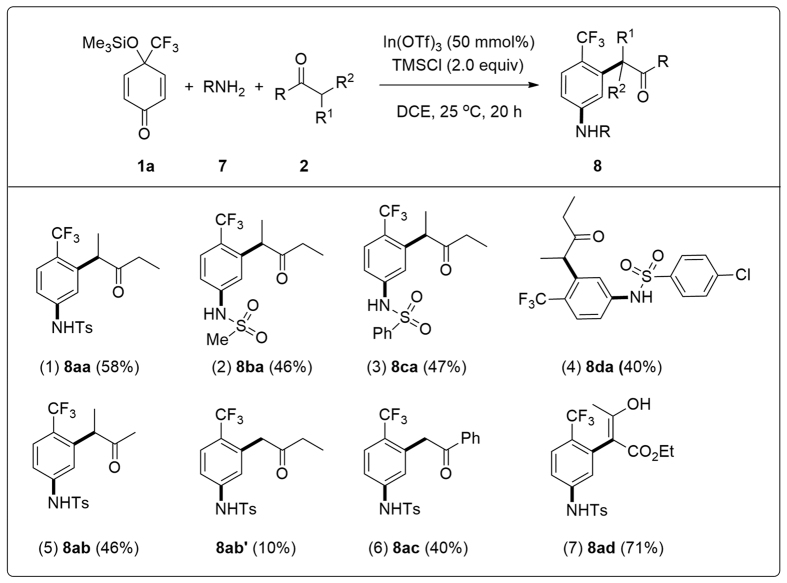
Synthesis of 3-carbonyl methyl-4-(trifluoromethyl)phenyl)benzene sulfonamides.

Promoted by the successful synthesis of functionalized trifluoromethyl arenes **3** (Fig. 4), trifluoromethylated biaryl compounds **4** (Fig. 5), and trifluoromethylated terphenyls **6** (Fig. 6), the three-component reaction using an amine component **7** as the N-nucleophiles (**Nu1**) was examined. Optimization of the reaction conditions for the model reaction of **1a**, 4-methylbenzenesulfonamide **7a** (TsNH_2_), and pentan-3-one **2c** led to the formation of the desired product, benzenesulfonamide **8aa**, in good yield (Fig. 7, entry 1), while **8aa** was obtained in 28% isolated yield without the addition of TMSCl (2.0 equiv) as the additive. In comparison, trifluoromethylated sulfonamides **8ba**–**8da** were obtained in relatively lower yields by using methanesulfonamide **7b**, benzenesulfonamide **7c**, and 4-chlorobenzenesulfonamide **7d** as the N-nucleophiles, respectively (Fig. 7, entries 2–4). Furthermore, the desired trifluoromethylated sulfonamides **8ab**/**8ab′**, **8ac**, and **8ad** were prepared in moderate to high yields (Fig. 7, entries 5–7).

### Reaction mechanism

**Figure 8:**
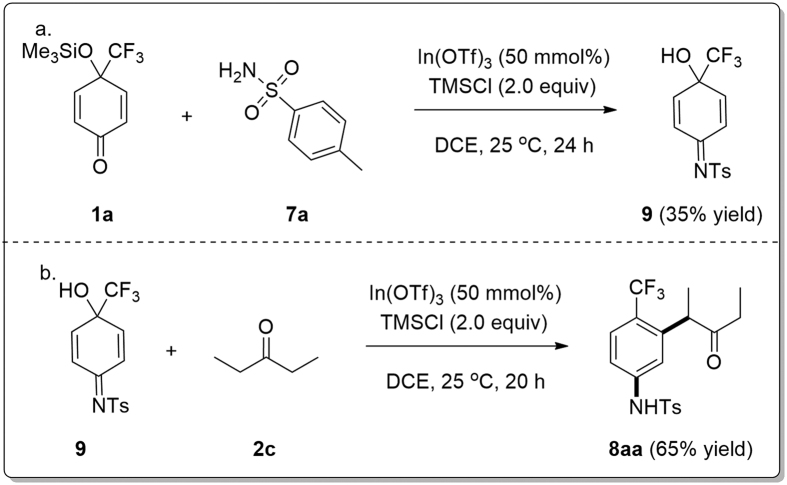
Mechanism studies.

**Figure 9:**
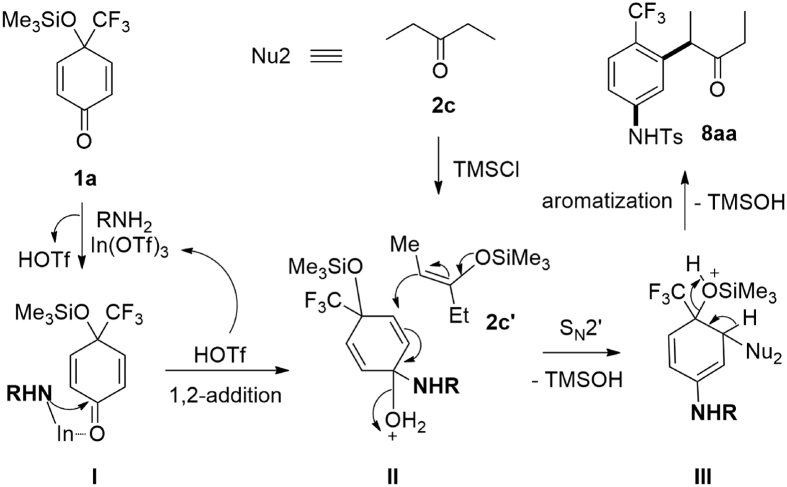
Proposed mechanism for the formation of 8.

To our knowledge, there have been no reports so far of 1,3-carboamination reaction^33^,^63^,^64^,^65^,^66^,^67^. To understand the mechanism for the formation of **8**, the reaction of **1a** with TsNH_2_ **7a** was performed under the identical conditions as used for the synthesis of **8** (Fig. 7) but in the absence of a C-nucleophile. As a result, imine **9** was produced in 35% yield along with 4-(trifluoromethyl)-*p*-quinol in 32% yield (Fig. 8a). Furthermore, it was proven that **8aa** could be formed by the reaction of **9** with pentan-3-one **2c** (Fig. 8b), indicating that imine **9** or the 1,2-adduct of **7** with **1a** (Fig. 9) might be the intermediate for the formation of **8aa** in the three-component, 1,3-carboamination/aromatization reactions (Fig. 7).

Accordingly, a possible mechanism for the formation of **8** was proposed (Fig. 9), with the reaction of **1a** with **7a** (RNH_2_) and **2c** (**Nu2**) as an example), which involves (1) formation of complex **I** from **1a**, In(OTf)_3_ and RNH_2_ along with the release of HOTf^32^; (2) 1,2-addition of RNH at the carbonyl group of **I** in a pseudointramolecular manner to give intermediate **II** along with the regeneration of the catalyst, In(OTf)_3_^32^,^68^; (3) attack of the π-nucleophile **2c′** (generated *in-situ* from ketone **2c** with TMSCl) at C3 of **II** in a S_N_2′manner with the release of TMSOH to afford intermediate **III**^43^, and finally, (4) the release of TMSOH driven by aromatization gives **8** (Fig. 9)^32^,^43^.

**Figure 10:**
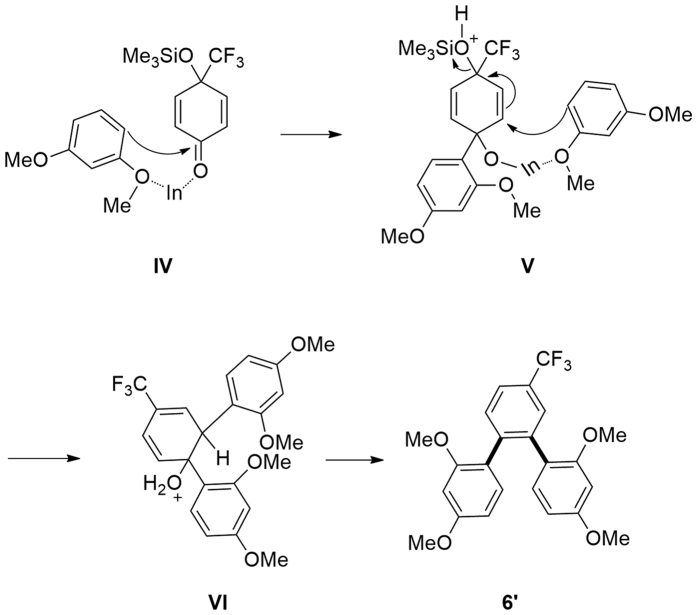
Proposed mechanism for the formation of 6′.

The proposed mechanism (Fig. 9) tells that the regioselective nucleophilic 1,2-addition (**I** → **II**) is the crucial step for the three-component, double nucleophilic addition/aromatization reaction^32^. On the other hand, the addition of TMSCl as an additive is important (Fig. 3) for the activation of ketones through the formation of siloxyalkenes (Figs 4 and 7)^43^,^63^,^64^,^65^,^66^,^67^. Therefore, the formation of trifluoromethylated terphenyls **6** using 1,3-dimethoxybenzene **4i** as **Nu1** should follow a similar mechanism, in which, the 1,2- addition of **4i** at the carbonyl group of complex **IV** (Fig. 10) is to be involved. In this case, complex **IV** should be formed at first and this mechanism can also be used to interpret the formation of *o*-terphenyl product **6′** by the generation of complex **V** (Fig. 10). Furthermore, the formation of trifluoromethylated biaryls **5′** (Fig. 5) via 1,2-carbothiolation/aromatization is easy to understand.

In summary, it has been found that the readily available and relatively stable 4-trifluoromethyl-*p*-quinol silyl ethers are useful dielectrophiles in tandem and/or multi-component reactions. The three-component reactions of 4-trifluoromethyl-*p*-quinol silyl ethers with two nucleophiles provide a convenient access to a wide variety of trifluoromethylated arenes in a single operation under mild reaction conditions. The regioselective nucleophilic 1,2-addition of a nucleophile (**Nu1**) to a 4-trifluoromethyl-*p*-quinol silyl ether enables the formation of a highly reactive electrophilic intermediate, and thus create a useful template for further elaboration to highly functionalized arenes in a concise process. Further works focused on the synthetic applications of these dielectrophiles and analogues are in progress.

## Methods

Detailed experimental procedures, analytical and spectral data for all the new compounds and crystallographic data, see Supplementary Information.

### General procedure for the synthesis of 3,5,6,8 (taking 3aa as an example)

To the solution of 4-(trifluoromethyl)-4-((trimethylsilyl)oxy)cyclohexa-2,5-dienone **1a** (150 mg, 0.60 mmol) and propan-2-one **2a** (111 μL, 1.5 mmol) in DCE (1 mL) was added TMSCl (126 μL, 1 mmol) and In(OTf)_3_ (85 mg, 0.15 mmol). Then, DCE solution (2 mL) of dodecane-1-thiol (120 μL, 0.5 mmol) was added dropwise within 40 min. After the reaction was finished as indicated by TLC (reaction time, 8 h), the resulting mixture was poured into water (20 mL) and extracted with DCM (CH_2_Cl_2_, 20 mL × 3). The combined organic layer was dried over anhydrous Na_2_SO_4_ and concentrated *in vacuo*. The crude product was purified by column chromatography on silica gel (EtOAc/PE = 1: 120) to afford **3aa** (145 mg, 72%) as a white solid (m.p. 57–58 °C).^**1**^**H NMR** (500 MHz, CDCl_3_):δ 0.88 (t, *J* = 7.0 Hz, 3H), 1.26–1.30 (m, 16H), 1.40–1.45 (m, 2H), 1.65–1.71 (m, 2H), 2.19 (s, 3H), 2.95 (t, *J* = 7.5 Hz, 2H), 3.85 (s, 2H), 7.11 (s, 1H), 7.21 (d, *J* = 8.0 Hz, 1H), 7.53 (d, *J* = 8.0 Hz, 1H).^**13**^**C NMR** (125 MHz, CDCl_3_): δ14.1, 22.7, 28.6, 28.8, 29.1, 29.3, 29.4, 29.5 (2), 29.6 (2), 31.9, 32.0, 47.2, 124.9 (CF_3_, q, *J* = 271.4 Hz), 125.1, 125.2 (q, *J* = 30.1 Hz), 126.4 (q, *J* = 5.4 Hz), 130.6, 133.1, 143.2, 204.2.**HRMS** (ESI-TOF) Calcd for C_22_H_34_F_3_OS (M + H)^+^ 403.2277. Found 403.2284.

## Additional Information

**Accession codes**: The X-ray crystallographic data of **5ba′**, **6** and **8aa** have been deposited at the Cambridge Crystallographic Data Centre with CCDC number 1404120–1404122, which can be obtained free of charge via www.ccdc.cam.ac.uk/data_request/cif.

**How to cite this article**: Dong, J. *et al.* 4-Trifluoromethyl-*p*-quinols as dielectrophiles: three-component, double nucleophilic addition/aromatization reactions. *Sci. Rep.* **6**, 26957; doi: 10.1038/srep26957 (2016).

## Supplementary Material

Supplementary Information

## Figures and Tables

**Figure 2:**
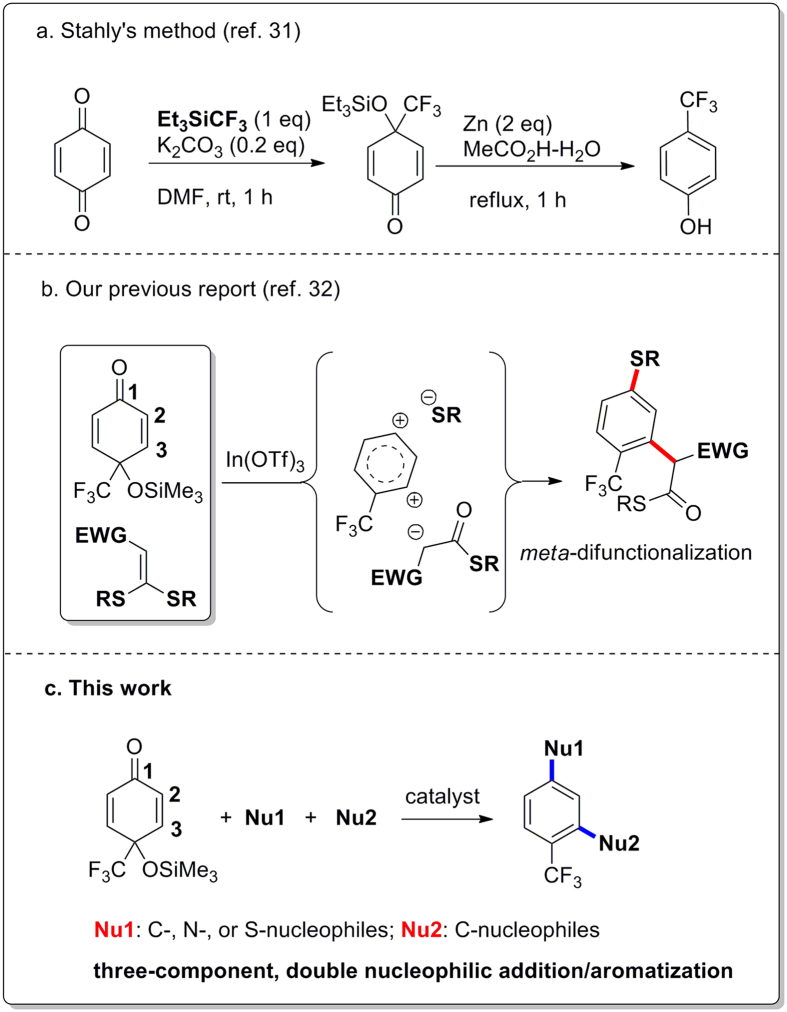
Synthesis of trifluoromethylated arenes based on “aromatic to be” strategy.
