# Supplementary material for: 4-Trifluoromethyl-p-quinols as dielectrophiles: three-component, double nucleophilic addition/aromatization reactions
Source: Sci Rep. 2016 Jun 1;6:26957. doi: 10.1038/srep26957 (PMC4887916; doi:10.1038/srep26957)
Supplement: Supplementary Information [file srep26957-s1.doc]

*Supplementary Information for:*

**4-Triﬂuoromethyl-*p*-quinols as dielectrophiles: three-component, double nucleophilic addition/aromatization reactions**

Jinhuan Dong, Lou Shi, Ling Pan,* Xianxiu Xu and Qun Liu*

Department of Chemistry, Northeast Normal University, Changchun 130024, China

E-mail: [panl948@nenu.edu.cn](mailto:panl948@nenu.edu.cn); [liuqun@nenu.edu.cn](mailto:liuqun@nenu.edu.cn)

**Contents**

Table of Contents -----------------------------------------------------------------------------S1

I. General Information------------------------------------------------------------------------S2

II. Procedures and Analytical Data for Compounds--------------------------------------S2

III. Crystal Data and OPTEP Drawings -------------------------------------------------- S32

IV. Copies of ^1^H NMR, ^13^C NMR and ^19^F NMR Spectra **-**---------------------------- S35

**I. General Information**

All reagents were purchased from commercial sources and used without further purification, unless otherwise indicated. *N*,*N*-Dimethylformamide (DMF) was dried over calcium hydride and distilled before use. *p*-Quinone derivatives were purchased or synthesized according to the literature.[[1]](#footnote-2) All reactions were carried out in the sealed tubes and monitored by TLC, which was performed on precoated aluminum sheets of silica gel 60 (F254). The products were purified by flash column chromatography on silica gel (300−400 mesh). Melting points were uncorrected. NMR spectra were obtained on a Varian Inova 500 spectrometer (500 MHz for ^1^H NMR; 125 MHz for ^13^C NMR; 470 MHz for ^19^F NMR). ^1^H NMR and ^13^C NMR were determined with TMS as the internal standard. ^19^F NMR was determined with C_6_H_5_F as external reference. All chemical shifts are given in ppm. High-resolution mass spectra (HRMS) were obtained using a Bruker microTOF II focus spectrometer (ESI).

**II. Procedures and Analytical Data for Compounds**

**Three-Component, Double Nucleophilic 1,3-Carbothiolation/Aromatization Reactions Using Active Methylenes as C-Nucleophiles**

**3aa**:1-(5-(dodecylthio)-2-(trifluoromethyl)phenyl)propan-2-one

To the solution of 4-(trifluoromethyl)-4-((trimethylsilyl)oxy)cyclohexa-2,5-dienone **1a** (150 mg, 0.60 mmol) and propan-2-one **2a** (111 μL, 1.5 mmol) in DCE (1 mL) was added TMSCl (126 μL, 1 mmol) and In(OTf)_3_ (85 mg, 0.15 mmol). Then, DCE solution (2 mL) of dodecane-1-thiol (120 μL, 0.5 mmol) was added dropwise within 40min. After the reaction was finished as indicated by TLC (reaction time, 8 h), the resulting mixture was poured into water (20 mL) and extracted with DCM (CH_2_Cl_2_, 20 mL×3). The combined organic layer was dried over anhydrous Na_2_SO_4_ and concentrated *in vacuo*. The crude product was purified by column chromatography on silica gel (EtOAc/PE = 1: 120) to afford **3aa** (145 mg, 72%).

White solid, m.p. 57–58 ^o^C. **^1^H NMR** (500 MHz, CDCl_3_): δ 0.88 (t, *J* = 7.0 Hz, 3H), 1.26–1.30 (m, 16H), 1.40–1.45 (m, 2H), 1.65–1.71 (m, 2H), 2.19 (s, 3H), 2.95 (t, *J* = 7.5 Hz, 2H), 3.85 (s, 2H), 7.11 (s, 1H), 7.21 (d, *J* = 8.0 Hz, 1H), 7.53 (d, *J* = 8.0 Hz, 1H). **^13^C NMR** (125 MHz, CDCl_3_): δ 14.1, 22.7, 28.6, 28.8, 29.1, 29.3, 29.4, 29.5 (2), 29.6 (2), 31.9, 32.0, 47.2, 124.9 (CF_3_, q, *J* = 271.4 Hz), 125.1, 125.2 (q, *J* = 30.1 Hz), 126.4 (q, *J* = 5.4 Hz), 130.6, 133.1, 143.2, 204.2. **HRMS** (ESI-TOF) Calcd for C_22_H_34_F_3_OS (M+H) ^+^ 403.2277. Found 403.2284.

**3ab**: 3-(5-(dodecylthio)-2-(trifluoromethyl)phenyl)butan-2-one

Following the procedure for the synthesis of **3aa**, the reaction of 4-(trifluoromethyl)-4-((trimethylsilyl)oxy)cyclohexa-2,5-dienone **1a** (150 mg, 0.60 mmol), dodecane-1-thiol (120 μL, 0.5 mmol) and butan-2-one **2b** (134 μL, 1.5 mmol) gave **3ab** (185 mg, 89%) after purification by column chromatography on silica gel (EtOAc/PE = 1: 120). Reaction time 6 h.

Colorless viscous liquid. **^1^H NMR** (500 MHz, CDCl_3_): δ 0.88 (t, *J* = 7.0 Hz, 3H), 1.26–1.30 (m, 16H), 1.37 (d, *J* = 7.0 Hz, 3H), 1.41–1.44 (m, 2H), 1.63–1.69 (m, 2H), 2.06 (s, 3H), 2.93 (t, *J* = 7.5 Hz, 2H), 4.13 (q, *J* = 7.0 Hz, 1H), 7.05 (s, 1H), 7.19 (d, *J* = 8.0 Hz, 1H), 7.56 (d, *J* = 8.0 Hz, 1H). **^13^C NMR** (125 MHz, CDCl_3_): δ 14.1, 18.0, 22.7, 28.6, 28.8, 29.1, 29.3, 29.4, 29.5, 29.6 (2), 31.8, 31.9, 48.5, 124.6 (CF_3_, q, *J* = 271.6 Hz), 124.8 (q, *J* = 29.9 Hz), 124.9, 126.4, 126.5 (q, *J* = 5.6 Hz), 139.8, 143.9, 207.3. **^19^F NMR** (470 MHz, CDCl_3_)  δ  -60.5. **HRMS** (ESI-TOF) Calcd for C_23_H_36_F_3_OS (M+H) ^+^ 417.2433. Found 417.2475.

**3ac**: 2-(5-(dodecylthio)-2-(trifluoromethyl)phenyl)pentan-3-one

Following the procedure for the synthesis of **3aa**, the reaction of 4-(trifluoromethyl)-4-((trimethylsilyl)oxy)cyclohexa-2,5-dienone **1a** (150 mg, 0.60 mmol), dodecane-1-thiol (120 μL, 0.5 mmol) and pentan-3-one **2c** (157 μL, 1.5 mmol) gave **3ac** (187 mg, 87%) after purification by column chromatography on silica gel (EtOAc/PE = 1: 120). Reaction time 6 h.

Colorless viscous liquid. **^1^H NMR** (500 MHz, CDCl_3_): δ 0.88 (t, *J* = 7.0 Hz, 3H), 0.99 (t, *J* = 7.5 Hz, 3H), 1.26–1.30 (m, 16H), 1.37 (d, *J* = 7.0 Hz, 3H), 1.42–1.45 (m, 2H), 1.63–1.69 (m, 2H), 2.37 (m, 2H), 2.93 (t, *J* = 7.5 Hz, 2H), 4.15 (q, *J* = 7.0 Hz, 1H), 7.09 (s, 1H), 7.18 (d, *J* = 8.5 Hz, 1H), 7.54 (d, *J* = 8.5 Hz, 1H). **^13^C NMR** (125 MHz, CDCl_3_): δ 7.8, 14.0, 18.3, 22.7, 28.6, 28.8, 29.1, 29.3, 29.4, 29.5, 29.6 (2), 31.8, 31.9, 34.6, 47.4, 124.6 (CF_3_, q, *J* = 271.5 Hz), 124.7 (q, *J* = 29.6 Hz), 124.9, 126.4 (q, *J* = 5.6 Hz), 126.5, 140.1, 143.8, 210.0. **HRMS** (ESI-TOF) Calcd for C_24_H_38_F_3_OS (M+H) ^+^ 431.2590. Found 431.2559.

**3ad**: 3-(5-(dodecylthio)-2-(trifluoromethyl)phenyl)-3-methylbutan-2-one

Following the procedure for the synthesis of **3aa**, the reaction of 4-(trifluoromethyl)-4-((trimethylsilyl)oxy)cyclohexa-2,5-dienone **1a** (150 mg, 0.60 mmol), dodecane-1-thiol (120 μL, 0.5 mmol) and 3-methylbutan-2-one **2d** (161 μL, 1.5 mmol) gave **3ad** (150 mg, 70%) after purification by column chromatography on silica gel (EtOAc/PE = 1: 120). Reaction time 6 h.

Colorless viscous liquid. **^1^H NMR** (500 MHz, CDCl_3_): δ 0.88 (t, *J* = 7.0 Hz, 3H), 1.26–1.30 (m, 16H), 1.45–1.48 (m, 2H), 1.54 (s, 6H), 1.70–1.73 (m, 2H), 2.01 (s, 3H), 2.99 (t, *J* = 7.5 Hz, 2H), 7.25 (d, *J* = 8.0 Hz, 1H), 7.43 (s, 1H), 7.59 (d, *J* = 8.0 Hz, 1H). **^13^C NMR** (125 MHz, CDCl_3_): δ 14.0, 22.6, 25.5, 26.8, 28.7, 28.8, 29.1, 29.3, 29.4, 29.5, 29.6 (2), 31.8, 32.0, 52.9, 124.4 (q, *J* = 30.8 Hz), 124.6 (CF_3_, q, *J* = 271.6 Hz), 124.7, 126.5, 129.1 (q, *J* = 5.5 Hz), 142.8, 143.4, 209.6. **^19^F NMR** (470 MHz, CDCl_3_)  δ  -56.7. **HRMS** (ESI-TOF) Calcd for C_24_H_38_F_3_OS (M+H) ^+^ 431.2590. Found 431.2598.

**3ae**: 1-(5-(dodecylthio)-2-(trifluoromethyl)phenyl)-3,3-dimethylbutan-2-one

Following the procedure for the synthesis of **3aa**, the reaction of 4-(trifluoromethyl)-4-((trimethylsilyl)oxy)cyclohexa-2,5-dienone **1a** (150 mg, 0.60 mmol), dodecane-1-thiol (120 μL, 0.5 mmol) and 3,3-dimethylbutan-2-one **2e** (187 μL, 1.5 mmol) gave **3ae** (44 mg, 20%) after purification by column chromatography on silica gel (EtOAc/PE = 1: 150). Reaction time 8 h.

Colorless viscous liquid. **^1^H NMR** (500 MHz, CDCl_3_): δ 0.88 (t, *J* = 7.0 Hz, 3H), 1.24 (s, 9H), 1.26–1.30 (m, 16H), 1.41–1.44 (m, 2H), 1.64–1.70 (m, 2H), 2.94 (t, *J* = 7.5 Hz, 2H), 3.98 (s, 2H), 7.03 (s, 1H), 7.19 (d, *J* = 8.5 Hz, 1H), 7.51 (d, *J* = 8.5 Hz, 1H). **^13^C NMR** (125 MHz, CDCl_3_): δ 14.1, 22.7, 26.7, 28.7, 28.9, 29.1, 29.3, 29.4, 29.5, 29.6 (2), 31.9, 32.3, 40.7, 44.5, 124.7 (CF_3_, q, *J* = 271.4 Hz), 125.0, 125.5 (q, *J* = 30.1 Hz), 126.3 (q, *J* = 5.4 Hz), 131.4, 133.8, 142.6, 211.3. **HRMS** (ESI-TOF) Calcd for C_25_H_40_F_3_OS (M+H) ^+^ 445.2746. Found 445.2751.

**3af**: 3-(5-(dodecylthio)-2-(trifluoromethyl)phenyl)pentan-2-one

**3af’**: 1-(5-(dodecylthio)-2-(trifluoromethyl)phenyl)pentan-2-one

Following the procedure for the synthesis of **3aa**, the reaction of 4-(trifluoromethyl)-4-((trimethylsilyl)oxy)cyclohexa-2,5-dienone **1a** (150 mg, 0.60 mmol), dodecane-1-thiol (120 μL, 0.5 mmol) and pentan-2-one **2f** (159 μL, 1.5 mmol) gave **3af** (189 mg, 88%) after purification by column chromatography on silica gel (EtOAc/PE = 1: 120). Reaction time 6 h.

Colorless viscous liquid. **^1^H NMR** (500 MHz, CDCl_3_): δ 0.84 (t, *J* = 7.5 Hz, 3H), 0.88 (t, *J* = 7.0 Hz, 3H), 1.26–1.30 (m, 16H), 1.41–1.44 (m, 2H), 1.63–1.69 (m, 3H), 2.06–2.10 (m, 4H), 2.93 (t, *J* = 7.5 Hz, 2H), 3.98 (t, *J* = 7.0 Hz, 1H), 7.13 (s, 1H), 7.19 (d, *J* = 8.5 Hz, 1H), 7.56 (t, *J* = 8.5 Hz, 1H). **^13^C NMR** (125 MHz, CDCl_3_): δ 12.0, 14.1, 22.7, 26.3, 28.6, 28.8, 29.1, 29.3, 29.4, 29.5, 29.6 (2), 29.8, 31.8, 31.9, 55.3, 124.8 (CF_3_, q, *J* = 271.6 Hz), 124.9, 125.3 (q, *J* = 29.6 Hz), 126.4 (q, *J* = 5.5 Hz), 126.5, 138.2, 143.7, 207.1. **HRMS** (ESI-TOF) Calcd for C_24_H_38_F_3_OS (M+H) ^+^ 431.2590. Found 431.2567.

**3ag**: 3-(5-(dodecylthio)-2-(trifluoromethyl)phenyl)-4-phenylbutan-2-one

**3ag’**: 1-(5-(dodecylthio)-2-(trifluoromethyl)phenyl)-4-phenylbutan-2-one

Following the procedure for the synthesis of **3aa**, the reaction of 4-(trifluoromethyl)-4-((trimethylsilyl)oxy)cyclohexa-2,5-dienone **1a** (150 mg, 0.60 mmol), dodecane-1-thiol (120 μL, 0.5 mmol) and 4-phenylbutan-2-one **2g** (225 μL, 1.5 mmol) gave **3ag** (199 mg, 81%) and **3ag’** (34 mg, 14%) after purification by column chromatography on silica gel (EtOAc/PE = 1: 120). Reaction time 6 h.

**3ag**, Colorless viscous liquid. **^1^H NMR** (500 MHz, CDCl_3_): δ 0.88 (t, *J* = 7.0 Hz, 3H), 1.26–1.30 (m, 16H), 1.43–1.47 (m, 2H), 1.65–1.71 (m, 2H), 1.96 (s, 3H), 2.81 (dd, *J* = 8.5 Hz, *J* = 4.0 Hz, 1H), 2.95 (t, *J* = 7.5 Hz, 2H), 3.39 (dd, *J* = 4.0 Hz, *J* = 8.5 Hz, 1H), 4.40 (dd, *J* = 8.5 Hz, *J* = 4.0 Hz, 1H), 7.14–7.19 (m, 4H), 7.21–7.24 (m, 2H), 7.28 (s, 1H), 7.54 (d, *J* = 8.5 Hz, 1H). **^13^C NMR** (125 MHz, CDCl_3_): δ 14.1, 22.6, 28.6, 28.8, 29.1, 29.3, 29.4, 29.5, 29.6 (2), 30.2, 31.9, 39.2, 55.7, 124.6 (CF_3_, q, *J* = 271.6 Hz), 124.7 (q, *J* = 29.8 Hz), 125.0, 126.3, 126.5 (q, *J* = 5.5 Hz), 126.6, 128.3, 129.1, 137.6, 139.2, 143.9, 206.1. **HRMS** (ESI-TOF) Calcd for C_29_H_40_F_3_OS (M+H) ^+^ 493.2746. Found 493.2784.

**3ag’,** Colorless viscous liquid. **^1^H NMR** (500 MHz, CDCl_3_): δ 0.88 (t, *J* = 7.0 Hz, 3H), 1.26–1.30 (m, 16H), 1.41–1.44 (m, 2H), 1.65–1.68 (m, 2H), 2.79 (t, *J* = 7.5 Hz, 2H), 2.89–2.94 (m, 4H), 3.81 (s, 2H), 7.05 (s, 1H), 7.16–7.20 (m, 4H), 7.27 (t, *J* = 7.5 Hz, 2H), 7.51 (d, *J* = 7.5 Hz, 1H). **^13^C NMR** (125 MHz, CDCl_3_): δ 14.1, 22.7, 28.6, 28.8, 29.1, 29.3, 29.4, 29.5, 29.6 (2), 31.9, 32.1, 43.8, 46.6, 124.8 (CF_3_, q, *J* = 271.6 Hz), 125.0, 125.1 (q, *J* = 30.1 Hz), 126.1, 126.4 (q, *J* = 5.4 Hz), 128.3, 128.5, 130.7, 132.9, 140.7, 143.1, 205.4. **HRMS** (ESI-TOF) Calcd for C_29_H_39_F_3_NaOS (M+Na) ^+^ 515.2566. Found 515.2566.

**3ah**: 2-(5-(dodecylthio)-2-(trifluoromethyl)phenyl)cyclohexanone

To the solution of 4-(trifluoromethyl)-4-((trimethylsilyl)oxy)cyclohexa-2,5-dienone **1a** (150 mg, 0.60 mmol) and cyclohexanone **2h** (154 μL, 1.5 mmol) in DCE (1 mL) was added TMSCl (126 μL, 1 mmol) and In(OTf)_3_ (141 mg, 0.25 mmol) in the ambient atmosphere. Then, DCE solution (2 mL) of dodecane-1-thiol (120 μL, 0.5 mmol) was added dropwise within 40min. After the reaction was finished as indicated by TLC (reaction time, 8 h), the resulting mixture was poured into water (20 mL) and extracted with DCM (CH_2_Cl_2_, 20 mL×3). The combined organic layer was dried over anhydrous Na_2_SO_4_ and concentrated *in vacuo*. The crude product was purified by column chromatography on silica gel (EtOAc/PE = 1: 150) to afford **3ah** (117 mg, 53%).

Colorless viscous liquid. **^1^H NMR** (500 MHz, CDCl_3_): δ 0.88 (t, *J* = 7.0 Hz, 3H), 1.26–1.30 (m, 16H), 1.40–1.43 (m, 2H), 1.65–1.70 (m, 2H), 1.82–1.87 (m, 2H), 1.95–2.01 (m, 2H), 2.19–2.29 (m, 2H), 2.49–2.56 (m, 2H), 2.94 (t, *J* = 7.5 Hz, 2H), 4.01 (dd, *J* = 13.5 Hz, *J* = 5.0 Hz, 1H), 7.17 (s, 1H), 7.19 (d, *J* = 8.5 Hz, 1H), 7.51 (d, *J* = 8.5 Hz, 1H). **^13^C NMR** (125 MHz, CDCl_3_): δ 14.1, 22.7, 25.7, 27.8, 28.7, 28.8, 29.1, 29.3, 29.4, 29.5, 29.6 (2), 31.9, 32.2, 35.8, 42.3, 53.1, 124.7 (CF_3_, q, *J* = 271.4 Hz), 124.8, 125.1 (q, *J* = 30.1 Hz), 126.0 (q, *J* = 5.6 Hz), 128.9, 137.9, 142.5, 208.4. **^19^F NMR** (470 MHz, CDCl_3_)  δ  -60.6. **HRMS** (ESI-TOF) Calcd for C_25_H_37_F_3_OS (M+H) ^+^ 443.2571. Found 443.2587.

**3ai**: 2-(5-(dodecylthio)-2-(trifluoromethyl)phenyl)-4-methylcyclohexanone

Following the procedure for the synthesis of **3ah**, the reaction of 4-(trifluoromethyl)-4-((trimethylsilyl)oxy)cyclohexa-2,5-dienone **1a** (150 mg, 0.60 mmol), dodecane-1-thiol (120 μL, 0.5 mmol) and 4-methylcyclohexanone **2i** (183 μL, 1.5 mmol) gave **3ai** (73 mg, 32%) after purification by column chromatography on silica gel (EtOAc/PE = 1: 150). Reaction time 8 h.

Colorless viscous liquid. **^1^H NMR** (500 MHz, CDCl_3_): δ 0.88 (t, *J* = 7.0 Hz, 3H), 1.06 (d, *J* = 6.0 Hz, 3H), 1.26–1.30 (m, 16H), 1.42–1.45 (m, 2H), 1.54–1.58 (m, 1H), 1.67–1.73 (m, 3H), 2.12–2.18 (m, 3H), 2.48–2.57 (m, 2H), 2.95 (t, *J* = 7.5 Hz, 2H), 4.08 (dd, *J* = 13.5 Hz, *J* = 5.0 Hz, 1H), 7.16 (s, 1H), 7.19 (d, *J* = 8.0 Hz, 1H), 7.51 (d, *J* = 8.0 Hz, 1H). **^13^C NMR** (125 MHz, CDCl_3_): δ 14.1, 21.1, 22.7, 28.7, 28.9, 29.1, 29.3, 29.4, 29.5, 29.6 (2), 31.9, 32.2, 32.3, 35.7, 41.5, 43.7, 52.1, 124.8 (CF_3_, q, *J* = 271.4 Hz), 124.9, 125.1 (q, *J* = 29.5 Hz), 126.0 (q, *J* = 5.6 Hz), 129.0, 137.8, 142.5, 208.7. **HRMS** (ESI-TOF) Calcd for C_26_H_40_F_3_OS (M+H) ^+^ 457.2746. Found 457.2755.

**3aj**: 2-(5-(dodecylthio)-2-(trifluoromethyl)phenyl)-4-phenylcyclohexanone

Following the procedure for the synthesis of **3ah**, the reaction of 4-(trifluoromethyl)-4-((trimethylsilyl)oxy)cyclohexa-2,5-dienone **1a** (150 mg, 0.60 mmol), dodecane-1-thiol (120 μL, 0.5 mmol) and 4-phenylcyclohexanone **2j** (261 mg, 1.5 mmol) gave **3aj** (77 mg, 30%) after purification by column chromatography on silica gel (EtOAc/PE = 1: 150). Reaction time 8 h.

Yellow viscous liquid. **^1^H NMR** (500 MHz, CDCl_3_): δ 0.88 (t, *J* = 7.0 Hz, 3H), 1.25–1.30 (m, 16H), 1.41–1.44 (m, 2H), 1.66–1.69 (m, 2H), 2.07–2.16 (m, 1H), 2.19–2.27 (m, 1H), 2.34–2.43 (m, 2H), 2.64–2.67 (m, 1H), 2.71–2.77 (m, 1H), 2.95 (t, *J* = 7.0 Hz, 2H), 3.29–3.34 (m, 1H), 4.25 (dd, *J* = 13.0 Hz, *J* = 5.0 Hz, 1H), 7.21 (t, *J* = 7.5 Hz, 2H), 7.24 (d, *J* = 7.5 Hz, 1H), 7.27 (d, *J* = 7.5 Hz, 1H), 7.28 (s, 1H), 7.32 (d, *J* = 7.5 Hz, 1H), 7.33 (t, *J* = 7.5 Hz, 1H), 7.52 (d, *J* = 7.5 Hz, 1H). **^13^C NMR** (125 MHz, CDCl_3_): δ 14.1, 22.6, 28.7, 28.8, 29.1, 29.3, 29.4, 29.5, 29.6 (2), 31.9, 32.3, 34.6, 41.7, 42.7, 43.4, 52.5, 124.8 (CF_3_, q, *J* = 271.4 Hz), 124.9, 125.1 (q, *J* = 29.5 Hz), 126.1 (q, *J* = 5.6 Hz), 126.6, 126.8, 128.6, 129.0, 137.3, 142.7, 143.8, 207.8. **HRMS** (ESI-TOF) Calcd for C_31_H_42_F_3_OS (M+H) ^+^ 519.2903. Found 519.2906.

**3ak**: 2-(5-(dodecylthio)-2-(trifluoromethyl)phenyl)-6-methylcyclohexanone

**3ak’**: 2-(5-(dodecylthio)-2-(trifluoromethyl)phenyl)-2-methylcyclohexanone

Following the procedure for the synthesis of **3ah**, the reaction of 4-(trifluoromethyl)-4-((trimethylsilyl)oxy)cyclohexa-2,5-dienone **1a** (150 mg, 0.60 mmol), dodecane-1-thiol (120 μL, 0.5 mmol) and 2-methylcyclohexanone **2k** (182 μL, 1.5 mmol) gave **3ak** (71 mg, 31%) and **3ak’** (50 mg, 22%) after purification by column chromatography on silica gel (EtOAc/PE = 1: 150). Reaction time 8 h.

**3ak,** Colorless viscous liquid. **^1^H NMR** (500 MHz, CDCl_3_): δ 0.88 (t, *J* = 7.0 Hz, 3H), 1.06 (d, *J* = 6.0 Hz, 3H), 1.26–1.30 (m, 16H), 1.42–1.44 (m, 2H), 1.52–1.55 (m, 1H), 1.66–1.70 (m, 2H), 1.92–1.99 (m, 3H), 2.21–2.26 (m, 2H), 2.59–2.65 (m, 1H), 2.95 (t, *J* = 7.5 Hz, 2H), 4.03 (t, *J* = 7.5 Hz, 1H), 7.19 (d, *J* = 8.0 Hz, 1H), 7.21 (s, 1H), 7.51 (d, *J* = 8.0 Hz, 1H). **^13^C NMR** (125 MHz, CDCl_3_): δ 14.1, 14.7, 22.7, 25.7, 28.7, 28.9, 29.1, 29.3, 29.4, 29.5, 29.6 (2), 31.9, 32.3, 36.4, 37.2, 45.8, 53.0, 124.7 (CF_3_, q, *J* = 271.4 Hz), 124.8, 125.0 (q, *J* = 30.1 Hz), 125.9 (q, *J* = 5.6 Hz), 129.1, 137.8, 142.4, 209.7. **HRMS** (ESI-TOF) Calcd for C_26_H_40_F_3_OS (M+H) ^+^ 457.2746. Found 457.2753.

**3ak’,** Colorless viscous liquid. **^1^H NMR** (500 MHz, CDCl_3_): δ 0.88 (t, *J* = 7.0 Hz, 3H), 1.26–1.30 (m, 16H), 1.41–1.46 (m, 2H), 1.54 (s, 3H), 1.65–1.77 (m, 5H), 1.92–1.99 (m, 2H), 2.43–2.51 (m, 3H), 2.96 (t, *J* = 7.5 Hz, 2H), 7.23 (d, *J* = 8.0 Hz, 1H), 7.39 (s, 1H), 7.59 (d, *J* = 8.0 Hz, 1H). **^13^C NMR** (125 MHz, CDCl_3_): δ 14.1, 21.5, 22.7, 25.9, 26.8, 28.8, 28.9, 29.1, 29.3, 29.4, 29.5, 29.6, 29.6, 31.9, 32.2, 38.8, 41.5, 54.8, 124.5 (q, *J* = 30.1 Hz), 124.6 (CF_3_, q, *J* = 271.4 Hz), 124.7, 126.9, 129.2 (q, *J* = 5.6 Hz), 142.7, 143.0, 211.9. **HRMS** (ESI-TOF) Calcd for C_26_H_40_F_3_OS (M+H) ^+^ 457.2746. Found 457.2759.

**3al**: 2-(5-(dodecylthio)-2-(trifluoromethyl)phenyl)cycloheptanone

Following the procedure for the synthesis of **3ah**, the reaction of 4-(trifluoromethyl)-4-((trimethylsilyl)oxy)cyclohexa-2,5-dienone **1a** (150 mg, 0.60 mmol), dodecane-1-thiol (120 μL, 0.5 mmol) and cycloheptanone **2l** (176 μL, 1.5 mmol) gave **3al** (164 mg, 72%) after purification by column chromatography on silica gel (EtOAc/PE = 1: 150). Reaction time 8 h.

Colorless viscous liquid. **^1^H NMR** (500 MHz, CDCl_3_): δ 0.89 (t, *J* = 7.0 Hz, 3H), 1.26–1.31 (m, 17H), 1.43–1.44 (m, 2H), 1.61–1.64 (m, 1H), 1.66–1.71 (m, 2H), 1.81–1.91 (m, 2H), 1.97–2.04 (m, 4H), 2.51–2.57 (m, 1H), 2.70–2.73 (m, 1H), 2.96 (t, *J* = 7.0 Hz, 2H), 4.33 (d, *J* = 11.0 Hz, 1H), 7.17 (d, *J* = 8.5 Hz, 1H), 7.27 (s, 1H), 7.47 (d, *J* = 8.5 Hz, 1H). **^13^C NMR** (125 MHz, CDCl_3_): δ 14.0, 22.6, 23.3, 28.6, 28.7, 28.8, 29.1, 29.3, 29.4, 29.5, 29.6, 29.9, 31.8, 32.1, 33.4, 43.6, 51.9, 123.9 (q, *J* = 29.1 Hz), 124.3 (CF_3_, q, *J* = 271.4 Hz), 124.5, 125.6 (q, *J* = 5.6 Hz), 128.7, 140.1, 142.6, 211.1. **HRMS** (ESI-TOF) Calcd for C_26_H_40_F_3_OS (M+H) ^+^ 457.2746. Found 457.2753.

**3am**: 2-(5-(dodecylthio)-2-(trifluoromethyl)phenyl)-1-phenylethanone

Following the procedure for the synthesis of **3ah**, the reaction of 4-(trifluoromethyl)-4-((trimethylsilyl)oxy)cyclohexa-2,5-dienone **1a** (150 mg, 0.60 mmol), dodecane-1-thiol (120 μL, 0.5 mmol) and acetophenone **2m** (175 μL, 1.5 mmol) gave **3am** (162 mg, 70%) after purification by column chromatography on silica gel (EtOAc/PE = 1: 150). Reaction time 8 h.

Colorless viscous liquid. **^1^H NMR** (500 MHz, CDCl_3_): δ 0.88 (t, *J* = 7.0 Hz, 3H), 1.26–1.30 (m, 16H), 1.38–1.42 (m, 2H), 1.62–1.67 (m, 2H), 2.93 (t, *J* = 7.5 Hz, 2H), 4.44 (s, 2H), 7.14 (s, 1H), 7.23 (d, *J* = 8.5 Hz, 1H), 7.48 (t, *J* = 7.5 Hz, 2H), 7.56 (d, *J* = 8.5 Hz, 1H), 7.59 (t, *J* = 7.5 Hz, 1H), 8.00 (d, *J* = 7.5 Hz, 2H). **^13^C NMR** (125 MHz, CDCl_3_): δ 14.1, 22.7, 28.6, 28.8, 29.1, 29.3, 29.4, 29.5, 29.6 (2), 31.9, 32.1, 42.3, 124.6 (CF_3_, q, *J* = 272.0 Hz), 125.1, 125.2 (q, *J* = 30.1 Hz), 126.4 (q, *J* = 5.1 Hz), 128.2, 128.7, 130.8, 133.4, 133.5, 136.3, 142.9, 195.9. **HRMS** (ESI-TOF) Calcd for C_27_H_36_F_3_OS (M+H) ^+^ 465.2433. Found 465.2426.

**3an**: 2-(5-(dodecylthio)-2-(trifluoromethyl)phenyl)-1-(p-tolyl)ethanone

Following the procedure for the synthesis of **3ah**, the reaction of 4-(trifluoromethyl)-4-((trimethylsilyl)oxy)cyclohexa-2,5-dienone **1a** (150 mg, 0.60 mmol), dodecane-1-thiol (120 μL, 0.5 mmol) and 1-(p-tolyl)ethanone **2n** (201 μL, 1.5 mmol) gave **3an** (160 mg, 67%) after purification by column chromatography on silica gel (EtOAc/PE = 1: 150). Reaction time 8 h.

Colorless viscous liquid. **^1^H NMR** (500 MHz, CDCl_3_): δ 0.88 (t, *J* = 7.0 Hz, 3H), 1.26–1.30 (m, 16H), 1.39–1.41 (m, 2H), 1.61–1.67 (m, 2H), 2.39 (s, 3H), 2.91 (t, *J* = 7.5 Hz, 2H), 4.40 (s, 2H), 7.13 (s, 1H), 7.21 (d, *J* = 8.5 Hz, 1H), 7.25 (d, *J* = 8.0 Hz, 2H), 7.53 (d, *J* = 8.5 Hz, 1H), 7.89 (d, *J* = 8.0 Hz, 2H). **^13^C NMR** (125 MHz, CDCl_3_): δ 14.1, 21.5, 22.6, 28.6, 28.8, 29.0, 29.3, 29.4, 29.5, 29.6 (2), 31.9, 32.1, 42.2, 124.8 (CF_3_, q, *J* = 272.0 Hz), 125.0, 125.4 (q, *J* = 30.1 Hz), 126.3 (q, *J* = 5.4 Hz), 128.3, 129.3, 130.8, 133.7, 133.9, 142.9, 144.1, 195.5. **^19^F NMR** (470 MHz, CDCl_3_)  δ  -61.5. **HRMS** (ESI-TOF) Calcd for C_28_H_38_F_3_OS (M+H) ^+^ 479.2590. Found 479.2580.

**3ao**: 1-(3,4-dimethoxyphenyl)-2-(5-(dodecylthio)-2-(trifluoromethyl)phenyl)ethanone

Following the procedure for the synthesis of **3ah**, the reaction of 4-(trifluoromethyl)-4-((trimethylsilyl)oxy)cyclohexa-2,5-dienone **1a** (150 mg, 0.60 mmol), dodecane-1-thiol (120 μL, 0.5 mmol) and 1-(3,4-dimethoxyphenyl)ethanone **2o** (270 mg, 1.5 mmol) gave **3ao** (92 mg, 35%) after purification by column chromatography on silica gel (EtOAc/PE = 1: 150). Reaction time 8 h.

White solid, m.p. 80–82 ^o^C. **^1^H NMR** (500 MHz, CDCl_3_): δ 0.88 (t, *J* = 7.0 Hz, 3H), 1.26–1.30 (m, 16H), 1.38–1.42 (m, 2H), 1.64–1.67 (m, 2H), 2.93 (t, *J* = 7.5 Hz, 2H), 3.92 (s, 3H), 3.95 (s, 3H), 4.40 (s, 2H), 6.91 (d, *J* = 8.5 Hz, 1H), 7.15 (s, 1H), 7.22 (d, *J* = 8.0 Hz, 1H), 7.54 (s, 1H), 7.55 (d, *J* = 8.0 Hz, 1H), 7.64 (d, *J* = 8.5 Hz, 1H). **^13^C NMR** (125 MHz, CDCl_3_): δ 14.1, 22.6, 28.6, 28.8, 29.1, 29.3, 29.4, 29.5, 29.6 (2), 31.9, 32.1, 41.8, 55.9, 56.0, 110.0, 110.3, 122.9, 124.9 (CF_3_, q, *J* = 272.0 Hz), 125.1, 125.3 (q, *J* = 30.1 Hz), 126.3 (q, *J* = 5.4 Hz), 129.5, 130.6, 133.8, 142.9, 149.1, 153.5, 194.6. **^19^F NMR** (470 MHz, CDCl_3_)  δ  -61.5. **HRMS** (ESI-TOF) Calcd for C_29_H_40_F_3_O_3_S (M+H) ^+^ 525.2645. Found 525.2681.

**3ap**: 1-(4-chlorophenyl)-2-(5-(dodecylthio)-2-(trifluoromethyl)phenyl)ethanone

Following the procedure for the synthesis of **3ah**, the reaction of 4-(trifluoromethyl)-4-((trimethylsilyl)oxy)cyclohexa-2,5-dienone **1a** (150 mg, 0.60 mmol), dodecane-1-thiol (120 μL, 0.5 mmol) and 1-(4-chlorophenyl)ethanone **2p** (194 μL, 1.5 mmol) gave **3ap** (156 mg, 63%) after purification by column chromatography on silica gel (EtOAc/PE = 1: 150). Reaction time 8 h.

White solid, m.p. 36–38 ^o^C. **^1^H NMR** (500 MHz, CDCl_3_): δ 0.88 (t, *J* = 7.0 Hz, 3H), 1.26–1.30 (m, 16H), 1.39–1.42 (m, 2H), 1.61–1.68 (m, 2H), 2.92 (t, *J* = 7.5 Hz, 2H), 4.39 (s, 2H), 7.13 (s, 1H), 7.23 (d, *J* = 8.0 Hz, 1H), 7.44 (d, *J* = 8.5 Hz, 2H), 7.55 (d, *J* = 8.0 Hz, 1H), 7.93 (d, *J* = 8.5 Hz, 2H). **^13^C NMR** (125 MHz, CDCl_3_): δ 14.1, 22.6, 28.6, 28.8, 29.1, 29.3, 29.4, 29.5, 29.6 (2), 31.8, 32.1, 42.3, 124.8 (CF_3_, q, *J* = 272.0 Hz), 125.2, 125.3 (q, *J* = 28.8 Hz), 126.4 (q, *J* = 5.4 Hz), 129.0, 129.6, 130.7, 133.1, 134.6, 139.8, 143.1, 194.8. **HRMS** (ESI-TOF) Calcd for C_27_H_33_ClF_3_OS (M-H) – 497.1898. Found 497.1862.

**3aq**: 1-(2,4-dichlorophenyl)-2-(5-(dodecylthio)-2-(trifluoromethyl)phenyl)ethanone

Following the procedure for the synthesis of **3ah**, the reaction of 4-(trifluoromethyl)-4-((trimethylsilyl)oxy)cyclohexa-2,5-dienone **1a** (150 mg, 0.60 mmol), dodecane-1-thiol (120 μL, 0.5 mmol) and 1-(2,4-dichlorophenyl)ethanone **2q** (284 mg, 1.5 mmol) gave **3aq** (162 mg, 61%) after purification by column chromatography on silica gel (EtOAc/PE = 1: 150). Reaction time 8 h.

Colorless viscous liquid. **^1^H NMR** (500 MHz, CDCl_3_): δ 0.88 (t, *J* = 7.0 Hz, 3H), 1.26–1.31 (m, 16H), 1.41–1.45 (m, 2H), 1.65–1.71 (m, 2H), 2.96 (t, *J* = 7.5 Hz, 2H), 4.39 (s, 2H), 7.19 (s, 1H), 7.24 (d, *J* = 8.5 Hz, 1H), 7.32 (d, *J* = 7.5 Hz, 1H), 7.46 (d, *J* = 7.5 Hz, 1H), 7.47 (s, 1H), 7.54 (d, *J* = 8.5 Hz, 1H). **^13^C NMR** (125 MHz, CDCl_3_): δ 14.1, 22.7, 28.7, 28.9, 29.1, 29.3, 29.4, 29.5, 29.6 (2), 31.9, 32.2, 46.5, 124.6 (CF_3_, q, *J* = 272.0 Hz), 125.4 (q, *J* = 31.2 Hz), 125.5, 126.5 (q, *J* = 5.4 Hz), 127.5, 130.3, 130.5, 131.1, 132.0, 132.3, 136.9, 137.7, 143.2, 197.5. **HRMS** (ESI-TOF) Calcd for C_27_H_34_Cl_2_F_3_OS (M+H) ^+^ 533.1654. Found 533.1644.

**3ar**: 2-(5-(dodecylthio)-2-(trifluoromethyl)phenyl)-1-(thiophen-2-yl)ethanone

Following the procedure for the synthesis of **3aa**, the reaction of 4-(trifluoromethyl)-4-((trimethylsilyl)oxy)cyclohexa-2,5-dienone **1a** (150 mg, 0.60 mmol), dodecane-1-thiol (120 μL, 0.5 mmol) and 1-(thiophen-2-yl)ethanone **2r** (163 μL, 1.5 mmol) gave **3ar** (148 mg, 63%) after purification by column chromatography on silica gel (EtOAc/PE = 1: 120). Reaction time 6 h.

Colorless viscous liquid. **^1^H NMR** (500 MHz, CDCl_3_): δ 0.88 (t, *J* = 7.0 Hz, 3H), 1.26–1.30 (m, 16H), 1.39–1.42 (m, 2H), 1.63–1.67 (m, 2H), 2.93 (t, *J* = 7.5 Hz, 2H), 4.36 (s, 2H), 7.13 (t, *J* = 4.5 Hz, 1H), 7.20 (s, 1H), 7.21 (d, *J* = 8.0 Hz, 1H), 7.53 (d, *J* = 8.0 Hz, 1H), 7.64 (d, *J* = 4.5 Hz, 1H), 7.76 (d, *J* = 4.5 Hz, 1H). **^13^C NMR** (125 MHz, CDCl_3_): δ 14.1, 22.6, 28.6, 28.8, 29.1, 29.3, 29.4, 29.5, 29.6 (2), 31.9, 32.1, 42.6, 124.8 (CF_3_, q, *J* = 272.0 Hz), 125.1, 125.2 (q, *J* = 30.1 Hz), 126.3 (q, *J* = 5.4 Hz), 128.2, 130.6, 132.3, 132.9, 134.1, 143.1, 143.3, 188.7. **^19^F NMR** (470 MHz, CDCl_3_)  δ  -61.6. **HRMS** (ESI-TOF) Calcd for C_25_H_34_F_3_OS_2_ (M+H) ^+^ 471.1998. Found 471.2004.

**3as**: 2-chloro-2-(5-(dodecylthio)-2-(trifluoromethyl)phenyl)-1-phenylethanone

Following the procedure for the synthesis of **3ah**, the reaction of 4-(trifluoromethyl)-4-((trimethylsilyl)oxy)cyclohexa-2,5-dienone **1a** (150 mg, 0.60 mmol), dodecane-1-thiol (120 μL, 0.5 mmol) and 2-chloro-1-phenylethanone **2s** (231 mg, 1.5 mmol) gave **3as** (95 mg, 38%) after purification by column chromatography on silica gel (EtOAc/PE = 1: 150). Reaction time 8 h.

Colorless viscous liquid. **^1^H NMR** (500 MHz, CDCl_3_): δ 0.88 (t, *J* = 7.0 Hz, 3H), 1.26–1.31 (m, 16H), 1.39–1.44 (m, 2H), 1.63–1.69 (m, 2H), 2.97 (t, *J* = 7.5 Hz, 2H), 6.64 (s, 1H), 7.29 (d, *J* = 8.5 Hz, 1H), 7.49 (t, *J* = 7.5 Hz, 2H), 7.55 (d, *J* = 8.5 Hz, 1H), 7.59–7.62 (m, 2H), 7.99 (d, *J* = 7.5 Hz, 2H). **^13^C NMR** (125 MHz, CDCl_3_): δ 14.1, 22.7, 28.6, 28.8, 29.1, 29.3, 29.4, 29.5, 29.6 (2), 31.9, 32.0, 55.2, 123.4 (CF_3_, q, *J* = 271.6 Hz), 123.8 (CF_3_, q, *J* = 30.1 Hz), 126.2 (q, *J* = 5.6 Hz), 126.8, 128.9, 129.0, 129.1, 133.9, 134.0, 134.1, 144.3, 190.6. **^19^F NMR** (470 MHz, CDCl_3_)  δ -59.9. **HRMS** (ESI-TOF) Calcd for C_27_H_35_ClF_3_OS (M+H) ^+^ 499.2044. Found 499.2052.

**3at**: 2-(5-(dodecylthio)-2-(trifluoromethyl)phenyl)-1-phenylpropan-1-one

Following the procedure for the synthesis of **3ah**, the reaction of 4-(trifluoromethyl)-4-((trimethylsilyl)oxy)cyclohexa-2,5-dienone **1a** (150 mg, 0.60 mmol), dodecane-1-thiol (120 μL, 0.5 mmol) and propiophenone **2t** (201 μL, 1.5 mmol) gave **3at** (145 mg, 61%) after purification by column chromatography on silica gel (EtOAc/PE = 1: 150). Reaction time 8 h.

Colorless viscous liquid. **^1^H NMR** (500 MHz, CDCl_3_): δ 0.88 (t, *J* = 7.0 Hz, 3H), 1.26–1.30 (m, 16H), 1.36–1.38 (m, 2H), 1.54 (d, *J* = 7.0 Hz, 3H), 1.53–1.57 (m, 2H), 2.85 (t, *J* = 7.0 Hz, 2H), 4.98 (q, *J* = 7.0 Hz, 1H), 7.13 (s, 1H), 7.14 (d, *J* = 8.0 Hz, 1H), 7.38 (t, *J* = 7.5 Hz, 2H), 7.48 (t, *J* = 7.5 Hz, 1H), 7.55 (d, *J* = 8.0 Hz, 1H), 7.91 (d, *J* = 7.5 Hz, 2H). **^13^C NMR** (125 MHz, CDCl_3_): δ 14.1, 19.8, 22.7, 28.6, 28.8, 29.1, 29.3, 29.4, 29.5, 29.6 (2), 31.8, 31.9, 43.6, 123.8 (q, *J* = 30.0 Hz), 124.4 (CF_3_, q, *J* = 271.6 Hz), 124.9, 126.8 (q, *J* = 5.3 Hz), 126.9, 128.5, 128.6, 132.9, 136.1, 140.2, 143.7, 199.7. **^19^F NMR** (470 MHz, CDCl_3_)  δ -60.4. **HRMS** (ESI-TOF) Calcd for C_28_H_38_F_3_OS (M+H) ^+^ 479.2590. Found 479.2594.

**3au**: 2-(5-(dodecylthio)-2-(trifluoromethyl)phenyl)-1-(p-tolyl)propan-1-one

Following the procedure for the synthesis of **3ah**, the reaction of 4-(trifluoromethyl)-4-((trimethylsilyl)oxy)cyclohexa-2,5-dienone **1a** (150 mg, 0.60 mmol), dodecane-1-thiol (120 μL, 0.5 mmol) and 1-(p-tolyl)propan-1-one **2u** (223 μL, 1.5 mmol) gave **3au** (135 mg, 55%) after purification by column chromatography on silica gel (EtOAc/PE = 1: 150). Reaction time 8 h.

Colorless viscous liquid. **^1^H NMR** (500 MHz, CDCl_3_): δ 0.88 (t, *J* = 7.0 Hz, 3H), 1.26–1.30 (m, 16H), 1.35–1.38 (m, 2H), 1.52 (d, *J* = 6.5 Hz, 3H), 1.53–1.56 (m, 2H), 2.35 (s, 3H), 2.85 (t, *J* = 7.5 Hz, 2H), 4.96 (q, *J* = 6.5 Hz, 1H), 7.13 (s, 1H), 7.14 (d, *J* = 9.0 Hz, 1H), 7.18 (d, *J* = 8.0 Hz, 2H), 7.54 (d, *J* = 9.0 Hz, 1H), 7.81 (d, *J* = 8.0 Hz, 2H). **^13^C NMR** (125 MHz, CDCl_3_): δ 14.1, 19.8, 21.6, 22.7, 28.6, 28.8, 29.1, 29.3, 29.4, 29.5, 29.6 (2), 31.9, 32.0, 43.5, 123.8 (q, *J* = 29.6 Hz), 124.4 (CF_3_, q, *J* = 271.6 Hz), 124.9, 126.7 (q, *J* = 5.5 Hz), 126.9, 128.8, 129.3, 133.6, 140.5, 143.7, 143.8, 199.4. **^19^F NMR** (470 MHz, CDCl_3_)  δ  -60.4. **HRMS** (ESI-TOF) Calcd for C_29_H_40_F_3_OS (M+H) ^+^ 493.2746. Found 493.2704.

**3av**: 1-(4-chlorophenyl)-2-(5-(dodecylthio)-2-(trifluoromethyl)phenyl)propan-1-one

Following the procedure for the synthesis of **3ah**, the reaction of 4-(trifluoromethyl)-4-((trimethylsilyl)oxy)cyclohexa-2,5-dienone **1a** (150 mg, 0.60 mmol), dodecane-1-thiol (120 μL, 0.5 mmol) and 1-(4-chlorophenyl)propan-1-one **3o** (252 mg, 1.5 mmol) gave **3av** (158 mg, 62%) after purification by column chromatography on silica gel (EtOAc/PE = 1: 150). Reaction time 8 h.

White solid, m.p. 53–54 ^o^C. **^1^H NMR** (500 MHz, CDCl_3_): δ 0.88 (t, *J* = 7.0 Hz, 3H), 1.26–1.30 (m, 16H), 1.35–1.38 (m, 2H), 1.53 (d, *J* = 6.5 Hz, 3H), 1.53–1.57 (m, 2H), 2.85 (t, *J* = 7.0 Hz, 2H), 4.91 (q, *J* = 6.5 Hz, 1H), 7.07 (s, 1H), 7.15 (d, *J* = 8.5 Hz, 1H), 7.35 (d, *J* = 8.5 Hz, 2H), 7.56 (d, *J* = 8.5 Hz, 1H), 7.84 (d, *J* = 8.5 Hz, 2H). **^13^C NMR** (125 MHz, CDCl_3_): δ 14.1, 19.7, 22.7, 28.6, 28.8, 29.1, 29.3, 29.4, 29.5, 29.6 (2), 31.8, 31.9, 43.8, 123.5 (q, *J* = 30.1 Hz), 124.3 (CF_3_, q, *J* = 271.6 Hz), 125.1, 126.8, 126.9 (q, *J* = 5.6 Hz), 128.9, 130.0, 134.4, 139.4, 139.9, 143.9, 198.5. **HRMS** (ESI-TOF) Calcd for C_28_H_37_ClF_3_OS (M+H) ^+^ 513.2200. Found 513.2204.

**3aw**: 1-(5-(dodecylthio)-2-(trifluoromethyl)phenyl)-1-(4-methoxyphenyl)propan-2-one

Following the procedure for the synthesis of **3aa**, the reaction of 4-(trifluoromethyl)-4-((trimethylsilyl)oxy)cyclohexa-2,5-dienone **1a** (150 mg, 0.60 mmol), dodecane-1-thiol (120 μL, 0.5 mmol) and 1-(4-methoxyphenyl)propan-2-one **2w** (231 μL, 1.5 mmol) gave **3aw** (94 mg, 37%) after purification by column chromatography on silica gel (EtOAc/PE = 1: 120). Reaction time 6 h.

Colorless viscous liquid. **^1^H NMR** (500 MHz, CDCl_3_): δ 0.88 (t, *J* = 7.0 Hz, 3H), 1.26–1.30 (m, 16H), 1.34–1.36 (m, 2H), 1.54–1.59 (m, 2H), 2.24 (s, 3H), 2.83 (t, *J* = 7.5 Hz, 2H), 3.79 (s, 3H), 5.53 (s, 1H), 6.89 (d, *J* = 8.5 Hz, 2H), 7.06 (s, 1H), 7.13 (d, *J* = 8.5 Hz, 2H), 7.16 (d, *J* = 8.5 Hz, 1H), 7.52 (d, *J* = 8.5 Hz, 1H). **^13^C NMR** (125 MHz, CDCl_3_): δ 14.1, 22.7, 28.6, 28.8, 29.1, 29.3, 29.4, 29.5, 29.6 (2), 29.8, 31.9, 32.0, 55.2, 58.9, 114.4, 124.4 (CF_3_, q, *J* = 30.0 Hz), 124.5 (CF_3_, q, *J* = 271.6 Hz), 124.9, 126.1 (q, *J* = 5.6 Hz), 128.7, 129.1, 130.2, 137.3, 143.0, 159.0, 205.1. **HRMS** (ESI-TOF) Calcd for C_29_H_40_F_3_O_2_S (M+H) ^+^ 509.2696. Found 509.2692.

**3ax**: 1-(4-chlorophenyl)-1-(5-(dodecylthio)-2-(trifluoromethyl)phenyl)propan-2-one

Following the procedure for the synthesis of **3aa**, the reaction of 4-(trifluoromethyl)-4-((trimethylsilyl)oxy)cyclohexa-2,5-dienone **1a** (150 mg, 0.60 mmol), dodecane-1-thiol (120 μL, 0.5 mmol) and 1-(4-chlorophenyl)propan-2-one **2x** (252 mg, 1.5 mmol) gave **3ax** (148 mg, 58%) after purification by column chromatography on silica gel (EtOAc/PE = 1: 120). Reaction time 6 h.

Colorless viscous liquid. **^1^H NMR** (500 MHz, CDCl_3_): δ 0.88 (t, *J* = 7.0 Hz, 3H), 1.26–1.30 (m, 16H), 1.36–1.38 (m, 2H), 1.56–1.60 (m, 2H), 2.25 (s, 3H), 2.85 (t, *J* = 7.5 Hz, 2H), 5.55 (s, 1H), 7.05 (s, 1H), 7.14 (d, *J* = 8.5 Hz, 2H), 7.19 (d, *J* = 8.5 Hz, 1H), 7.33 (d, *J* = 8.5 Hz, 2H), 7.54 (d, *J* = 8.5 Hz, 1H). **^13^C NMR** (125 MHz, CDCl_3_): δ 14.1, 22.7, 28.7, 28.8, 29.1, 29.3, 29.4, 29.5, 29.6, 29.6, 29.9, 31.9, 58.8, 124.6 (CF_3_, q, *J* = 29.6 Hz), 124.7 (CF_3_, q, *J* = 271.6 Hz), 125.6, 126.4 (q, *J* = 5.5 Hz), 128.7, 129.0, 130.4, 133.7, 135.3, 136.3, 143.5, 204.4. **^19^F NMR** (470 MHz, CDCl_3_)  δ -53.0. **HRMS** (ESI-TOF) Calcd for C_28_H_37_ClF_3_OS (M+H) ^+^ 513.2200. Found 513.2208.

**3ay**: 3-(5-(dodecylthio)-2-(trifluoromethyl)phenyl)-4-hydroxypent-3-en-2-one

Following the procedure for the synthesis of **3aa**, the reaction of 4-(trifluoromethyl)-4-((trimethylsilyl)oxy)cyclohexa-2,5-dienone **1a** (150 mg, 0.60 mmol), dodecane-1-thiol (120 μL, 0.5 mmol) and pentane-2,4-dione **2y** (154 μL, 1.5 mmol) gave **3ay** (202 mg, 91%) after purification by column chromatography on silica gel (EtOAc/PE = 1: 120). Reaction time 6 h.

Colorless viscous liquid. **^1^H NMR** (500 MHz, CDCl_3_): δ 0.88 (t, *J* = 7.0 Hz, 3H), 1.26–1.31 (m, 16H), 1.44–1.47 (m, 2H), 1.68–1.71 (m, 2H), 1.82 (s, 6H), 2.99 (t, *J* = 7.5 Hz, 2H), 7.14 (s, 1H), 7.32 (d, *J* = 8.5 Hz, 1H), 7.63 (d, *J* = 8.5 Hz, 1H), 16.79 (s, 1H). **^13^C NMR** (125 MHz, CDCl_3_): δ 14.1, 22.7, 23.9, 28.6, 28.8, 29.0, 29.1, 29.3, 29.4, 29.5, 29.6 (2), 31.8, 31.9, 111.3, 123.9 (CF_3_, q, *J* = 271.6 Hz), 125.9, 126.5 (CF_3_, q, *J* = 29.6 Hz), 126.9 (q, *J* = 5.3 Hz), 131.1, 135.9, 143.9, 190.6. **^19^F NMR** (470 MHz, CDCl_3_)  δ  -62.7. **HRMS** (ESI-TOF) Calcd for C_24_H_35_F_3_NaO_2_S (M+Na) ^+^ 467.2202. Found 467.2204.

**3az**: ethyl 2-(5-(dodecylthio)-2-(trifluoromethyl)phenyl)-3-hydroxybut-2-enoate

Following the procedure for the synthesis of **3aa**, the reaction of 4-(trifluoromethyl)-4-((trimethylsilyl)oxy)cyclohexa-2,5-dienone **1a** (150 mg, 0.60 mmol), dodecane-1-thiol (120 μL, 0.5 mmol) and ethyl 3-oxobutanoate **2z** (189 μL, 1.5 mmol) gave **3az** (177 mg, 75%) after purification by column chromatography on silica gel (EtOAc/PE = 1: 120). Reaction time 6 h.

Colorless viscous liquid. **^1^H NMR** (500 MHz, CDCl_3_): δ 0.79 (t, *J* = 7.0 Hz, 3H), 1.03 (t, *J* = 7.0 Hz, 3H), 1.17–1.20 (m, 16H), 1.35–1.38 (m, 2H), 1.57–1.63 (m, 2H), 1.64 (s, 3H), 2.88 (t, *J* = 7.5 Hz, 2H), 3.93–3.99 (m, 1H), 4.11–4.17 (m, 1H), 7.00 (s, 1H), 7.19 (d, *J* = 8.0 Hz, 1H), 7.48 (d, *J* = 8.0 Hz, 1H), 12.9 (s, 1H). **^13^C NMR** (125 MHz, CDCl_3_): δ 13.9, 14.0, 19.8, 22.6, 28.6, 28.8, 29.1, 29.3, 29.4, 29.5, 29.6 (2), 31.9, 32.1, 60.7, 100.9, 124.1 (CF_3_, q, *J* = 271.6 Hz), 125.8, 126.4 (q, *J* = 5.2 Hz), 126.7 (CF_3_, q, *J* = 29.8 Hz), 131.4, 134.4, 142.7, 171.8, 173.9. **^19^F NMR** (470 MHz, CDCl_3_)  δ  -62.9. **HRMS** (ESI-TOF) Calcd for C_25_H_38_F_3_O_3_S (M+H) ^+^ 475.2488. Found 475.2489.

**3aa1**: 4-(5-(dodecylthio)-2-(trifluoromethyl)phenyl)-5-hydroxyhept-4-en-3-one

Following the procedure for the synthesis of **3aa**, the reaction of 4-(trifluoromethyl)-4-((trimethylsilyl)oxy)cyclohexa-2,5-dienone **1a** (150 mg, 0.60 mmol), dodecane-1-thiol (120 μL, 0.5 mmol) and heptane-3,5-dione **2a1** (203 μL, 1.5 mmol) gave **3aa1** (205 mg, 87%) after purification by column chromatography on silica gel (EtOAc/PE = 1: 120). Reaction time 6 h.

Colorless viscous liquid. **^1^H NMR** (500 MHz, CDCl_3_): δ 0.88 (t, *J* = 7.0 Hz, 3H), 1.03 (t, *J* = 7.5 Hz, 6H), 1.26–1.31 (m, 16H), 1.43–1.46 (m, 2H), 1.65–1.71 (m, 2H), 1.98–2.07 (m, 4H), 2.97 (t, *J* = 7.5 Hz, 2H), 7.12 (s, 1H), 7.31 (d, *J* = 8.0 Hz, 1H), 7.62 (d, *J* = 8.0 Hz, 1H), 16.79 (s, 1H). **^13^C NMR** (125 MHz, CDCl_3_): δ 8.9, 14.1, 22.7, 28.7, 28.8, 29.1, 29.3, 29.4, 29.5, 29.6 (2), 29.8, 31.9, 32.1, 110.1, 124.0 (CF_3_, q, *J* = 271.6 Hz), 126.1, 126.6 (CF_3_, q, *J* = 29.0 Hz), 126.9 (q, *J* = 5.2 Hz), 131.4, 135.5, 143.7, 193.6. **HRMS** (ESI-TOF) Calcd for C_26_H_40_F_3_O_2_S (M+H) ^+^ 473.2696. Found 473.2608.

**3aa2**: 3-(5-(dodecylthio)-2-(trifluoromethyl)phenyl)-3-methylpentane-2,4-dione

Following the procedure for the synthesis of **3aa**, the reaction of 4-(trifluoromethyl)-4-((trimethylsilyl)oxy)cyclohexa-2,5-dienone **1a** (150 mg, 0.60 mmol), dodecane-1-thiol (120 μL, 0.5 mmol) and 3-methylpentane-2,4-dione **2a2** (173 μL, 1.5 mmol) gave **3aa2** (94 mg, 41%) after purification by column chromatography on silica gel (EtOAc/PE = 1: 120). Reaction time 6 h.

Colorless viscous liquid. **^1^H NMR** (500 MHz, CDCl_3_): δ 0.88 (t, *J* = 7.5 Hz, 3H), 1.26–1.31 (m, 16H), 1.43–1.45 (m, 2H), 1.67–1.70 (m, 2H), 1.79 (s, 3H), 2.19 (s, 6H), 2.96 (t, *J* = 7.5 Hz, 2H), 7.08 (s, 1H), 7.31 (d, *J* = 8.5 Hz, 1H), 7.62 (d, *J* = 8.5 Hz, 1H). **^13^C NMR** (125 MHz, CDCl_3_): δ 14.1, 22.5, 22.7, 27.9, 28.7, 28.8, 29.1, 29.3, 29.4, 29.5, 29.6 (2), 31.9, 32.2, 70.5, 124.7 (CF_3_, q, *J* = 272.0 Hz), 125.0 (CF_3_, q, *J* = 31.1 Hz), 125.6, 127.8, 129.2 (q, *J* = 5.2 Hz), 138.4, 143.7. **^19^F NMR** (470 MHz, CDCl_3_)  δ  -57.3. **HRMS** (ESI-TOF) Calcd for C_25_H_37_F_3_NaO_2_S (M+H)^+^ 481.2359. Found 481.2373.

**3’a**: 2-(5-(benzylthio)-2-(trifluoromethyl)phenyl)pentan-3-one

Following the procedure for the synthesis of **3aa**, the reaction of 4-(trifluoromethyl)-4-((trimethylsilyl)oxy)cyclohexa-2,5-dienone **1a** (150 mg, 0.60 mmol), phenylmethanethiol (57 μL, 0.5 mmol) and pentan-3-one **2c** (157 μL, 1.5 mmol) gave **3’a** (142 mg, 87%) after purification by column chromatography on silica gel (EtOAc/PE = 1: 120). Reaction time 6 h.

Colorless viscous liquid. **^1^H NMR** (500 MHz, CDCl_3_): δ 0.95 (t, *J* = 7.5 Hz, 3H), 1.31 (d, *J* = 7.0 Hz, 3H), 2.21–2.36 (m, 2H), 4.11 (q, *J* = 7.0 Hz, 1H), 4.14 (s, 2H), 7.11 (s, 1H), 7.19 (d, *J* = 8.5 Hz, 1H), 7.23 (t, *J* = 7.5 Hz, 1H), 7.28 (t, *J* = 7.5 Hz, 2H), 7.33 (d, *J* = 7.5 Hz, 2H), 7.50 (d, *J* = 8.5 Hz, 1H). **^13^C NMR** (125 MHz, CDCl_3_): δ 7.8, 18.2, 34.5, 37.1, 47.4, 124.8 (CF_3_, q, *J* = 271.5 Hz), 125.2 (q, *J* = 29.5 Hz), 125.8, 126.4 (q, *J* = 5.6 Hz), 127.2, 127.4, 128.5, 128.6, 136.1, 140.1, 142.7, 209.9. **^19^F NMR** (470 MHz, CDCl_3_)  δ  -60.5. **HRMS** (ESI-TOF) Calcd for C_19_H_19_F_3_NaOS (M+Na) ^+^ 375.1001. Found 375.1000.

**3’b**: 3-(5-(benzylthio)-2-(trifluoromethyl)phenyl)-4-hydroxypent-3-en-2-one

Following the procedure for the synthesis of **3aa**, the reaction of 4-(trifluoromethyl)-4-((trimethylsilyl)oxy)cyclohexa-2,5-dienone **1a** (150 mg, 0.60 mmol), phenylmethanethiol (57 μL, 0.5 mmol) and pentane-2,4-dione **2y** (154 μL, 1.5 mmol) gave **3’b** (118 mg, 65%) after purification by column chromatography on silica gel (EtOAc/PE = 1: 120). Reaction time 6 h.

White solid, m.p. 69–70 ^o^C. **^1^H NMR** (500 MHz, CDCl_3_): δ 1.71 (s, 6H), 4.20 (s, 2H), 7.10 (s, 1H), 7.25 (d, *J* = 8.5 Hz, 1H), 7.29 (t, *J* = 7.5 Hz, 2H), 7.33–7.37 (m, 3H), 7.60 (d, *J* = 8.5 Hz, 1H), 16.6 (s, 1H). **^13^C NMR** (125 MHz, CDCl_3_): δ 23.9, 37.3, 111.2, 123.9 (CF_3_, q, *J* = 271.5 Hz), 126.9 (q, *J* = 5.4 Hz), 127.3 (q, *J* = 28.4 Hz), 127.4, 127.6, 128.6, 128.7, 132.4, 135.9, 142.6, 190.6. **^19^F NMR** (470 MHz, CDCl_3_)  δ  -62.7. **HRMS** (ESI-TOF) Calcd for C_19_H_18_F_3_O_2_S (M+H) ^+^ 367.0974. Found 367.0964.

**3ba**: 1-(4-(dodecylthio)-1-(trifluoromethyl)naphthalen-2-yl)propan-2-one

Following the procedure for the synthesis of **3aa**, the reaction of 4-(trifluoromethyl)-4-((trimethylsilyl)oxy)naphthalen-1(4*H*)-one **1b** (180 mg, 0.60 mmol), dodecane-1-thiol (120 μL, 0.5 mmol) and propan-2-one **2a** (111 μL, 1.5 mmol) gave **3ba** (104 mg, 46%) after purification by column chromatography on silica gel (EtOAc/PE = 1: 120). Reaction time 6 h.

White solid, m.p. 60–61 ^o^C. **^1^H NMR** (500 MHz, CDCl_3_): δ 0.88 (t, *J* = 7.0 Hz, 3H), 1.26–1.31 (m, 16H), 1.45–1.48 (m, 2H), 1.70–1.74 (m, 2H), 2.25 (s, 3H), 3.01 (t, *J* = 7.5 Hz, 2H), 4.07 (s, 2H), 7.04 (s, 1H), 7.53–7.60 (m, 2H), 8.21 (d, *J* = 8.0 Hz, 1H), 8.33 (d, *J* = 8.0 Hz, 1H). **^13^C NMR** (125 MHz, CDCl_3_): δ 14.1, 22.7, 28.4, 28.9, 29.1, 29.3, 29.4, 29.5, 29.6, 29.6, 31.9, 32.7, 50.6, 121.5 (CF_3_, q, *J* = 28.4 Hz), 124.6, 125.3 (CF_3_, q, *J* = 5.5 Hz), 126.2, 127.3, 127.7 (CF_3_, q, *J* = 274.1 Hz), 127.8, 130.3, 131.3, 131.5, 141.2, 204.3. **^19^F NMR** (470 MHz, CDCl_3_)  δ  -53.5. **HRMS** (ESI-TOF) Calcd for C_26_H_36_F_3_OS (M+H) ^+^ 453.2433. Found 453.2444.

**3bc**: 2-(4-(dodecylthio)-1-(trifluoromethyl)naphthalen-2-yl)pentan-3-one

Following the procedure for the synthesis of **3aa**, the reaction of 4-(trifluoromethyl)-4-((trimethylsilyl)oxy)naphthalen-1(4*H*)-one **1b** (180 mg, 0.60 mmol), dodecane-1-thiol (120 μL, 0.5 mmol) and pentan-3-one **2c** (157 μL, 1.5 mmol) gave **3bc** (170 mg, 71%) after purification by column chromatography on silica gel (EtOAc/PE = 1: 120). Reaction time 6 h.

Colorless viscous liquid. **^1^H NMR** (500 MHz, CDCl_3_): δ 0.88 (t, *J* = 7.0 Hz, 3H), 0.98 (t, *J* = 7.0 Hz, 3H), 1.26–1.30 (m, 16H), 1.47–1.50 (m, 5H), 1.68–1.74 (m, 2H), 2.28–2.40 (m, 2H), 3.01 (t, *J* = 7.5 Hz, 2H), 4.54 (q, *J* = 7.0 Hz, 1H), 7.07 (s, 1H), 7.55 (t, *J* = 8.0 Hz, 1H), 7.59 (t, *J* = 8.0 Hz, 1H), 8.31 (d, *J* = 8.0 Hz, 2H). **^13^C NMR** (125 MHz, CDCl_3_): δ 7.9, 14.1, 17.9, 22.7, 28.5, 28.9, 29.2, 29.3, 29.4, 29.5, 29.6 (2), 31.9, 32.2, 34.6, 49.1, 120.4 (CF_3_, q, *J* = 28.4 Hz), 121.9, 124.6, 125.3 (CF_3_, q, *J* = 5.5 Hz), 125.8 (CF_3_, q, *J* = 274.1 Hz), 126.2, 127.9, 130.3, 130.8, 138.7, 141.8, 210.5. **HRMS** (ESI-TOF) Calcd for C_28_H_40_F_3_OS (M+H) + 481.2746. Found 481.2737.

**3cc**: 2-(3-(tert-butyl)-5-(dodecylthio)-2-(trifluoromethyl)phenyl)pentan-3-one

Following the procedure for the synthesis of **3aa**, the reaction of 3-(tert-butyl)-4-(trifluoromethyl)-4-((trimethylsilyl)oxy)cyclohexa-2,5-dienone **1c** (184 mg, 0.60 mmol), dodecane-1-thiol (120 μL, 0.5 mmol) and pentan-3-one **2c** (157 μL, 1.5 mmol) gave **3cc** (189 mg, 78%) after purification by column chromatography on silica gel (EtOAc/PE = 1: 120). Reaction time 6 h.

Colorless viscous liquid. **^1^H NMR** (500 MHz, CDCl_3_): δ 0.88 (t, *J* = 7.0 Hz, 3H), 0.96 (t, *J* = 7.5 Hz, 3H), 1.26–1.30 (m, 16H), 1.38 (d, *J* = 7.0 Hz, 3H), 1.42–1.46 (m, 11H), 1.63–1.68 (m, 2H), 2.27–2.32 (m, 2H), 2.91 (t, *J* = 7.5 Hz, 2H), 4.18 (q, *J* = 7.0 Hz, 1H), 6.89 (s, 1H), 7.37 (s, 1H). **^13^C NMR** (125 MHz, CDCl_3_): δ 7.9, 14.0, 18.6, 22.6, 28.8, 29.1, 29.3, 29.4, 29.5, 29.6, 29.6, 31.8, 31.9, 32,4, 32.5, 34.2, 37.2, 49.3, 123.9 (CF_3_, q, *J* = 29.5 Hz), 124.0, 125.4, 125.5 (CF_3_, q, *J* = 271.5 Hz), 141.8, 142.9 (q, *J* = 5.6 Hz), 150.6, 210.4. **^19^F NMR** (470 MHz, CDCl_3_)  δ  -48.1. **HRMS** (ESI-TOF) Calcd for C_28_H_46_F_3_OS (M+H) ^+^ 487.3216. Found 487.3206.

**3dc**: 2-(5-(dodecylthio)-3-methyl-2-(trifluoromethyl)phenyl)pentan-3-one

Following the procedure for the synthesis of **3aa**, the reaction of 3-methyl-4-(trifluoromethyl)-4-((trimethylsilyl)oxy)cyclohexa-2,5-dienone **1d** (158 mg, 0.60 mmol), dodecane-1-thiol (120 μL, 0.5 mmol) and pentan-3-one **2c** (157 μL, 1.5 mmol) gave **3dc** (133 mg, 60%) after purification by column chromatography on silica gel (EtOAc/PE = 1: 120). Reaction time 6 h.

Colorless viscous liquid. **^1^H NMR** (500 MHz, CDCl_3_): δ 0.88 (t, *J* = 7.0 Hz, 3H), 0.98 (t, *J* = 7.5 Hz, 3H), 1.26–1.30 (m, 16H), 1.36 (d, *J* = 7.0 Hz, 3H), 1.41–1.44 (m, 2H), 1.61–1.67 (m, 2H), 2.30–2.35 (m, 2H), 2.48 (q, *J* = 4.0 Hz, 3H), 2.90 (t, *J* = 7.5 Hz, 2H), 4.18 (q, *J* = 7.0 Hz, 1H), 6.86 (s, 1H), 6.99 (s, 1H). **^13^C NMR** (125 MHz, CDCl_3_): δ 7.9, 14.0, 18.3, 22.2 (q, *J* = 4.8 Hz), 22.6, 28.7, 28.8, 29.1, 29.3, 29.4, 29.5, 29.6, 29.6, 31.7, 31.9, 34.3, 48.4, 123.5 (CF_3_, q, *J* = 29.5 Hz), 124.3, 125.8 (CF_3_, q, *J* = 274.2 Hz), 129.1, 137.8, 141.3 (q, *J* = 5.6 Hz), 142.4, 210.4. **^19^F NMR** (470 MHz, CDCl_3_)  δ  -59.9. **HRMS** (ESI-TOF) Calcd for C_19_H_39_F_3_NaOS (M+Na)^+^ 467.2566. Found 467.2574.

**3ec**: 2-(5-(dodecylthio)-2-(perfluoroethyl)phenyl)pentan-3-one

Following the procedure for the synthesis of **3aa**, the reaction of 4-(perfluoroethyl)-4-((trimethylsilyl)oxy)cyclohexa-2,5-dienone **1e** (180 mg, 0.60 mmol), dodecane-1-thiol (120 μL, 0.5 mmol) and pentan-3-one **2c** (157 μL, 1.5 mmol) gave **3ec** (206 mg, 86%) after purification by column chromatography on silica gel (EtOAc/PE = 1: 120). Reaction time 6 h.

Colorless viscous liquid. **^1^H NMR** (500 MHz, CDCl_3_): δ 0.88 (t, *J* = 7.0 Hz, 3H), 0.99 (t, *J* = 7.0 Hz, 3H), 1.26–1.30 (m, 16H), 1.37 (d, *J* = 7.0 Hz, 3H), 1.41–1.46 (m, 2H), 1.64–1.70 (m, 2H), 2.37 (q, *J* = 7.0 Hz, 2H), 2.94 (t, *J* = 7.5 Hz, 2H), 4.17 (q, *J* = 7.0 Hz, 1H), 7.08 (s, 1H), 7.21 (d, *J* = 8.5 Hz, 1H), 7.47 (d, *J* = 8.5 Hz, 1H). **^13^C NMR** (125 MHz, CDCl_3_): δ 7.8, 14.0, 18.6, 22.6, 28.6, 28.8, 29.1, 29.3, 29.4, 29.5, 29.6 (2), 31.8, 31.9, 34.4, 47.7, 114.8 (qt, *J*_1_ = 253.1 Hz, *J*_2_ = 38.6 Hz), 120.2 (tq, *J*_1_ = 249.4 Hz, *J*_2_ = 39.4 Hz), 122.3 (t, *J* = 22.0 Hz), 124.9, 126.6, 128.6 (t, *J* = 8.9 Hz), 141.2, 144.1, 210.1. **^19^F NMR** (470 MHz, CDCl_3_) δ  -85.9 (s, 3F), -107.8 (d, *J* = 274.5 Hz, 1F), -110.1 (d, *J* = 274.5 Hz, 1F). **HRMS** (ESI-TOF) Calcd for C_25_H_38_F_5_OS (M+H) ^+^ 481.2558. Found 481.2554.

**10a**: (4-(trifluoromethyl)-1,3-phenylene)bis(dodecylsulfane)

Colorless viscous liquid. **^1^H NMR** (500 MHz, CDCl_3_): δ 0.88 (t, *J* = 7.0 Hz, 6H), 1.26–1.31 (m, 32H), 1.44–1.46 (m, 4H), 1.66–1.72 (m, 4H), 2.94 (t, *J* = 7.5 Hz, 2H), 2.95 (t, *J* = 7.5 Hz, 2H), 7.26 (d, *J* = 8.0 Hz, 1H), 7.35 (d, *J* = 8.0 Hz, 1H), 7.45 (s, 1H). **^13^C NMR** (125 MHz, CDCl_3_): δ 14.1, 22.7, 28.5, 28.6, 28.8, 28.9, 29.1, 29.2, 29.3, 29.4 (2), 29.5, 29.6 (2), 31.9, 32.6, 33.3, 122.5 (q, *J* = 3.8 Hz), 124.4 (CF_3_, q, *J* = 271.4 Hz), 124.8 (q, *J* = 3.8 Hz), 126.1, 127.3 (q, *J* = 30.1 Hz), 136.9, 142.8. **HRMS** (ESI-TOF) Calcd for C_31_H_54_F_3_S_2_ (M+H) ^+^ 547.3614. Found 547.3618.

**Three-Component, Double Nucleophilic Carbothiolation/Aromatization Reactions Using Electron-Rich Arenes as C-Nucleophiles.**

**5aa**: dodecyl(2',4',6'-trimethoxy-6-(trifluoromethyl)-[1,1'-biphenyl]-3-yl)sulfane

**5aa’**: dodecyl(2',4',6'-trimethoxy-5-(trifluoromethyl)-[1,1'-biphenyl]-2-yl)sulfane

To the solution of 4-(trifluoromethyl)-4-((trimethylsilyl)oxy)cyclohexa-2,5-dienone **1a** (150 mg, 0.60 mmol) and 1,3,5-trimethoxybenzene **4a** (252 mg, 1.5 mmol) in DCE (1 mL) was added TMSCl (126 μL, 1 mmol) and In(OTf)_3_ (85 mg, 0.15 mmol). The solution was heated to 60 ^o^C and DCE solution (2 mL) of dodecane-1-thiol (120 μL, 0.5 mmol) was added dropwise within 40min. After the reaction was finished as indicated by TLC (reaction time, 4 h), the resulting mixture was poured into water (20 mL) and extracted with DCM (CH_2_Cl_2_, 20 mL×3). The combined organic layer was dried over anhydrous Na_2_SO_4_ and concentrated *in vacuo*. The crude product was purified by column chromatography on silica gel (EtOAc/PE = 1: 120) to afford **5aa/5aa’** (230 mg, 90%) and unisolated **4a** (113 mg, 45%).

Colorless viscous liquid, **5aa/5aa’** = 1.0/1.2.

**^1^H NMR** (500 MHz, CDCl_3_):

**5aa**, δ 0.88 (t, *J* = 7.0 Hz, 3H), 1.25–1.29 (m, 16H), 1.39–1.41 (m, 2H), 1.64–1.69 (m, 2H), 2.92 (t, *J* = 7.5 Hz, 2H), 3.64 (s, 6H), 3.81 (s, 3H), 6.17 (s, 2H), 7.07 (s, 1H), 7.24 (d, *J* = 8.0 Hz, 1H), 7.57 (d, *J* = 8.0 Hz, 1H).

**5aa’**, δ 0.88 (t, *J* = 7.0 Hz, 3H), 1.25–1.29 (m, 16H), 1.32–1.35 (m, 2H), 1.54–1.59 (m, 2H), 2.81 (t, *J* = 7.5 Hz, 2H), 3.67 (s, 6H), 3.82 (s, 3H), 6.20 (s, 2H), 7.35–7.36 (m, 2H), 7.47 (d, *J* = 8.5 Hz, 1H).

**^13^C NMR** (125 MHz, CDCl_3_): δ 14.0, 22.6, 28.5, 28.6, 28.7, 28.8, 29.1, 29.3, 29.4 (2), 29.5, 29.6 (2), 31.8, 32.0, 32.2, 55.4, 55.6, 90.1, 90.6, 109.2, 109.3, 123.9 (q, *J* = 3.8 Hz), 124.1 (CF_3_, q, *J* = 272.0 Hz), 124.5 (CF_3_, q, *J* = 272.0 Hz), 125.2, 125.6, 126.2 (q, *J* = 3.8 Hz), 126.6 (q, *J* = 30.0 Hz), 128.3 (q, *J* = 3.8 Hz), 131.1, 134.4, 134.6, 141.7, 143.9, 158.3, 158.4, 161.3, 161.5. **^19^F NMR** (470 MHz, CDCl_3_)  δ  -63.8, -63.0.

**HRMS** (ESI-TOF) Calcd for C_28_H_40_F_3_O_3_S (M + H)^+^ 513.2645. Found 513.2646.

**5ba**: benzyl(2',4',6'-trimethoxy-6-(trifluoromethyl)-[1,1'-biphenyl]-3-yl)sulfane

**5ba’**: benzyl(2',4',6'-trimethoxy-5-(trifluoromethyl)-[1,1'-biphenyl]-2-yl)sulfane

Following the procedure for the synthesis of **5aa/5aa’**, the reaction of 4-(trifluoromethyl)-4-((trimethylsilyl)oxy)cyclohexa-2,5-dienone **1a** (150 mg, 0.60 mmol), phenylmethanethiol (59 μL, 0.5 mmol) and 1,3,5-trimethoxybenzene **4a** (252 mg, 1.5 mmol) gave **5ba/5ba’** (191 mg, 88%) after purification by column chromatography on silica gel (EtOAc/PE = 2: 150). Reaction time 4 h.

White solid, m.p. 119–122 ^o^C. **5ba/5ba’** = 0.7/1.0.

**^1^H NMR** (500 MHz, CDCl_3_):

**5ba**, δ 3.64 (s, 6H), 3.83 (s, 3H), 4.14 (s, 2H), 6.17 (s, 2H), 7.14 (s, 1H), 7.18–7.28 (m, 5H), 7.32 (d, *J* = 8.0 Hz, 1H), 7.56 (d, *J* = 8.0 Hz, 1H).

**5ba’**, δ 3.70 (s, 6H), 3.84 (s, 3H), 4.05 (s, 2H), 6.20 (s, 2H), 7.21–7.29 (m, 5H), 7.33–7.37 (m, 2H), 7.44 (d, *J* = 8.0 Hz, 1H).

**^13^C NMR** (125 MHz, CDCl_3_): δ 37.1, 37.5, 55.3, 55.6, 55.8, 90.2, 90.7, 109.0, 109.1, 123.9 (CF_3_, q, *J* = 270.4 Hz), 124.1 (q, *J* = 3.8 Hz), 124.3 (CF_3_, q, *J* = 270.4 Hz), 125.9, 126.3, 126.4 (q, *J* = 3.8 Hz), 126.9 (q, *J* = 32.3 Hz), 127.2, 127.3, 126.9 (q, *J* = 30.0 Hz), 128.4, 128.5, 128.8, 131.9, 134.5, 134.6, 136.5, 136.6, 141.1, 143.3, 158.4, 158.5, 161.3, 161.6.

**HRMS** (ESI-TOF) Calcd for C_23_H_22_F_3_O_3_S (M+H) ^+^ 435.1563. Found 435.1565.

The single crstal of **5ba’** was obtained, the X-ray diffraction analysis is on page S33 and its **^1^H NMR** and **^13^C NMR** data is as follows.

**5ba’**, White solid, m.p. 136–138 ^o^C. **^1^H NMR** (500 MHz, CDCl_3_): δ 3.70 (s, 6H), 3.84 (s, 3H), 4.05 (s, 2H), 6.20 (s, 2H), 7.21–7.29 (m, 5H), 7.33–7.37 (m, 2H), 7.44 (d, *J* = 8.0 Hz, 1H). **^13^C NMR** (125 MHz, CDCl_3_): δ 37.1, 55.3, 55.8, 90.7, 109.1, 124.1 (q, *J* = 3.8 Hz), 124.3 (CF_3_, q, *J* = 270.4 Hz), 126.3, 126.9 (q, *J* = 32.3 Hz), 127.2, 128.4, 128.5, 128.8, 134.6, 136.6, 143.3, 158.4, 161.6.

**5ab**: dodecyl(4'-methoxy-6-(trifluoromethyl)-[1,1'-biphenyl]-3-yl)sulfane

**5ab’**: dodecyl(4'-methoxy-5-(trifluoromethyl)-[1,1'-biphenyl]-2-yl)sulfane

Following the procedure for the synthesis of **5aa/5aa’**, the reaction of 4-(trifluoromethyl)-4-((trimethylsilyl)oxy)cyclohexa-2,5-dienone **1a** (150 mg, 0.60 mmol), dodecane-1-thiol (120 μL, 0.5 mmol) and anisole **4b** (163 μL, 1.5 mmol) gave **5ab/5ab’** (106 mg, 47%) and a small amount of an unknown compound after purification by column chromatography on silica gel (EtOAc/PE = 1: 120). Reaction time 6 h.

Colorless viscous liquid, **5ab/5ab’** = 1.0/0.8.

**^1^H NMR** (500 MHz, CDCl_3_):

**5ab**, δ 0.88 (t, *J* = 7.0 Hz, 3H), 1.25–1.29 (m, 16H), 1.39–1.44 (m, 2H), 1.67–1.70 (m, 2H), 2.96 (t, *J* = 7.5 Hz, 2H), 3.84 (s, 3H), 6.97 (d, *J* = 9.0 Hz, 2H), 7.16 (s, 1H), 7.27 (d, *J* = 8.0 Hz, 1H), 7.34 (d, *J* = 9.0 Hz, 2H), 7.59 (d, *J* = 8.0 Hz, 1H).

**5ab’**, δ 0.88 (t, *J* = 7.0 Hz, 3H), 1.25–1.29 (m, 16H), 1.36–1.39 (m, 2H), 1.59–1.63 (m, 2H), 2.84 (t, *J* = 7.5 Hz, 2H), 3.85 (s, 3H), 6.93 (d, *J* = 8.5 Hz, 2H), 7.25 (d, *J* = 8.5 Hz, 2H), 7.35 (d, *J* = 8.0 Hz, 1H), 7.42 (s, 1H), 7.49 (d, *J* = 8.0 Hz, 1H).

**^13^C NMR** (125 MHz, CDCl_3_): δ 14.1, 22.7, 28.4, 28.7, 28.8, 29.0, 29.1, 29.3, 29.4 (2), 29.5, 29.6, 31.9, 32.1, 32.4, 55.2 (2), 110.5, 113.2, 113.7, 119.8, 124.0 (q, *J* = 3.8 Hz), 124.5 (CF_3_, q, *J* = 275.0 Hz), 124.9, 125.5, 126.4 (q, *J* = 3.8 Hz), 126.7 (q, *J* = 3.8 Hz), 129.6 (q, *J* = 43.7 Hz), 129.7, 129.9, 130.1, 130.4, 131.6, 131.8, 140.9, 141.5, 141.8, 142.3, 159.2, 159.4. **^19^F NMR** (470 MHz, CDCl_3_)  δ  -64.1, -58.5.

**HRMS** (ESI-TOF) Calcd for C_26_H_36_F_3_OS (M + H)^+^ 453.2433. Found 453.2439.

**5ac**: dodecyl(2'-methoxy-5'-methyl-6-(trifluoromethyl)-[1,1'-biphenyl]-3-yl)sulfane

**5ac’**: dodecyl(2'-methoxy-5'-methyl-5-(trifluoromethyl)-[1,1'-biphenyl]-2-yl)sulfane

Following the procedure for the synthesis of **5aa/5aa’**, the reaction of 4-(trifluoromethyl)-4-((trimethylsilyl)oxy)cyclohexa-2,5-dienone **1a** (150 mg, 0.60 mmol), dodecane-1-thiol (120 μL, 0.5 mmol) and 1-methoxy-4-methylbenzene **4c** (188 μL, 1.5 mmol) gave **5ac/5ac’** (163 mg, 70%) after purification by column chromatography on silica gel (EtOAc/PE = 1: 120). Reaction time 6 h.

Colorless viscous liquid, **5ac/5ac’** = 1.0/0.4.

**^1^H NMR** (500 MHz, CDCl_3_):

**5ac**, δ 0.88 (t, *J* = 7.0 Hz, 3H), 1.24–1.29 (m, 16H), 1.40–1.44 (m, 2H), 1.66–1.69 (m, 2H), 2.30 (s, 3H), 2.94 (t, *J* = 7.5 Hz, 2H), 3.69 (s, 3H), 6.83 (d, *J* = 8.5 Hz, 1H), 6.95 (s, 1H), 7.12 (s, 1H), 7.14 (d, *J* = 8.5 Hz, 1H), 7.28 (d, *J* = 8.5 Hz, 1H), 7.58 (d, *J* = 8.5 Hz, 1H).

**5ac’**, δ 0.88 (t, *J* = 7.0 Hz, 3H), 1.24–1.29 (m, 16H), 1.31–1.35 (m, 2H), 1.55–1.59 (m, 2H), 2.32 (s, 3H), 2.84 (t, *J* = 7.5 Hz, 2H), 3.73 (s, 3H), 6.87 (d, *J* = 8.5 Hz, 1H), 6.97 (s, 1H), 7.18 (d, *J* = 8.5 Hz, 1H), 7.37 (d, *J* = 8.0 Hz, 1H), 7.41 (s, 1H), 7.51 (d, *J* = 8.0 Hz, 1H).

**^13^C NMR** (125 MHz, CDCl_3_): δ 14.1, 20.3, 20.4, 22.7, 28.5, 28.7, 28.8, 28.9, 29.1, 29.3, 29.4, 29.5, 29.6, 31.9, 32.1, 32.5, 55.5, 55.7, 110.4, 110.9, 124.0 (CF_3_, q, *J* = 271.8 Hz), 124.2 (q, *J* = 3.8 Hz), 124.6 (CF_3_, q, *J* = 268.8 Hz), 125.3, 125.7 (q, *J* = 30.0 Hz), 126.3 (q, *J* = 5.0 Hz), 127.9 (2), 128.9, 129.6 (2), 130.0, 131.2, 131.4, 138.1, 138.6, 141.9, 142.7, 154.6. **^19^F NMR** (470 MHz, CDCl_3_)  δ  -64.0, -60.8.

**HRMS** (ESI-TOF) Calcd for C_27_H_38_F_3_OS (M + H)^+^ 467.2590. Found 467.2596.

**5ad**: 5'-(dodecylthio)-6-methoxy-2'-(trifluoromethyl)-[1,1'-biphenyl]-3-ol

**5ad’**: 2'-(dodecylthio)-6-methoxy-5'-(trifluoromethyl)-[1,1'-biphenyl]-3-ol

Following the procedure for the synthesis of **5aa/5aa’**, the reaction of 4-(trifluoromethyl)-4-((trimethylsilyl)oxy)cyclohexa-2,5-dienone **1a** (150 mg, 0.60 mmol), dodecane-1-thiol (120 μL, 0.5 mmol) and 4-methoxyphenol **4d** (186 mg, 1.5 mmol) gave **5ad/5ad’** (152 mg, 65%) after purification by column chromatography on silica gel (EtOAc/PE = 2: 150). Reaction time 5 h.

Colorless viscous liquid, **5ad/5ad’** = 1.0/0.7.

**^1^H NMR** (500 MHz, CDCl_3_):

**5ad**, δ 0.88 (t, *J* = 7.0 Hz, 3H), 1.25–1.29 (m, 16H), 1.41–1.44 (m, 2H), 1.66–1.70 (m, 2H), 2.96 (t, *J* = 7.5 Hz, 2H), 3.75 (s, 3H), 4.46 (s, 1H), 6.69 (d, *J* = 9.0 Hz, 1H), 6.85 (s, 1H), 6.88 (d, *J* = 9.0 Hz, 1H), 7.19 (s, 1H), 7.33 (d, *J* = 8.0 Hz, 1H), 7.65 (d, *J* = 8.0 Hz, 1H).

**5ab’**, δ 0.88 (t, *J* = 7.0 Hz, 3H), 1.25–1.29 (m, 16H), 1.37–1.41 (m, 2H), 1.62–1.66 (m, 2H), 2.87 (t, *J* = 7.5 Hz, 2H), 3.77 (s, 3H), 4.79 (s, 1H), 6.69 (d, *J* = 8.5 Hz, 1H), 6.85 (s, 1H), 6.93 (d, *J* = 8.5 Hz, 1H), 7.39 (d, *J* = 8.0 Hz, 1H), 7.47 (s, 1H), 7.58 (d, *J* = 8.0 Hz, 1H).

**^13^C NMR** (125 MHz, CDCl_3_): δ 14.1, 22.7, 28.4, 28.6, 28.8, 28.9, 29.1, 29.3, 29.4 (2), 29.5, 29.6, 29.7, 31.8, 31.9, 55.7, 115.4, 115.6, 115.8, 116.3, 117.7, 123.8 (CF_3_, q, *J* = 271.8 Hz), 125.3 (q, *J* = 3.8 Hz), 126.0, 126.9 (q, *J* = 5.0 Hz), 127.2 (q, *J* = 32.4 Hz), 127.8 (q, *J* = 3.8 Hz), 129.8, 136.0, 136.4, 142.6, 143.6, 146.3, 146.5, 152.8, 153.8. **^19^F NMR** (470 MHz, CDCl_3_)  δ  -64.2, -60.7.

**HRMS** (ESI-TOF) Calcd for C_26_H_36_F_3_O_2_S (M + H)^+^ 469.2383. Found 469.2372.

The structure of **5ad**/**5ad’** was also confirmed by Nuclear Overhauser Enhancement Spectroscopy (NOESY), please see pS31.

**5ae**: 5'-(dodecylthio)-5-methyl-2'-(trifluoromethyl)-[1,1'-biphenyl]-2-ol

**5ae’**: 2'-(dodecylthio)-5-methyl-5'-(trifluoromethyl)-[1,1'-biphenyl]-2-ol

Following the procedure for the synthesis of **5aa/5aa’**, the reaction of 4-(trifluoromethyl)-4-((trimethylsilyl)oxy)cyclohexa-2,5-dienone **1a** (150 mg, 0.60 mmol), dodecane-1-thiol (120 μL, 0.5 mmol) and p-cresol **4e** (162 mg, 1.5 mmol) gave **5ae/5ae’** (176 mg, 78%) after purification by column chromatography on silica gel (EtOAc/PE = 1: 120). Reaction time 6 h.

Colorless viscous liquid, **5ae/5ae’** = 1.0/0.8.

**^1^H NMR** (500 MHz, CDCl_3_):

**5ae**, δ 0.88 (t, *J* = 7.0 Hz, 3H), 1.25–1.29 (m, 16H), 1.41–1.44 (m, 2H), 1.66–1.69 (m, 2H), 2.28 (s, 3H), 2.94 (t, *J* = 7.5 Hz, 2H), 4.62 (s, 1H), 6.80 (d, *J* = 8.0 Hz, 1H), 6.92 (s, 1H), 7.07 (d, *J* = 8.0 Hz, 1H), 7.17 (s, 1H), 7.31 (d, *J* = 8.0 Hz, 1H), 7.63 (d, *J* = 8.0 Hz, 1H).

**5ae’**, δ 0.88 (t, *J* = 7.0 Hz, 3H), 1.25–1.29 (m, 16H), 1.38–1.41 (m, 2H), 1.61–1.66 (m, 2H), 2.31 (s, 3H), 2.86 (t, *J* = 7.5 Hz, 2H), 4.93 (s, 1H), 6.87 (d, *J* = 8.0 Hz, 1H), 6.94 (s, 1H), 7.11 (d, *J* = 8.0 Hz, 1H), 7.37 (d, *J* = 8.0 Hz, 1H), 7.46 (s, 1H), 7.56 (d, *J* = 8.0 Hz, 1H).

**^13^C NMR** (125 MHz, CDCl_3_): δ 14.1, 20.3, 20.4, 22.7, 28.3, 28.5, 28.8, 28.9, 29.1, 29.3, 29.4 (2), 29.5, 29.6, 31.9, 115.3, 116.4, 123.5 (CF_3_, q, *J* = 271.8 Hz), 124.5 (CF_3_, q, *J* = 271.8 Hz), 125.1, 125.3, 125.7, 125.8, 126.1 (q, *J* = 30.0 Hz), 126.7 (q, *J* = 5.0 Hz), 127.1 (q, *J* = 32.5 Hz), 127.8 (q, *J* = 3.8 Hz), 129.1, 129.9, 130.2, 130.3, 130.7, 130.8, 130.9, 136.2, 136.4, 142.8, 143.4, 150.1, 150.3. **^19^F NMR** (470 MHz, CDCl_3_)  δ  -64.1, -60.7.

**HRMS** (ESI-TOF) Calcd for C_26_H_36_F_3_OS (M + H)^+^ 453.2433. Found 453.2434.

**5bf**: benzyl(2',5'-dimethoxy-6-(trifluoromethyl)-[1,1'-biphenyl]-3-yl)sulfane

**5bf’**: benzyl(2',5'-dimethoxy-5-(trifluoromethyl)-[1,1'-biphenyl]-2-yl)sulfane

Following the procedure for the synthesis of **5aa/5aa’**, the reaction of 4-(trifluoromethyl)-4-((trimethylsilyl)oxy)cyclohexa-2,5-dienone **1a** (150 mg, 0.60 mmol), phenylmethanethiol (59 μL, 0.5 mmol) and 1,4-dimethoxybenzene **4f** (207 mg, 1.5 mmol) gave **5bf/5bf’** (101 mg, 50%) after purification by column chromatography on silica gel (EtOAc/PE = 1: 120). Reaction time 5 h.

Colorless viscous liquid, **5bf/5bf’** = 1.0/0.3.

**^1^H NMR** (500 MHz, CDCl_3_):

**5bf**, δ 3.65 (s, 3H), 3.75 (s, 3H), 4.16 (s, 2H), 6.69 (d, *J* = 6.5 Hz, 1H), 6.86 (s, 1H), 6.88 (d, *J* = 6.5 Hz, 1H), 7.18 (s, 1H), 7.24–7.26 (m, 2H), 7.28–7.31 (m, 3H), 7.34 (d, *J* = 8.5 Hz, 1H), 7.57 (d, *J* = 8.5 Hz, 1H).

**5bf’**, δ 3.71 (s, 3H), 3.76 (s, 3H), 4.05 (s, 2H), 6.71 (d, *J* = 6.5 Hz, 1H), 6.84 (s, 1H), 6.89 (d, *J* = 6.5 Hz, 1H), 7.24–7.26 (m, 1H), 7.28–7.31 (m, 1H), 7.33–7.34 (m, 3H), 7.38 (d, *J* = 8.0 Hz, 1H), 7.43 (s, 1H), 7.48 (d, *J* = 8.0 Hz, 1H).

**^13^C NMR** (125 MHz, CDCl_3_): δ 37.3, 37.5, 55.7, 55.9, 56.1, 110.6, 112.1, 113.9, 114.3, 116.5, 124.2 (CF_3_, q, *J* = 272.0 Hz), 124.5 (q, *J* = 3.8 Hz), 126.1, 126.4 (q, *J* = 5.0 Hz), 126.7, 127.3, 127.4, 128.4, 128.6, 128.8, 130.6, 136.2, 137.7, 138.4, 141.3, 142.1, 150.9, 152.7, 153.3. **^19^F NMR** (470 MHz, CDCl_3_)  δ  -64.0, -60.7.

**HRMS** (ESI-TOF) Calcd for C_22_H_19_F_3_NaO_2_S (M+Na) ^+^ 427.0950. Found 427.0960.

**5ag**: dodecyl(2',4',6'-trimethyl-6-(trifluoromethyl)-[1,1'-biphenyl]-3-yl)sulfane

Following the procedure for the synthesis of **5aa/5aa’**, the reaction of 4-(trifluoromethyl)-4-((trimethylsilyl)oxy)cyclohexa-2,5-dienone **1a** (150 mg, 0.60 mmol), dodecane-1-thiol (120 μL, 0.5 mmol) and mesitylene **4g** (1.379 mL, 10 mmol) gave **5ag** (97 mg, 42%) after purification by column chromatography on silica gel (eluent: petroleum ether). Reaction time 6 h.

Colorless viscous liquid. **^1^H NMR** (500 MHz, CDCl_3_): δ 0.88 (t, *J* = 7.0 Hz, 3H), 1.25–1.31 (m, 16H), 1.39–1.43 (m, 2H), 1.63–1.69 (m, 2H), 1.93 (s, 6H), 2.33 (s, 3H), 2.93 (t, *J* = 7.5 Hz, 2H), 6.92 (s, 2H), 7.01 (s, 1H), 7.31 (d, *J* = 8.5 Hz, 1H), 7.63 (d, *J* = 8.5 Hz, 1H). **^13^C NMR** (125 MHz, CDCl_3_): δ 14.1, 20.4, 21.1, 22.7, 28.6, 28.8, 29.2, 29.3, 29.4, 29.5, 29.6, 31.9, 32.1, 124.3 (CF_3_, q, *J* = 271.6 Hz), 125.3 (q, *J* = 29.9 Hz), 125.4, 126.6 (q, *J* = 5.1 Hz), 127.2, 128.9, 135.4, 136.1, 137.3, 140.4, 142.9. **^19^F NMR** (470 MHz, CDCl_3_)  δ  -62.8.

**HRMS** (ESI-TOF) Calcd for C_28_H_39_F_3_NaS (M+Na) ^+^ 487.2617. Found 487.2589.

**Pseudo Three-Component, Double Nucleophilic Addition/Aromatization Reactions Using Electron-Rich Arenes as C-Nucleophiles.**

**6**: 2,2'',4,4''-tetramethoxy-4'-(trifluoromethyl)-1,1':3',1''-terphenyl

**6’**: 2,2'',4,4''-tetramethoxy-4'-(trifluoromethyl)-1,1':2',1''-terphenyl

To the solution of 1,3-dimethoxybenzene **4i** (392 μL, 3.0 mmol) in DCE (1 mL) was added TMSCl (126 μL, 1 mmol) and In(OTf)_3_ (85 mg, 0.15 mmol). The solution was heated to 80 ^o^C and the DCE solution (2 mL) of 4-(trifluoromethyl)-4-((trimethylsilyl)oxy)cyclohexa-2,5-dienone **1a** (125 mg, 0.50 mmol) was added dropwise within 40min. After the reaction was finished as indicated by TLC (reaction time, 5 h), the resulting mixture was poured into water (20 mL) and extracted with DCM (CH_2_Cl_2_, 20 mL×3). The combined organic layer was dried over anhydrous Na_2_SO_4_ and concentrated *in vacuo*. The crude product was purified by column chromatography on silica gel (EtOAc/PE = 6: 120) to afford **6/6’** (121 mg, 58%).

White solid. **6/6’** = 1.0/1.0.

**6**, White solid, m.p. 134–135 ^o^C. **^1^H NMR** (500 MHz, CDCl_3_): δ 3.71 (s, 3H), 3.79 (s, 3H), 3.83 (s, 3H), 3.84 (s, 3H), 6.52 (d, *J* = 9.0 Hz, 1H), 6.53 (s, 1H), 6.54 (s, 1H), 6.55 (d, *J* = 9.0 Hz, 1H), 7.12 (d, *J* = 9.0 Hz, 1H), 7.27 (d, *J* = 9.0 Hz, 1H), 7.42 (s, 1H), 7.60 (d, *J* = 8.0 Hz, 1H), 7.71 (d, *J* = 8.0 Hz, 1H). **^13^C NMR** (125 MHz, CDCl_3_): δ 55.2, 55.3, 55.4, 55.5, 98.3, 98.9, 103.4, 104.7, 121.5, 121.8, 124.4 (CF_3_, q, *J* = 271.6 Hz), 125.7 (q, *J* = 5.0 Hz), 127.4 (q, *J* = 29.5 Hz), 128.1, 131.1, 131.4, 133.5, 136.9, 141.1, 157.5, 157.8, 160.6, 160.8. **HRMS** (ESI-TOF) Calcd for C_23_H_22_F_3_O_4_ (M+H) ^+^ 419.1465. Found 419.1472.

**6’**, White solid, m.p. 109–110 ^o^C. **^1^H NMR** (500 MHz, CDCl_3_): δ 3.49 (s, 6H), 3.75 (s, 6H), 6.32 (s, 2H), 6.37 (d, *J* = 8.0 Hz, 2H), 6.92–6.95 (m, 2H), 7.45 (d, *J* = 8.0 Hz, 1H), 7.57 (d, *J* = 8.0 Hz, 1H), 7.61 (s, 1H). **^13^C NMR** (125 MHz, CDCl_3_): δ 54.9, 55.2, 98.1, 103.8, 122.4, 122.5, 123.4 (q, *J* = 3.8 Hz), 124.1 (CF_3_, q, *J* = 271.6 Hz), 127.7, 128.6 (q, *J* = 31.9 Hz), 131.2, 131.3, 131.4, 138.8, 141.9, 156.9, 160.1 (2). **HRMS** (ESI-TOF) Calcd for C_23_H_22_F_3_O_4_ (M+H) ^+^ 419.1465. Found 419.1468.

**Three-Component, Double Nucleophilic 1,3-Carboamination/Aromatization Reactions.**

**8aa**: 4-methyl-*N*-(3-(3-oxopentan-2-yl)-4-(trifluoromethyl)phenyl)benzenesulfonamide

To the solution of 4-(trifluoromethyl)-4-((trimethylsilyl)oxy)cyclohexa-2,5-dienone **1a** (150 mg, 0.60 mmol) and pentan-3-one **2a** (157 μL, 1.5 mmol) in DCE (3 mL) was added TMSCl (126 μL, 1 mmol), In(OTf)_3_ (141 mg, 0.25 mmol) and 4-methylbenzenesulfonamide **7a** (86 mg, 0.5 mmol) at room temperature. After the reaction was finished as indicated by TLC (reaction time, 20 h), the resulting mixture was poured into water (20 mL) and extracted with DCM (CH_2_Cl_2_, 20 mL×3). The combined organic layer was dried over anhydrous Na_2_SO_4_ and concentrated *in vacuo*. The crude product was purified by column chromatography on silica gel (EtOAc/PE = 8: 120) to afford **8aa** (115 mg, 58%).

Colorless crystals, m.p. 143–144 ^o^C. **^1^H NMR** (500 MHz, CDCl_3_): δ 0.92 (t, *J* = 7.5 Hz, 3H), 1.29 (d, *J* = 7.0 Hz, 3H), 2.19–2.24 (m, 1H), 2.32–2.36 (m, 1H), 2.38 (s, 3H), 4.13 (q, *J* = 7.0 Hz, 1H), 6.92 (s, 1H), 7.26 (m, 3H), 7.56 (d, *J* = 8.0 Hz, 1H), 7.75 (d, *J* = 8.0 Hz, 2H), 8.11 (s, 1H). **^13^C NMR** (125 MHz, CDCl_3_): δ 7.8, 18.2, 21.5, 34.6, 47.5, 117.2, 119.0, 123.8 (q, *J* = 30.0 Hz), 124.0 (CF_3_, q, *J* = 271.5 Hz), 127.3, 127.6 (q, *J* = 5.8 Hz), 129.7, 135.7, 140.8, 140.9, 144.3, 211.3. **HRMS** (ESI-TOF) Calcd for C_19_H_21_F_3_NO_3_S (M+H) ^+^ 400.1189. Found 400.1197.

**8ba**: *N*-(3-(3-oxopentan-2-yl)-4-(trifluoromethyl)phenyl)methanesulfonamide

Following the procedure for the synthesis of **8aa**, the reaction of 4-(trifluoromethyl)-4-((trimethylsilyl)oxy)cyclohexa-2,5-dienone **1a** (150 mg, 0.60 mmol), methanesulfonamide **7b** (48 mg, 0.5 mmol) and pentan-3-one **2a** (157 μL, 1.5 mmol) gave **8ba** (74 mg, 46%) after purification by column chromatography on silica gel (EtOAc/PE = 8: 120). Reaction time 20 h.

Colorless crystals, m.p. 89–90 ^o^C. **^1^H NMR** (500 MHz, CDCl_3_): δ 1.00 (t, *J* = 7.0 Hz, 3H), 1.39 (d, *J* = 6.5 Hz, 3H), 2.46 (q, *J* = 7.0 Hz, 2H), 3.11 (s, 3H), 4.25 (q, *J* = 6.5 Hz, 1H), 7.07 (s, 1H), 7.39 (d, *J* = 8.5 Hz, 1H), 7.67 (d, *J* = 8.5 Hz, 1H), 7.99 (s, 1H). **^13^C NMR** (125 MHz, CDCl_3_): δ 7.9, 18.5, 34.9, 39.9, 47.3, 116.3, 118.9, 123.9 (q, *J* = 30.0 Hz), 124.1 (CF_3_, q, *J* = 271.5 Hz), 127.9 (q, *J* = 5.8 Hz), 141.1, 141.2, 211.9. **HRMS** (ESI-TOF) Calcd for C_13_H_17_F_3_NO_3_S (M+H) ^+^ 324.0876. Found 324.0876.

**8ca**: *N*-(3-(3-oxopentan-2-yl)-4-(trifluoromethyl)phenyl)benzenesulfonamide

Following the procedure for the synthesis of **8aa**, the reaction of 4-(trifluoromethyl)-4-((trimethylsilyl)oxy)cyclohexa-2,5-dienone **1a** (150 mg, 0.60 mmol), benzenesulfonamide **7c** (79 mg, 0.5 mmol) and pentan-3-one **2a** (157 μL, 1.5 mmol) gave **8ca** (91 mg, 47%) after purification by column chromatography on silica gel (EtOAc/PE = 8: 120). Reaction time 20 h.

Colorless crystals, m.p. 134–136 ^o^C. **^1^H NMR** (500 MHz, CDCl_3_): δ 0.92 (t, *J* = 7.5 Hz, 3H), 1.29 (d, *J* = 7.0 Hz, 3H), 2.19–2.26 (m, 1H), 2.32–2.38 (m, 1H), 4.14 (q, *J* = 7.0 Hz, 1H), 6.91 (s, 1H), 7.28 (d, *J* = 9.0 Hz, 1H), 7.47 (t, *J* = 7.5 Hz, 2H), 7.56 (t, *J* = 7.5 Hz, 1H), 7.57 (d, *J* = 9.0 Hz, 1H), 7.86 (t, *J* = 7.5 Hz, 2H), 8.10 (s, 1H). **^13^C NMR** (125 MHz, CDCl_3_): δ 7.9, 18.3, 34.7, 47.4, 117.5, 119.2, 124.0 (q, *J* = 30.0 Hz), 124.1 (CF_3_, q, *J* = 271.5 Hz), 127.3, 127.7 (q, *J* = 5.8 Hz), 129.2, 133.3, 138.7, 140.8, 140.9, 211.6. **^19^F NMR** (470 MHz, CDCl_3_)  δ  -60.4. **HRMS** (ESI-TOF) Calcd for C_18_H_19_F_3_NO_3_S (M+H) ^+^ 386.1032. Found 386.1040.

**8da**: 4-chloro-*N*-(3-(3-oxopentan-2-yl)-4-(trifluoromethyl)phenyl)benzenesulfonamide

Following the procedure for the synthesis of **8aa**, the reaction of 4-(trifluoromethyl)-4-((trimethylsilyl)oxy)cyclohexa-2,5-dienone **1a** (150 mg, 0.60 mmol), 4-chlorobenzenesulfonamide **7d** (95 mg, 0.5 mmol) and pentan-3-one **2a** (157 μL, 1.5 mmol) gave **8da** (84 mg, 40%) after purification by column chromatography on silica gel (EtOAc/PE = 8: 120). Reaction time 20 h.

Colorless crystals, m.p. 151–152 ^o^C. **^1^H NMR** (500 MHz, CDCl_3_): δ 0.94 (t, *J* = 7.0 Hz, 3H), 1.30 (d, *J* = 6.5 Hz, 3H), 2.20–2.29 (m, 1H), 2.35–2.42 (m, 1H), 4.17 (q, *J* = 6.5 Hz, 1H), 6.91 (s, 1H), 7.27 (d, *J* = 8.5 Hz, 1H), 7.44 (d, *J* = 8.5 Hz, 2H), 7.59 (d, *J* = 8.5 Hz, 1H), 7.79 (d, *J* = 8.5 Hz, 2H), 8.05 (s, 1H). **^13^C NMR** (125 MHz, CDCl_3_): δ 7.9, 18.4, 34.9, 47.4, 117.5, 119.4, 124.2 (CF_3_, q, *J* = 271.5 Hz), 124.4 (q, *J* = 30.0 Hz), 127.8 (q, *J* = 5.8 Hz), 128.7, 129.5, 137.1, 139.9, 140.4, 141.0, 211.7. **HRMS** (ESI-TOF) Calcd for C_18_H_18_ClF_3_NO_3_S (M+H) ^+^ 420.0643. Found 420.0652.

**8ab**: 4-methyl-*N*-(3-(3-oxobutan-2-yl)-4-(trifluoromethyl)phenyl)benzenesulfonamide

**8ab’**: 4-methyl-*N*-(3-(2-oxobutyl)-4-(trifluoromethyl)phenyl)benzenesulfonamide

Following the procedure for the synthesis of **8ac**, the reaction of 4-(trifluoromethyl)-4-((trimethylsilyl)oxy)cyclohexa-2,5-dienone **1a** (150 mg, 0.60 mmol), 4-methylbenzenesulfonamide **7a** (86 mg, 0.5 mmol) and butan-2-one **2b** (134 μL, 1.5 mmol) gave **8ab** (88 mg, 46%) and **8ab’** (19 mg, 10%) after purification by column chromatography on silica gel (EtOAc/PE = 8: 120). Reaction time 20 h.

**8ab**, Colorless crystals, m.p. 117–118 ^o^C. **^1^H NMR** (500 MHz, CDCl_3_): δ 1.29 (d, *J* = 6.5 Hz, 3H), 1.99 (s, 3H), 2.39 (s, 3H), 4.12 (q, *J* = 6.5 Hz, 1H), 6.88 (s, 1H), 7.22 (d, *J* = 9.0 Hz, 1H), 7.27 (d, *J* = 8.5 Hz, 2H), 7.57 (d, *J* = 9.0 Hz, 1H), 7.73 (d, *J* = 8.5 Hz, 2H), 7.93 (s, 1H). **^13^C NMR** (125 MHz, CDCl_3_): δ 17.9, 21.5, 28.8, 48.4, 117.4, 119.1, 123.9 (q, *J* = 30.0 Hz), 124.1 (CF_3_, q, *J* = 271.5 Hz), 127.3, 127.7 (q, *J* = 5.8 Hz), 129.8, 135.6, 140.7, 140.9, 144.4, 208.4. **HRMS** (ESI-TOF) Calcd for C_18_H_19_F_3_NO_3_S (M+H) ^+^ 386.1032. Found 386.1026.

**8ab’**, Colorless crystals, m.p. 94–95 ^o^C. **^1^H NMR** (500 MHz, CDCl_3_): δ 1.05 (t, *J* = 7.5 Hz, 3H), 2.38 (s, 3H), 2.47 (q, *J* = 7.5 Hz, 2H), 3.81 (s, 2H), 7.02 (s, 1H), 7.04 (d, *J* = 8.5 Hz, 1H), 7.26 (d, *J* = 8.5 Hz, 2H), 7.47 (d, *J* = 8.5 Hz, 1H), 7.50 (s, 1H), 7.73 (d, *J* = 8.5 Hz, 2H). **^13^C NMR** (125 MHz, CDCl_3_): δ 7.7, 21.6, 35.7, 46.0, 117.6, 123.6, 124.2 (CF_3_, q, *J* = 271.5 Hz), 124.7 (q, *J* = 30.0 Hz), 127.2, 127.4 (q, *J* = 5.8 Hz), 129.9, 134.5, 135.6, 139.9, 144.4, 207.1. **HRMS** (ESI-TOF) Calcd for C_18_H_19_F_3_NO_3_S (M+H) ^+^ 386.1032. Found 386.1042.

**8ac**: 4-methyl-*N*-(3-(2-oxo-2-phenylethyl)-4-(trifluoromethyl)phenyl)benzenesulfonamide

Following the procedure for the synthesis of **8aa**, the reaction of 4-(trifluoromethyl)-4-((trimethylsilyl)oxy)cyclohexa-2,5-dienone **1a** (150 mg, 0.60 mmol), 4-methylbenzenesulfonamide **7a** (86 mg, 0.5 mmol) and acetophenone **2c** (175 μL, 1.5 mmol) gave **8ac** (87 mg, 40%) after purification by column chromatography on silica gel (EtOAc/PE = 8: 120). Reaction time 20 h.

Colorless crystals, m.p. 173–174 ^o^C. **^1^H NMR** (500 MHz, CDCl_3_): δ 2.37 (s, 3H), 4.41 (s, 2H), 7.06 (d, *J* = 7.5 Hz, 2H), 7.24 (t, *J* = 7.5 Hz, 2H), 7.45 (s, 1H), 7.49 (m,` 3H), 7.61 (t, *J* = 7.5 Hz, 1H), 7.71 (d, *J* = 7.5 Hz, 2H), 7.97 (d, *J* = 7.5 Hz, 2H). **^13^C NMR** (125 MHz, CDCl_3_): δ 21.6, 42.3, 117.6, 123.6, 124.3 (CF_3_, q, *J* = 271.5 Hz), 124.9 (q, *J* = 30.0 Hz), 127.2, 127.5 (q, *J* = 5.8 Hz), 128.2, 128.8, 129.9, 133.5, 134.8, 135.7, 136.2, 139.9, 144.3, 196.1. **HRMS** (ESI-TOF) Calcd for C_22_H_19_F_3_NO_3_S (M+H) ^+^ 434.1032. Found 434.1041.

**8ad**: ethyl 3-hydroxy-2-(5-(4-methylphenylsulfonamido)-2-(trifluoromethyl)phenyl)but-2-enoate

Following the procedure for the synthesis of **8aa**, the reaction of 4-(trifluoromethyl)-4-((trimethylsilyl)oxy)cyclohexa-2,5-dienone **1a** (150 mg, 0.60 mmol), 4-methylbenzenesulfonamide **7a** (86 mg, 0.5 mmol) and 3-oxobutanoate **2d** (189 μL, 1.5 mmol) gave **8ad** (157 mg, 71%) after purification by column chromatography on silica gel (EtOAc/PE = 8: 120). Reaction time 20 h.

Colorless crystals, m.p. 196–197 ^o^C. **^1^H NMR** (500 MHz, CDCl_3_): δ 1.06 (t, *J* = 7.0 Hz, 3H), 1.56 (s, 3H), 2.38 (s, 3H), 3.97–4.03 (m, 1H), 4.14–4.19 (m, 1H), 6.99 (s, 1H), 7.17 (d, *J* = 8.5 Hz, 1H), 7.27 (d, *J* = 8.5 Hz, 2H), 7.54 (d, *J* = 8.5 Hz, 1H), 7.76 (d, *J* = 8.5 Hz, 2H), 7.97 (s, 1H), 12.9 (s, 1H). **^13^C NMR** (125 MHz, CDCl_3_): δ 13.8, 19.5, 21.5, 60.6, 100.7, 118.7, 123.9 (CF_3_, q, *J* = 271.5 Hz), 124.3, 126.4 (q, *J* = 30.0 Hz), 127.3, 127.5 (q, *J* = 5.8 Hz), 129.8, 135.3, 135.6, 139.8, 144.5, 171.5, 173.8. **HRMS** (ESI-TOF) Calcd for C_20_H_21_F_3_NO_5_S (M+H)^+^ 444.1087. Found 444.1073.

**9**: *N*-(4-hydroxy-4-(trifluoromethyl)cyclohexa-2,5-dien-1-ylidene)-4-methylbenzenesulfonamide

Following the procedure for the synthesis of **8ac**, the reaction of 4-(trifluoromethyl)-4-((trimethylsilyl)oxy)cyclohexa-2,5-dienone **1a** (150 mg, 0.60 mmol) and 4-methylbenzenesulfonamide **7a** (86 mg, 0.5 mmol) gave the mixture of **9** (58 mg, 35%) and 4-hydroxy-4-(trifluoromethyl)cyclohexa-2,5-dienone (48 mg, 32%) after purification by column chromatography on silica gel (EtOAc/PE = 12: 120). Reaction time 24 h.

**9**, White solid. **^1^H NMR** (500 MHz, CDCl_3_): δ 2.45 (s, 3H), 4.39 (s, 1H), 6.46 (dd, *J* = 10.0 Hz, *J* = 1.5 Hz, 1H), 6.76 (dd, *J* = 10.0 Hz, *J* = 2.5 Hz, 1H), 6.82 (dd, *J* = 10.0 Hz, *J* = 2.5 Hz, 1H), 7.35 (d, *J* = 8.0 Hz, 2H), 7.65 (dd, *J* = 10.0 Hz, *J* = 1.5 Hz, 1H), 7.82 (d, *J* = 8.0 Hz, 2H). **^13^C NMR** (125 MHz, CDCl_3_): δ 21.6, 69.4 (q, *J* = 31.0 Hz), 123.3 (CF_3_, q, *J* = 271.5 Hz), 124.9, 127.2, 129.7, 132.3, 136.8, 140.0, 141.9, 144.7, 162.3. **HRMS** (ESI-TOF) Calcd for C_14_H_13_F_3_NO_3_S (M+H) ^+^ 332.0563. Found 332.0570.

The NOESY spectrum of **5ad**/**5ad’**


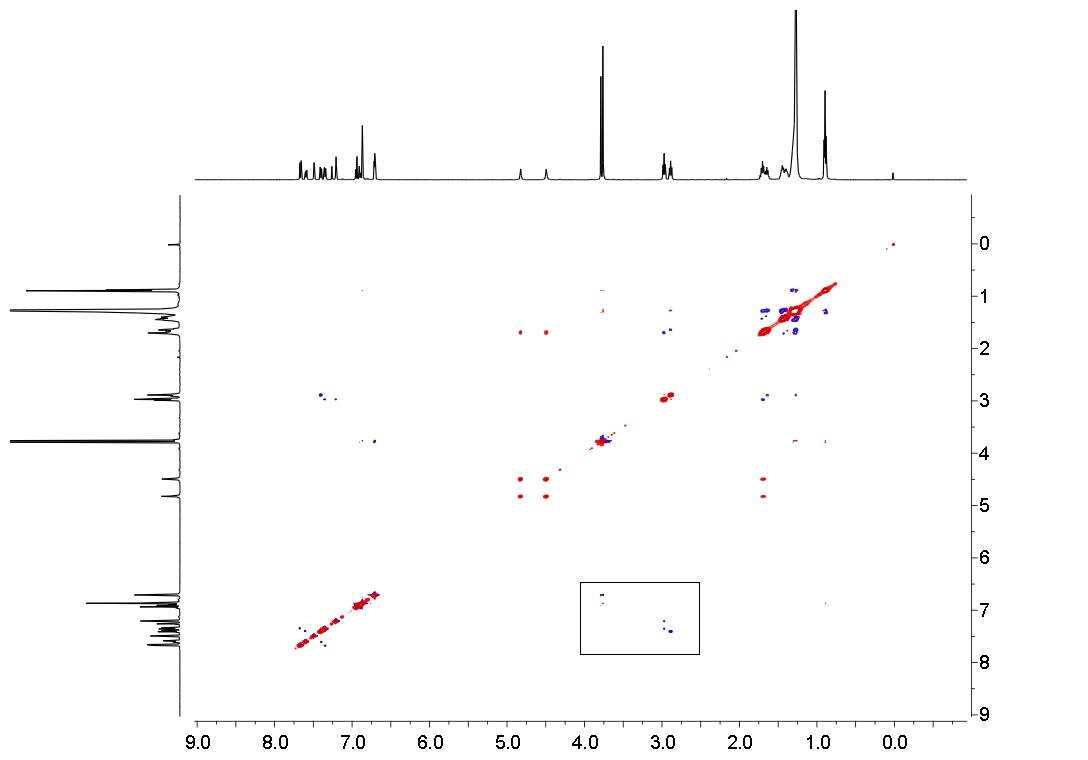


**III . Crystal Data and OPTEP Drawings**

Single-crystal X-ray diffraction data was collected at room temperature on a Oxford Diffraction Gemini R Ultra diffractometer, the X-ray generator using Mo-Kα (λ = 0.71073 Å) radiation with a ω scan technique. The crystal structures were solved by direct method of SHELXS-97[[2]](#footnote-3)2and refined by full-matrix least-squares techniques using the SHELXL-97 program. Non-hydrogen atoms were refined anisotropic. CCDC deposition number: 1404121 (**5ba’**); 1404122 (**6**); 1404120 (**8aa**). Data can be obtained free of charge via www.ccdc.cam.ac.uk/conts/retrieving.html (or from the Cambridge Crystallographic Data Center, 12 Union Road, Cambridge CB21EZ, UK; fax: (+44)1223-336-033; or [deposit@ccdc.cam.ac.uk](mailto:deposit@ccdc.cam.ac.uk)).

(1) Crystal data and OPTEP drawing of compound **5ba’**

ORTEP drawing:

Crystal data:

| Empirical formula | C_23_H_21_F_3_O_3_S |
| --- | --- |
| Formula weight | 434.46 |
| Crystal system | Triclinic |
| Space group | P -1 |
| a (Å) | 7.5818(5) |
| b (Å) | 10.0893(7) |
| c (Å) | 13.6798(9) |
| *α* (deg) | 83.4260(10) |
| *β* (deg) | 83.2270(10) |
| γ (deg) | 84.2990(10) |
| Volume (Å^3^) | 1028.39(12) |
| Z | 2 |
| Calculated density (mg/m^3^) | 1.40 |
| Absorption coefficient (mm^-1^) | 0.206 |
| F(000) | 452 |
| Theta range for data collection (deg) | 1.5 to 26.0 |
| Reflections collected/unique | 5571/3956 |
| Goodness-of-fit on F^2^ | 1.044 |
| Final R indices [*I* > 2*σ* *(I)*] | R1=0.057, WR2 =0.146 |
| R indices (all data) | R1=0.072, WR2 =0.162 |

(2) Crystal data and OPTEP drawing of compound **6.**

ORTEP drawing:

**Crystal data:**

| Empirical formula | C_23_H_21_F_3_O_4_ |
| --- | --- |
| Formula weight | 418.40 |
| Crystal system | Monoclinic |
| Space group | P 21/c |
| a (Å) | 12.573(2) |
| b (Å) | 21.245(4) |
| c (Å) | 7.8281(14) |
| *α* (deg) | 90.00 |
| *β* (deg) | 99.599(4) |
| γ (deg) | 90.00 |
| Volume (Å^3^) | 2061.7(6) |
| Z | 4 |
| Calculated density (mg/m^3^) | 1.348 |
| Absorption coefficient (mm^-1^) | 0.109 |
| F(000) | 872 |
| Theta range for data collection (deg) | 1.9 to 26.1 |
| Reflections collected/unique | 11084/4045 |
| Goodness-of-fit on F^2^ | 1.004 |
| Final R indices [*I* > 2*σ* *(I)*] | R1=0.067, WR2 =0.140 |
| R indices (all data) | R1=0.154, WR2 =0.181 |

(3) Crystal data and OPTEP drawing of compound **8aa**

ORTEP drawing:

Crystal data:

| Empirical formula | C_19_H_20_F_3_NO_3_S |
| --- | --- |
| Formula weight | 399.42 |
| Crystal system | Triclinic |
| Space group | P -1 |
| a (Å) | 10.2242(13) |
| b (Å) | 10.9396(14) |
| c (Å) | 11.8376(15) |
| *α* (deg) | 97.812(2) |
| *β* (deg) | 113.029(2) |
| γ (deg) | 99.496(2) |
| Volume (Å^3^) | 1172.1(3) |
| Z | 3 |
| Calculated density (mg/m^3^) | 1.698 |
| Absorption coefficient (mm^-1^) | 0.265 |
| F(000) | 624 |
| Theta range for data collection (deg) | 1.9 to 28.3 |
| Reflections collected/unique | 10097/5346 |
| Goodness-of-fit on F^2^ | 1.032 |
| Final R indices [*I* > 2*σ* *(I)*] | R1=0.065, WR2 =0.185 |
| R indices (all data) | R1=0.078, WR2 =0.198 |

**IV. Copies of ^1^H NMR , ^13^C NMR and ^19^F NMR Spectra**

**^1^H NMR** (500 MHz, CDCl_3_) for **3aa**


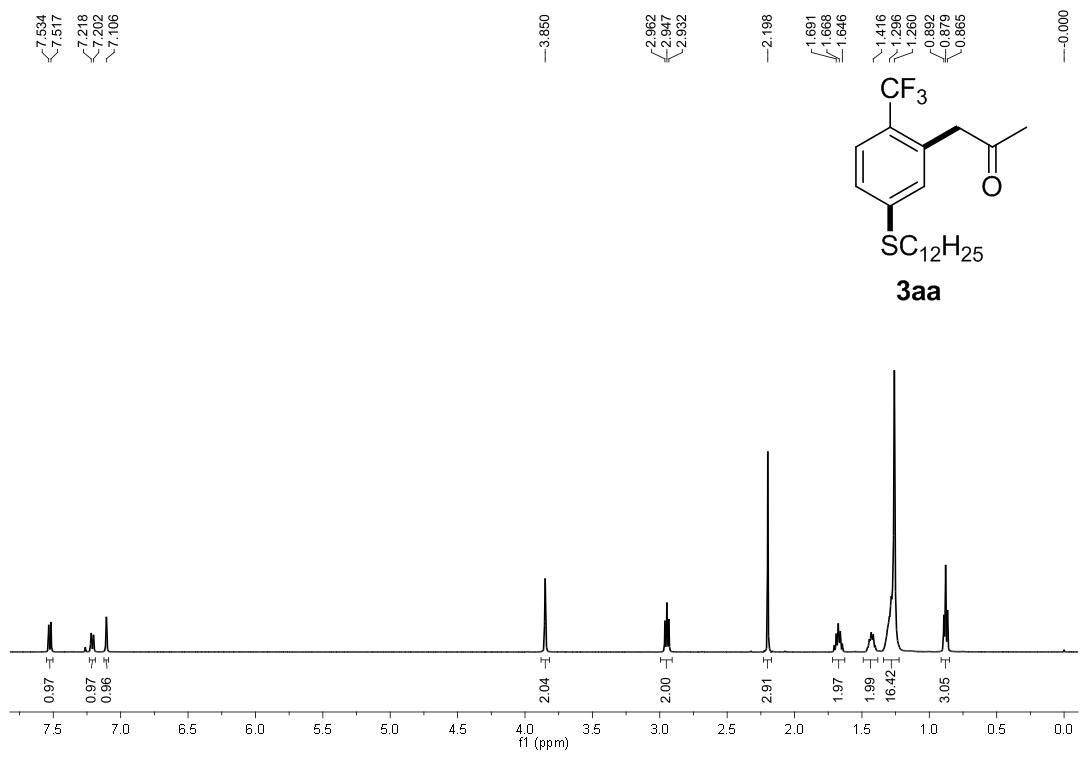


**^13^C NMR** (125 MHz, CDCl_3_) for **3aa**


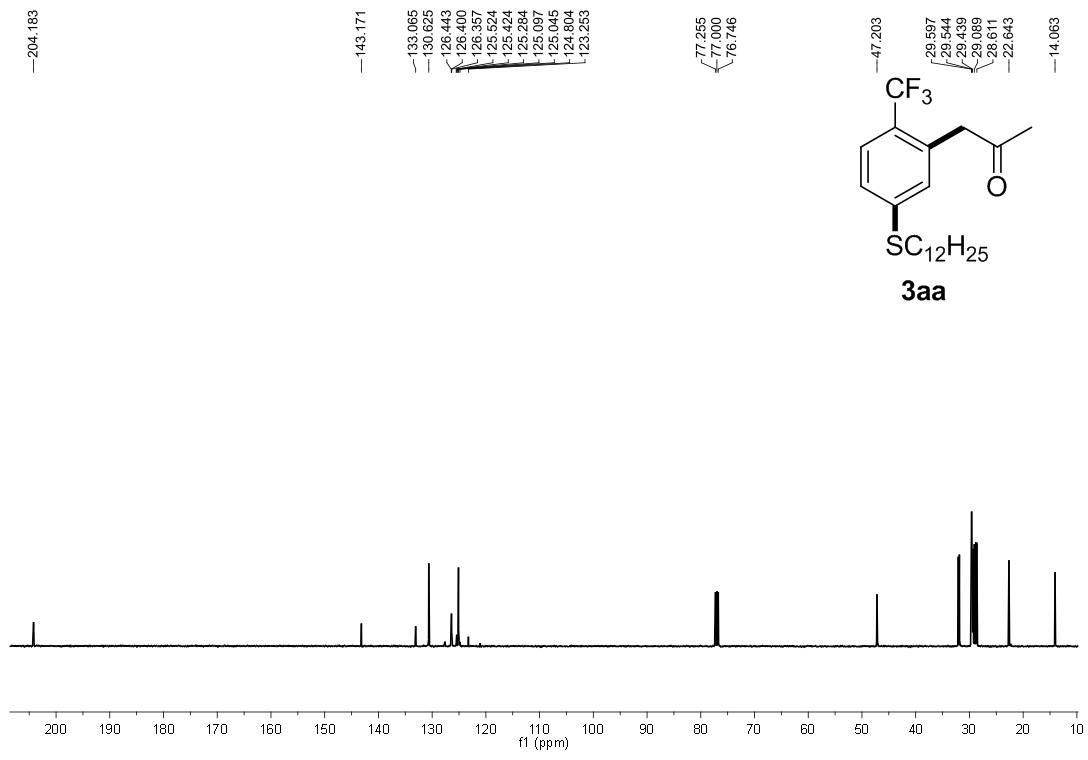


**^1^H NMR** (500 MHz, CDCl_3_) for **3ab**


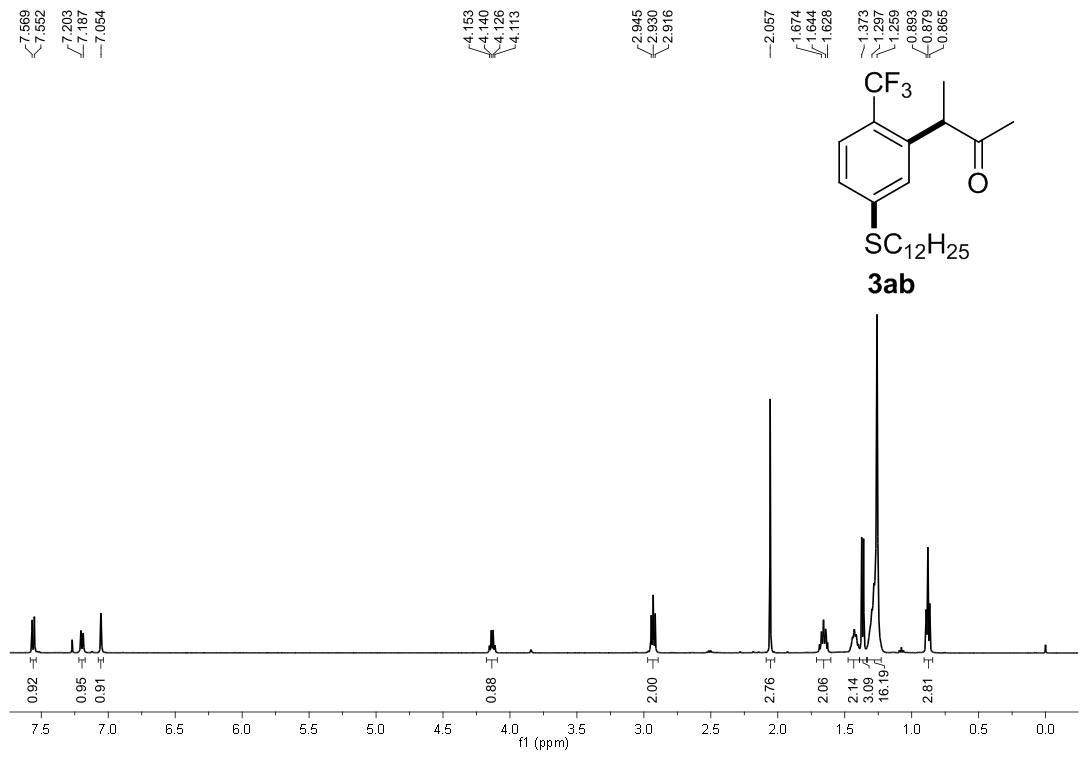


**^13^C NMR** (125 MHz, CDCl_3_) for **3ab**


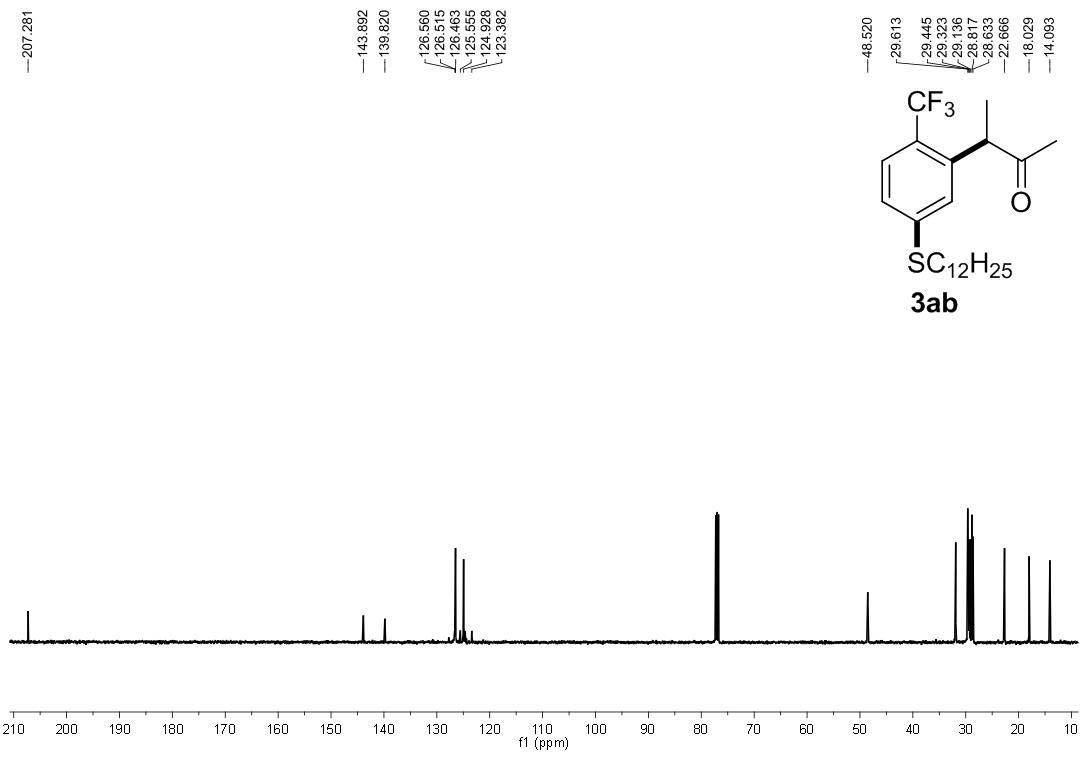


**^19^F NMR** (470 MHz, CDCl_3_) for **3ab**


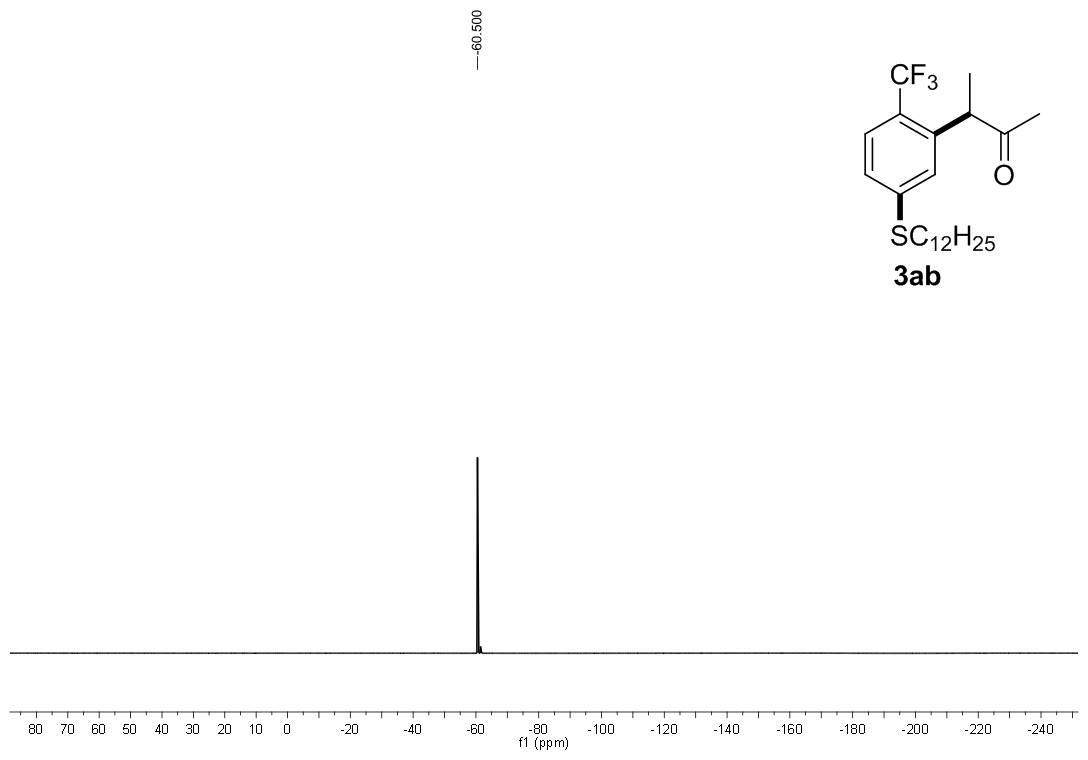


**^1^H NMR** (500 MHz, CDCl_3_) for **3ac**


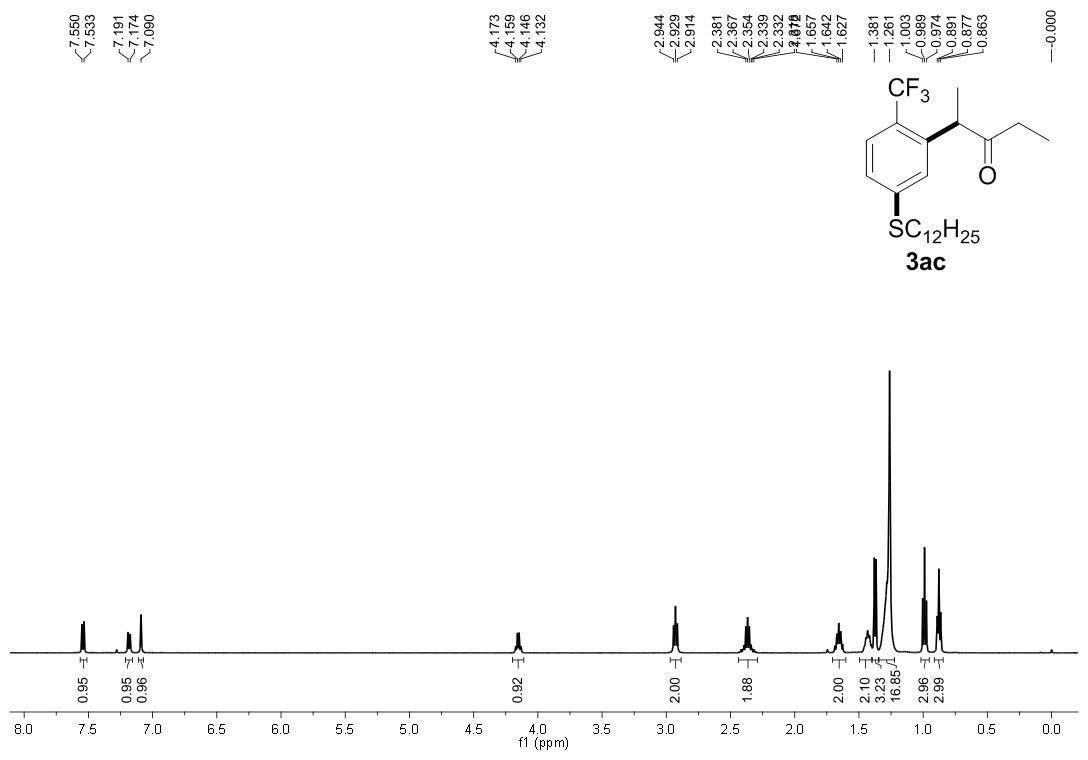


**^13^C NMR** (125 MHz, CDCl_3_) for **3ac**


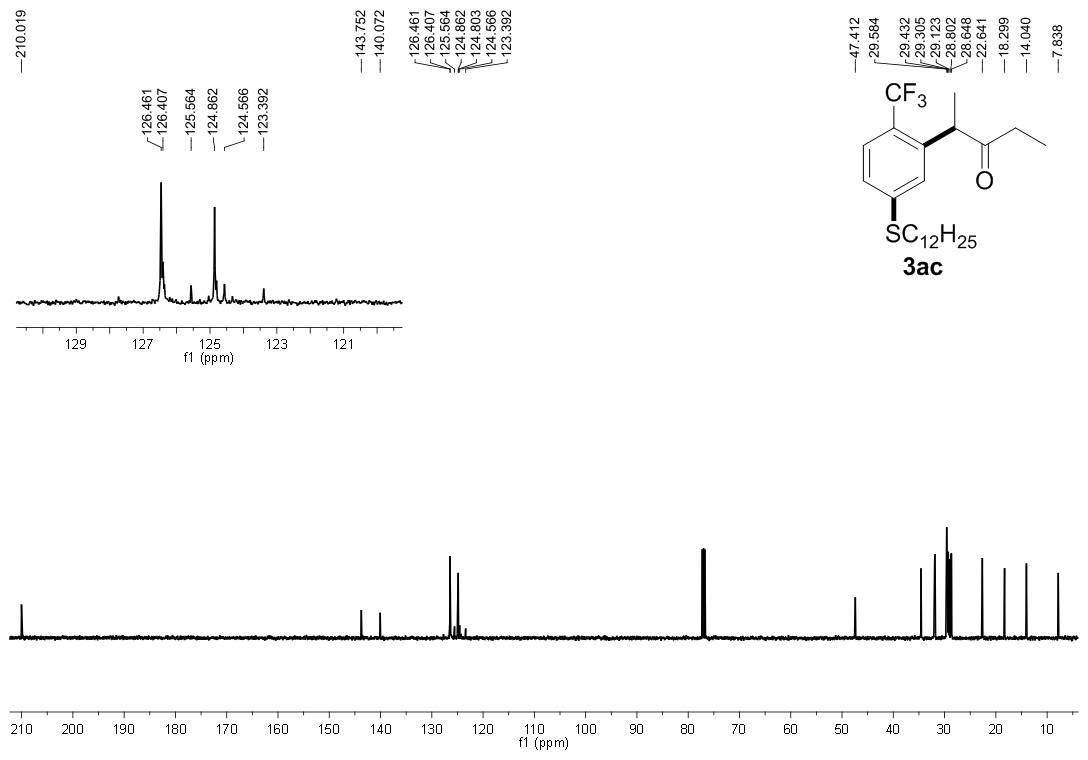


**^1^H NMR** (500 MHz, CDCl_3_) for **3ad**


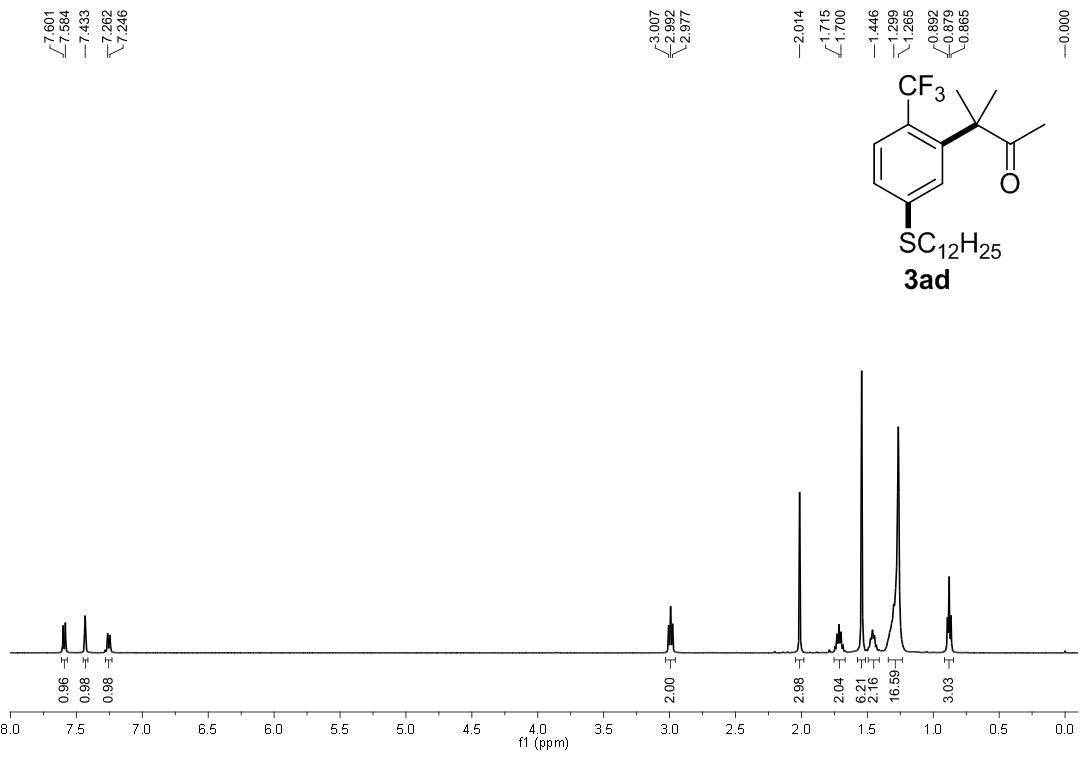


**^13^C NMR** (125 MHz, CDCl_3_) for **3ad**


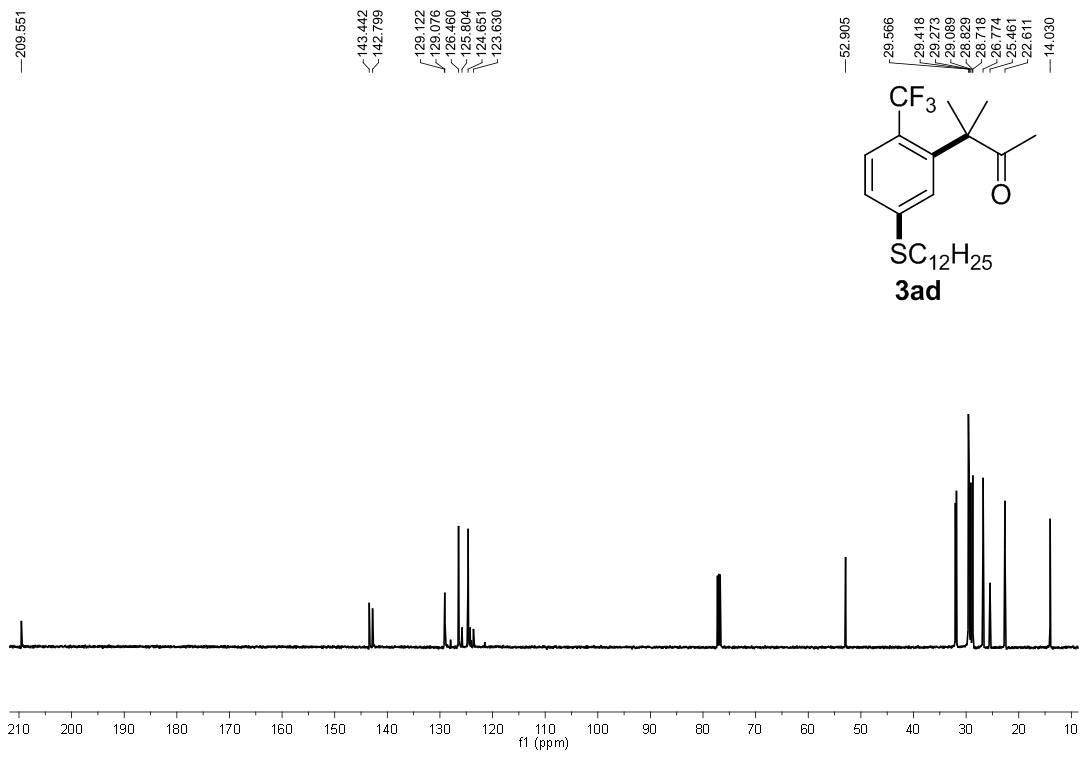


**^19^F NMR** (470 MHz, CDCl_3_) for **3ad**


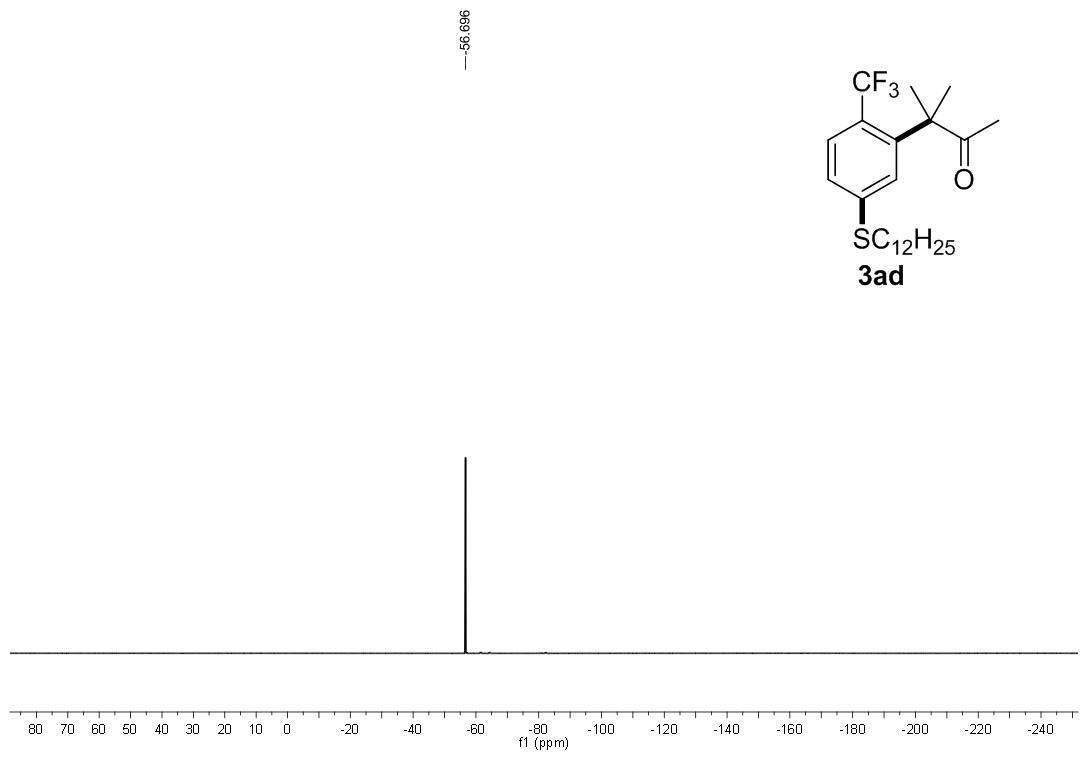


**^1^H NMR** (500 MHz, CDCl_3_) for **3ae**


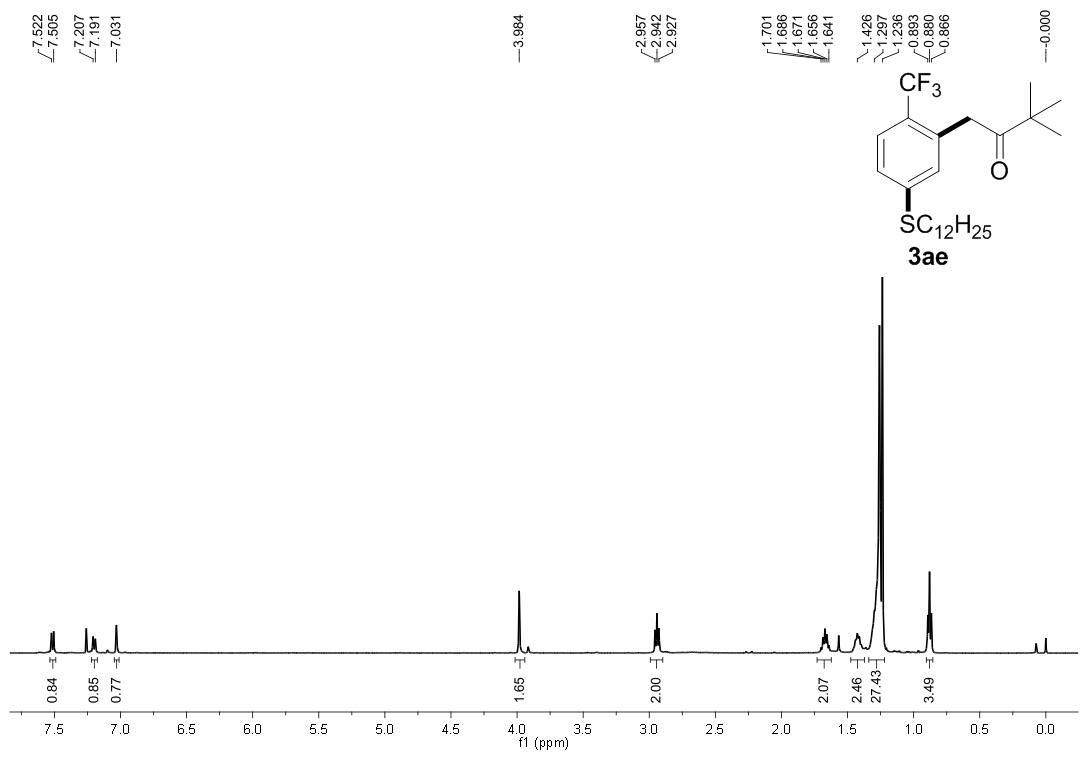


**^13^C NMR** (125 MHz, CDCl_3_) for **3ae**


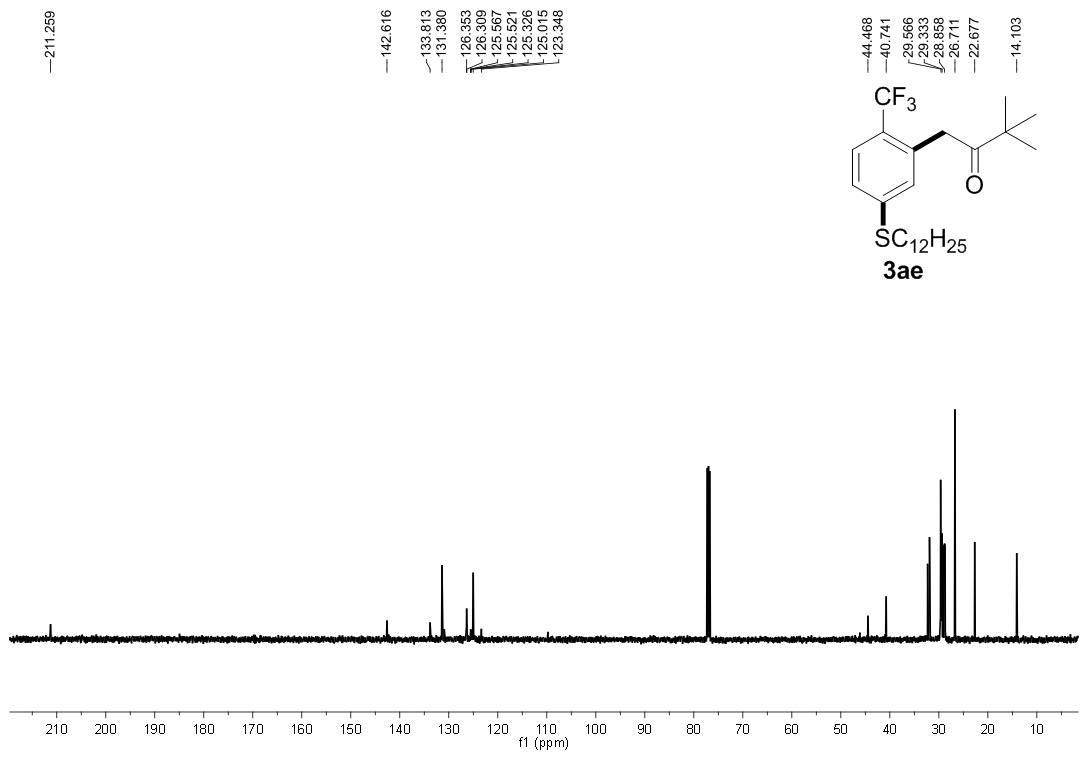


**^1^H NMR** (500 MHz, CDCl_3_) for **3af**


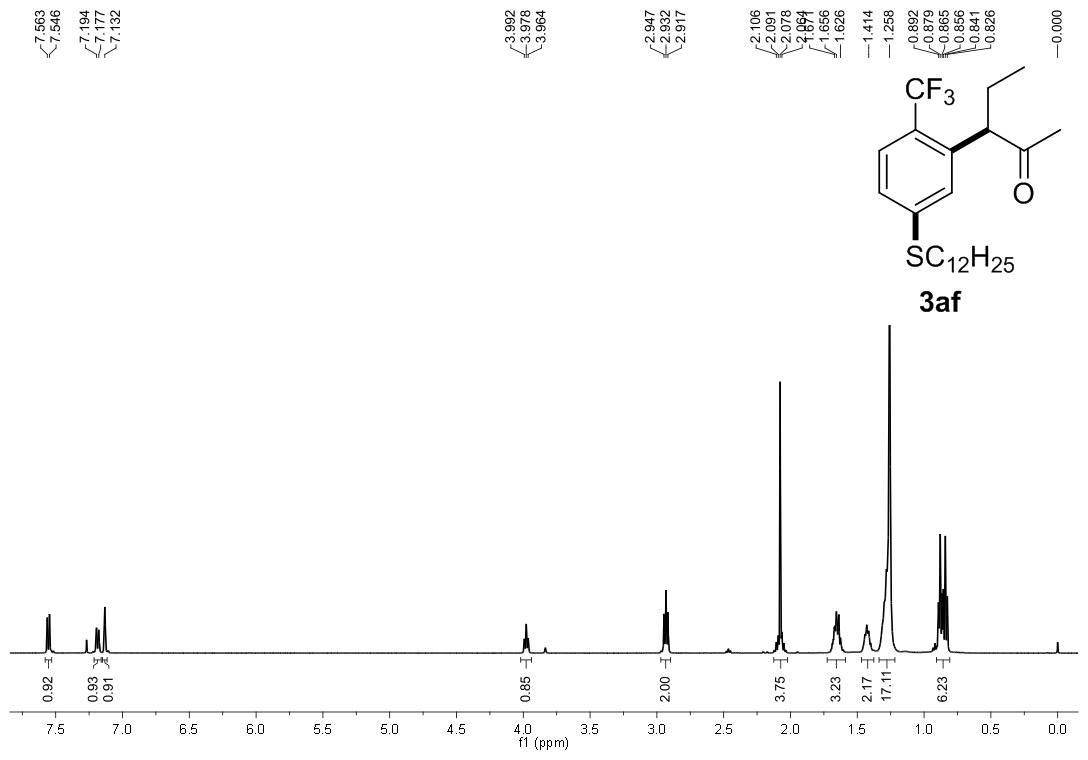


**^13^C NMR** (125 MHz, CDCl_3_) for **3af**


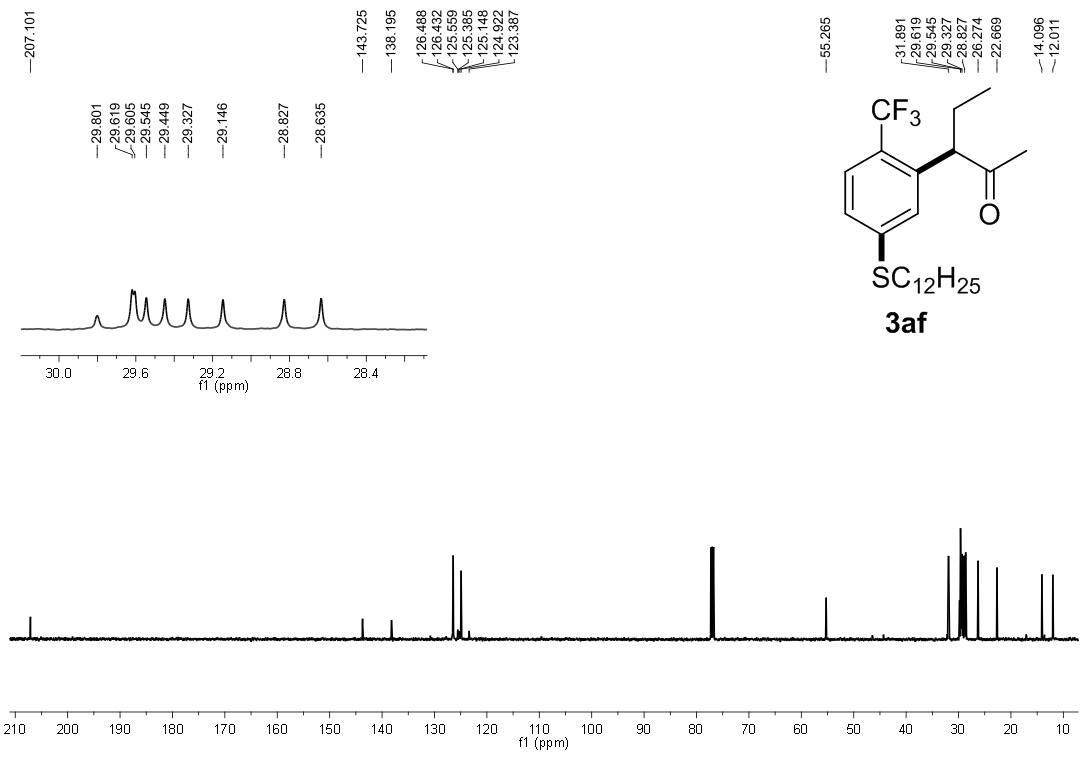


**^1^H NMR** (500 MHz, CDCl_3_) for **3ag**


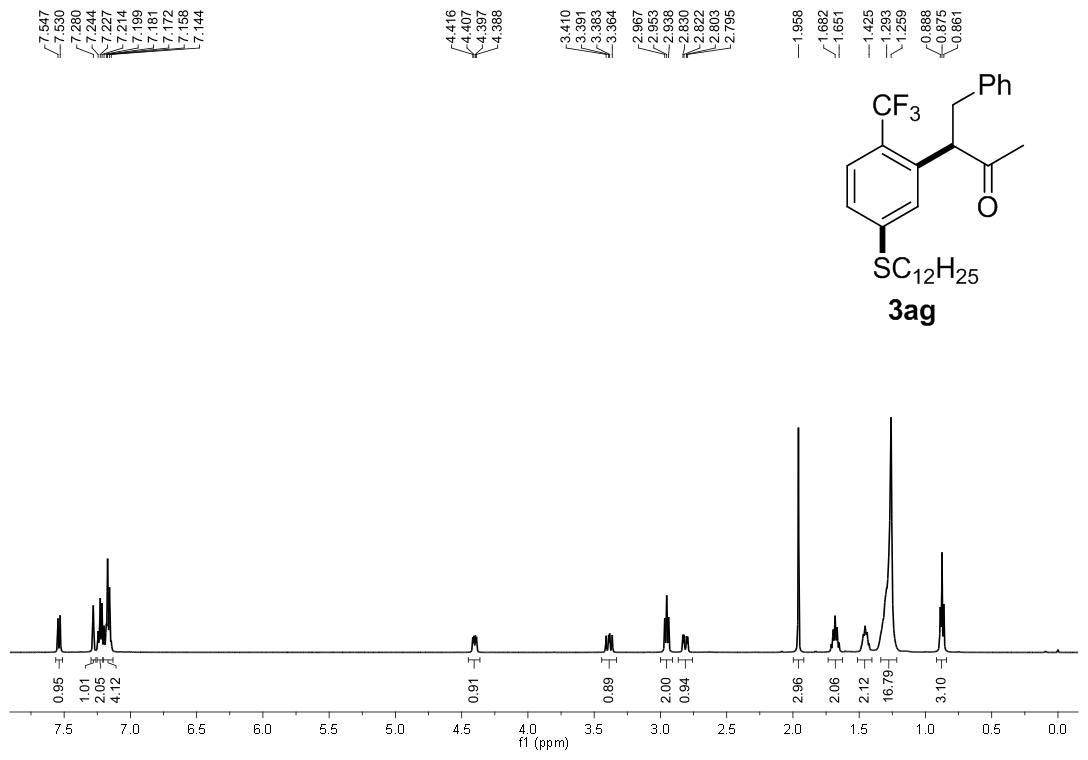


**^13^C NMR** (125 MHz, CDCl_3_) for **3ag**


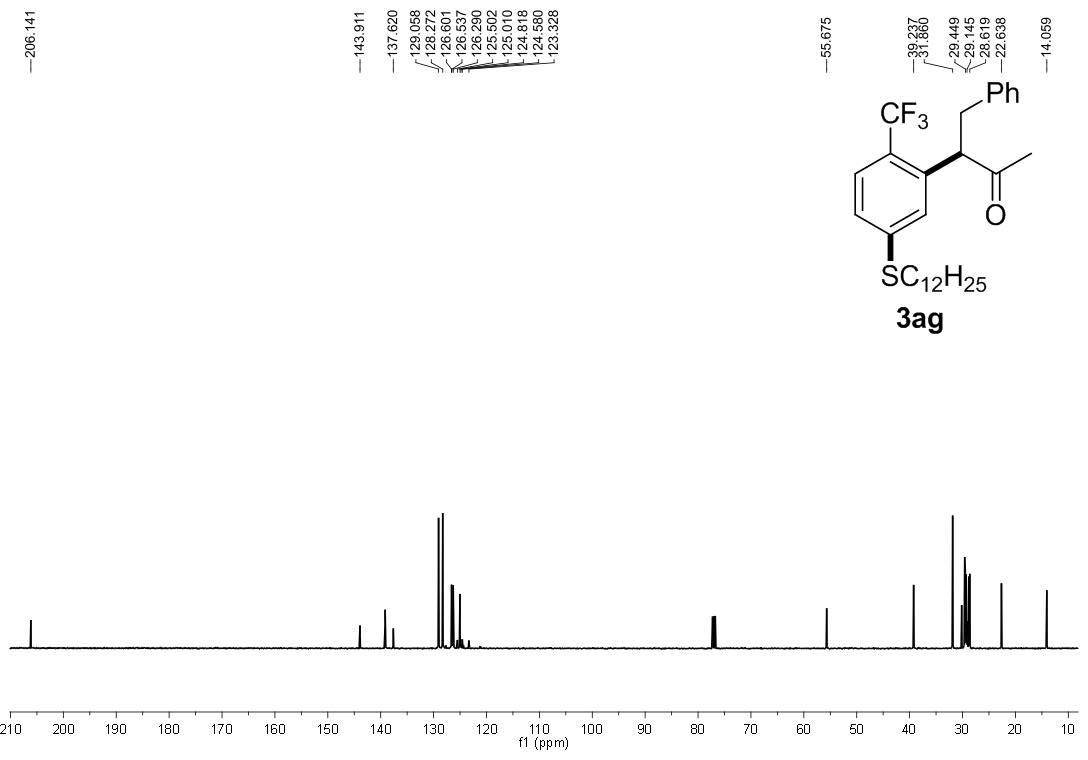


**^1^H NMR** (500 MHz, CDCl_3_) for **3ag’**


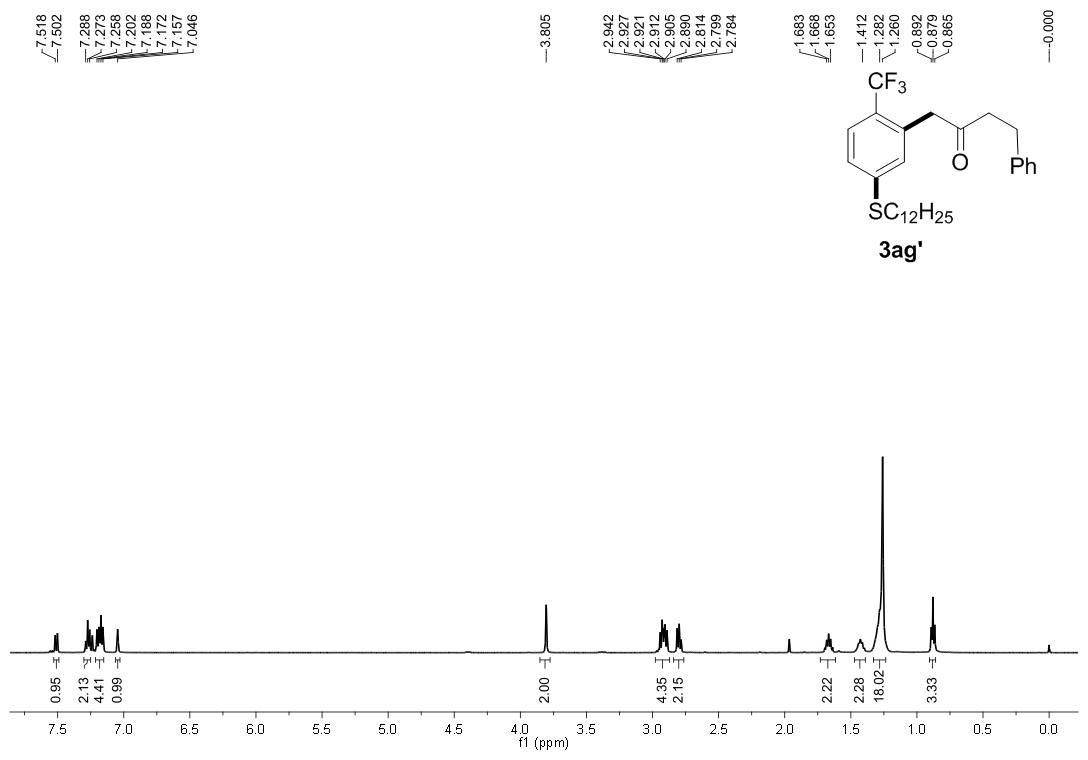


**^13^C NMR** (125 MHz, CDCl_3_) for **3ag’**


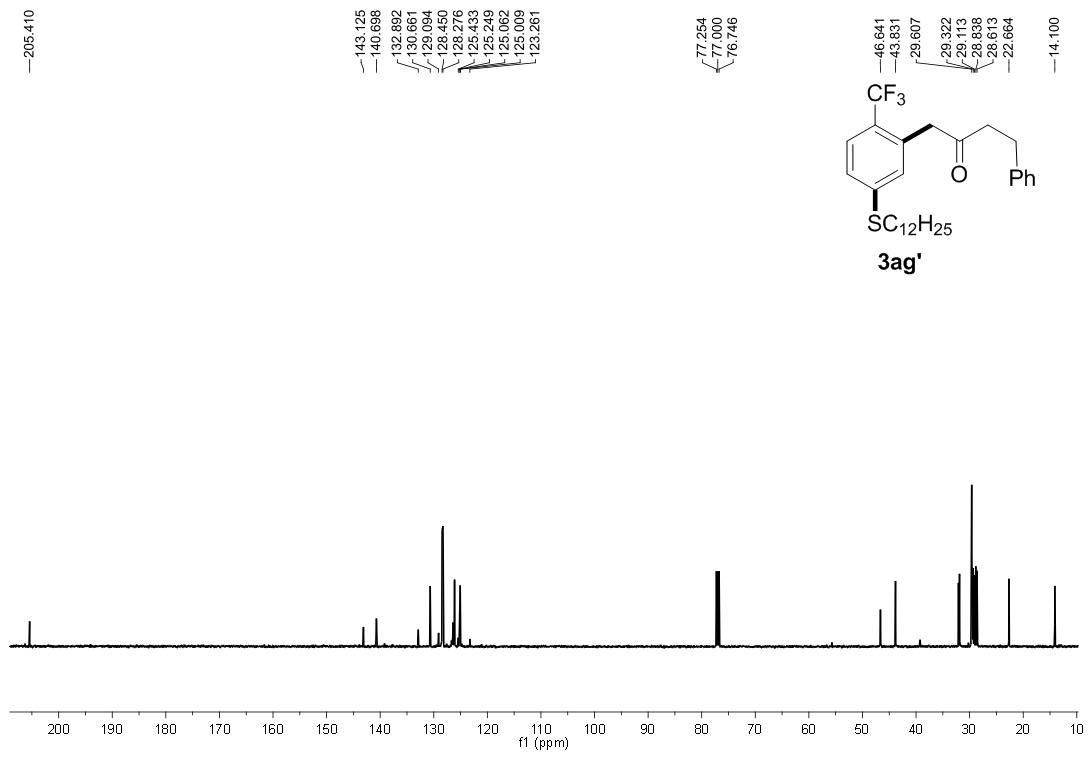


**^1^H NMR** (500 MHz, CDCl_3_) for **3ah**


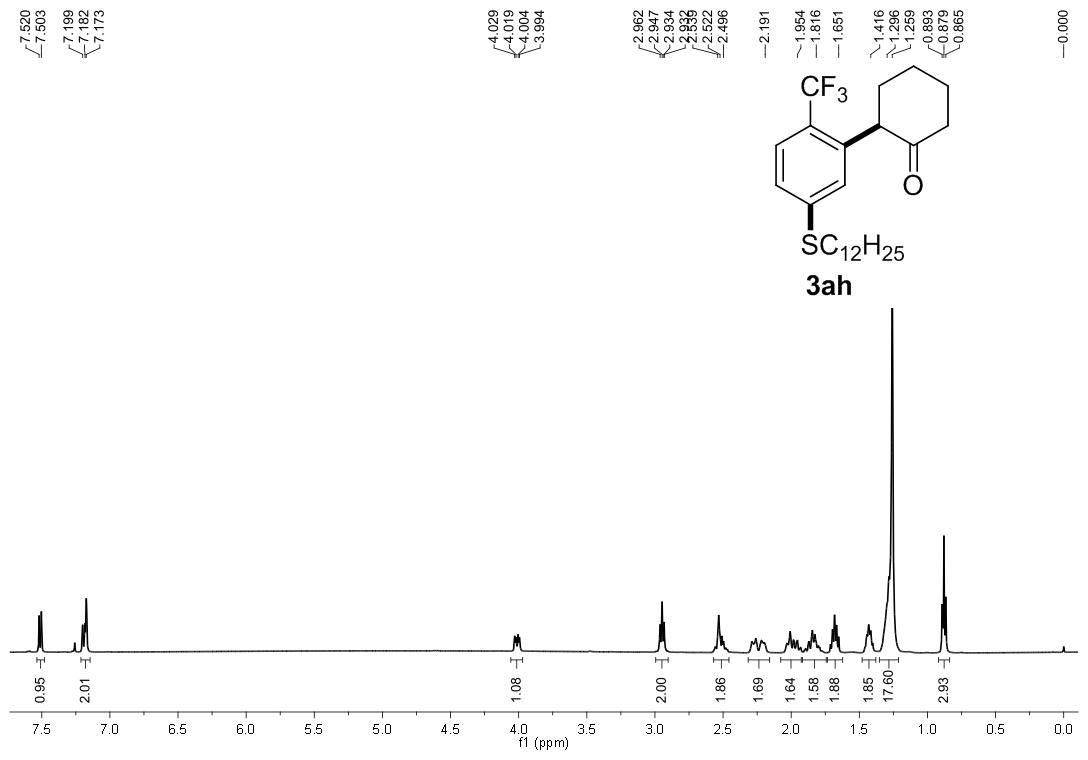


**^13^C NMR** (125 MHz, CDCl_3_) for **3ah**


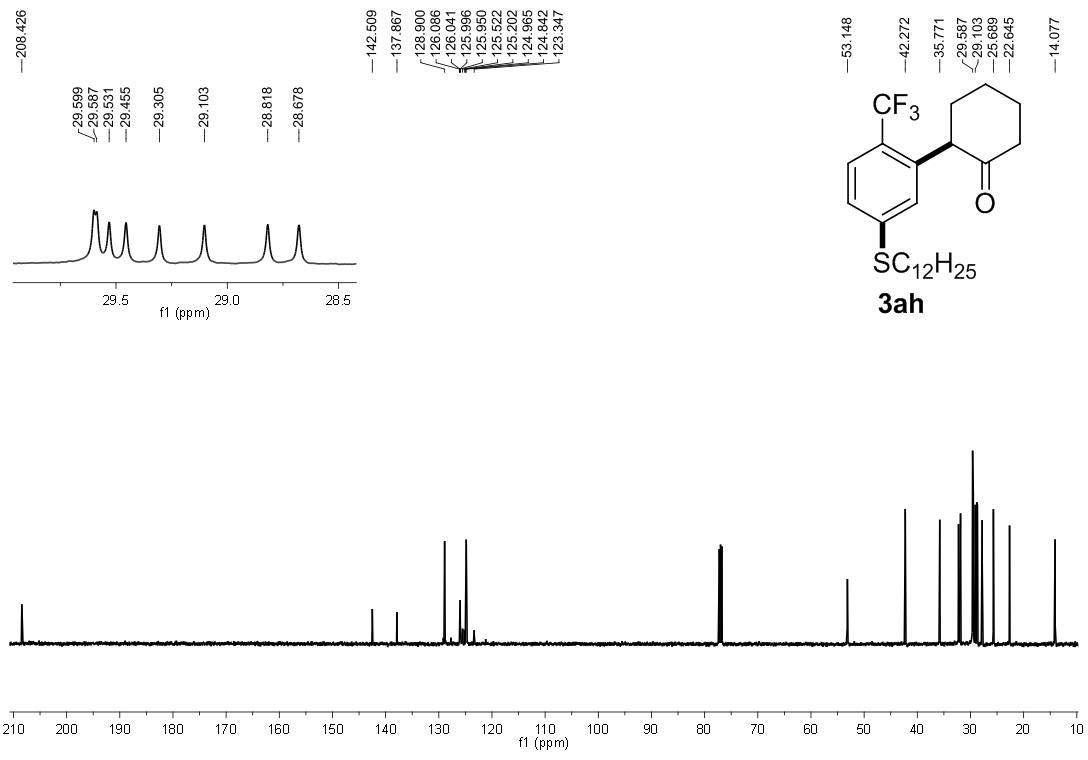


**^19^F NMR** (470 MHz, CDCl_3_) for **3ah**


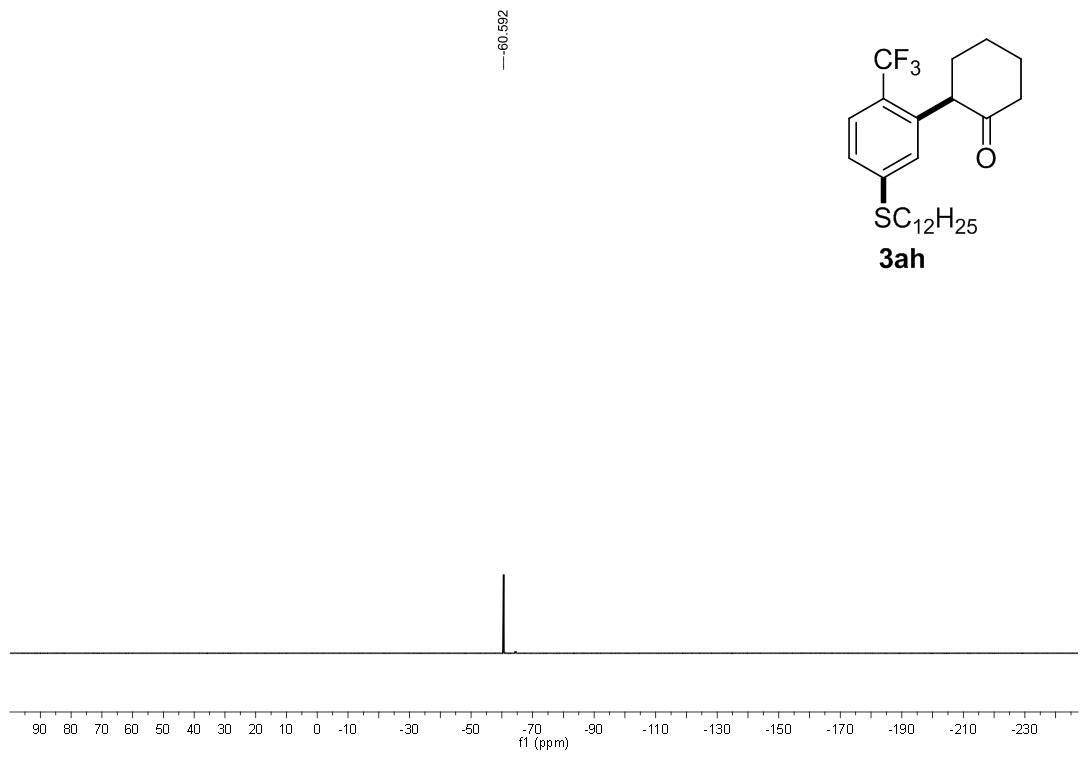


**^1^H NMR** (500 MHz, CDCl_3_) for **3ai**


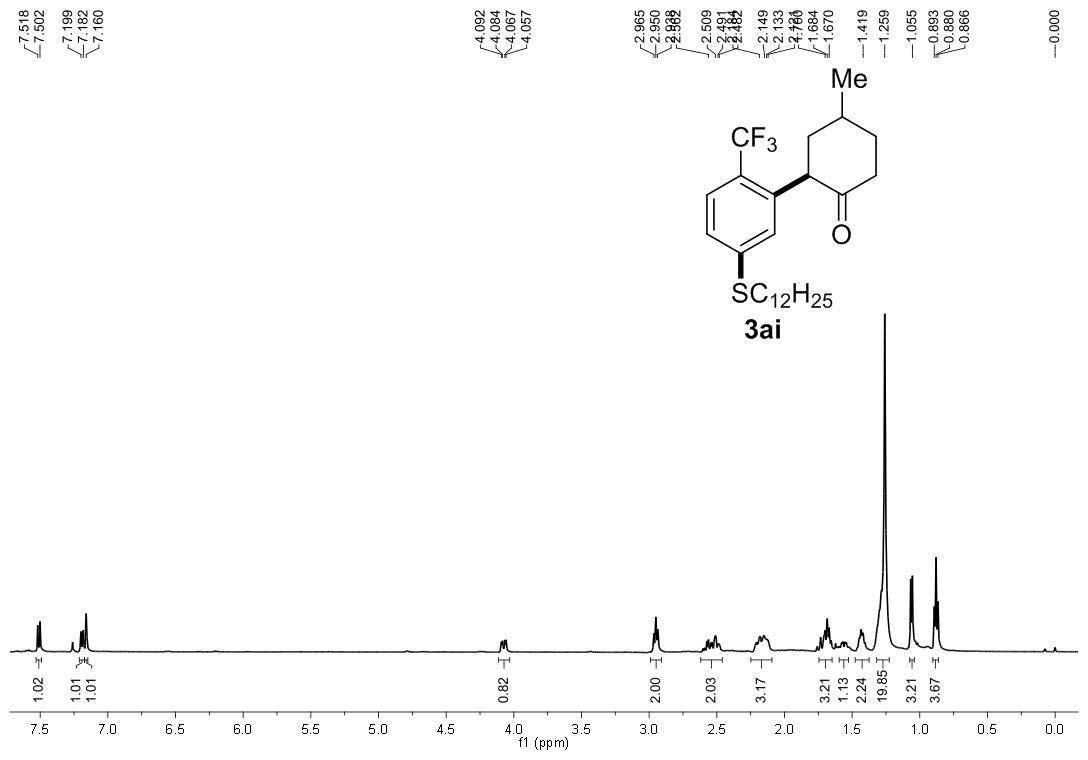


**^13^C NMR** (125 MHz, CDCl_3_) for **3ai**


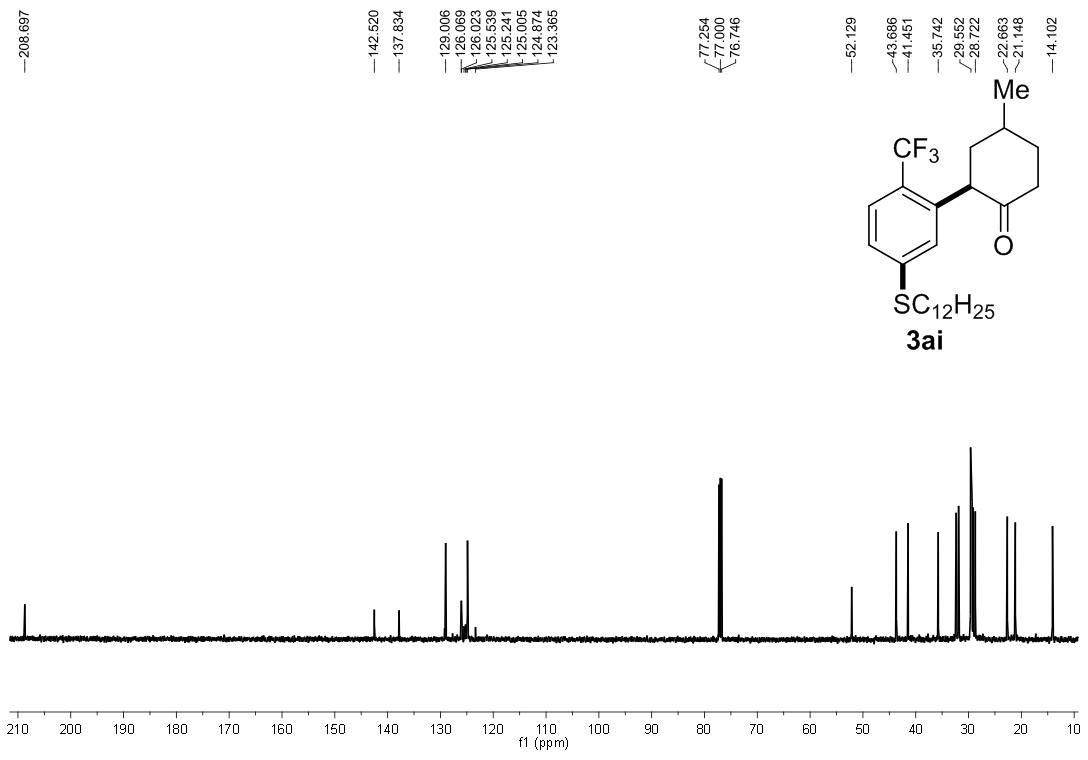


**^1^H NMR** (500 MHz, CDCl_3_) for **3aj**


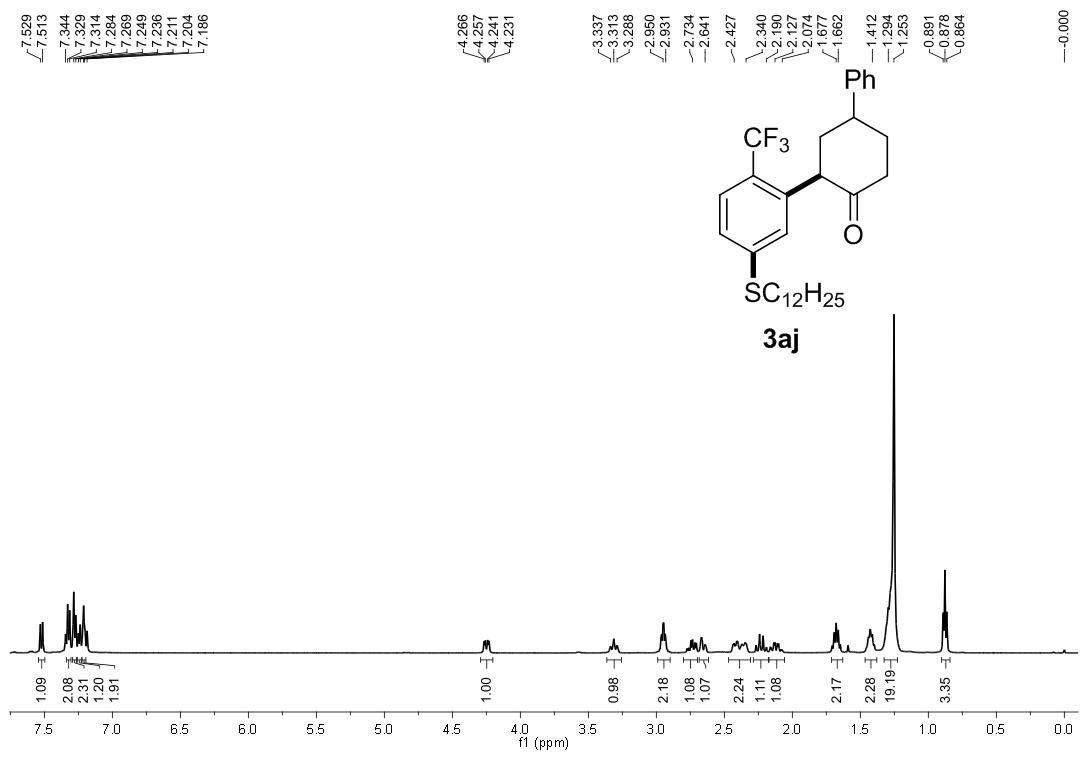


**^13^C NMR** (125 MHz, CDCl_3_) for **3aj**


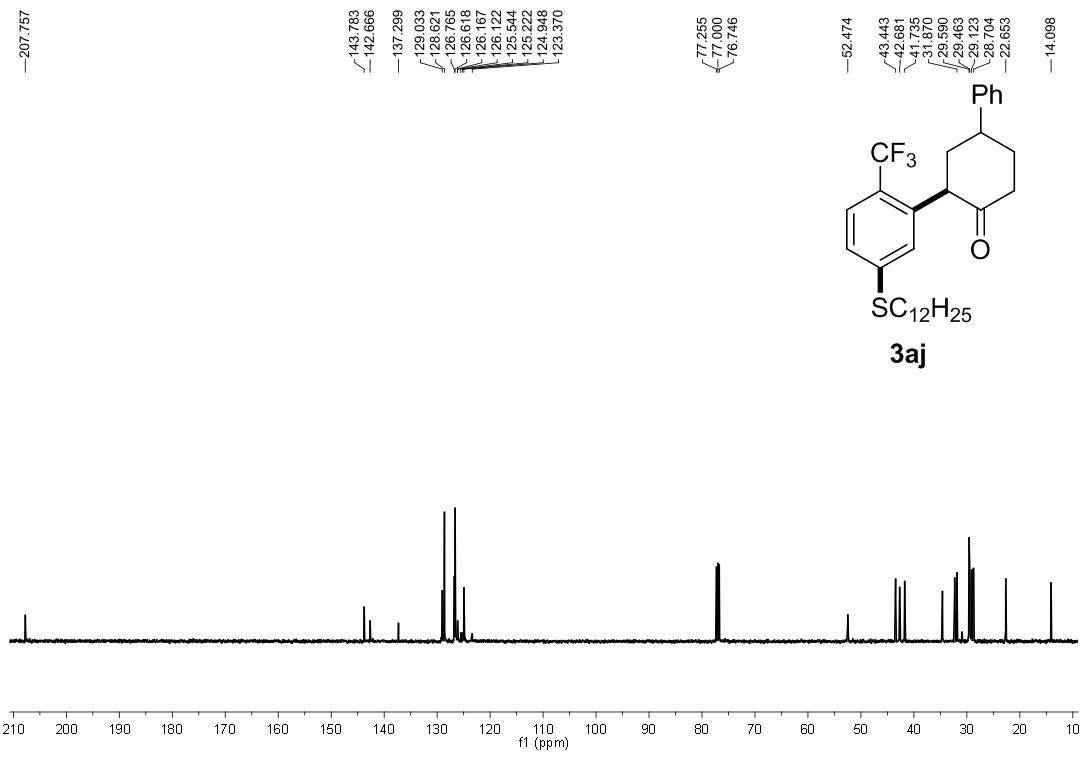


**^1^H NMR** (500 MHz, CDCl_3_) for **3ak**


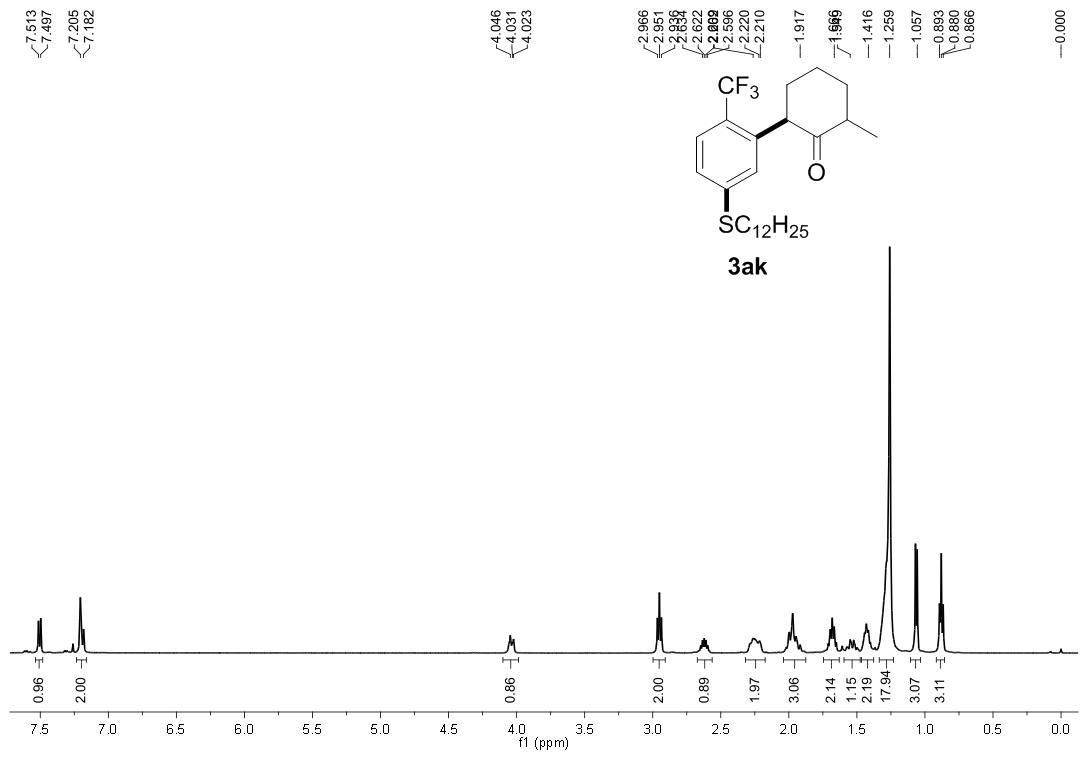


**^13^C NMR** (125 MHz, CDCl_3_) for **3ak**


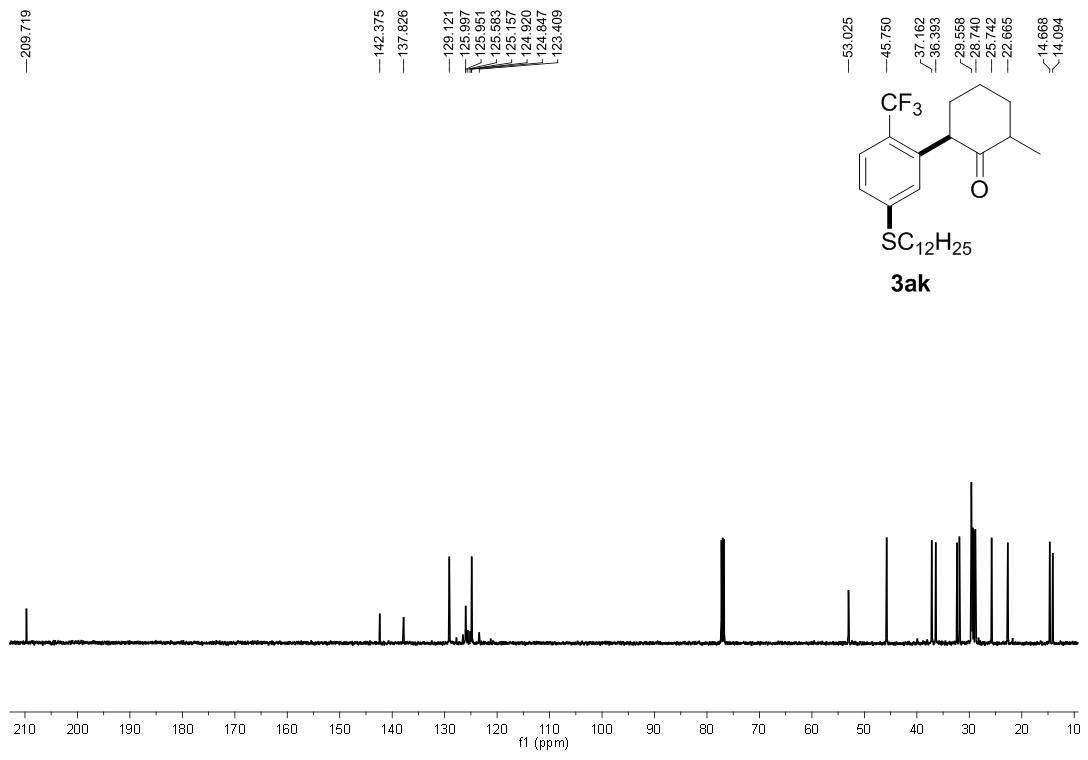


**^1^H NMR** (500 MHz, CDCl_3_) for **3ak’**


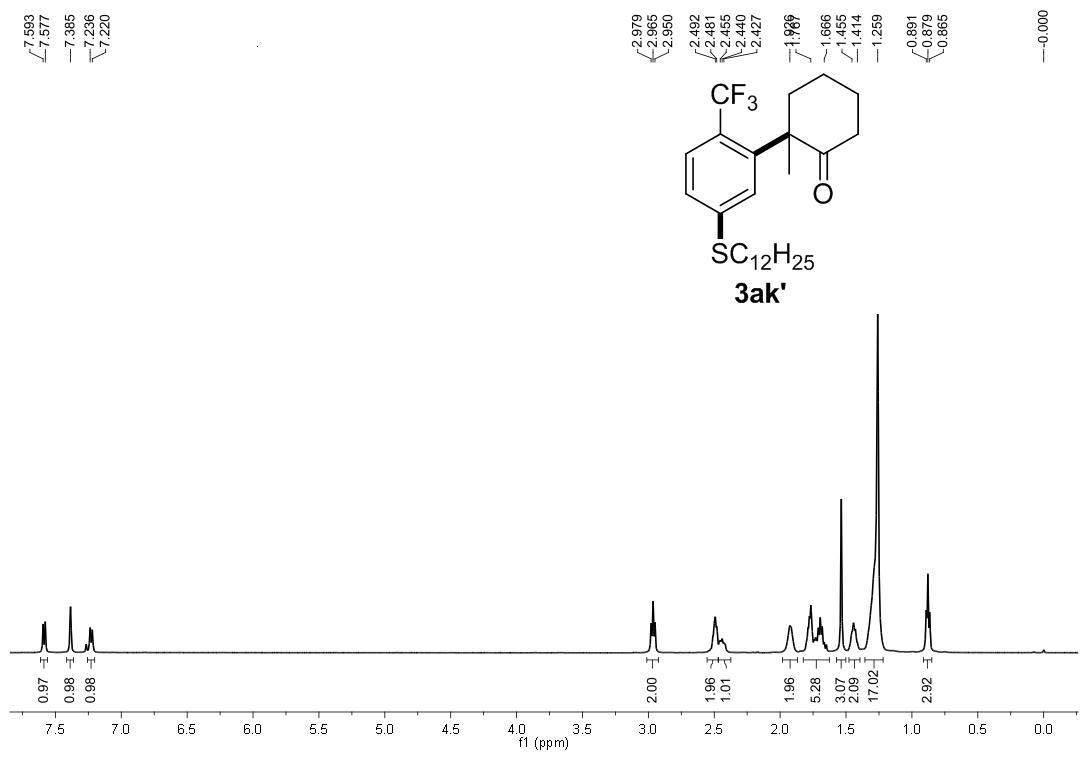


**^13^C NMR** (125 MHz, CDCl_3_) for **3ak’**


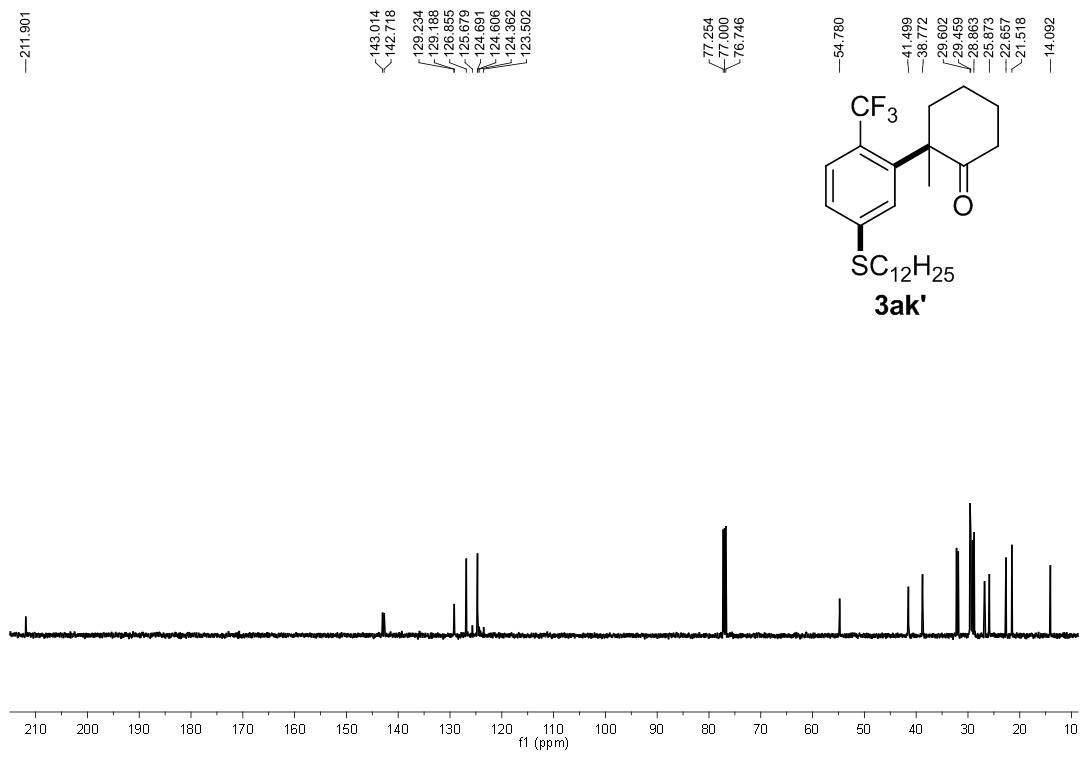


**^1^H NMR** (500 MHz, CDCl_3_) for **3al**


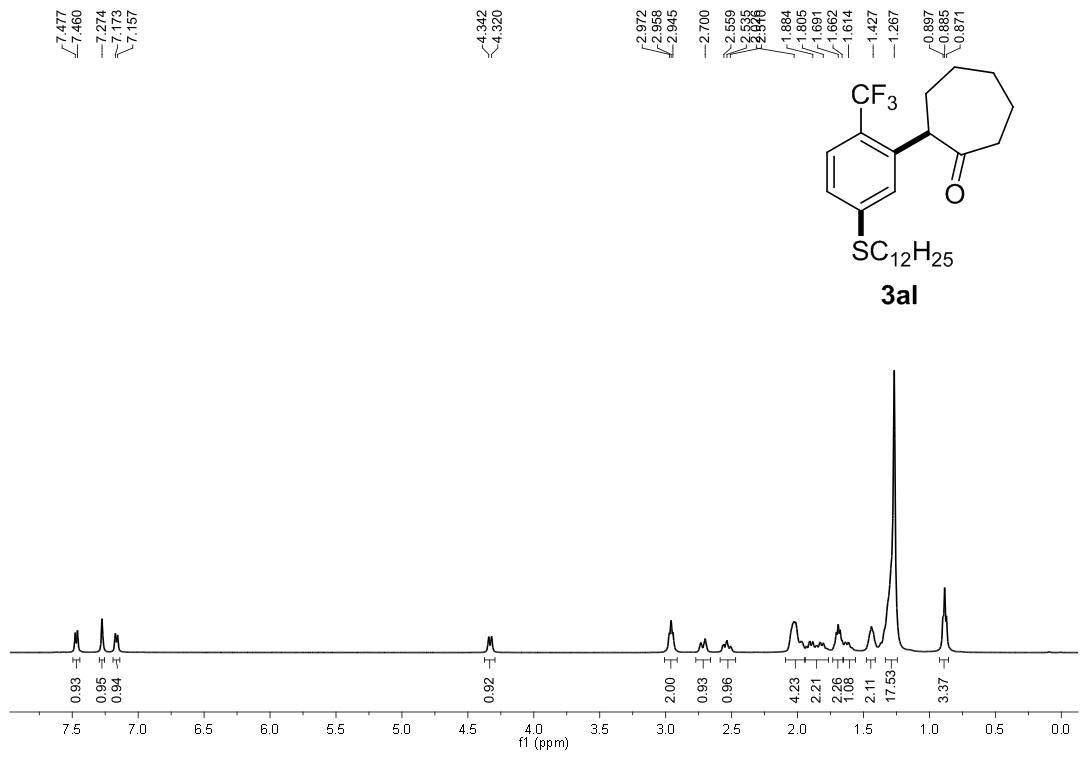


**^13^C NMR** (125 MHz, CDCl_3_) for **3al**


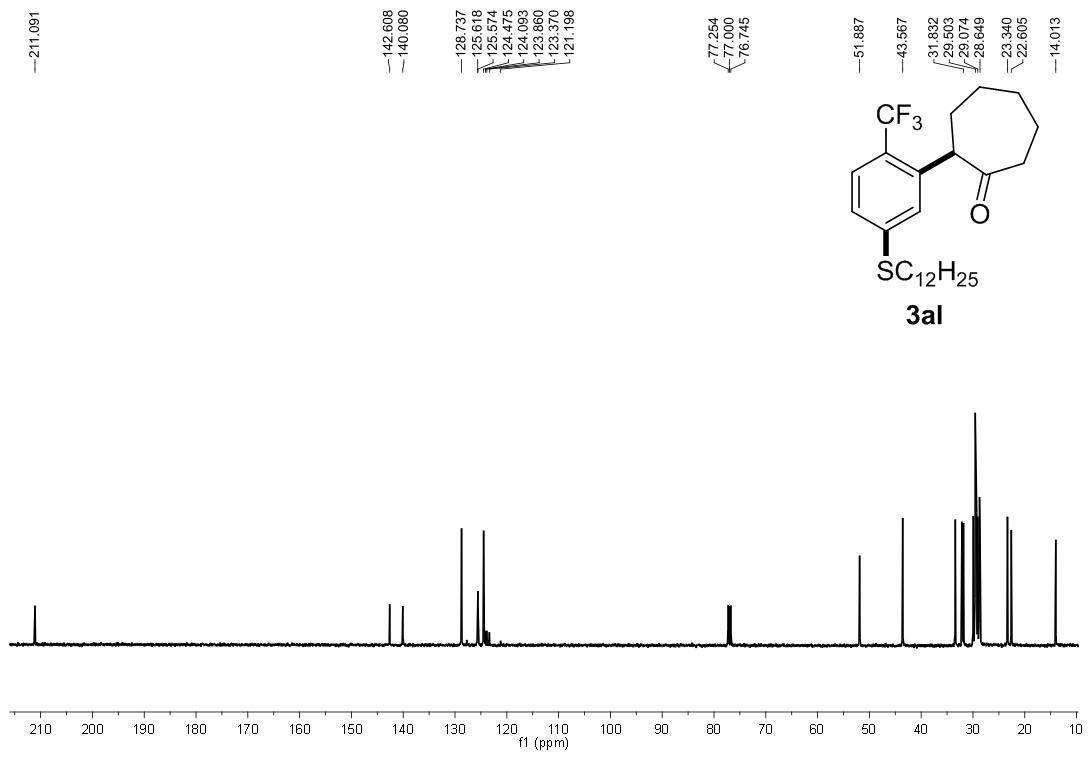


**^1^H NMR** (500 MHz, CDCl_3_) for **3am**


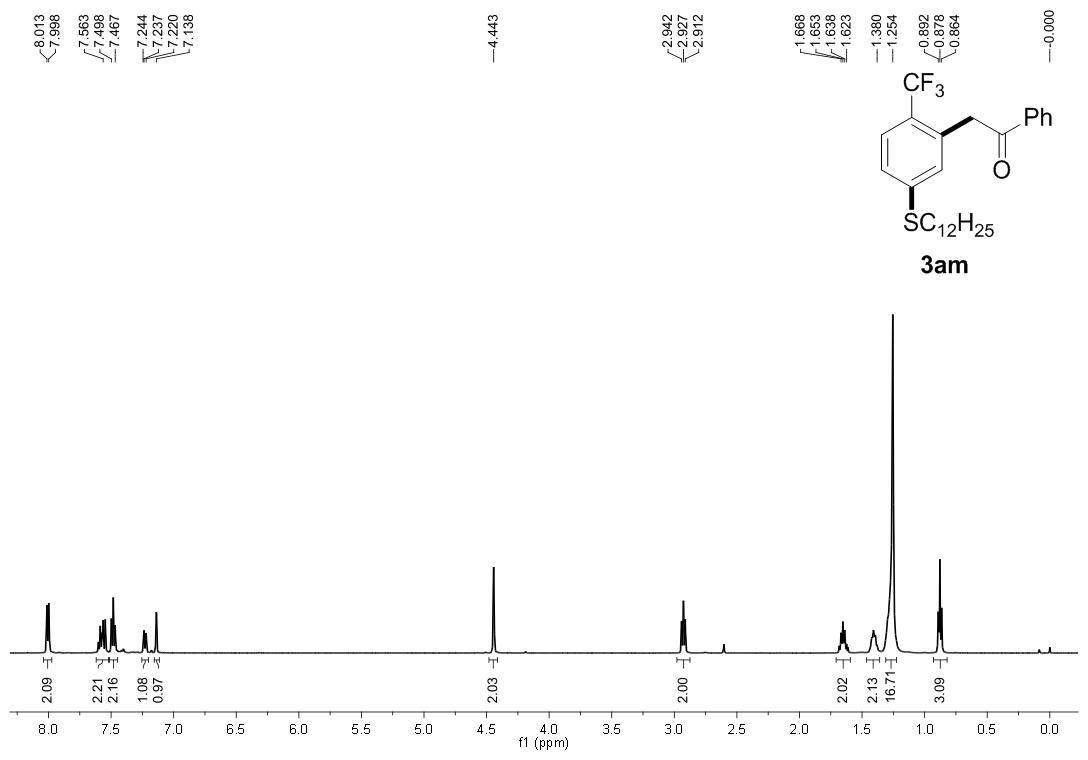


**^13^C NMR** (125 MHz, CDCl_3_) for **3am**


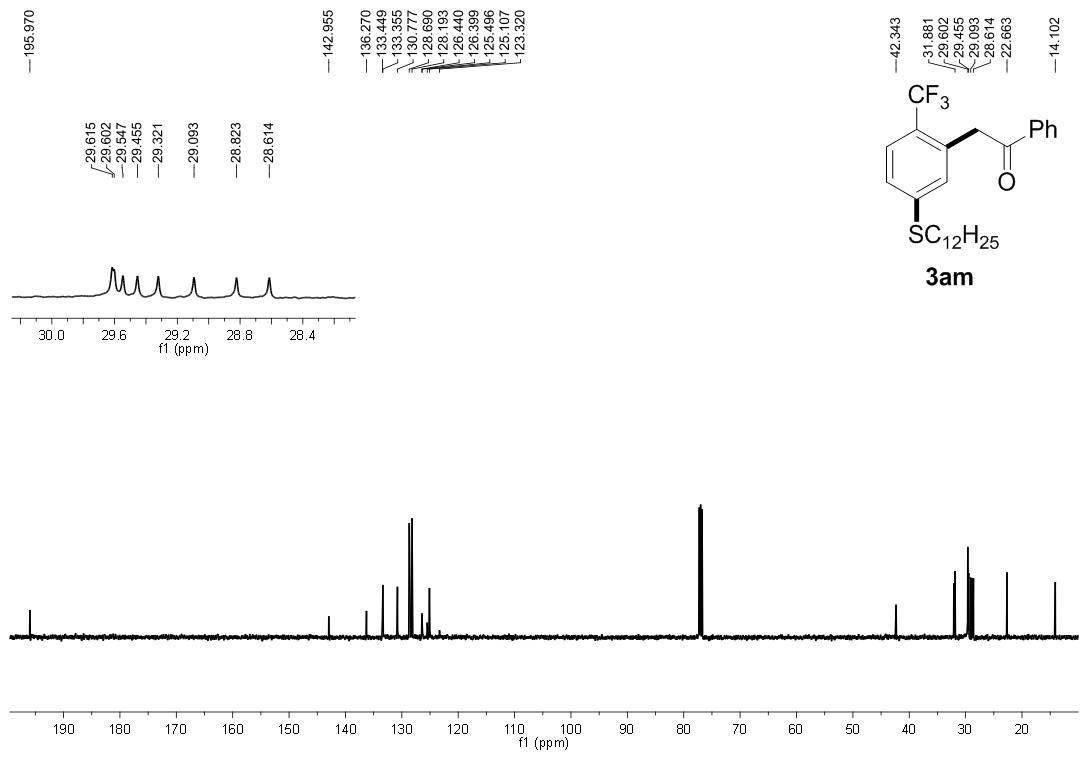


**^1^H NMR** (500 MHz, CDCl_3_) for **3an**


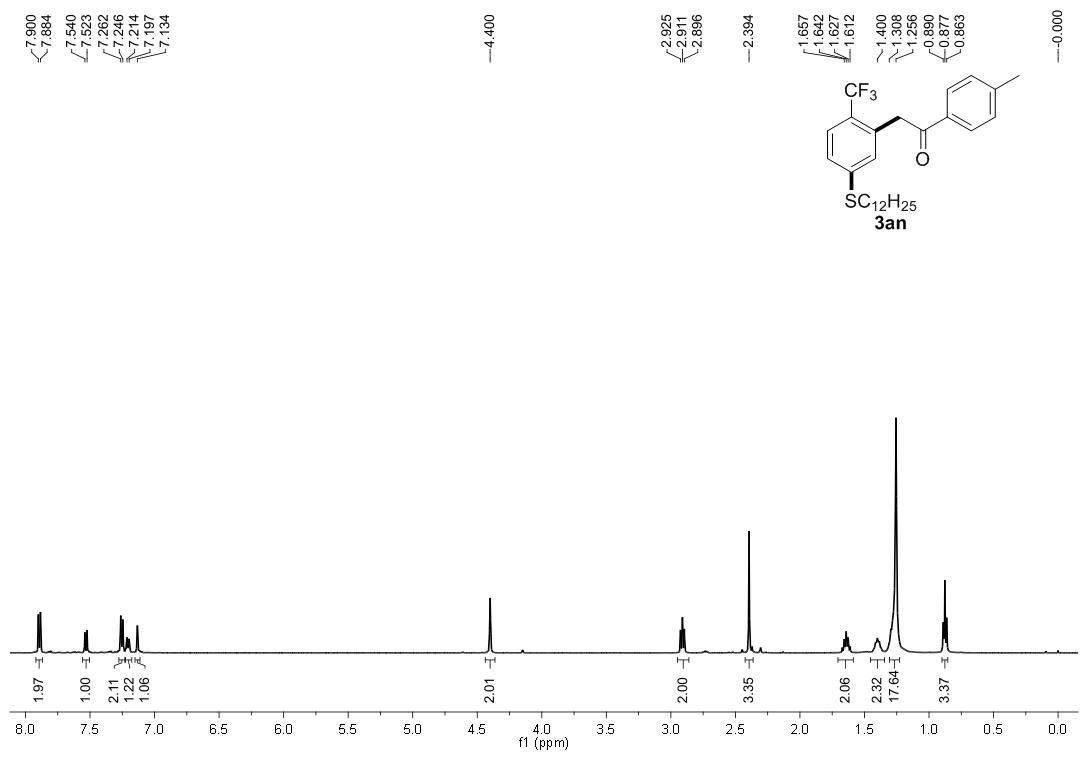


**^13^C NMR** (125 MHz, CDCl_3_) for **3an**


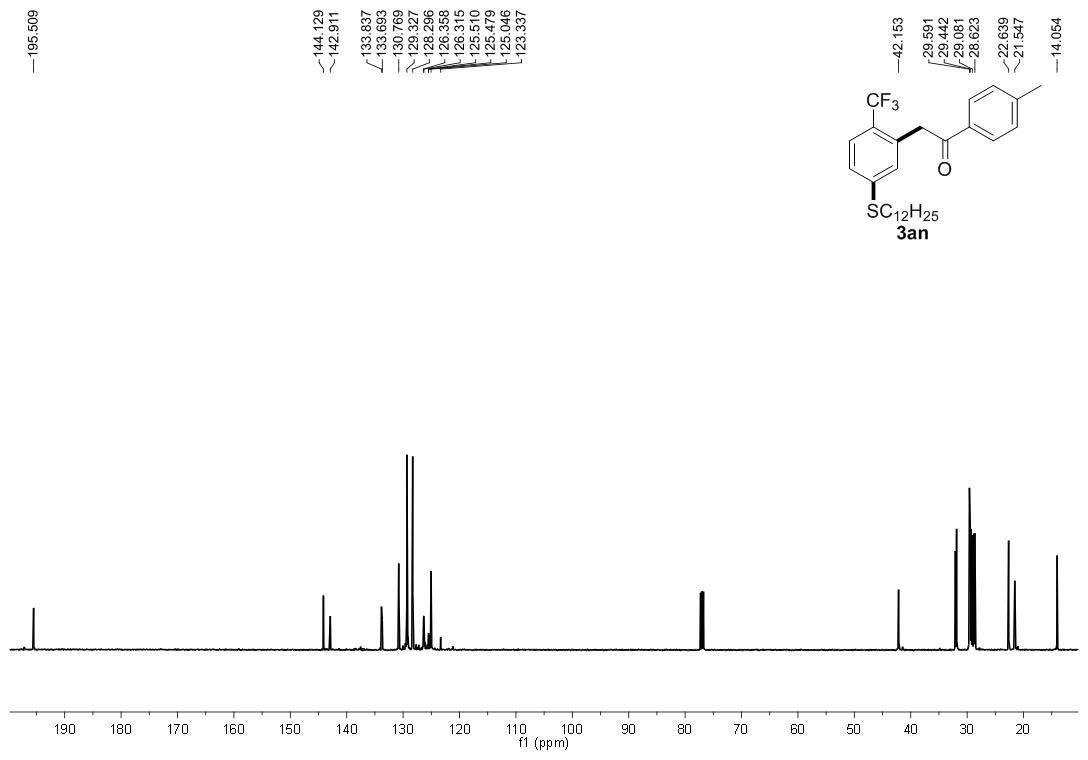


**^19^F NMR** (470 MHz, CDCl_3_) for **3an**


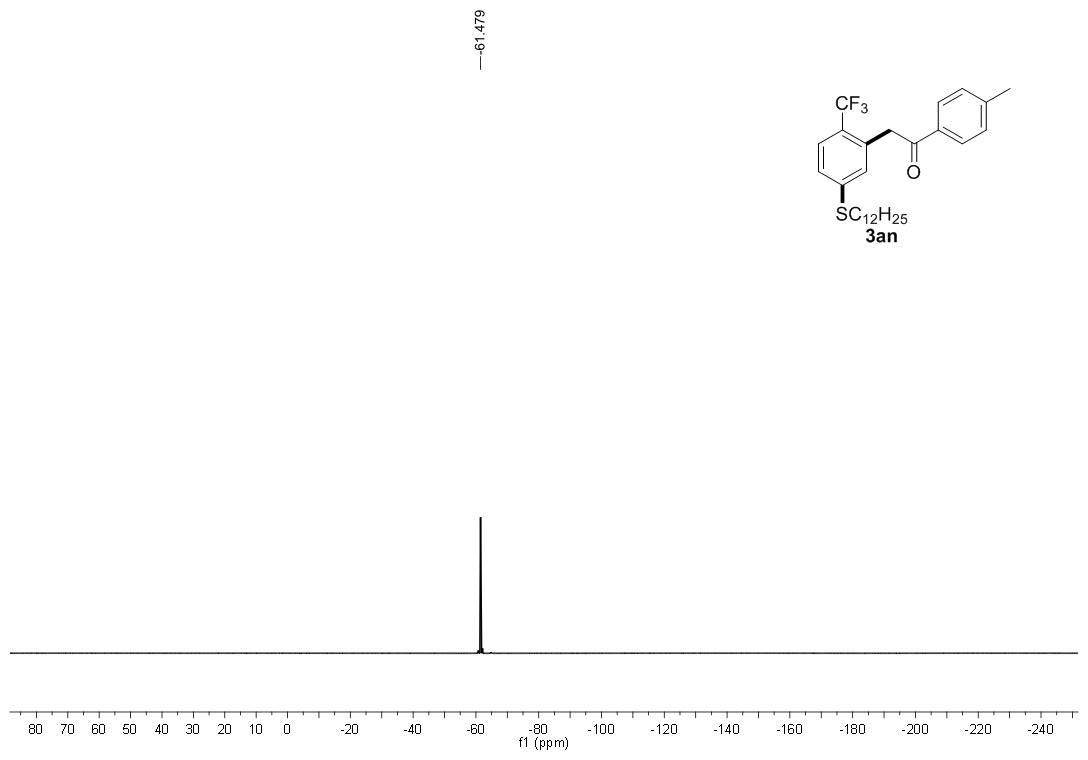


**^1^H NMR** (500 MHz, CDCl_3_) for **3ao**


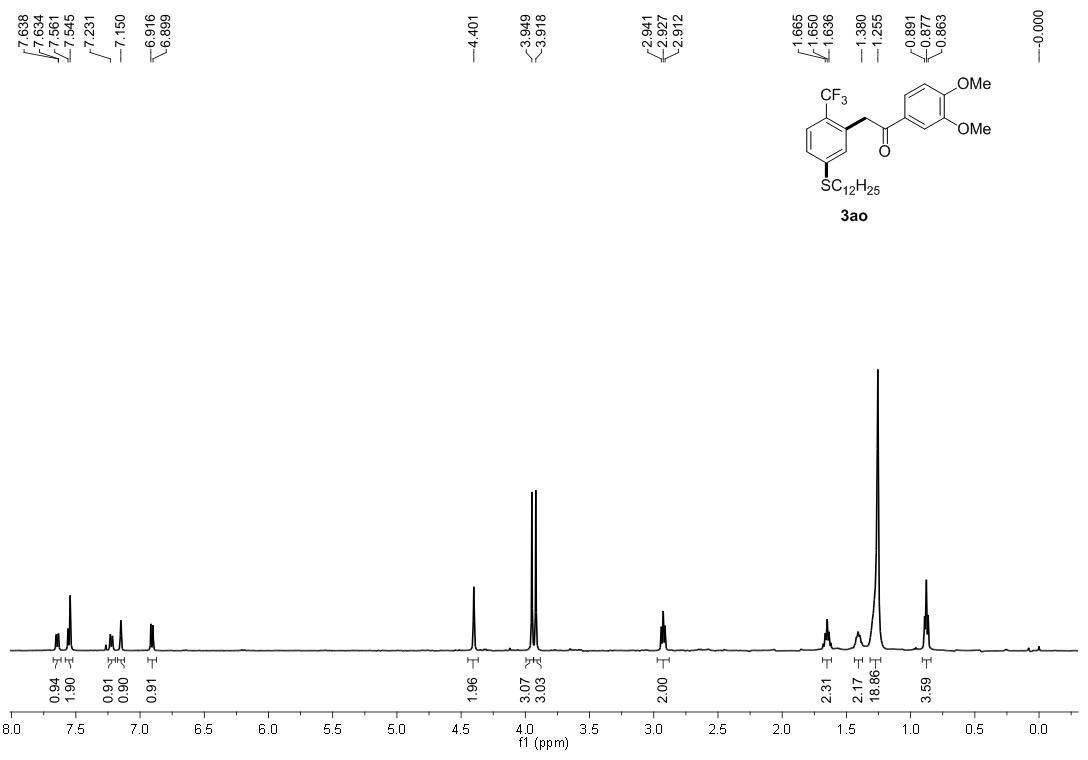


**^13^C NMR** (125 MHz, CDCl_3_) for **3ao**


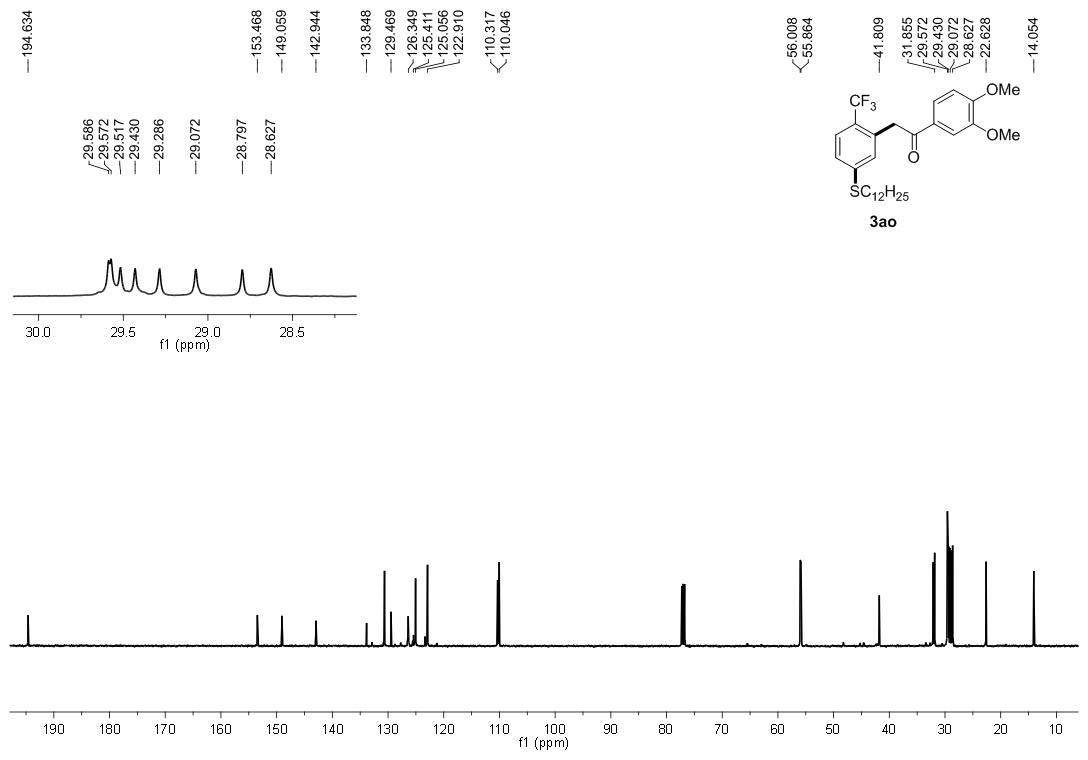


**^19^F NMR** (470 MHz, CDCl_3_) for **3ao**


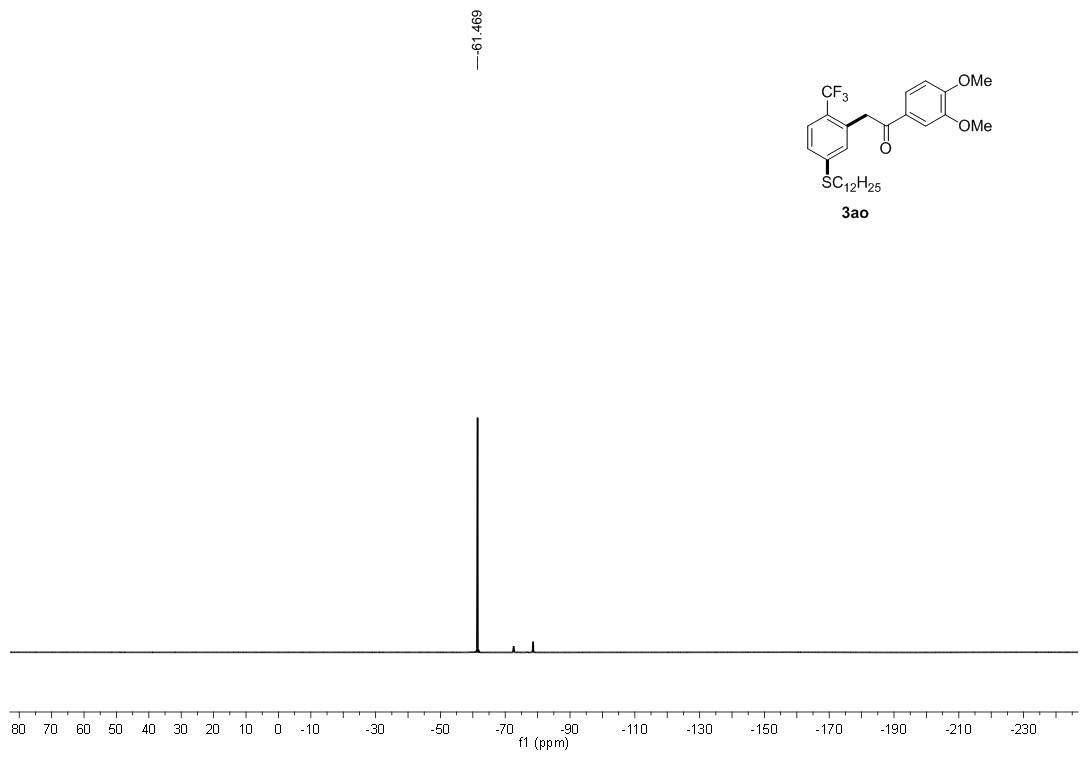


**^1^H NMR** (500 MHz, CDCl_3_) for **3ap**


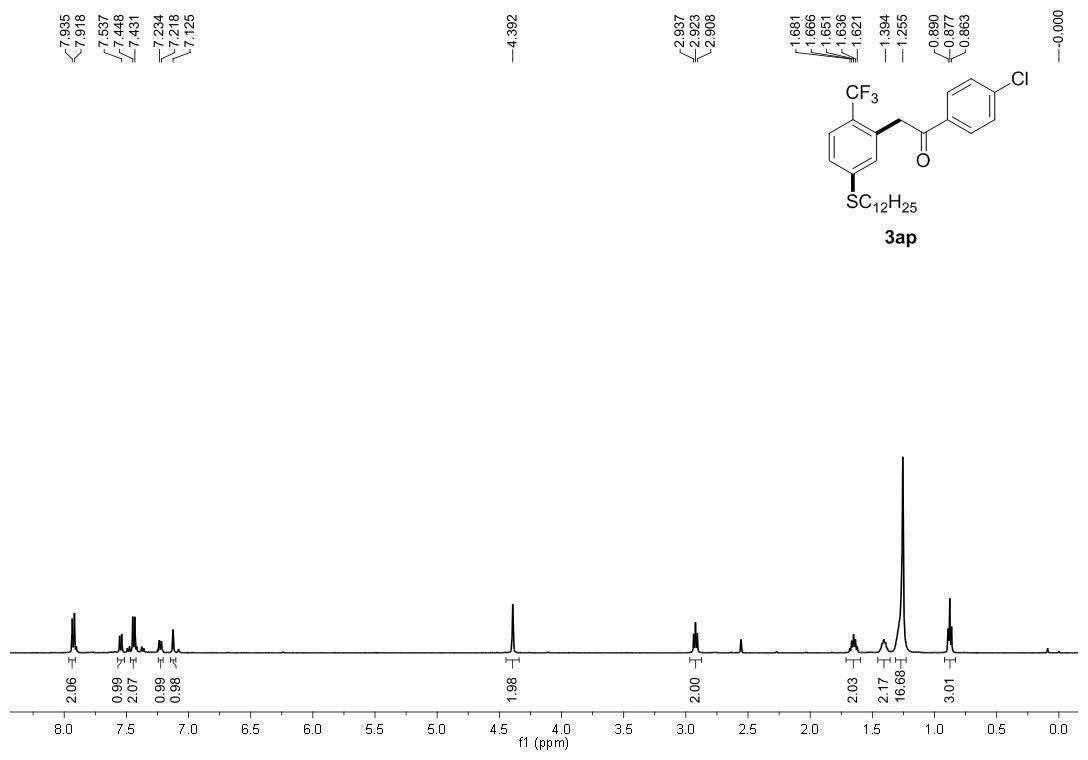


**^13^C NMR** (125 MHz, CDCl_3_) for **3ap**


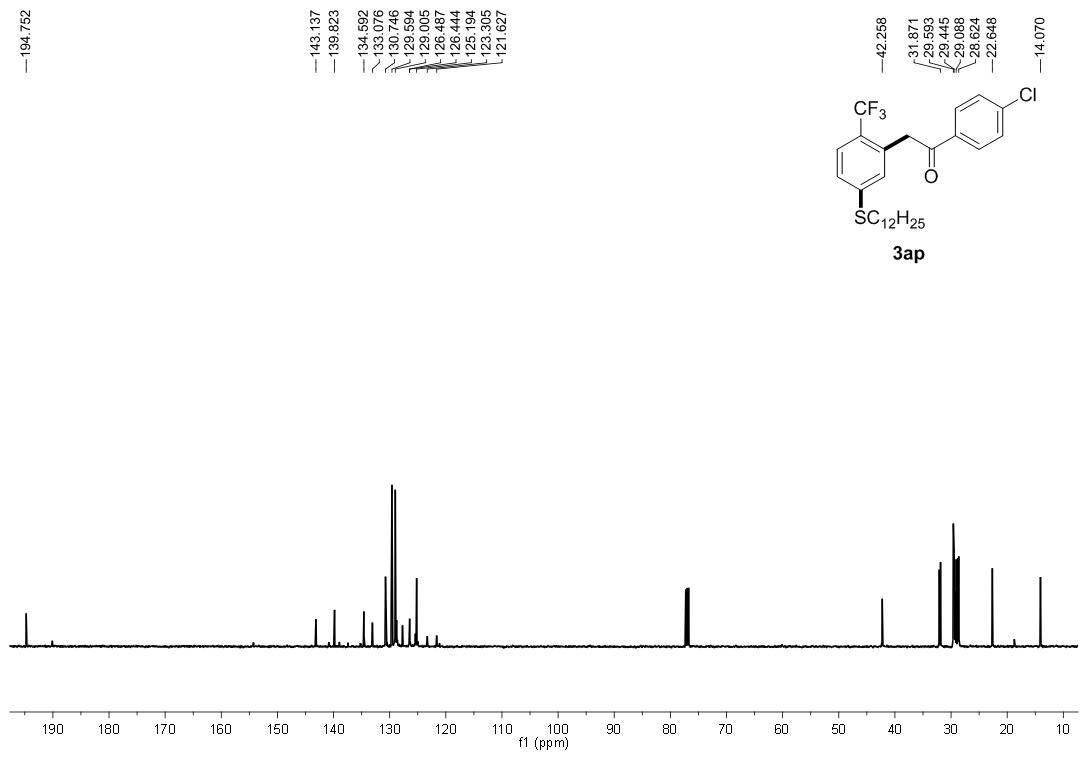


**^1^H NMR** (500 MHz, CDCl_3_) for **3aq**


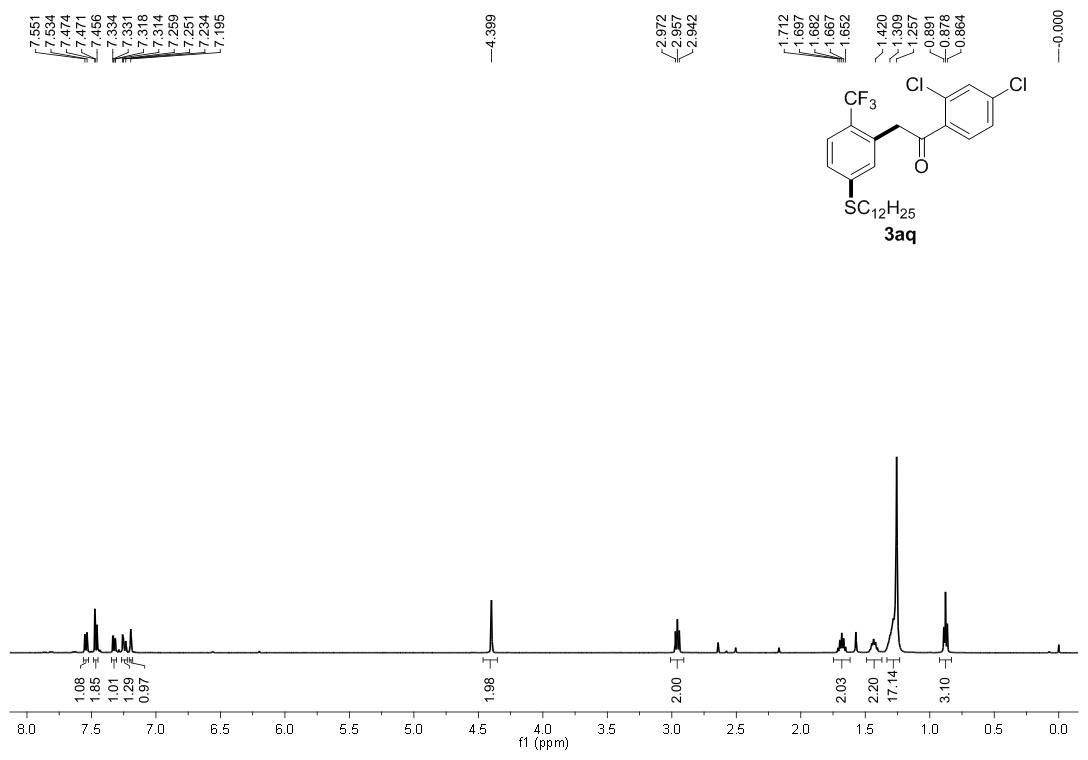


**^13^C NMR** (125 MHz, CDCl_3_) for **3aq**


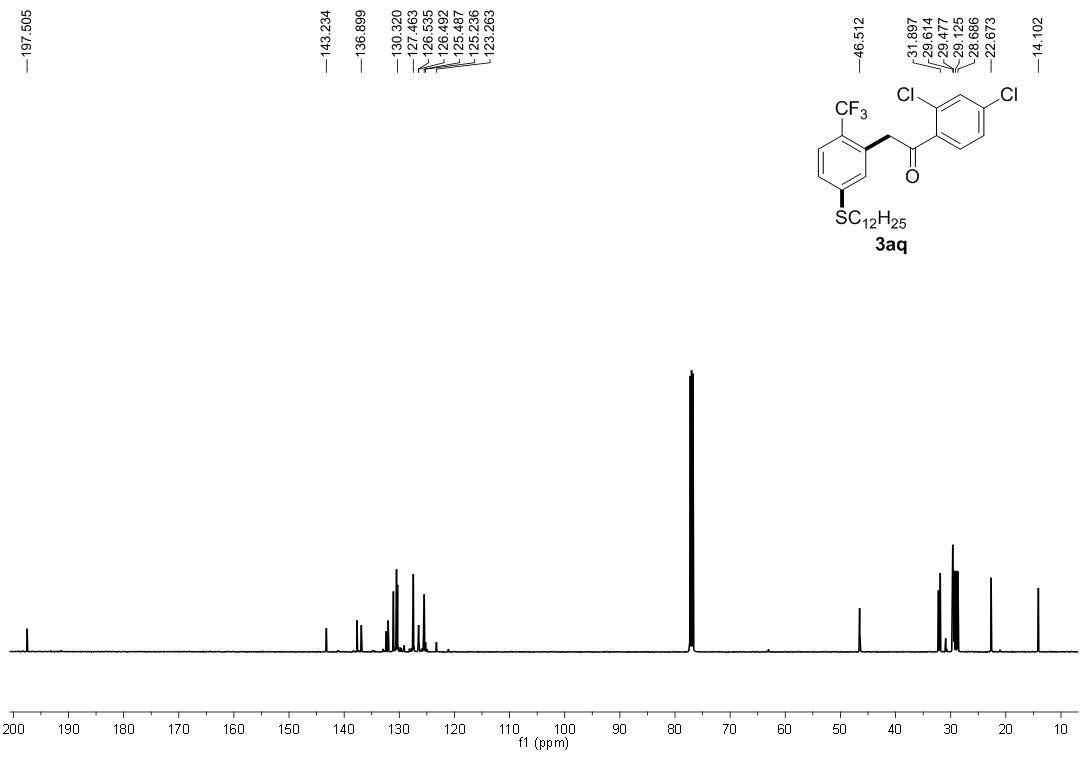


**^1^H NMR** (500 MHz, CDCl_3_) for **3ar**


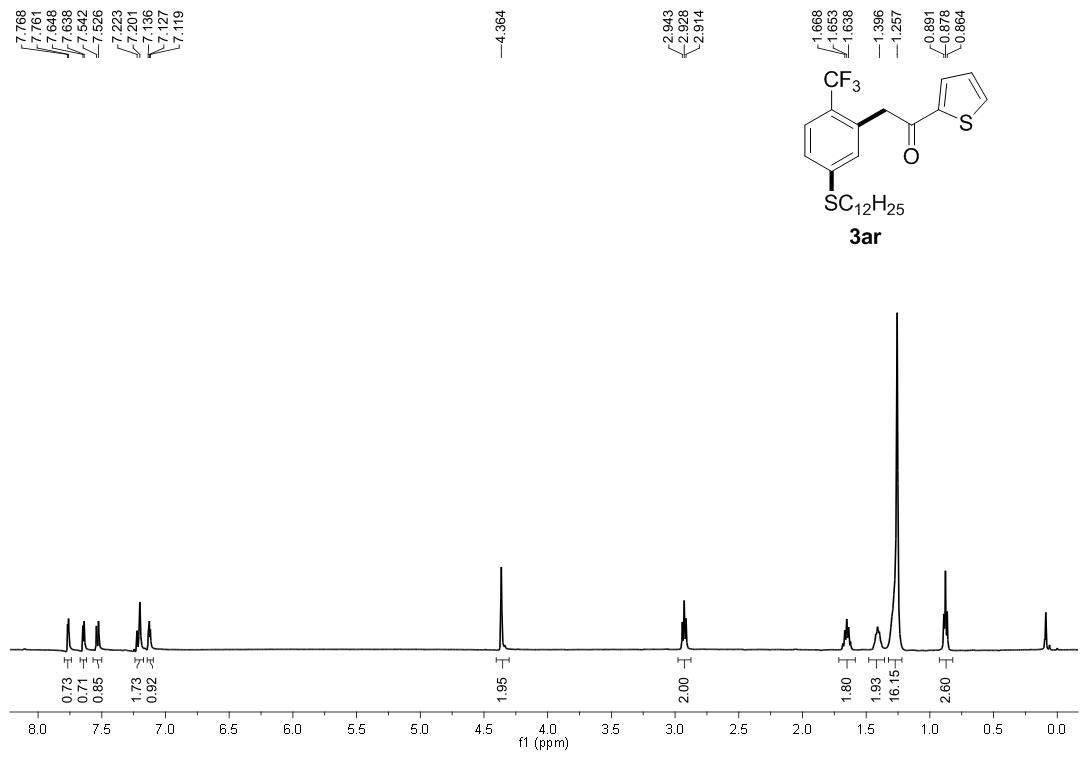


**^13^C NMR** (125 MHz, CDCl_3_) for **3ar**


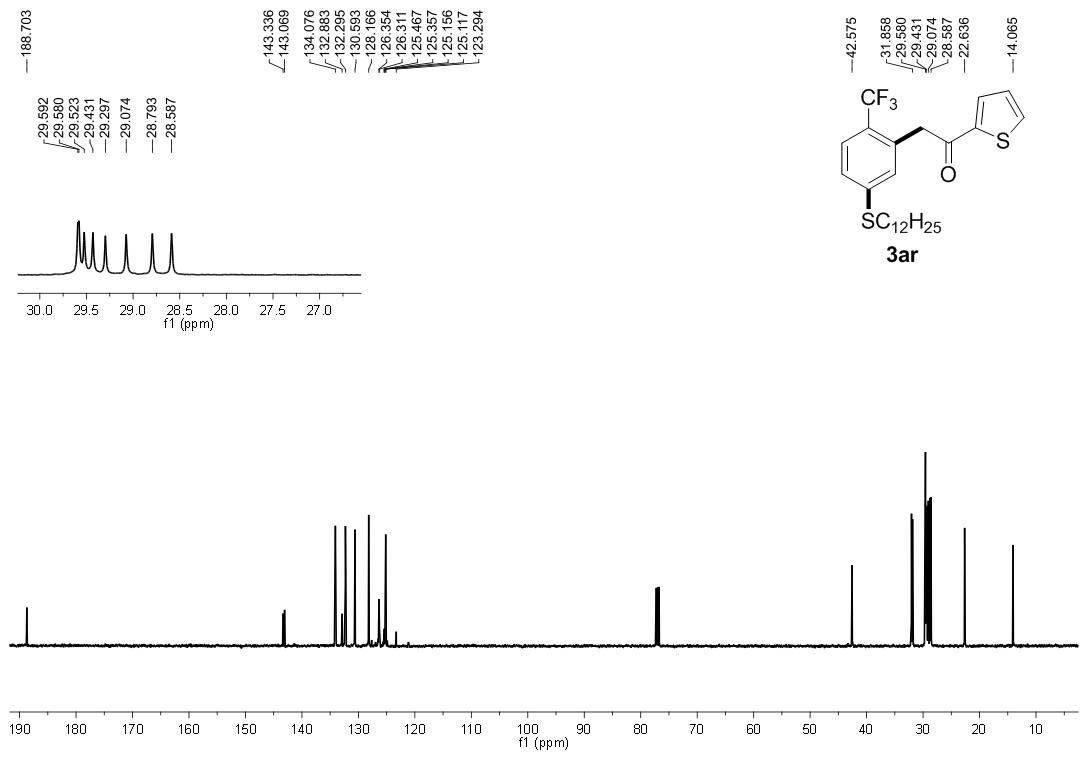


**^19^F NMR** (470 MHz, CDCl_3_) for **3ar**


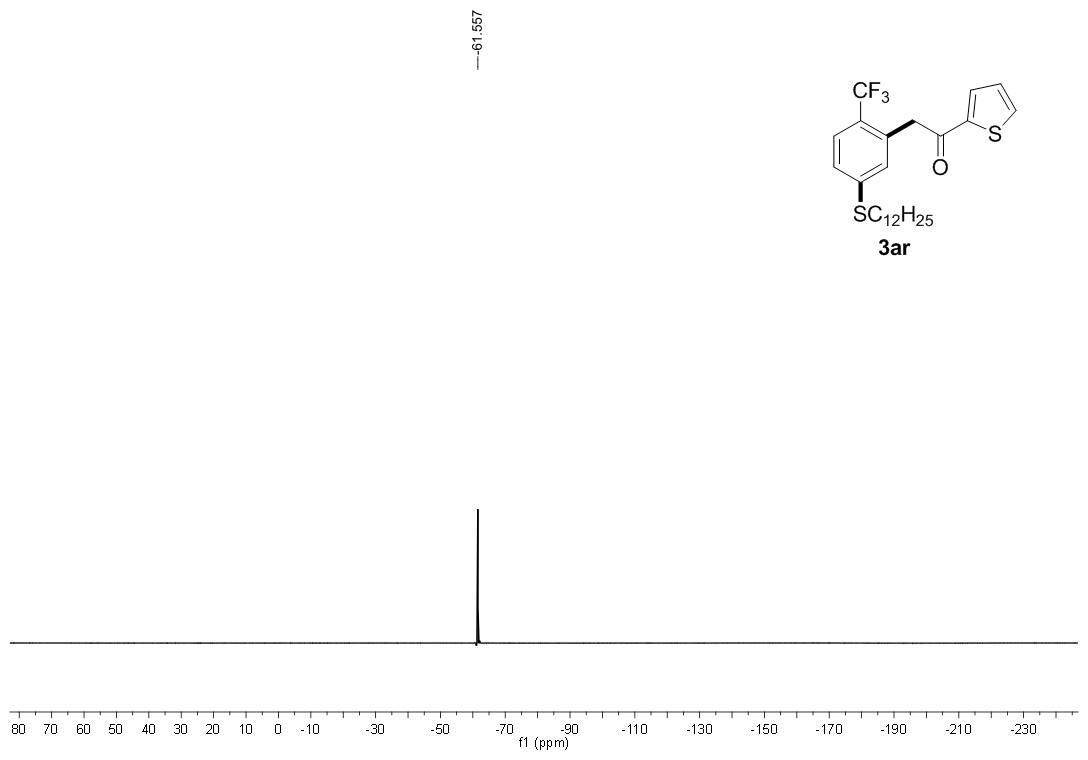


**^1^H NMR** (500 MHz, CDCl_3_) for **3as**


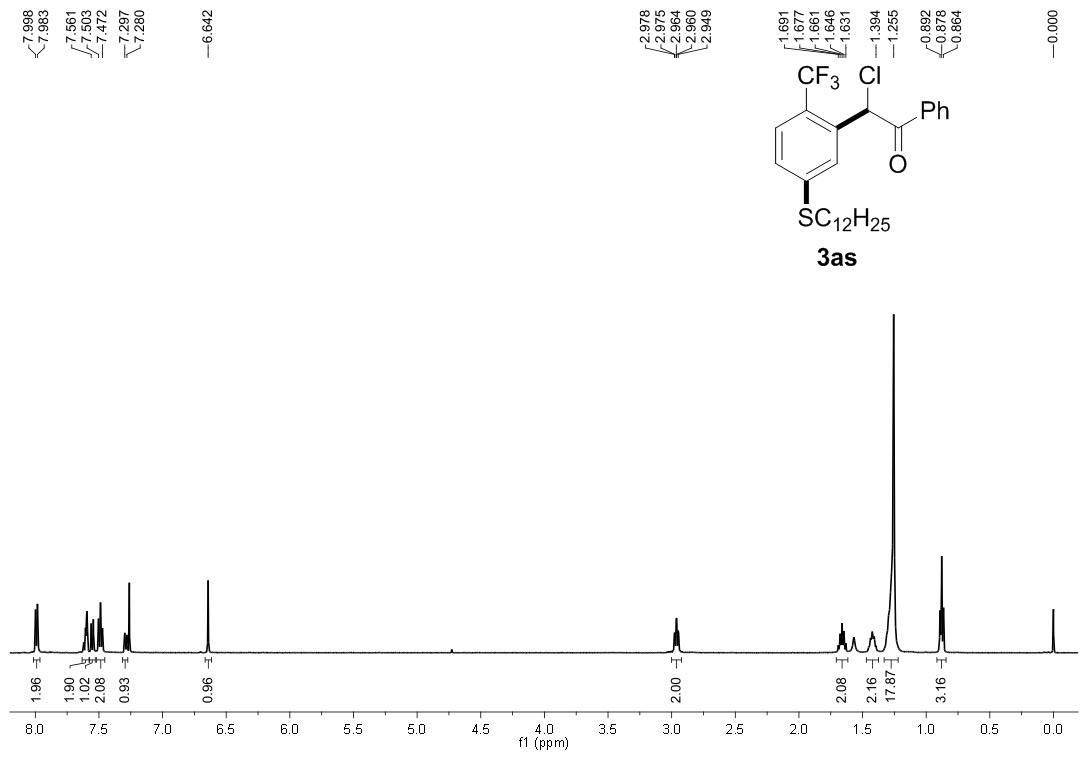


**^13^C NMR** (125 MHz, CDCl_3_) for **3as**


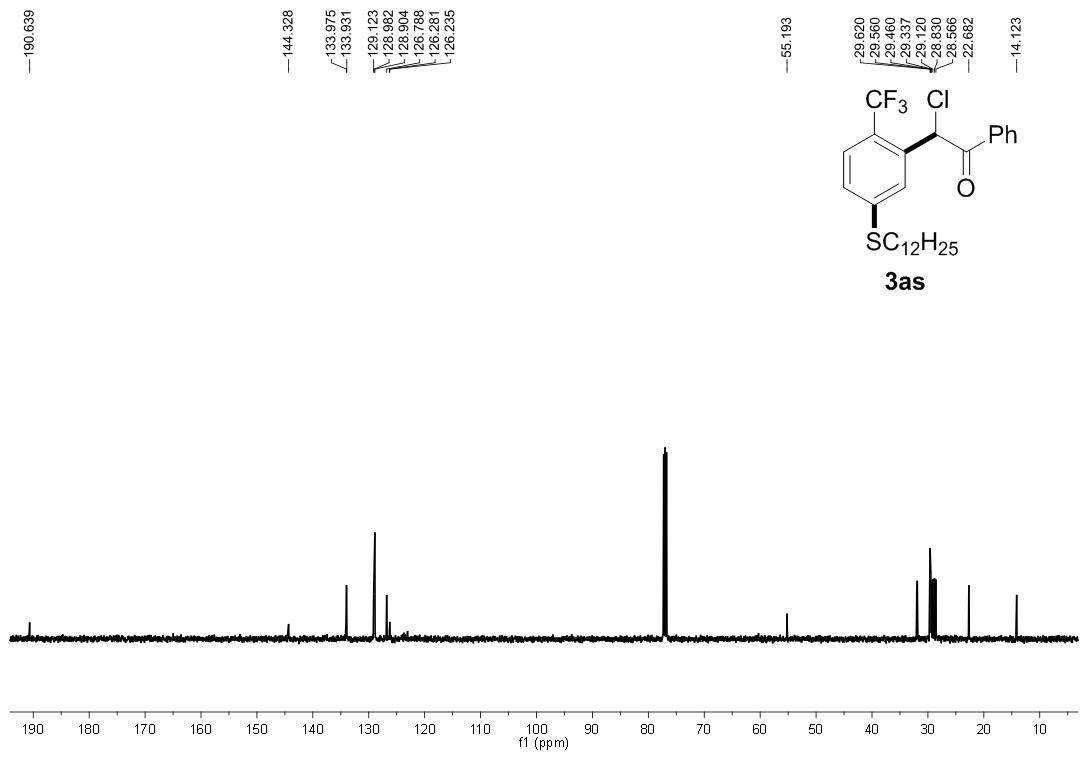


**^19^F NMR** (470 MHz, CDCl_3_) for **3as**


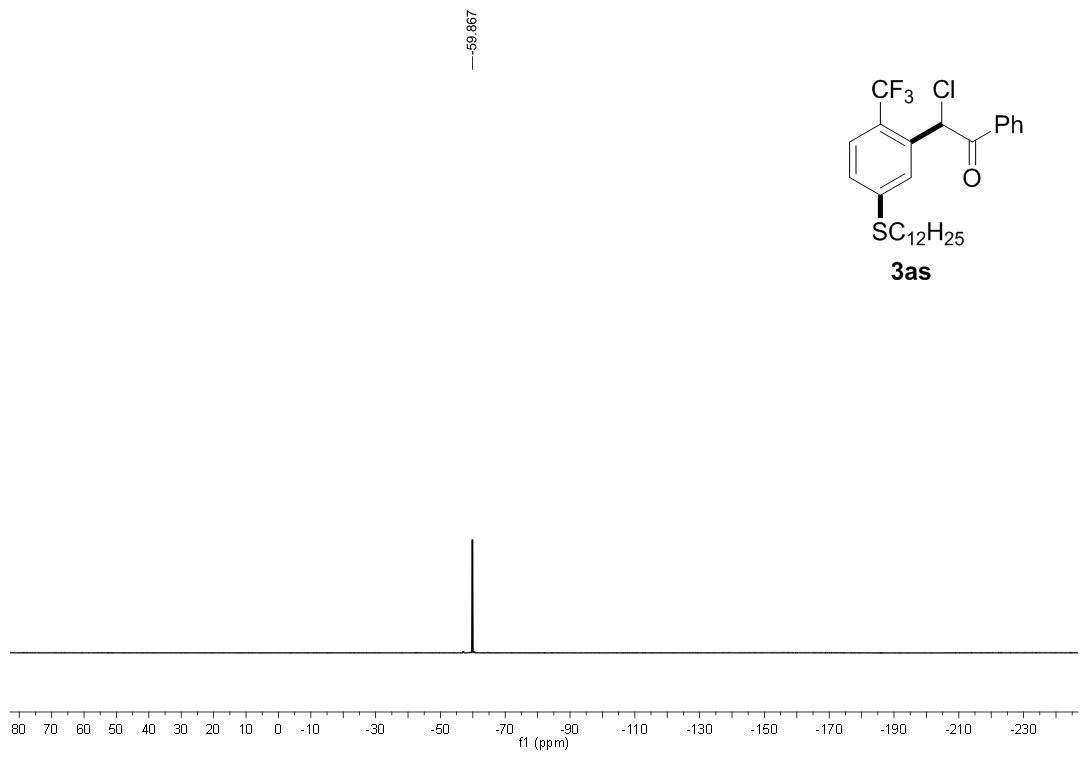


**^1^H NMR** (500 MHz, CDCl_3_) for **3at**


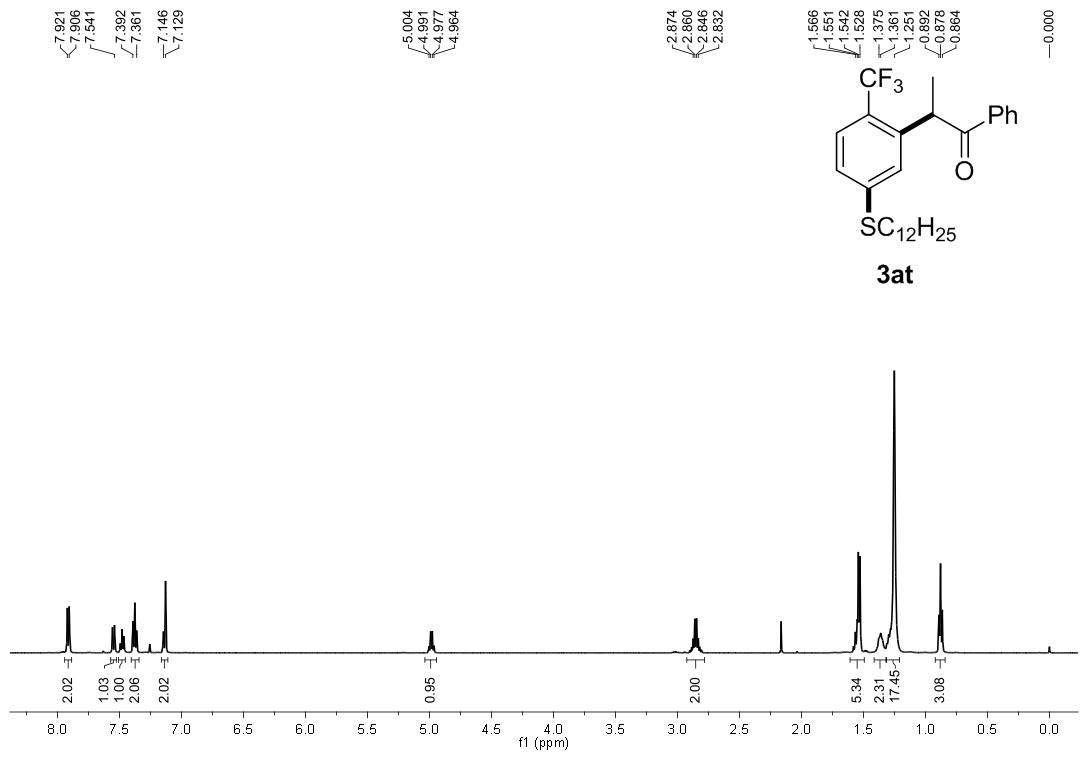


**^13^C NMR** (125 MHz, CDCl_3_) for **3at**


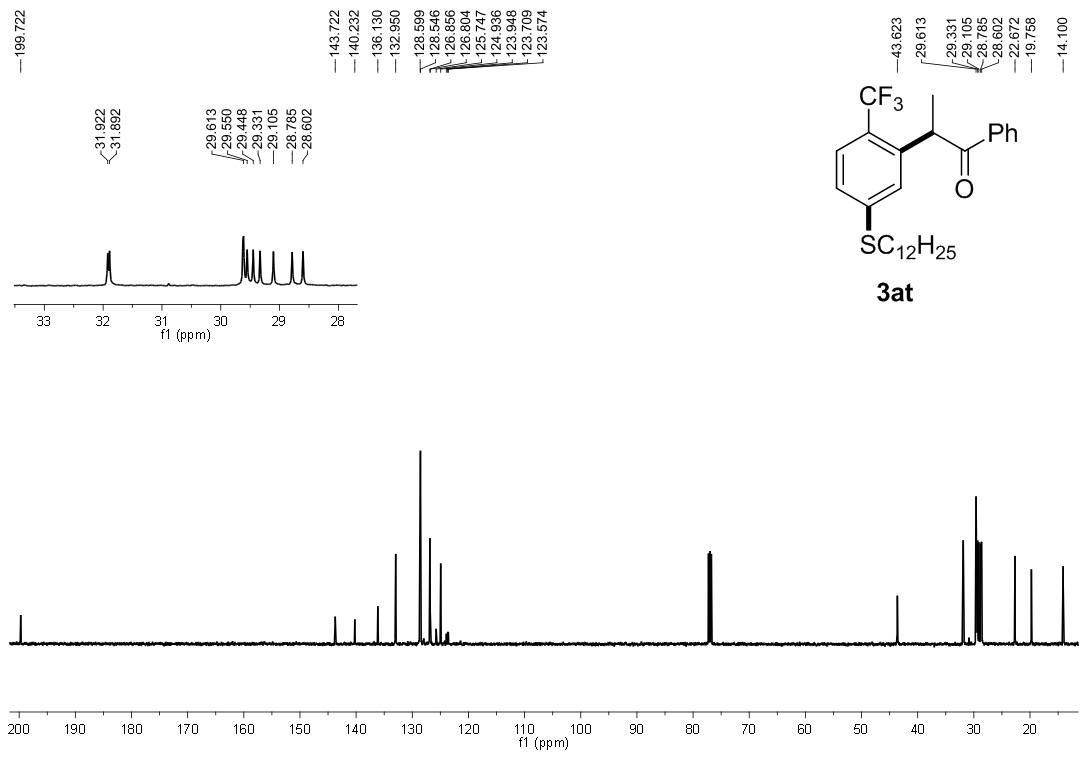


**^19^F NMR** (470 MHz, CDCl_3_) for **3at**


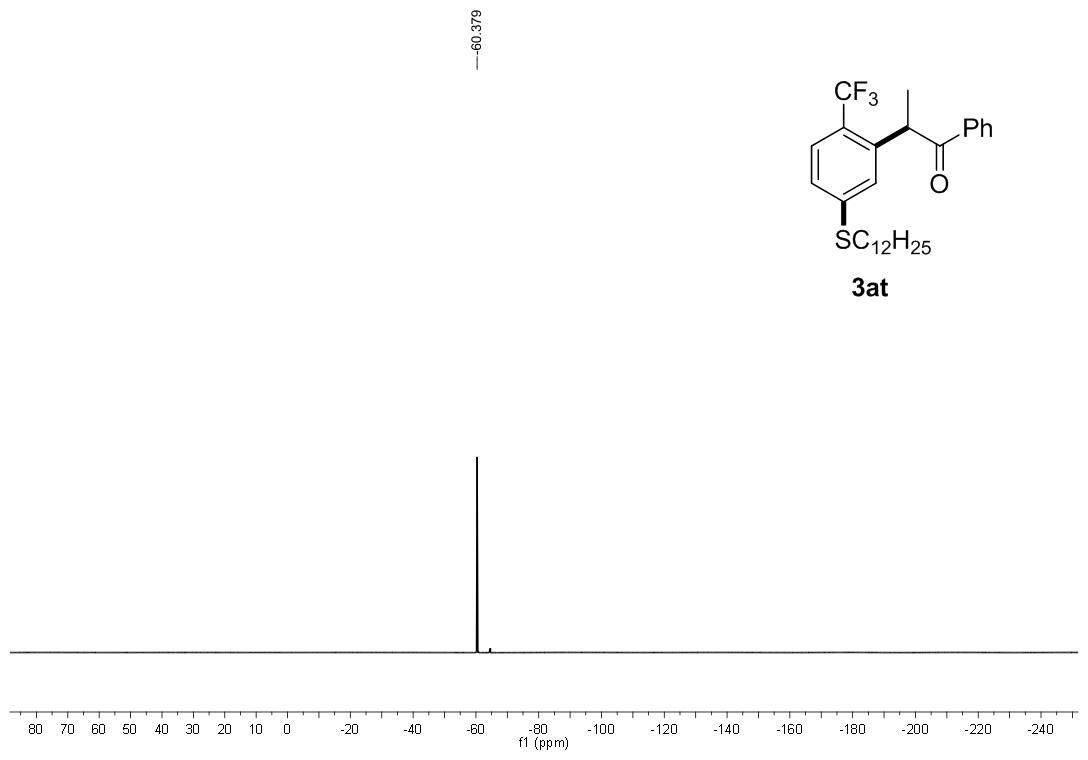


**^1^H NMR** (500 MHz, CDCl_3_) for **3au**


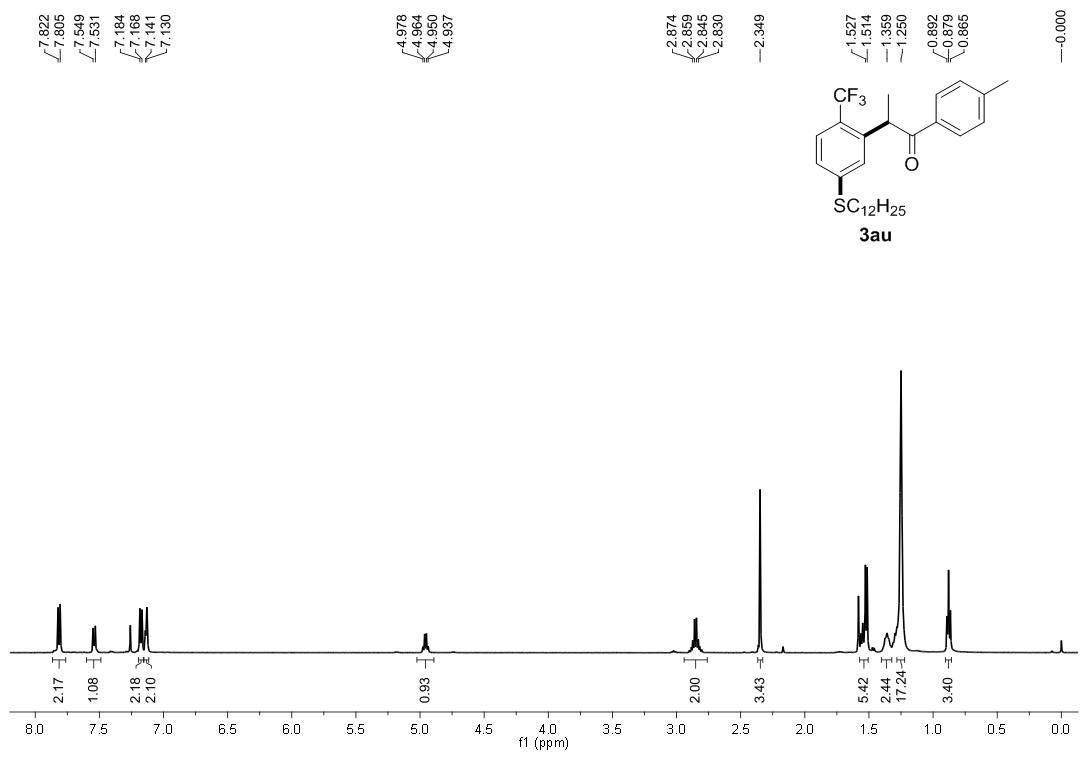


**^13^C NMR** (125 MHz, CDCl_3_) for **3au**


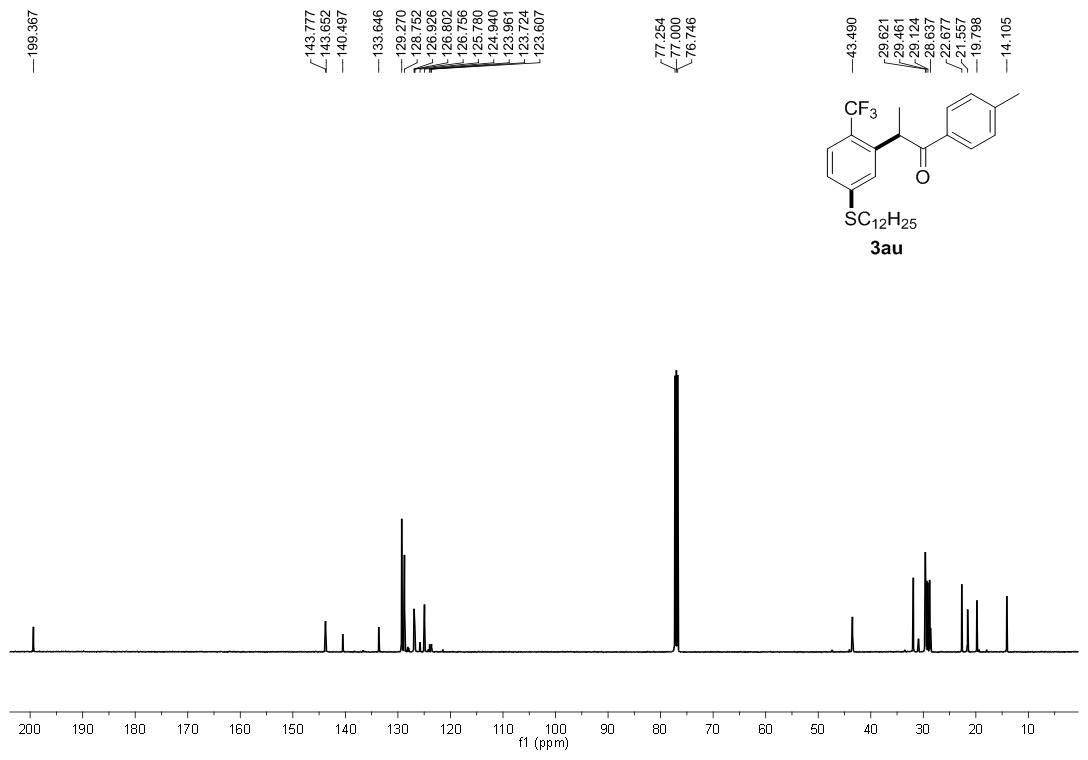


**^19^F NMR** (470 MHz, CDCl_3_) for **3au**


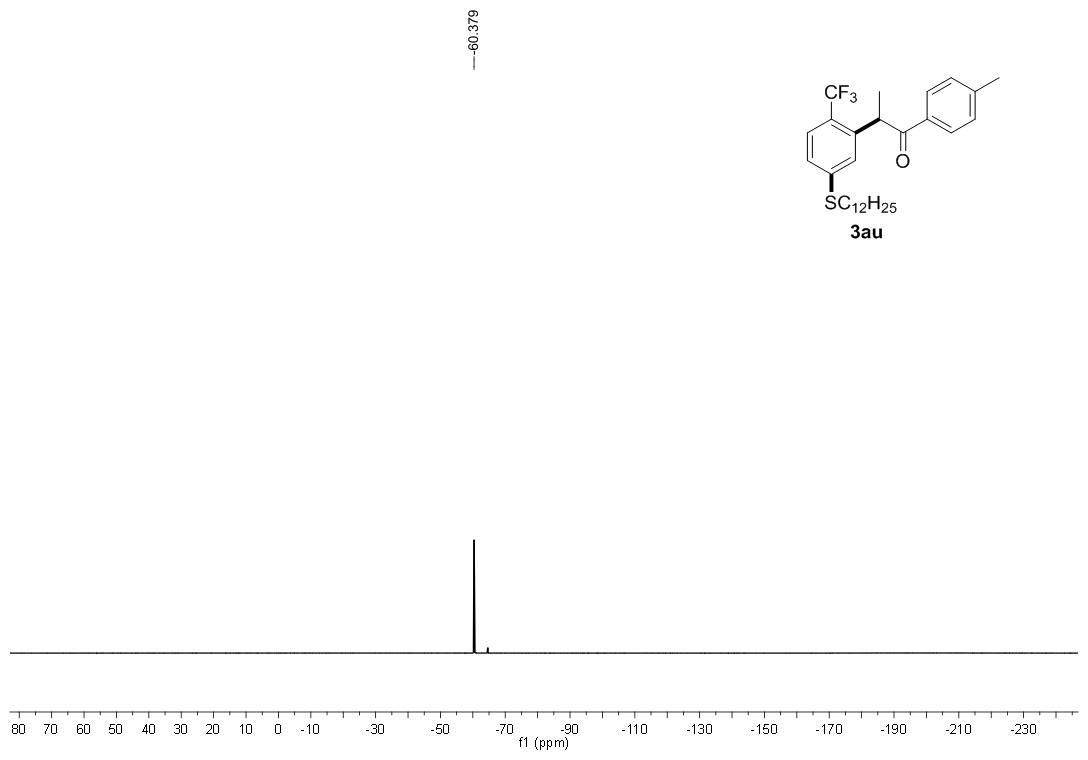


**^1^H NMR** (500 MHz, CDCl_3_) for **3av**


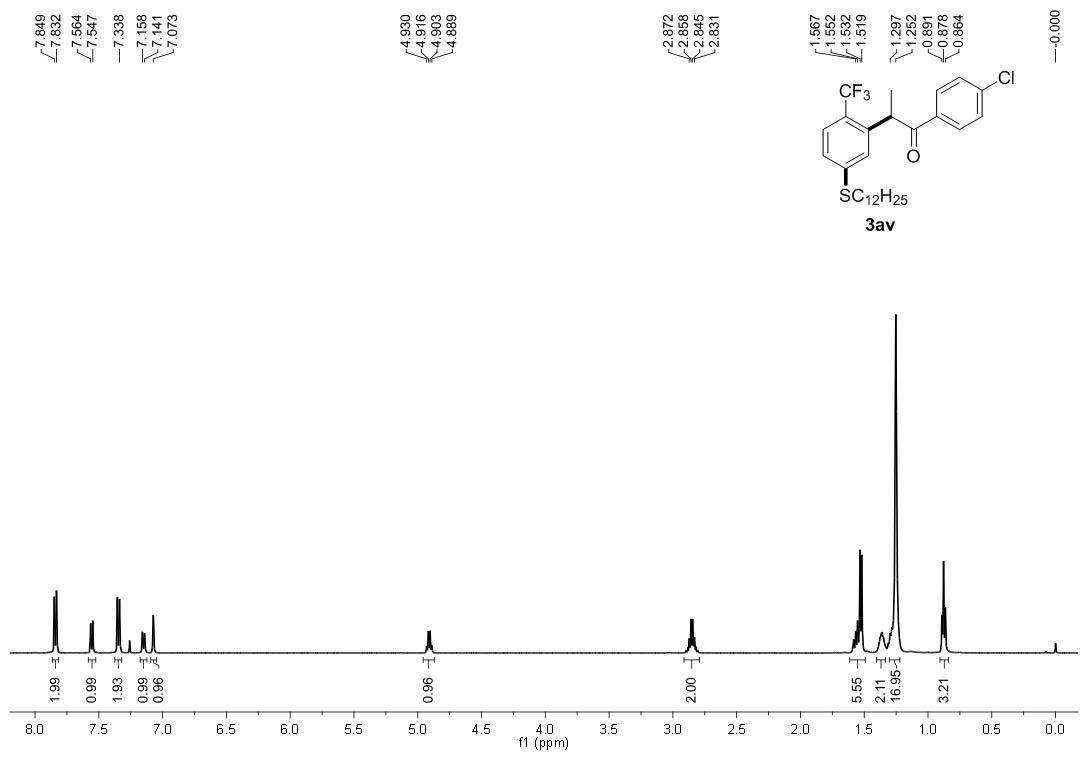


**^13^C NMR** (125 MHz, CDCl_3_) for **3av**


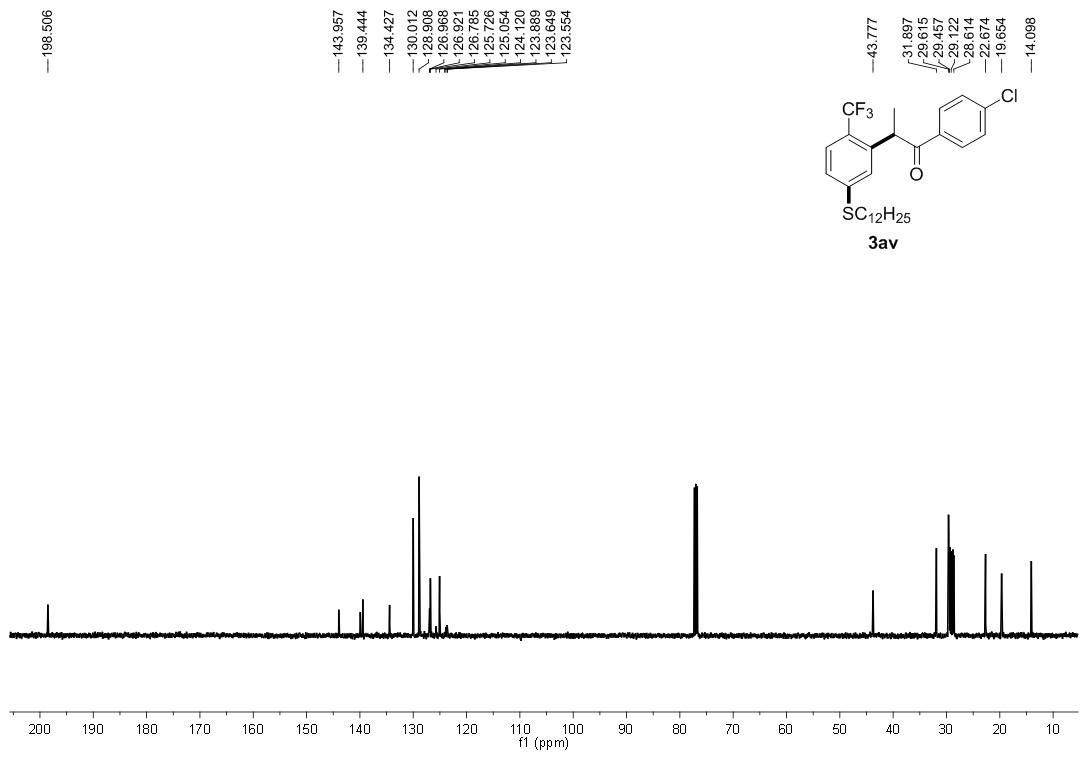


**^1^H NMR** (500 MHz, CDCl_3_) for **3aw**


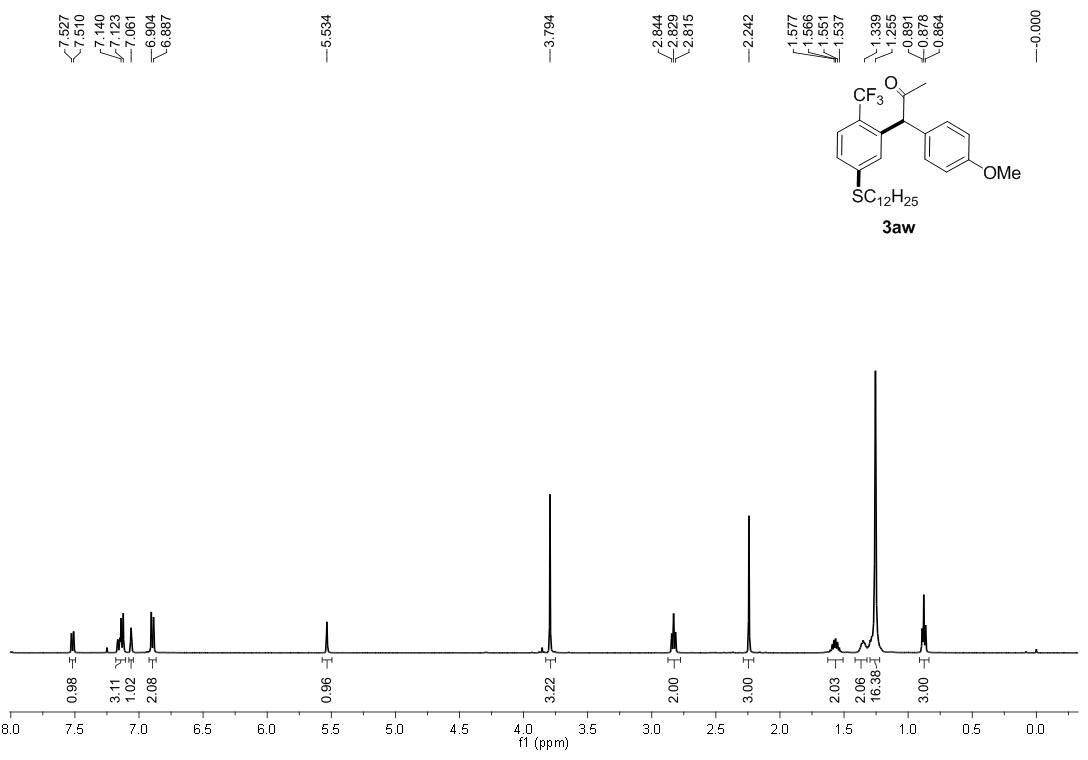


**^13^C NMR** (125 MHz, CDCl_3_) for **3aw**


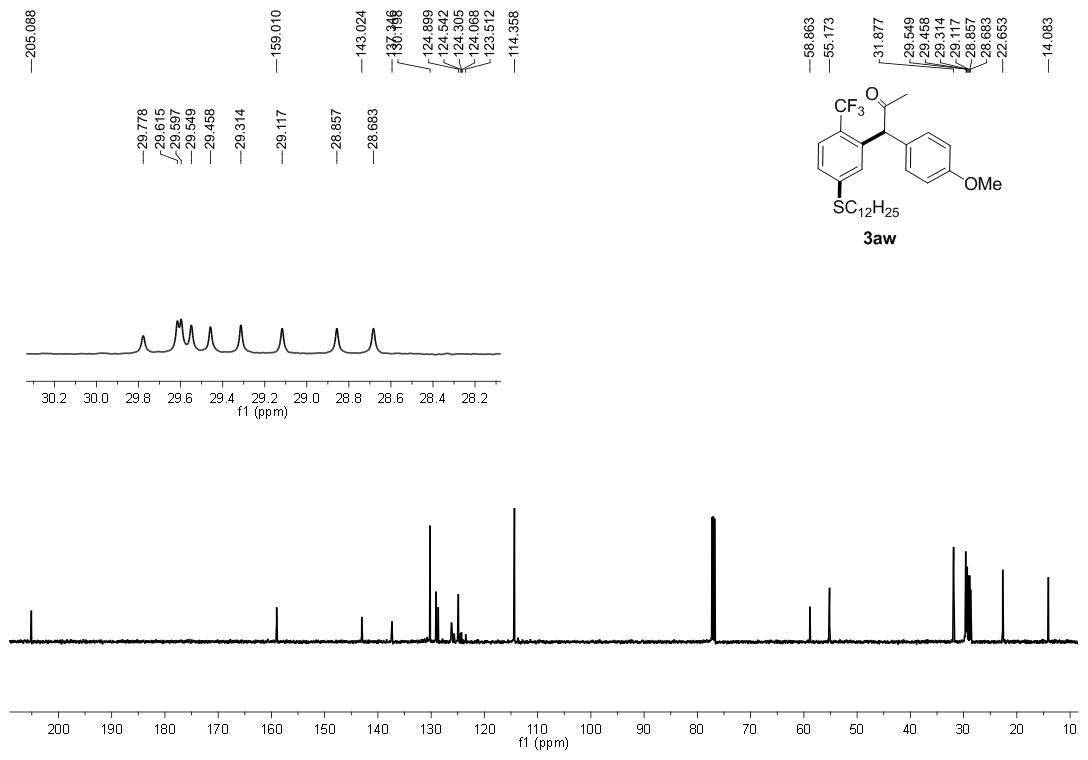


**^1^H NMR** (500 MHz, CDCl_3_) for **3ax**


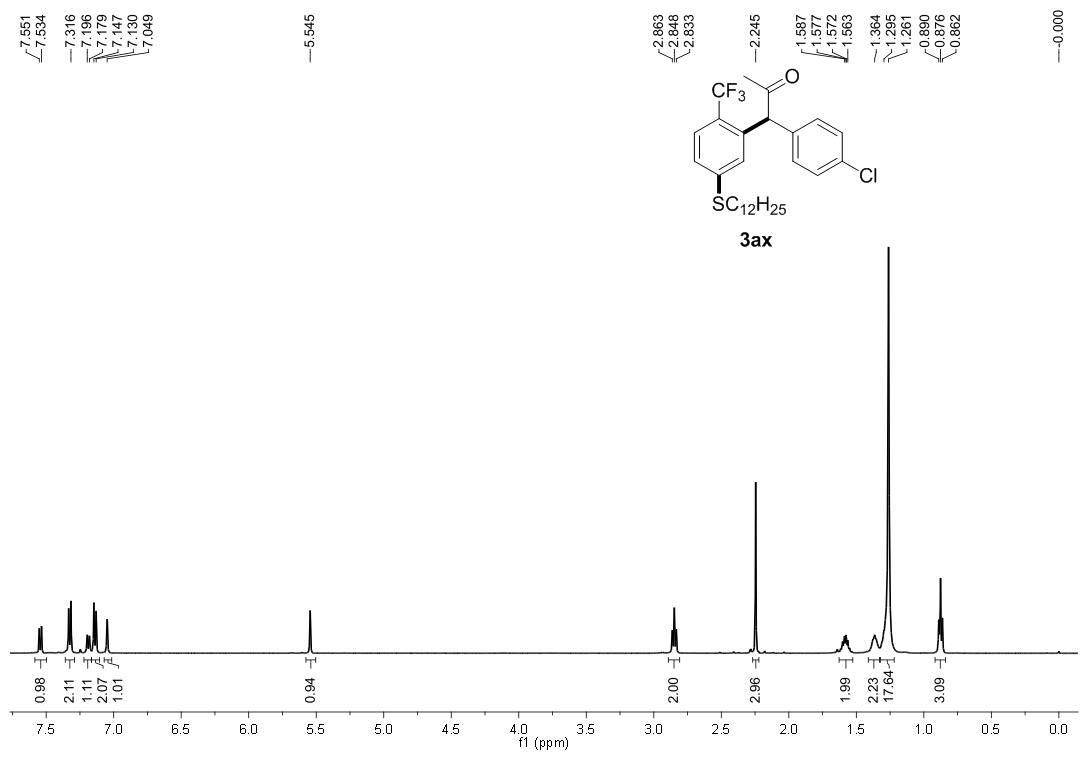


**^13^C NMR** (125 MHz, CDCl_3_) for **3ax**


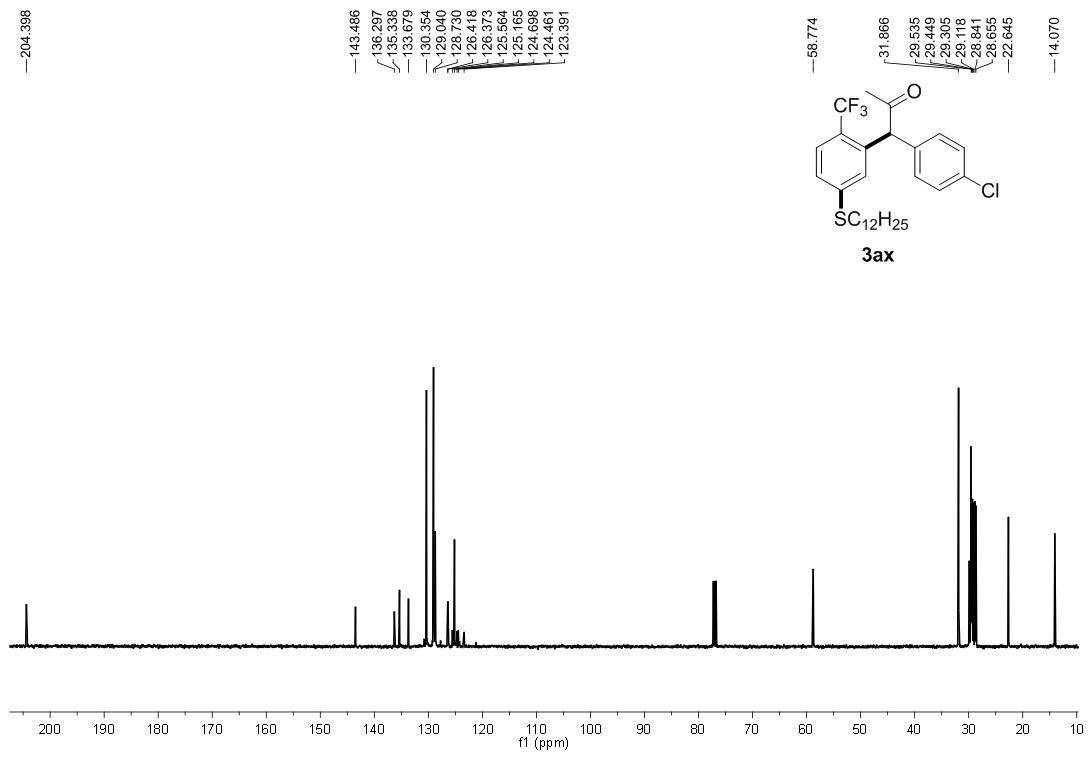


**^19^F NMR** (470 MHz, CDCl_3_) for **3ax**


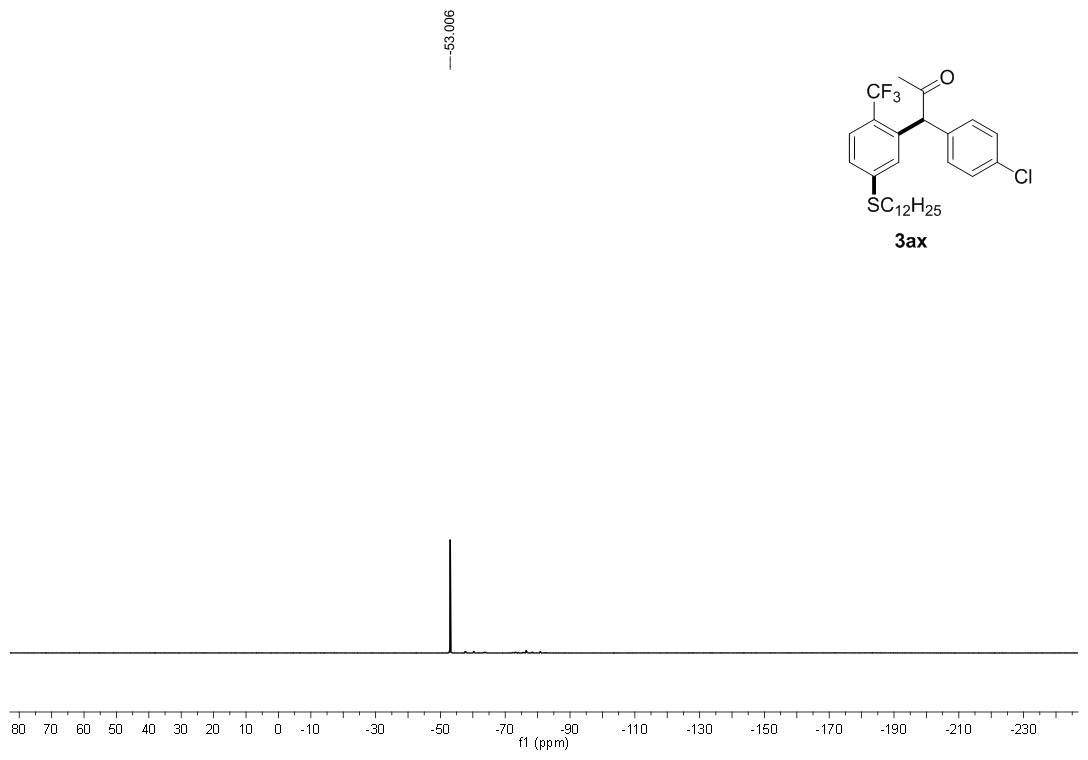


**^1^H NMR** (500 MHz, CDCl_3_) for **3ay**


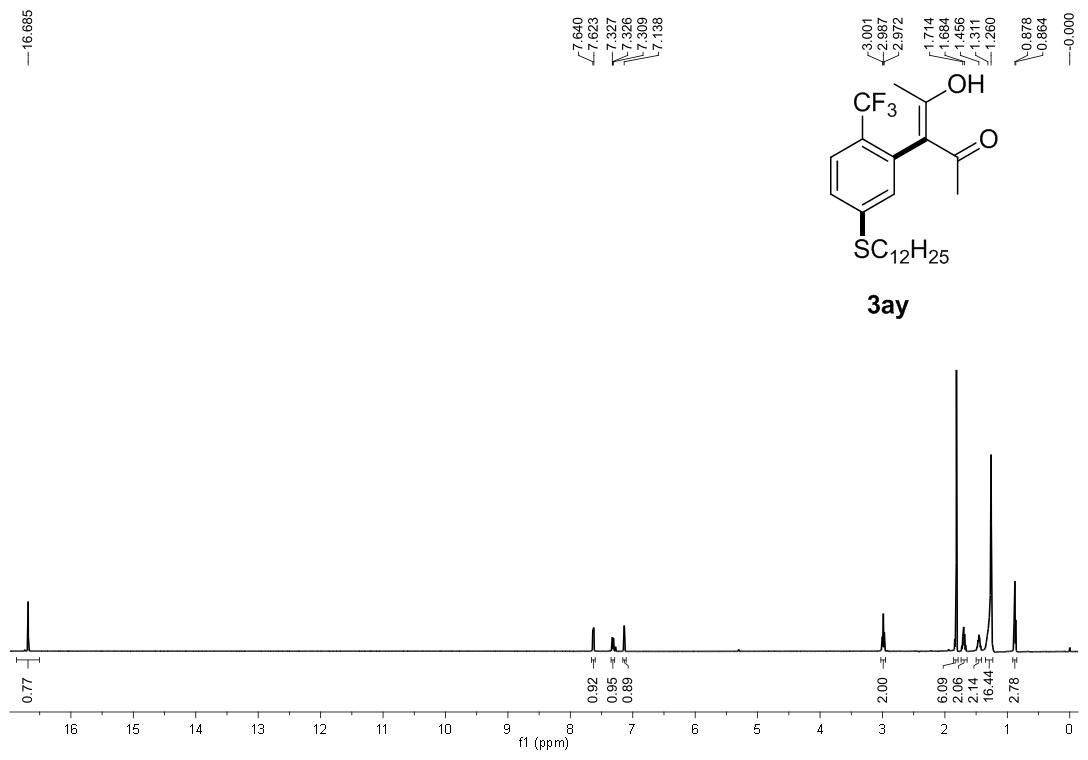


**^13^C NMR** (125 MHz, CDCl_3_) for **3ay**


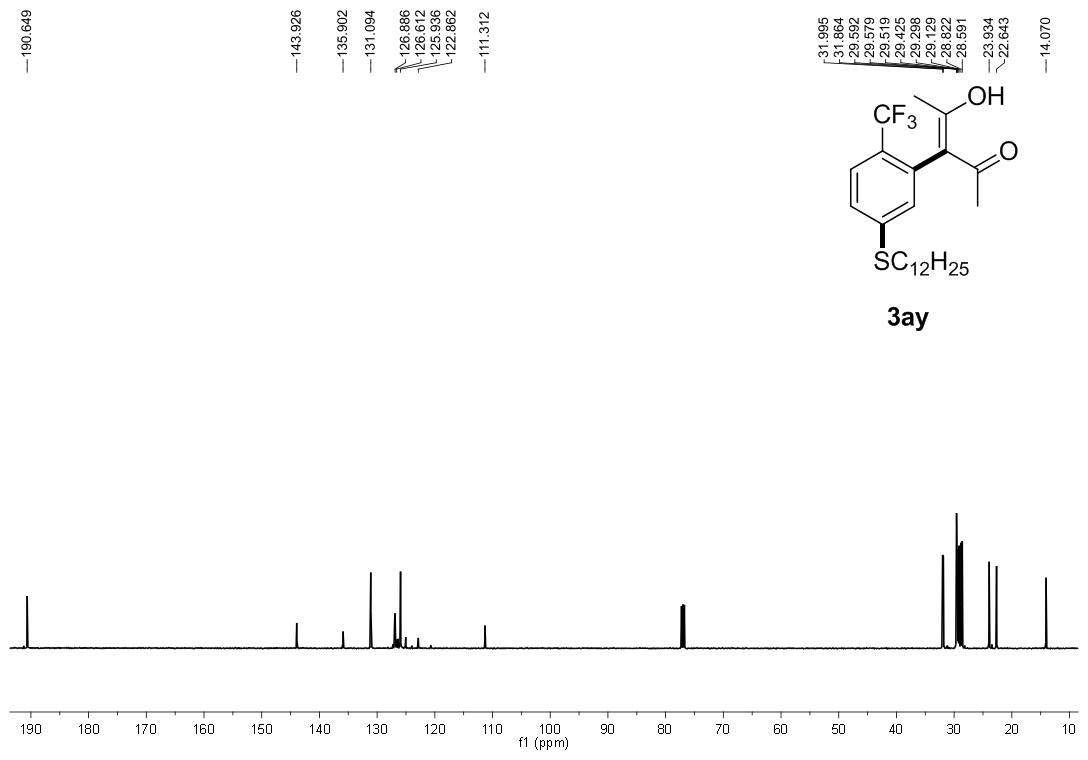


**^19^F NMR** (470 MHz, CDCl_3_) for **3ay**


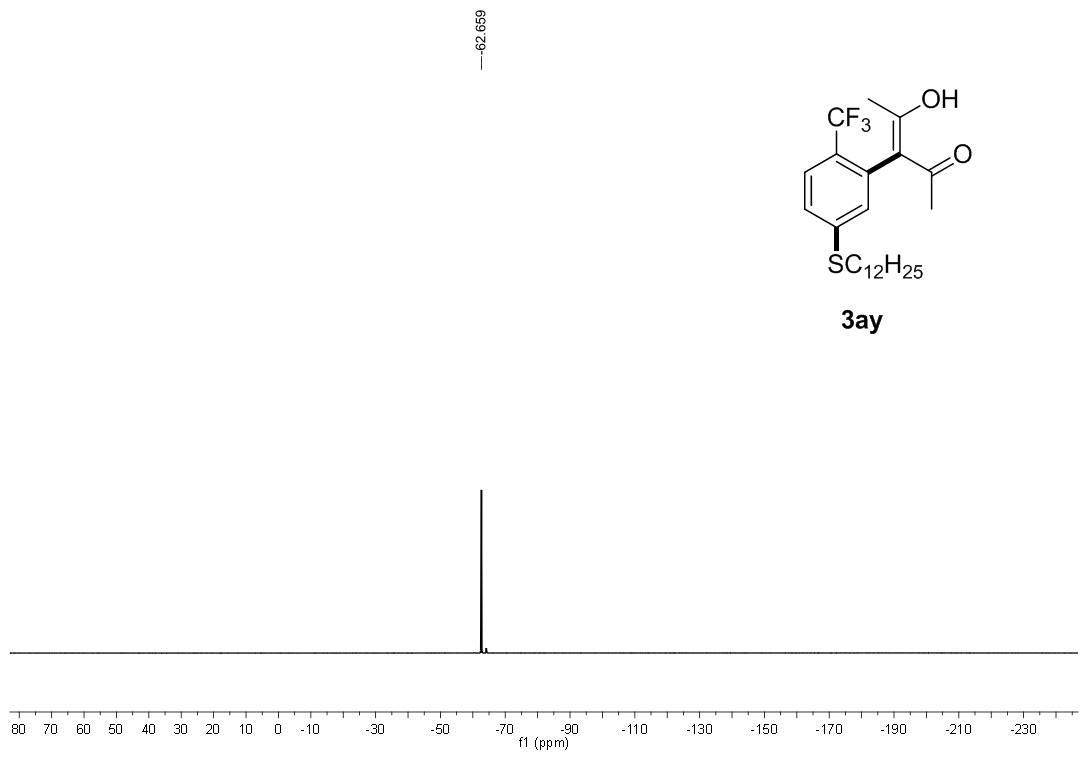


**^1^H NMR** (500 MHz, CDCl_3_) for **3az**


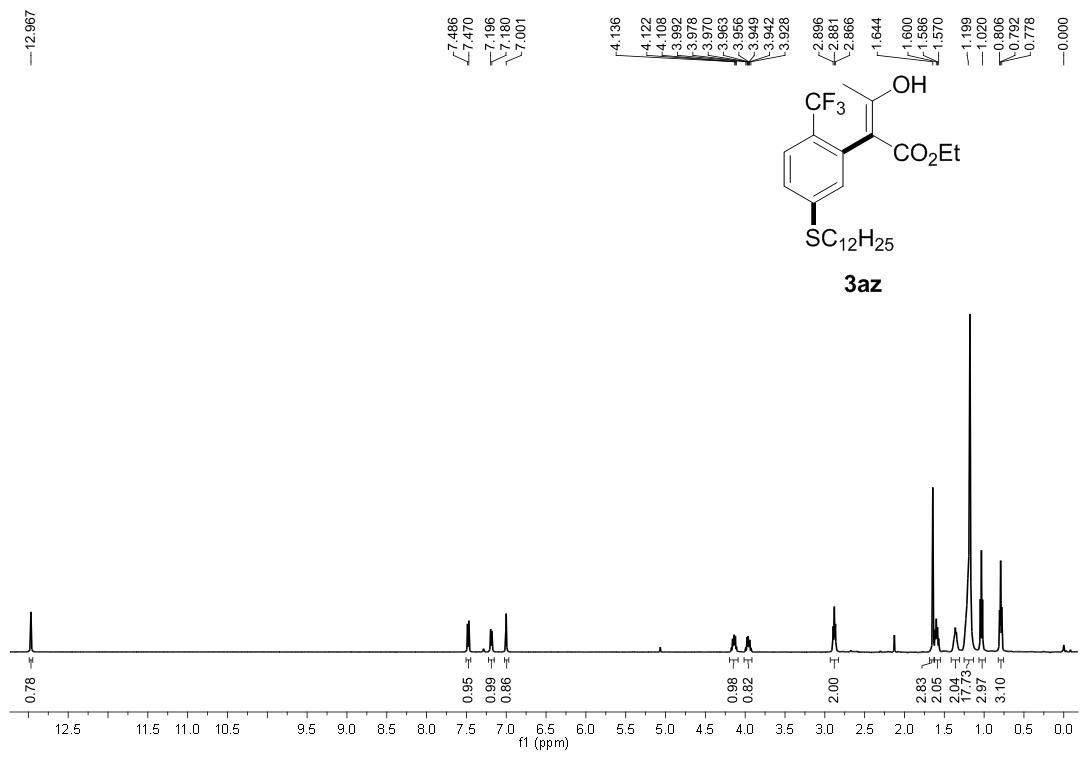


**^13^C NMR** (125 MHz, CDCl_3_) for **3az**


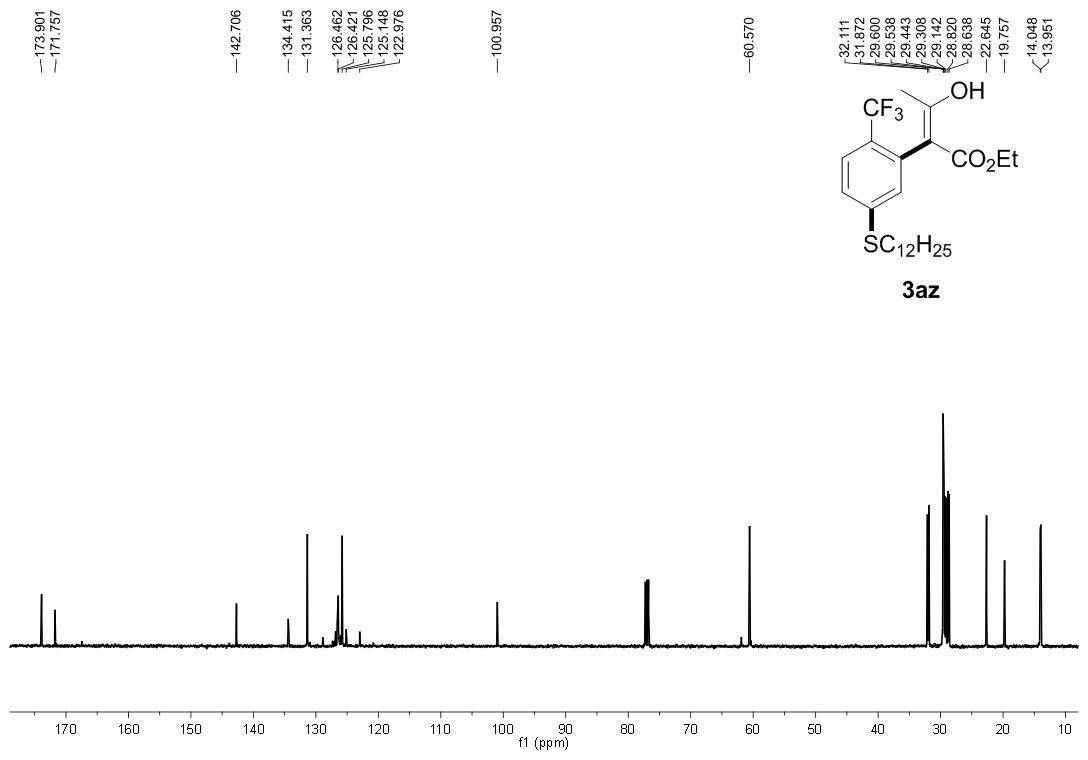


**^19^F NMR** (470 MHz, CDCl_3_) for **3az**


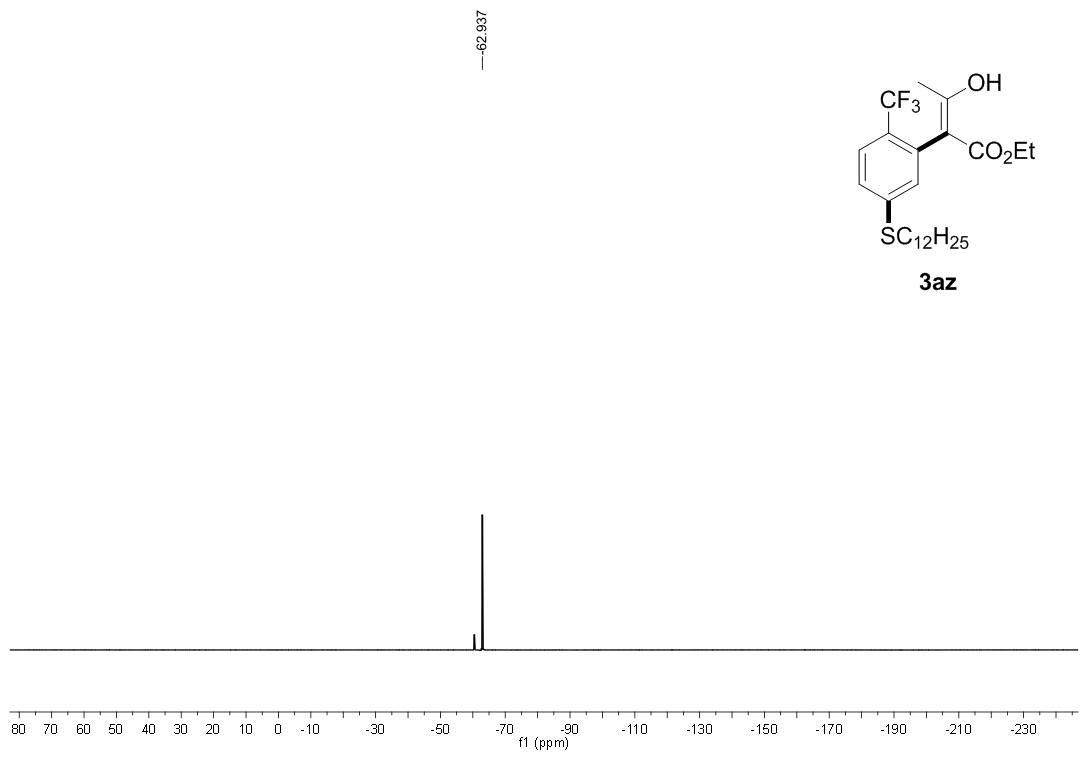


**^1^H NMR** (500 MHz, CDCl_3_) for **3aa1**

**
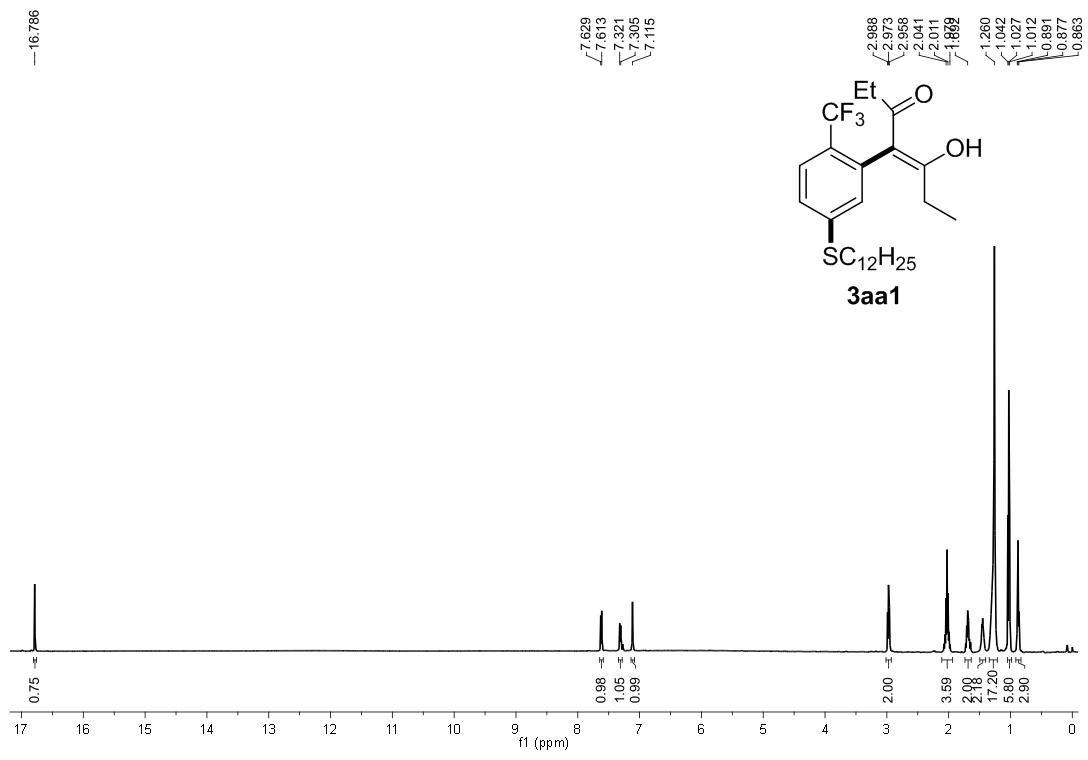
**

**^13^C NMR** (125 MHz, CDCl_3_) for **3aa1**

**
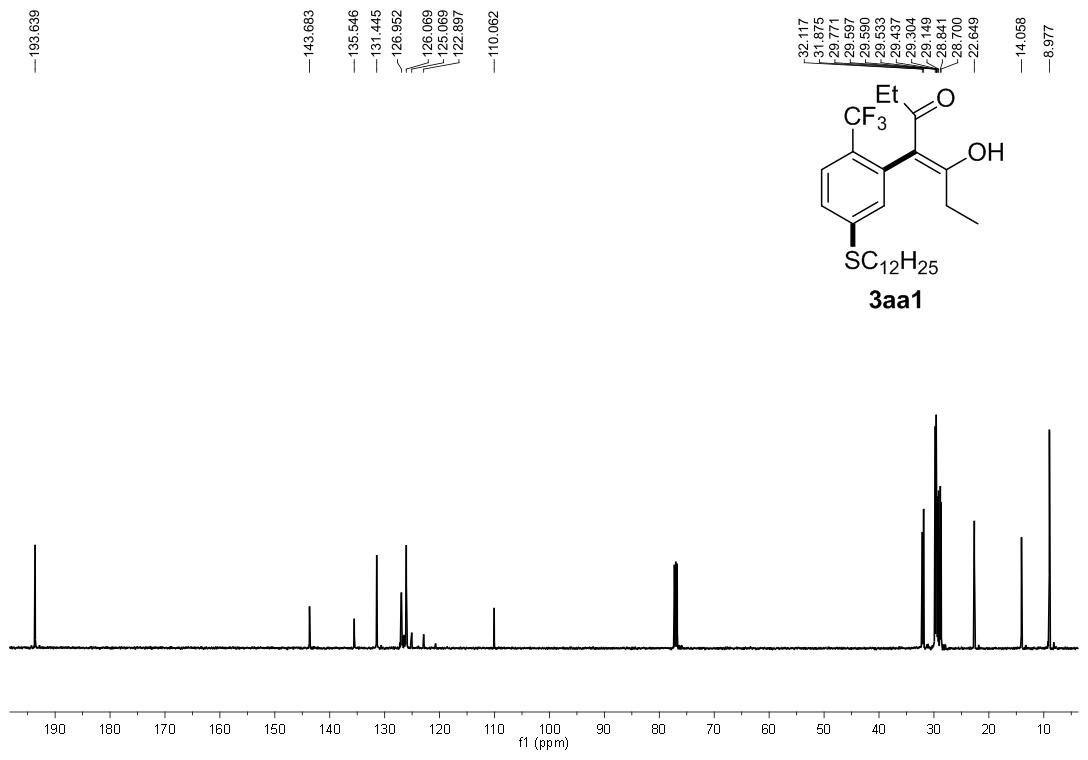
**

**^1^H NMR** (500 MHz, CDCl_3_) for **3aa2**

**
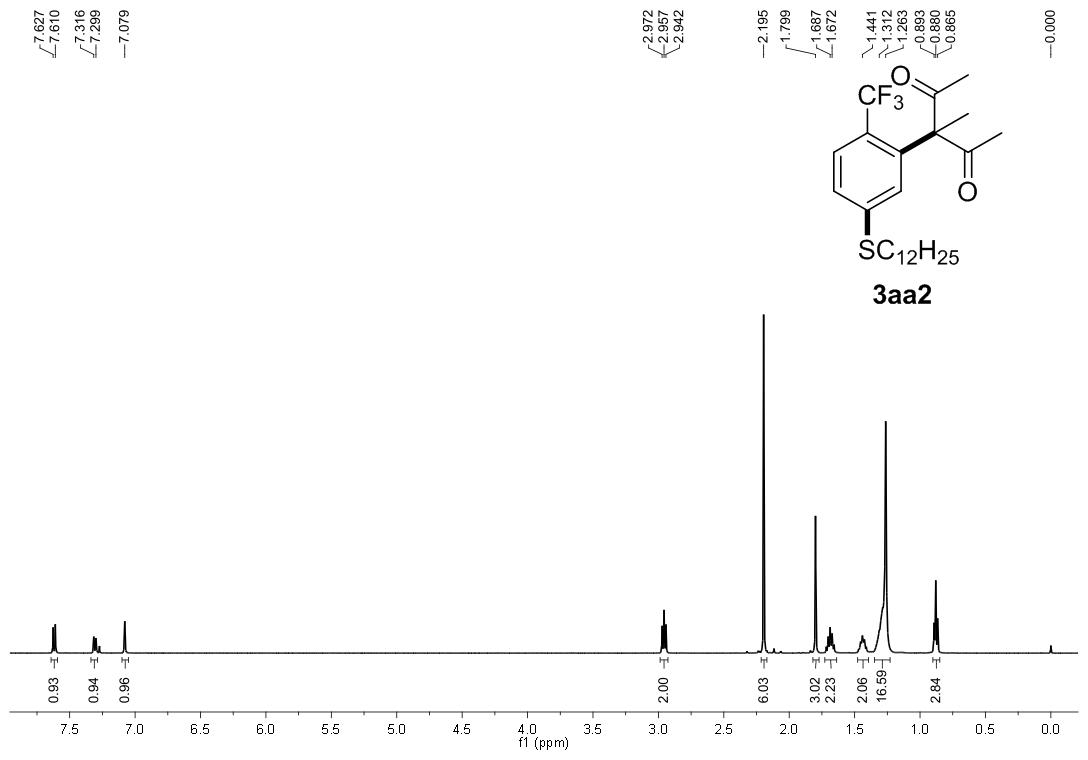
**

**^13^C NMR** (125 MHz, CDCl_3_) for **3aa2**

**
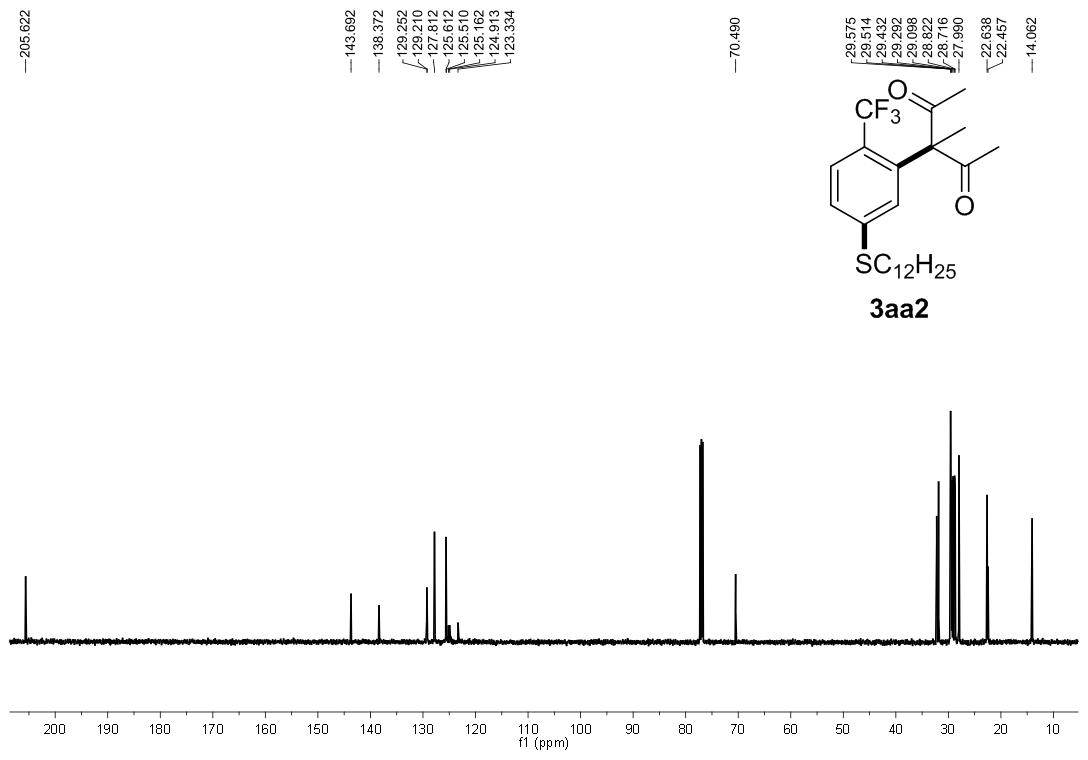
**

**^19^F NMR** (470 MHz, CDCl_3_) for **3aa2**

**
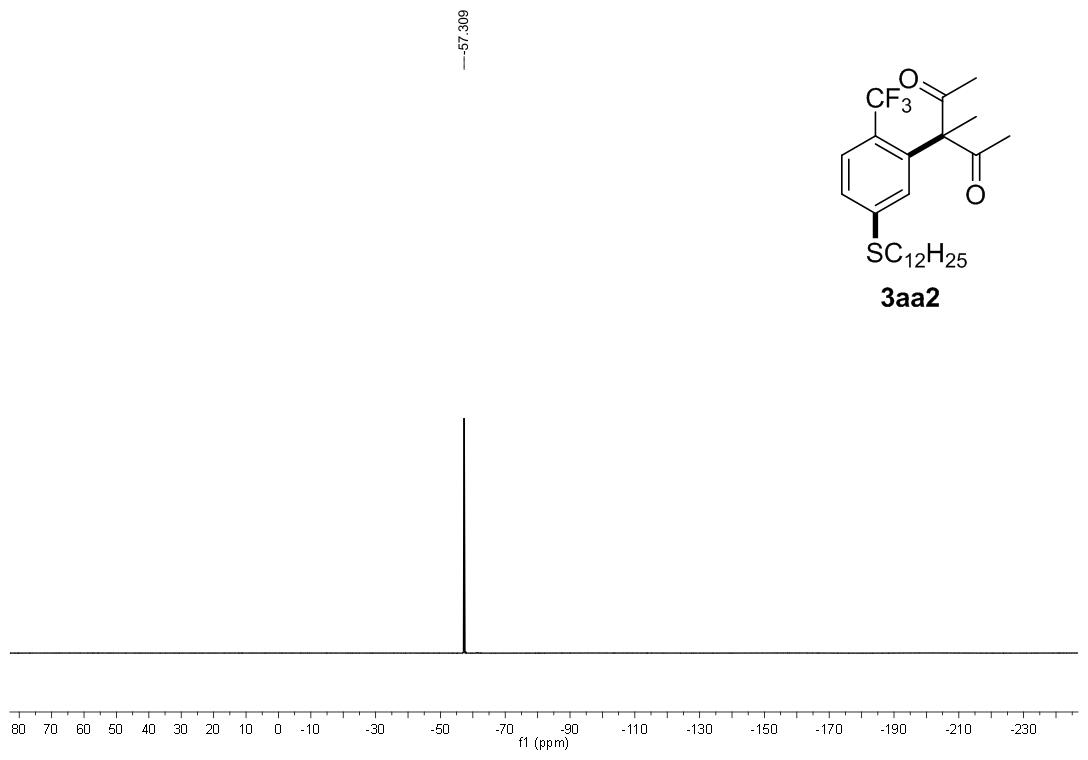
**

**^1^H NMR** (500 MHz, CDCl_3_) for **3’a**

**
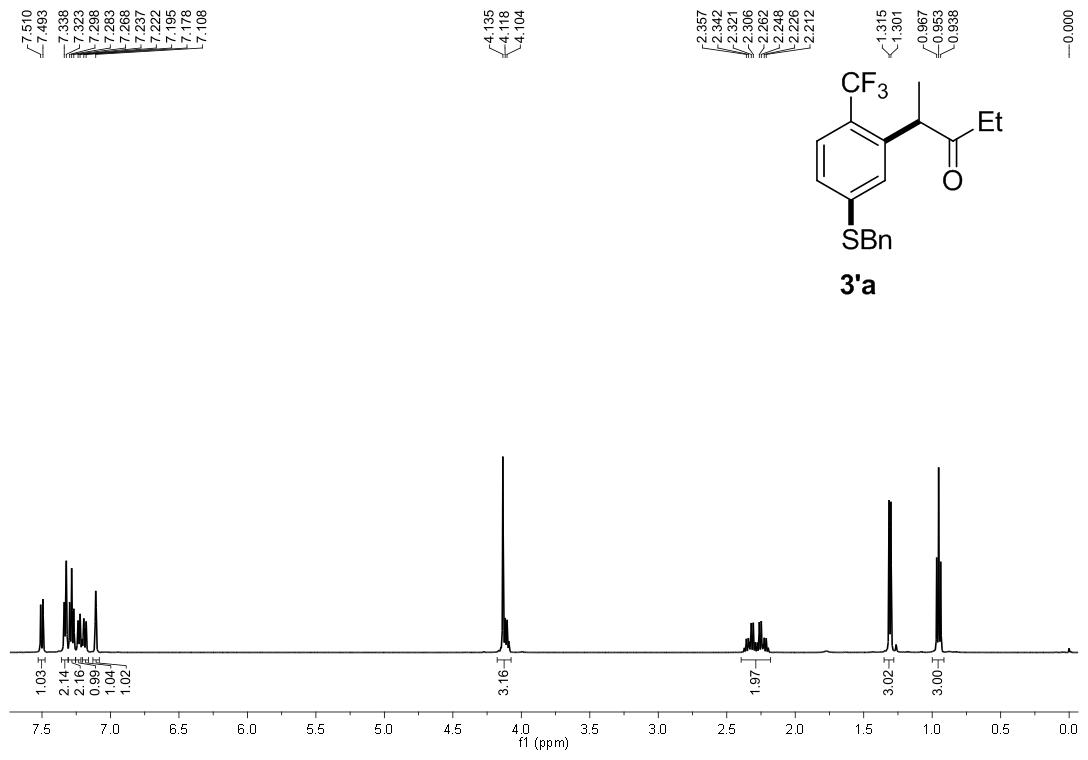
**

**^13^C NMR** (125 MHz, CDCl_3_) for **3’a**

**
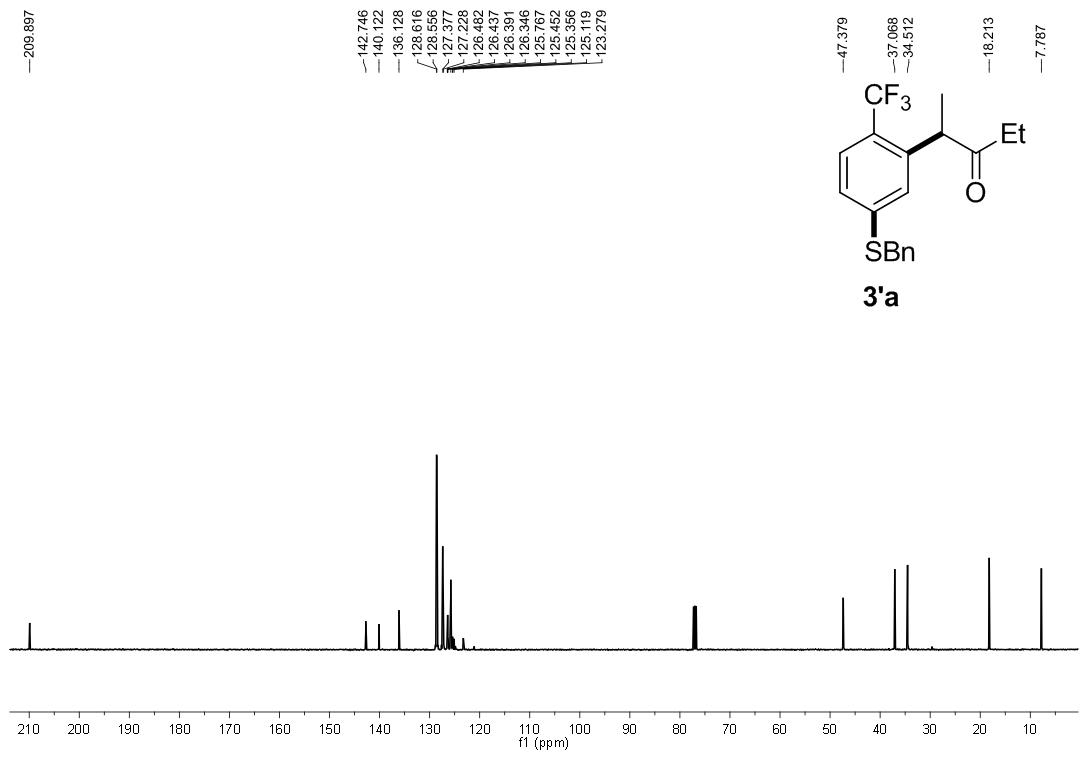
**

**^19^F NMR** (470 MHz, CDCl_3_) for **3’a**

**
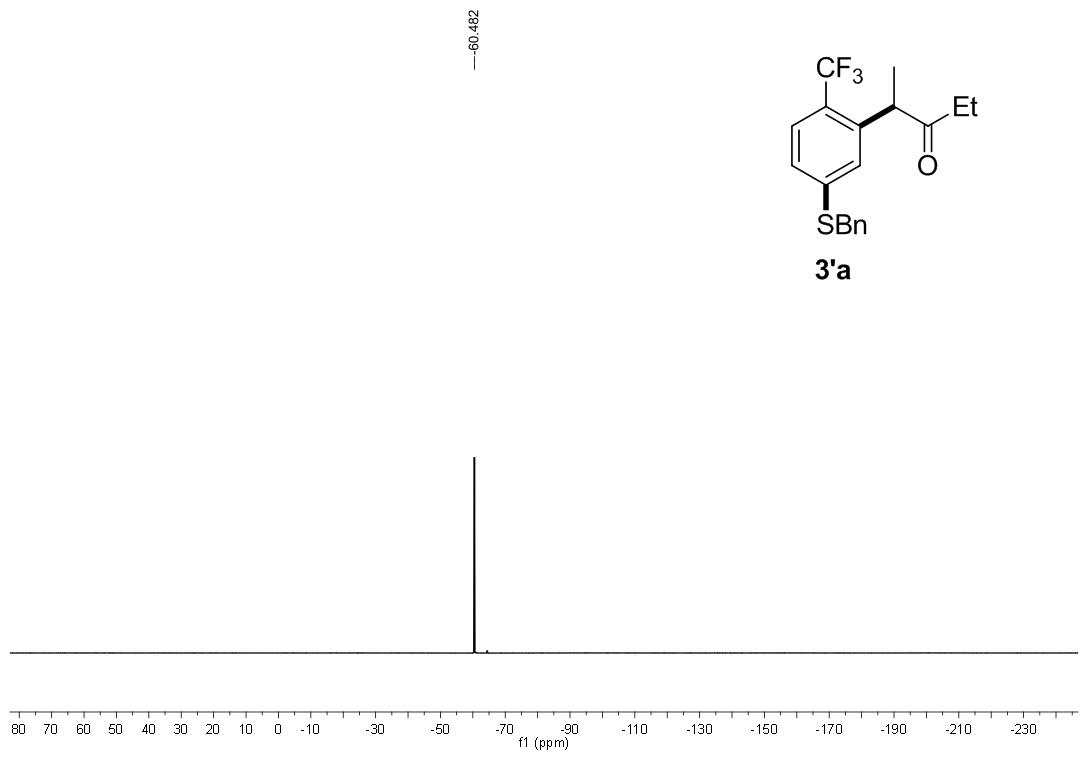
**

**^1^H NMR** (500 MHz, CDCl_3_) for **3’b**

**
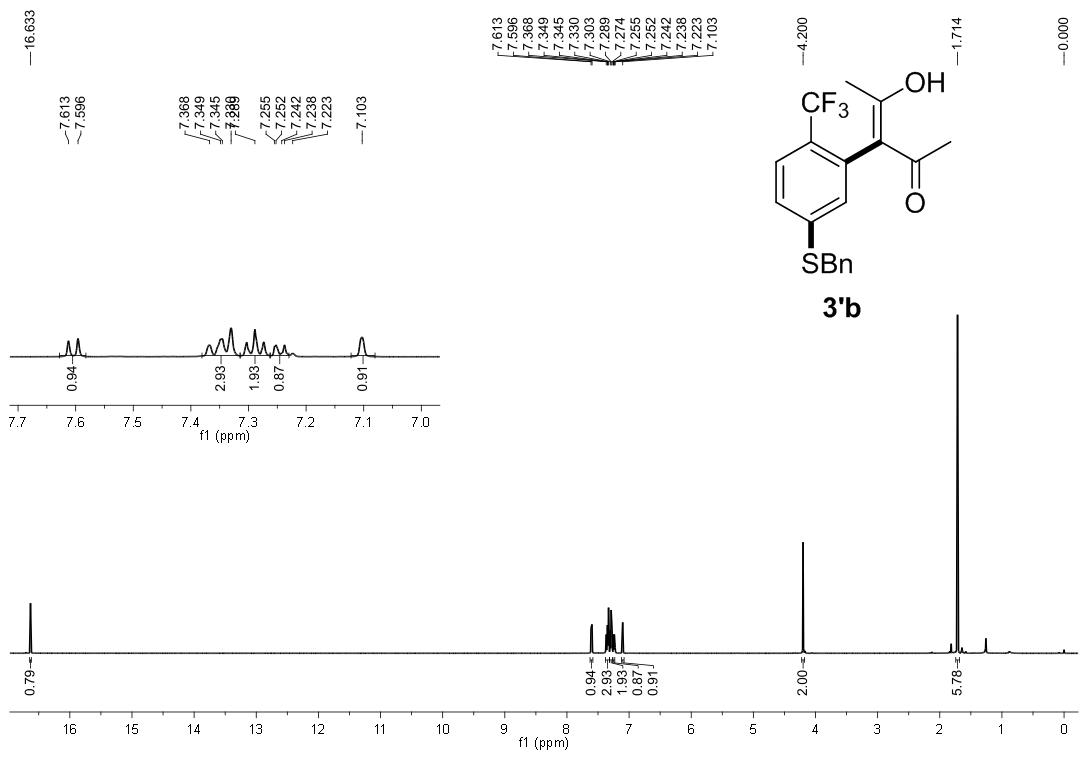
**

**^13^C NMR** (125 MHz, CDCl_3_) for **3’b**

**
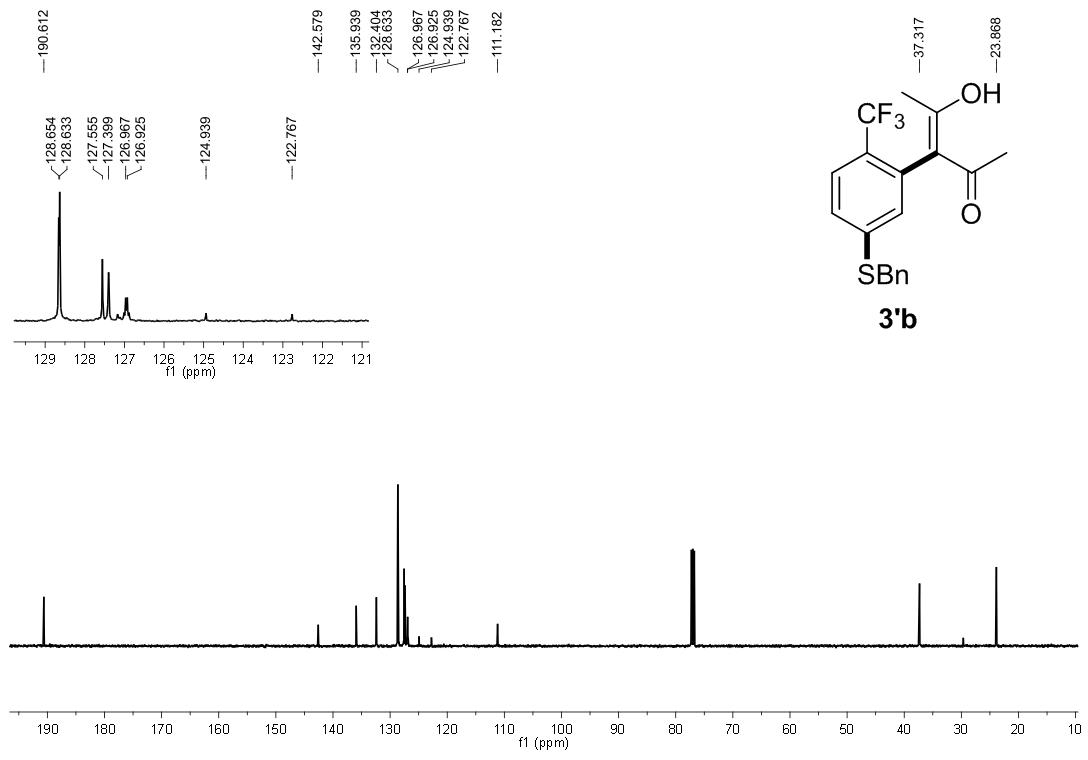
**

**^19^F NMR** (470 MHz, CDCl_3_) for **3’b**

**
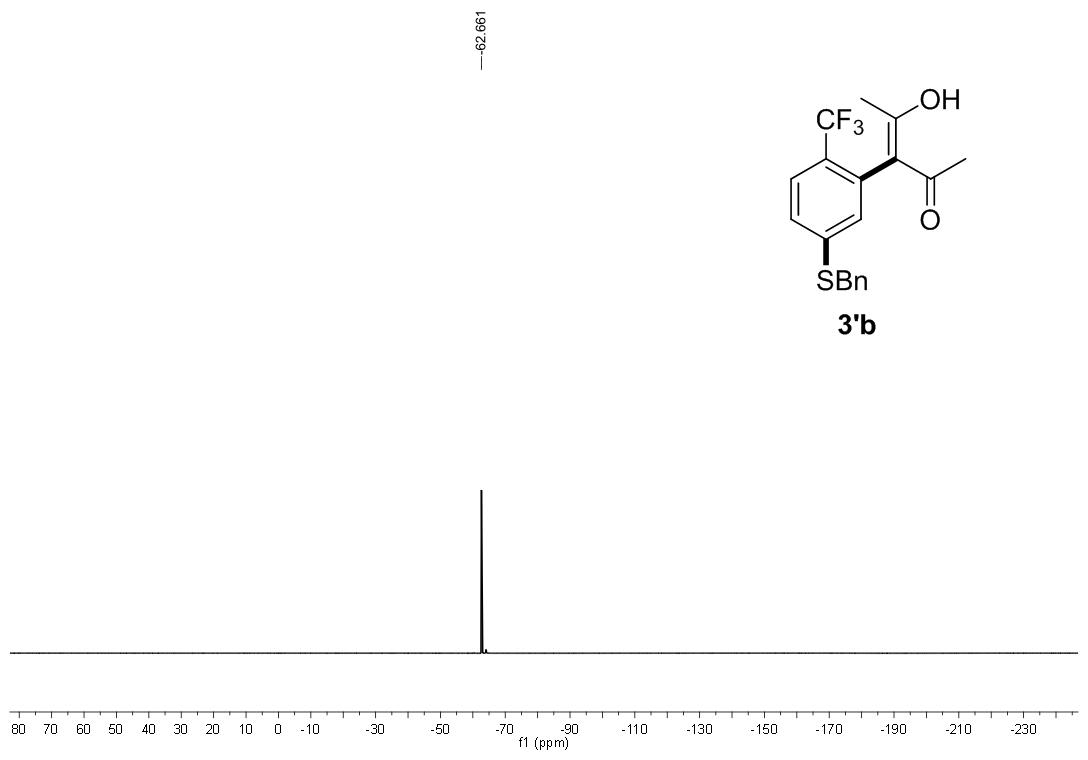
**

**^1^H NMR** (500 MHz, CDCl_3_) for **3ba**

**
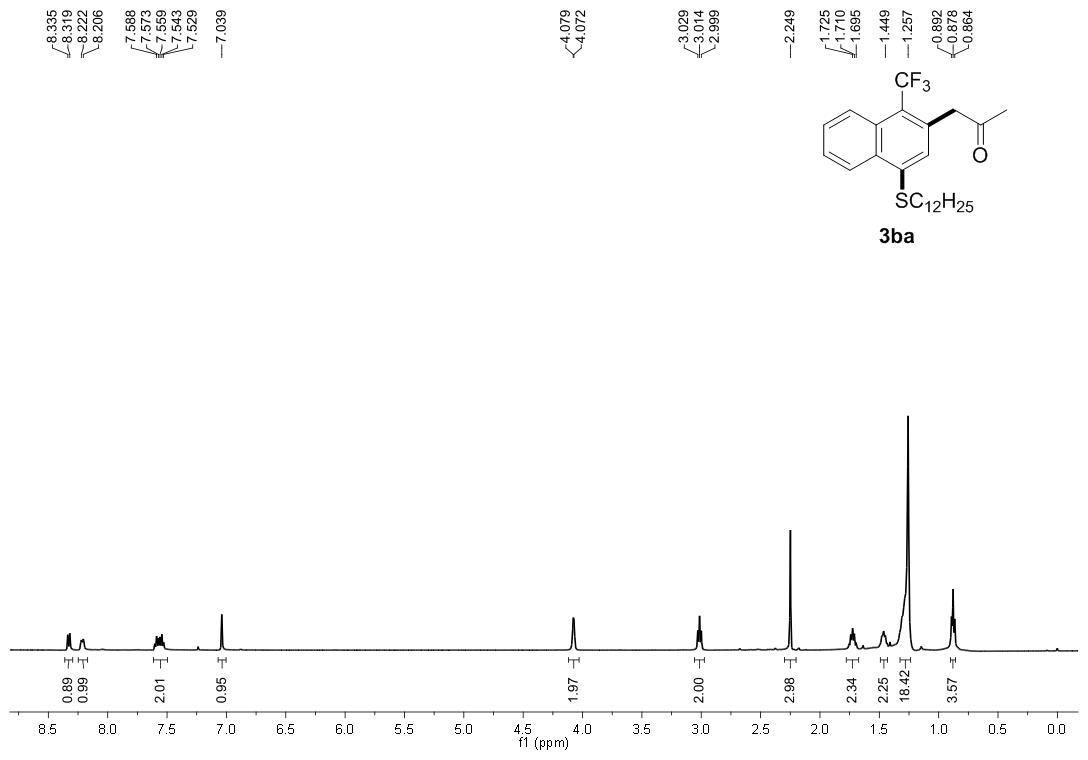
**

**^13^C NMR** (125 MHz, CDCl_3_) for **3ba**

**
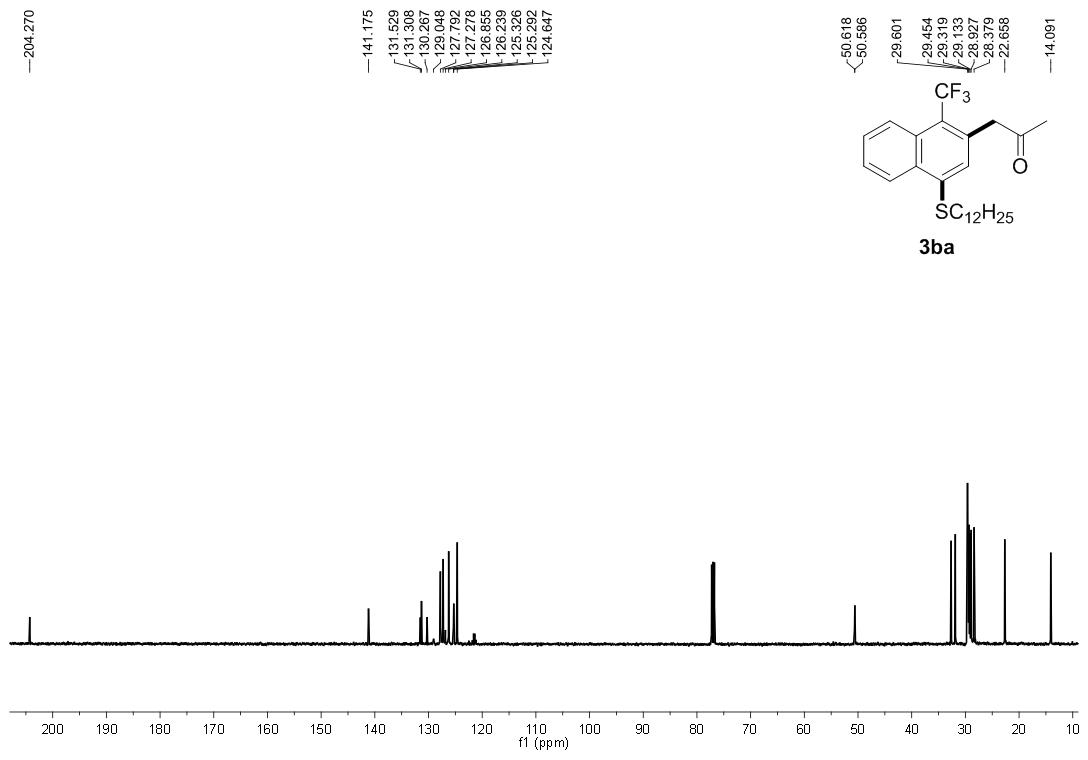
**

**^19^F NMR** (470 MHz, CDCl_3_) for **3ba**

**
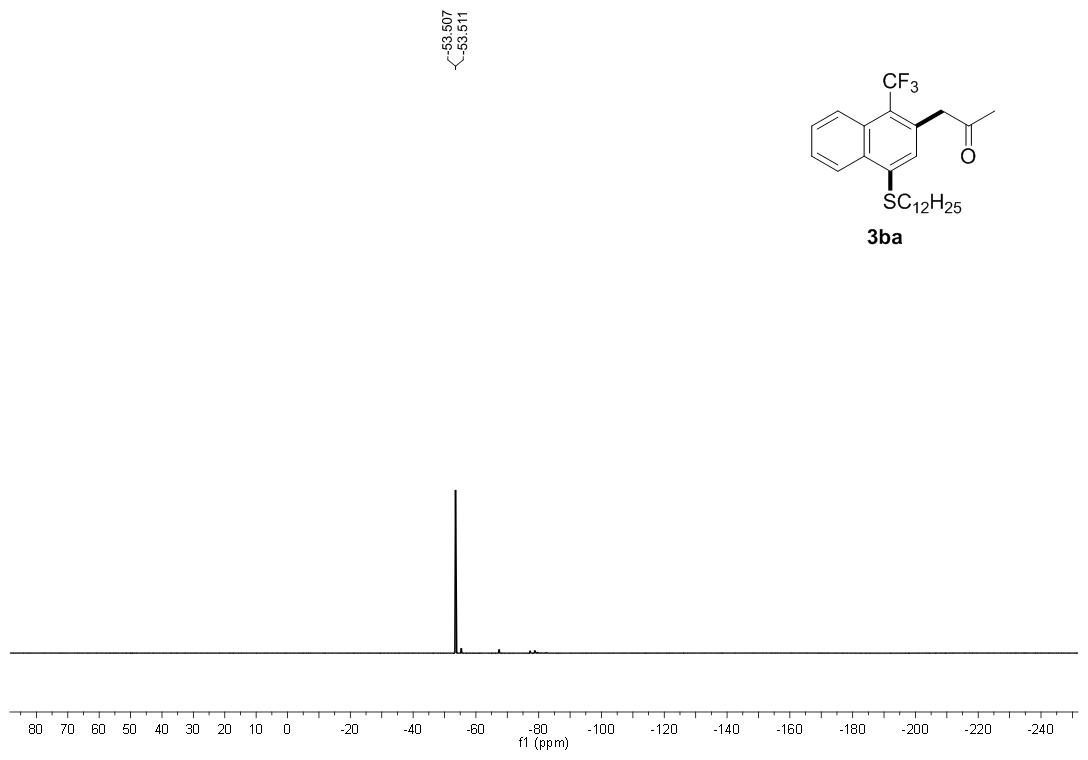
**

**^1^H NMR** (500 MHz, CDCl_3_) for **3bc**

**
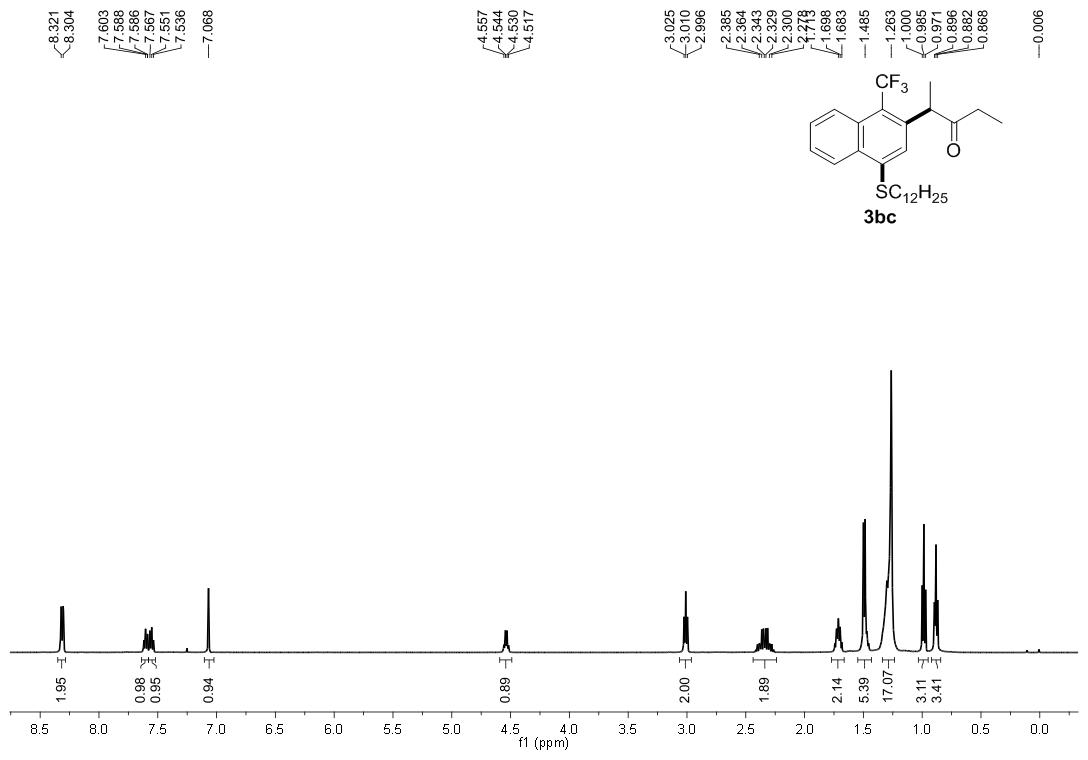
**

**^13^C NMR** (125 MHz, CDCl_3_) for **3bc**

**
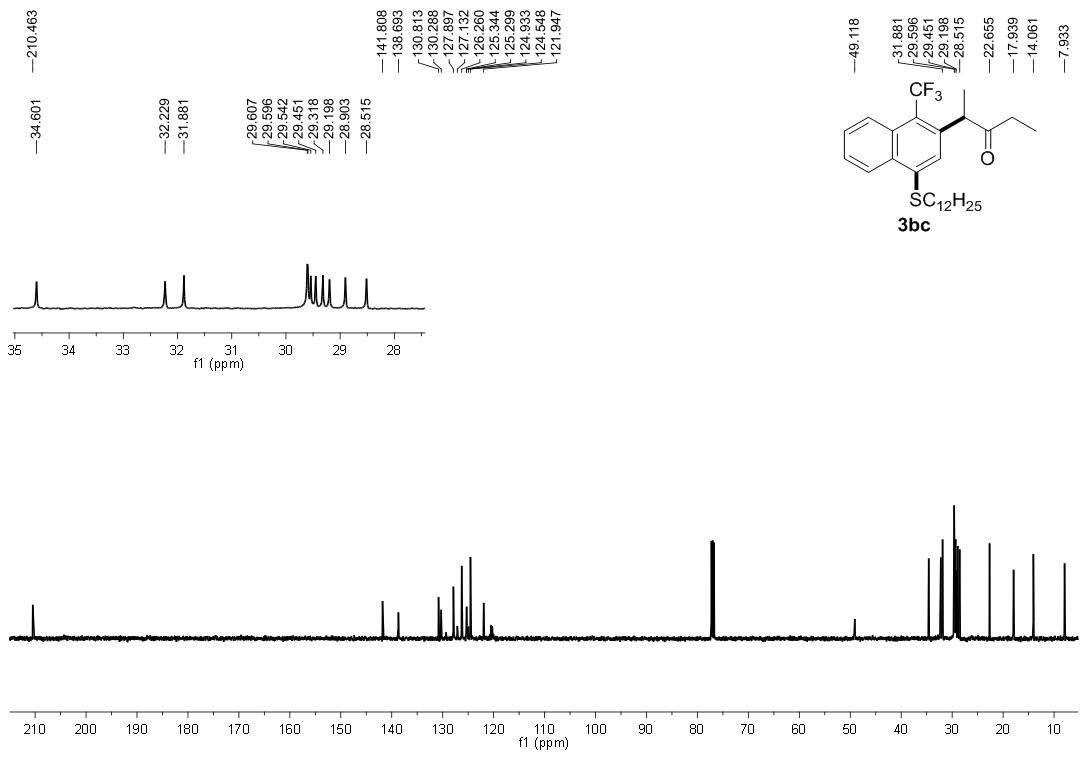
**

**^1^H NMR** (500 MHz, CDCl_3_) for **3cc**

**
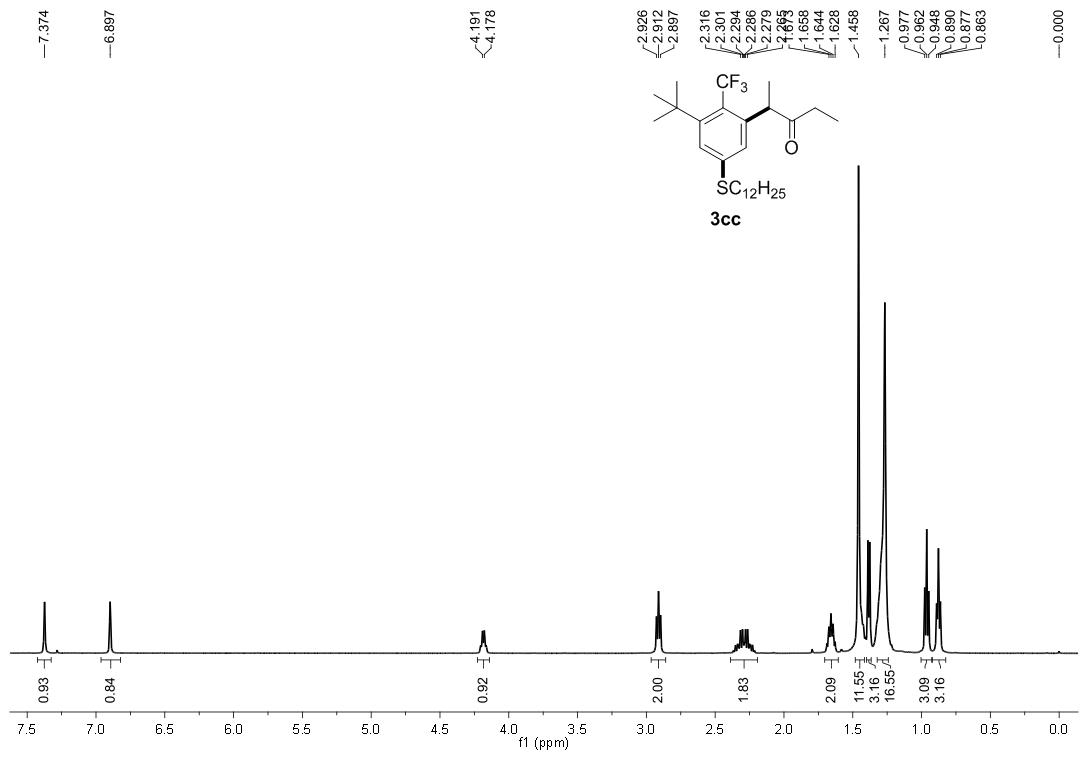
**

**^13^C NMR** (125 MHz, CDCl_3_) for **3cc**

**
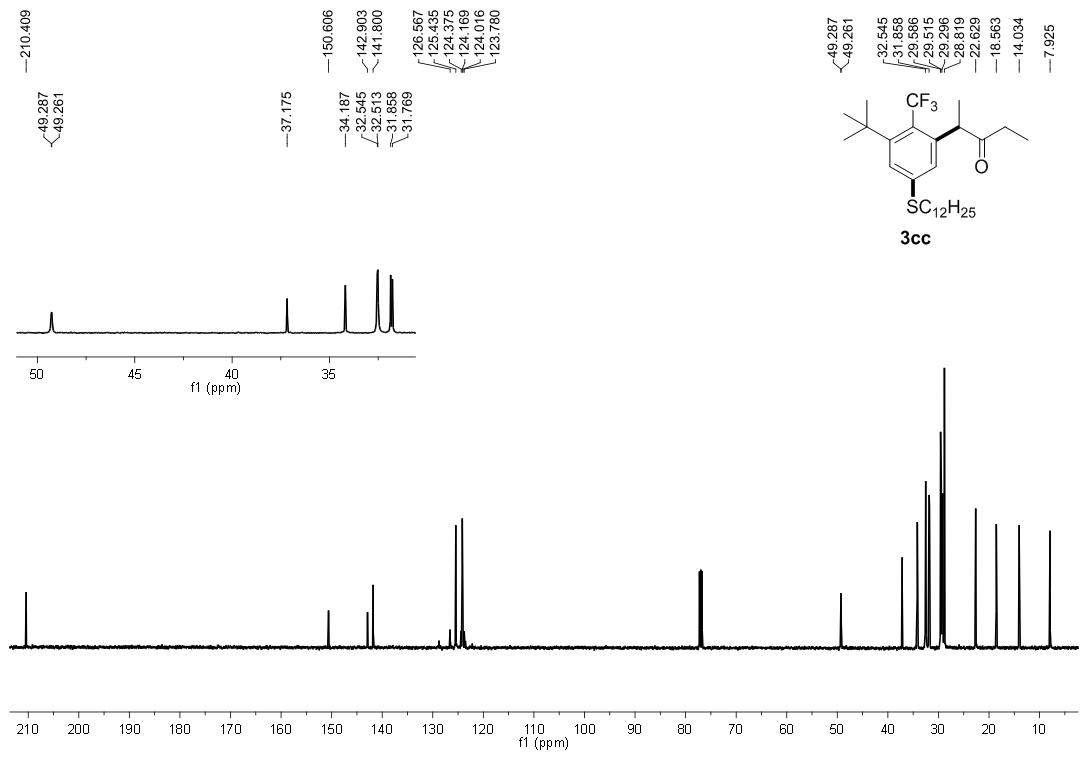
**

**^19^F NMR** (470 MHz, CDCl_3_) for **3cc**

**
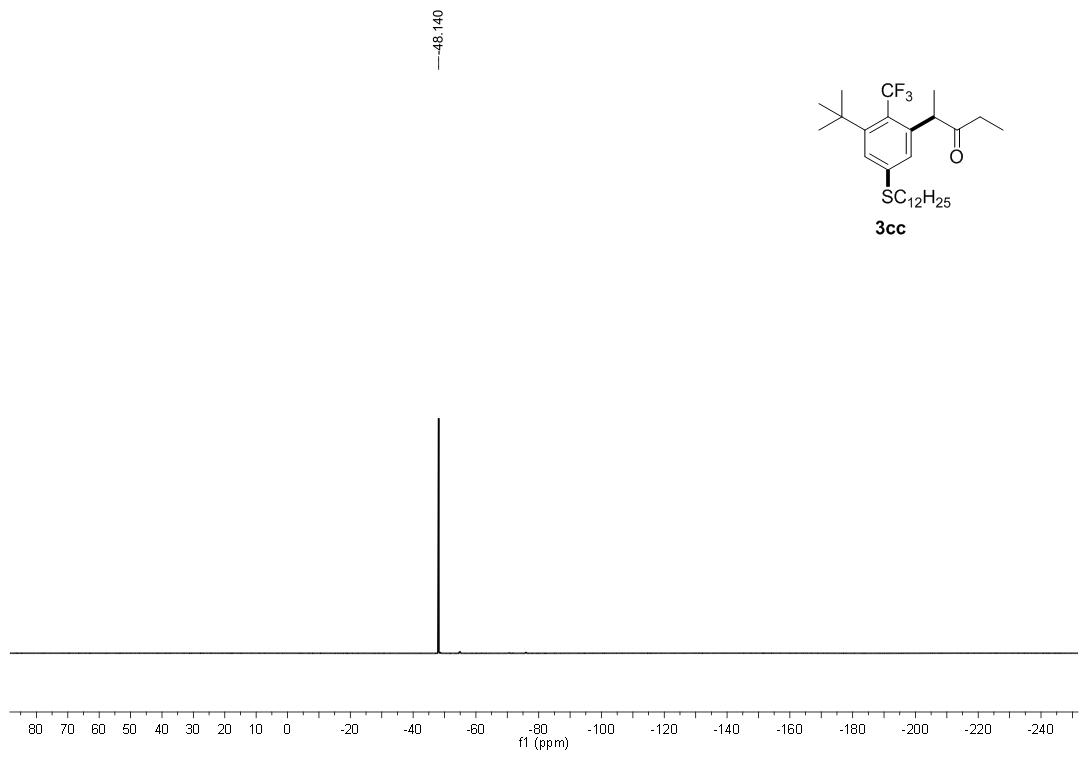
**

**^1^H NMR** (500 MHz, CDCl_3_) for **3dc**

**
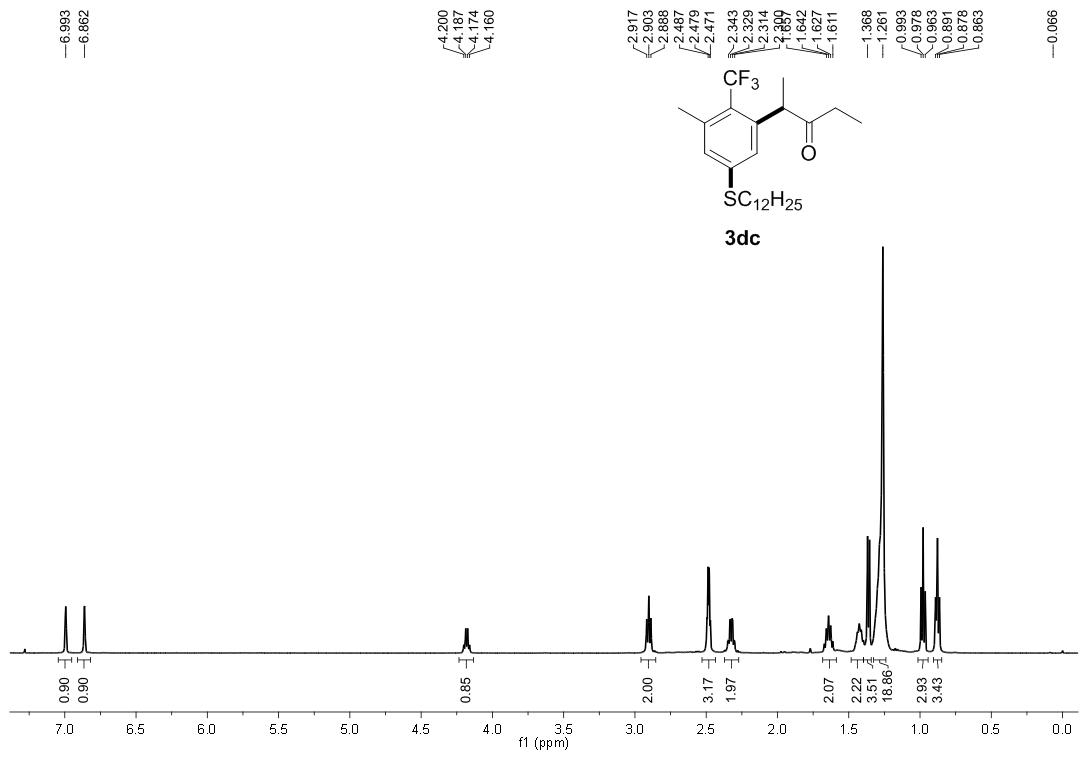
**

**^13^C NMR** (125 MHz, CDCl_3_) for **3dc**

**
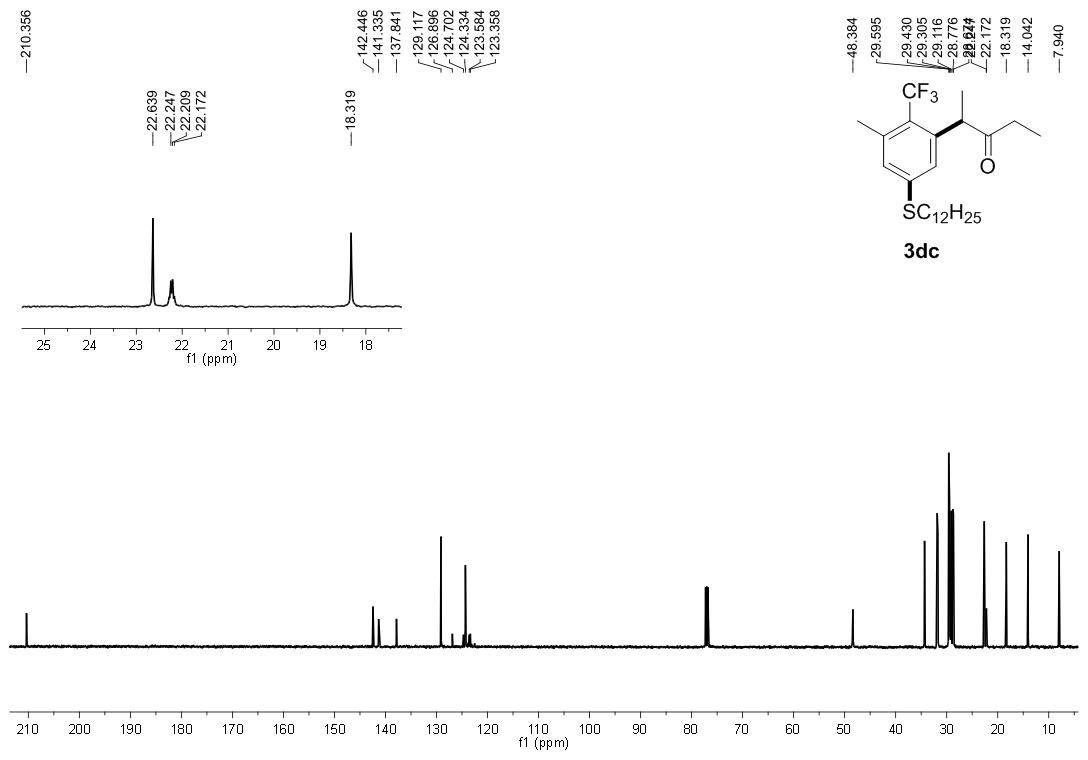
**

**^19^F NMR** (470 MHz, CDCl_3_) for **3dc**

**
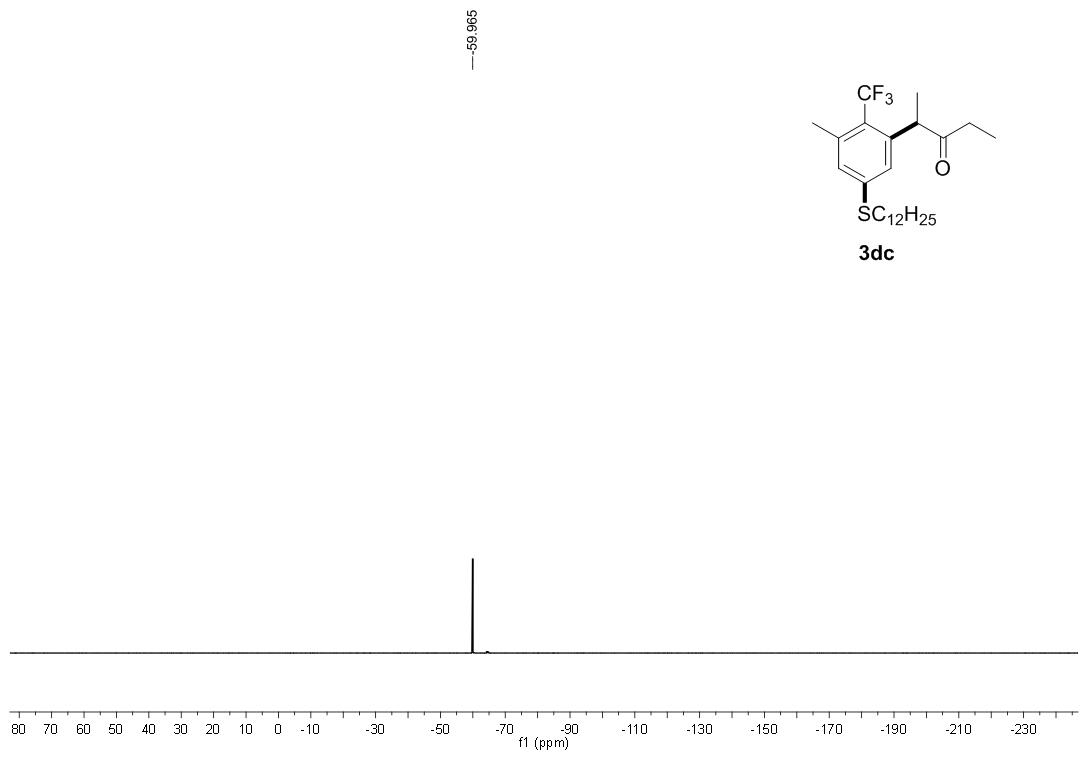
**

**^1^H NMR** (500 MHz, CDCl_3_) for **3ec**

**
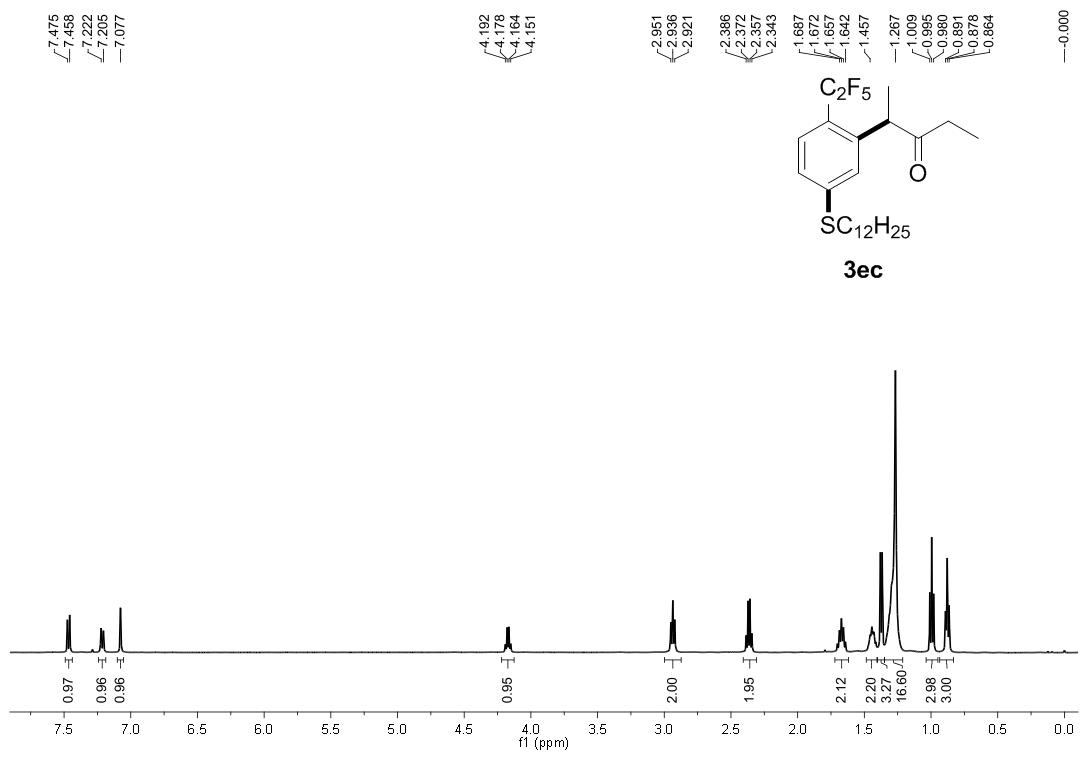
**

**^13^C NMR** (125 MHz, CDCl_3_) for **3ec**

**
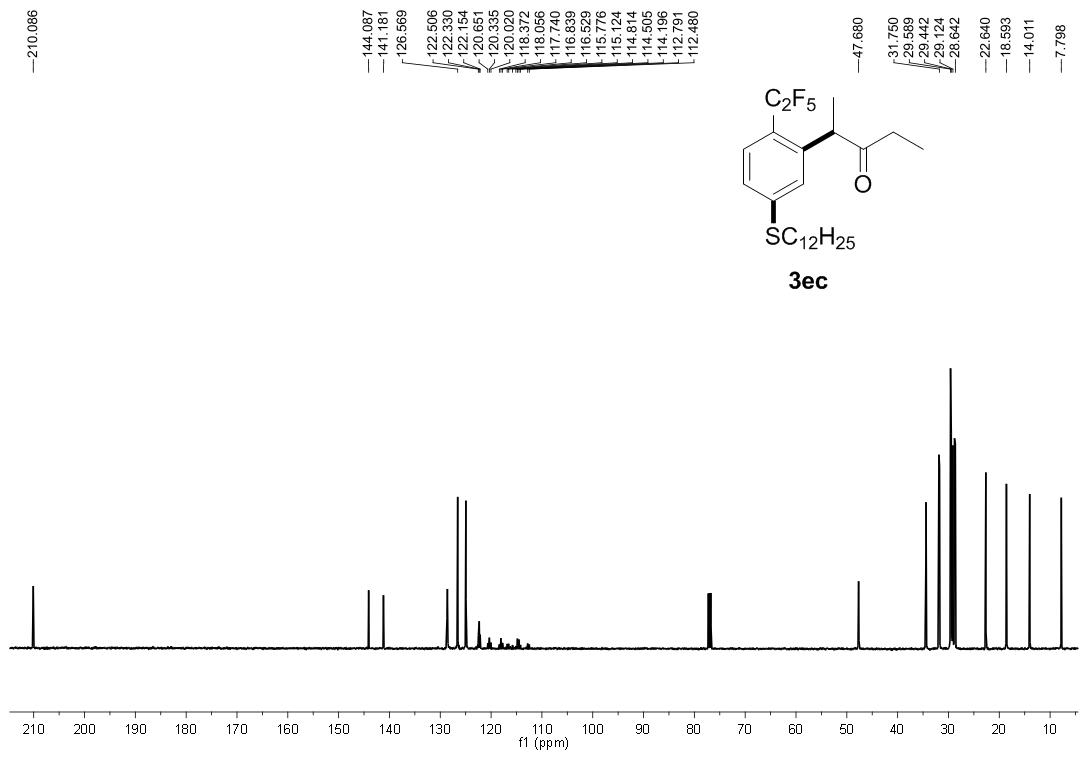
**

**^19^F NMR** (470 MHz, CDCl_3_) for **3ec**

**
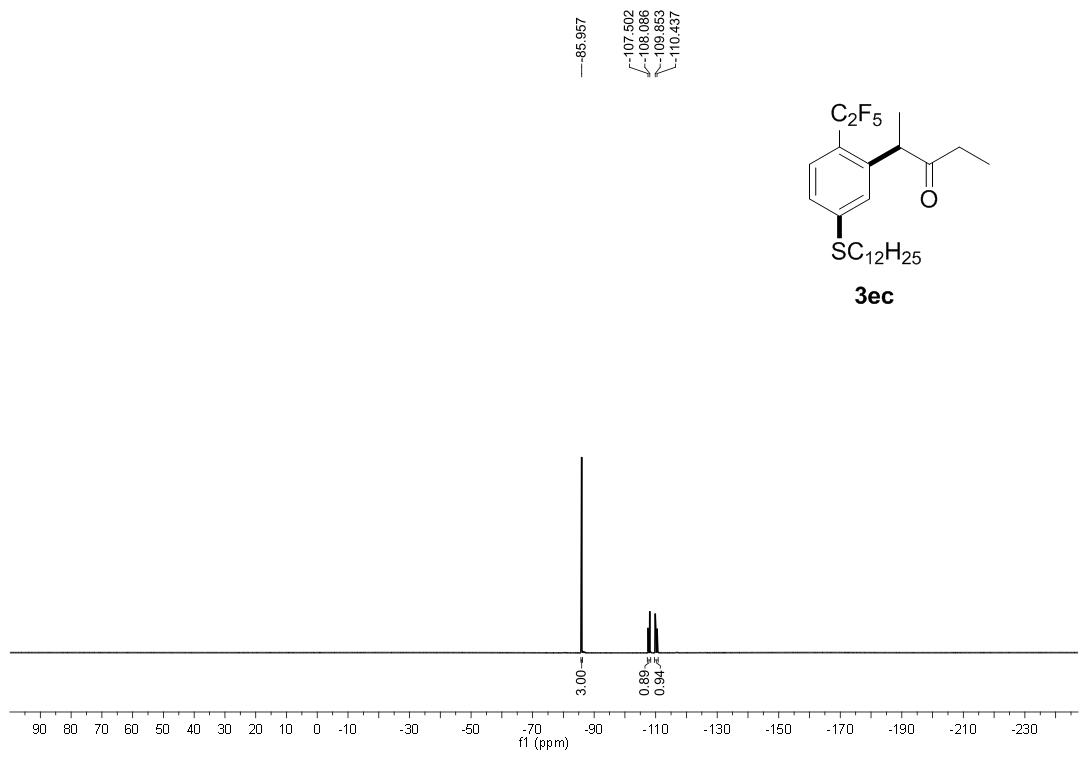
**

**^1^H NMR** (500 MHz, CDCl_3_) for **10a**

**
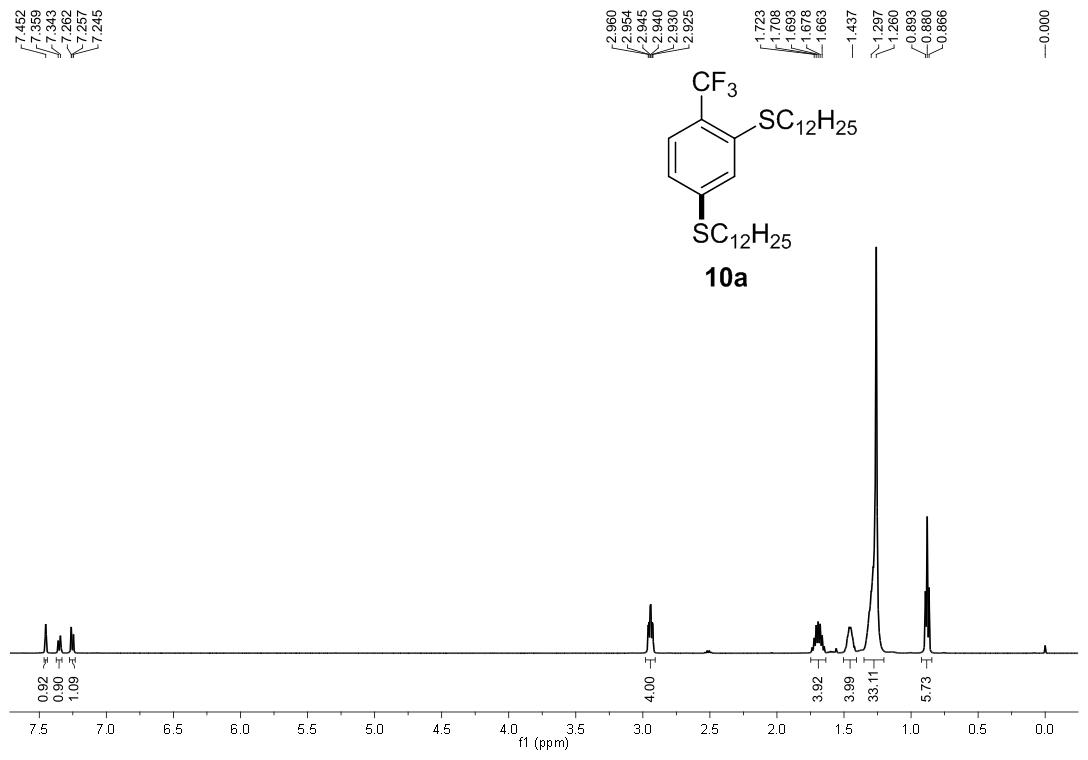
**

**^13^C NMR** (125 MHz, CDCl_3_) for **10a**

**
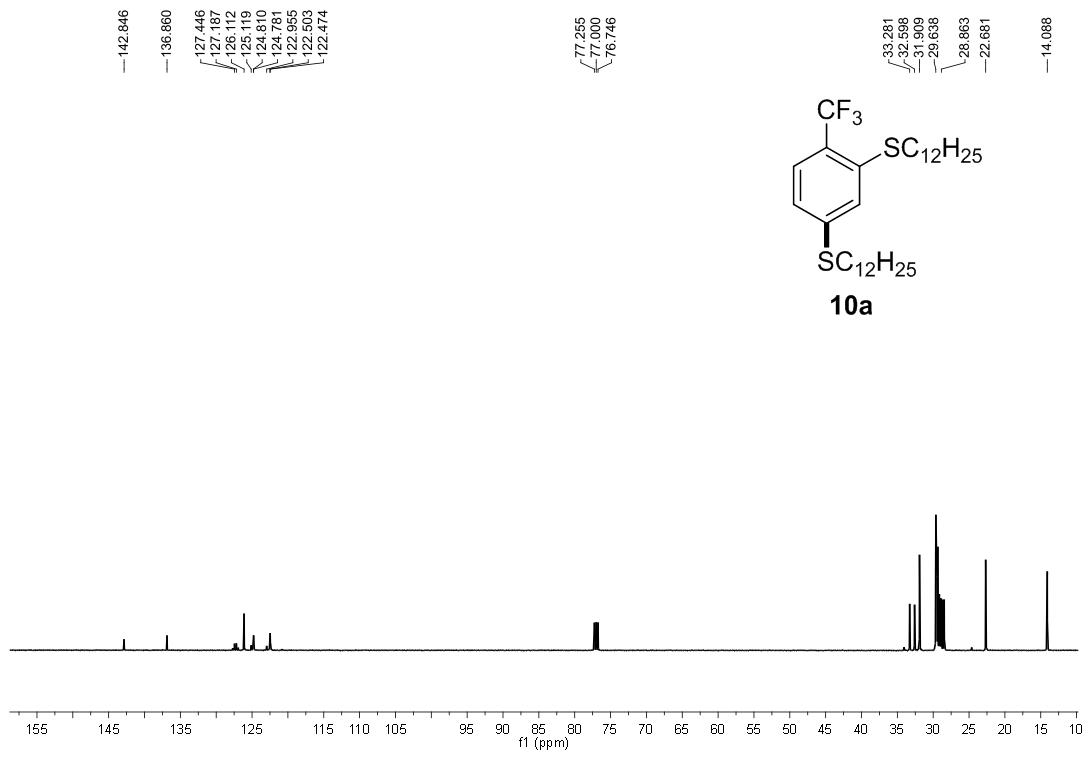
**

**^1^H NMR** (500 MHz, CDCl_3_) for **5aa/5aa’**

**
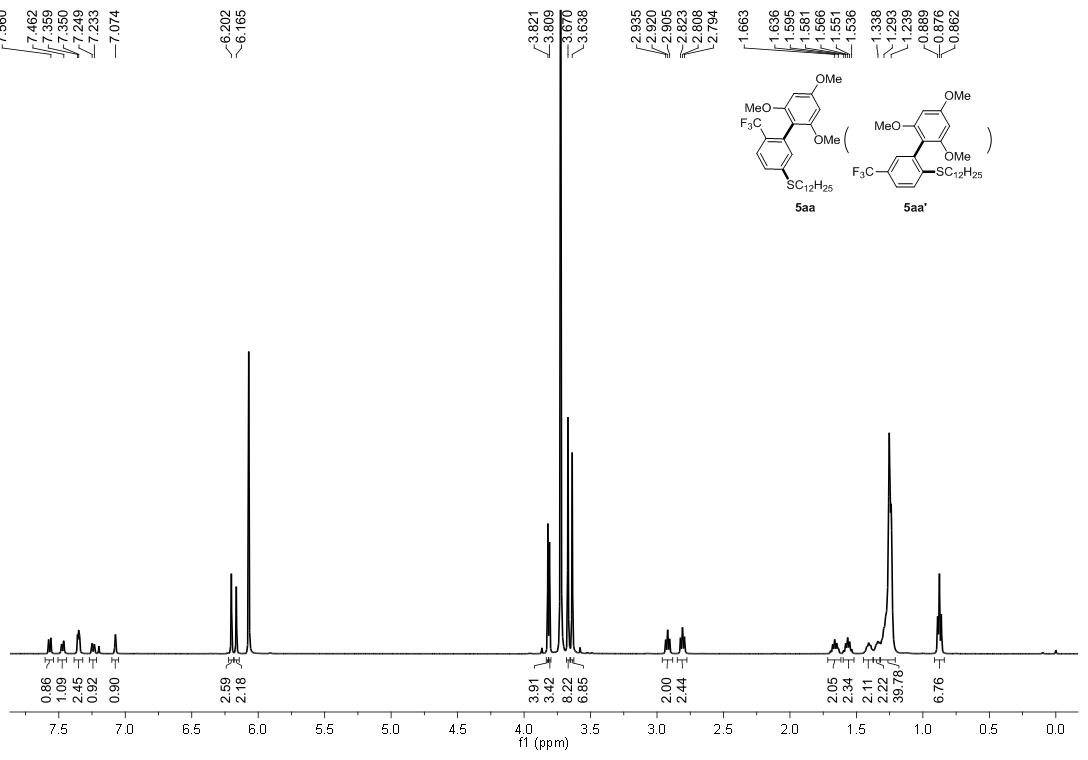
**

**^13^C NMR** (125 MHz, CDCl_3_) for **5aa/5aa’**

**
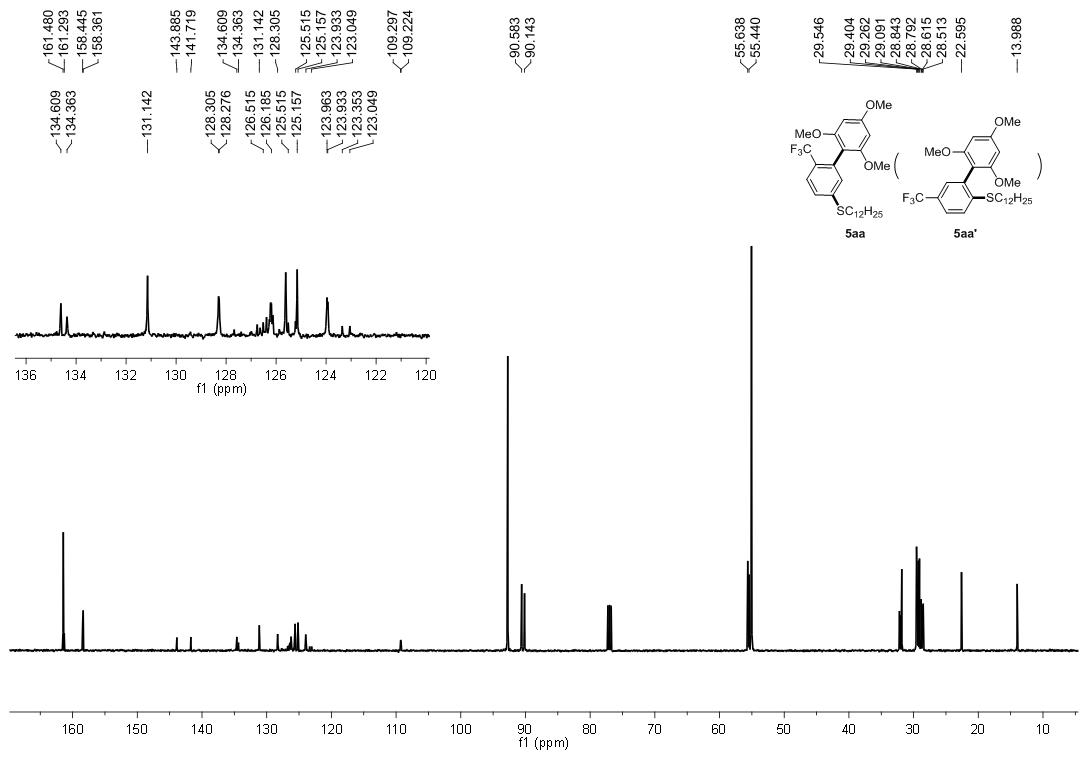
**

**^19^F NMR** (470 MHz, CDCl_3_) for **5aa/5aa’**

**
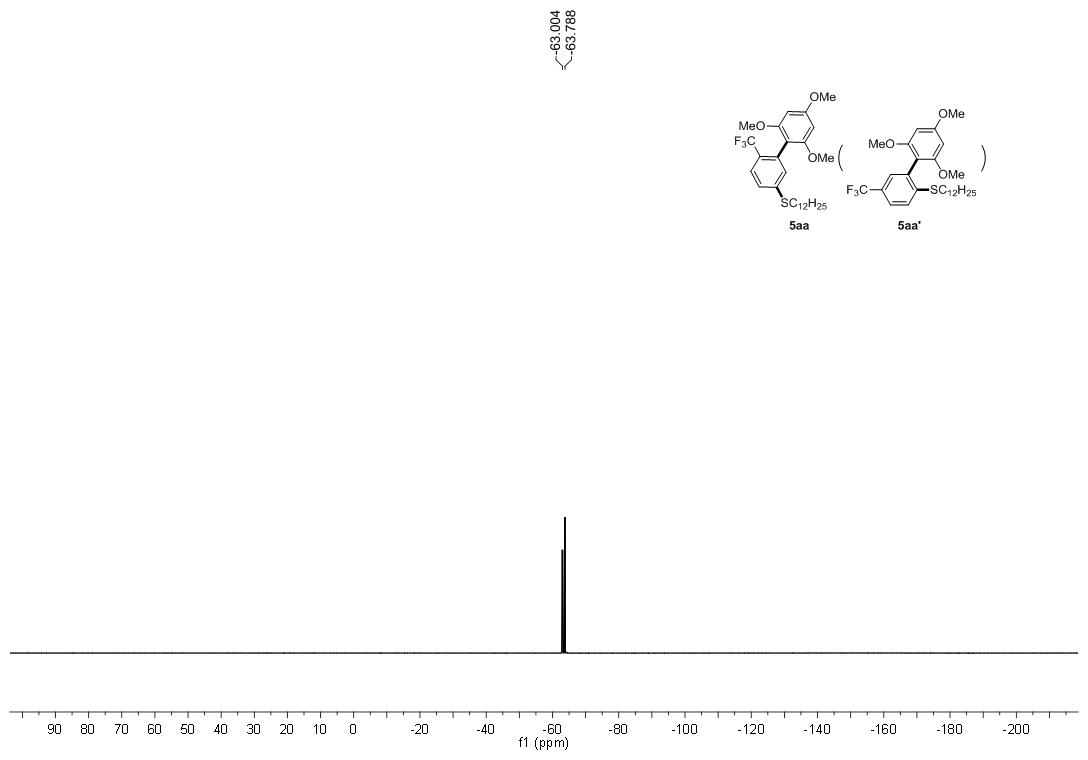
**

**^1^H NMR** (500 MHz, CDCl_3_) for **5ba/5ba’**

**
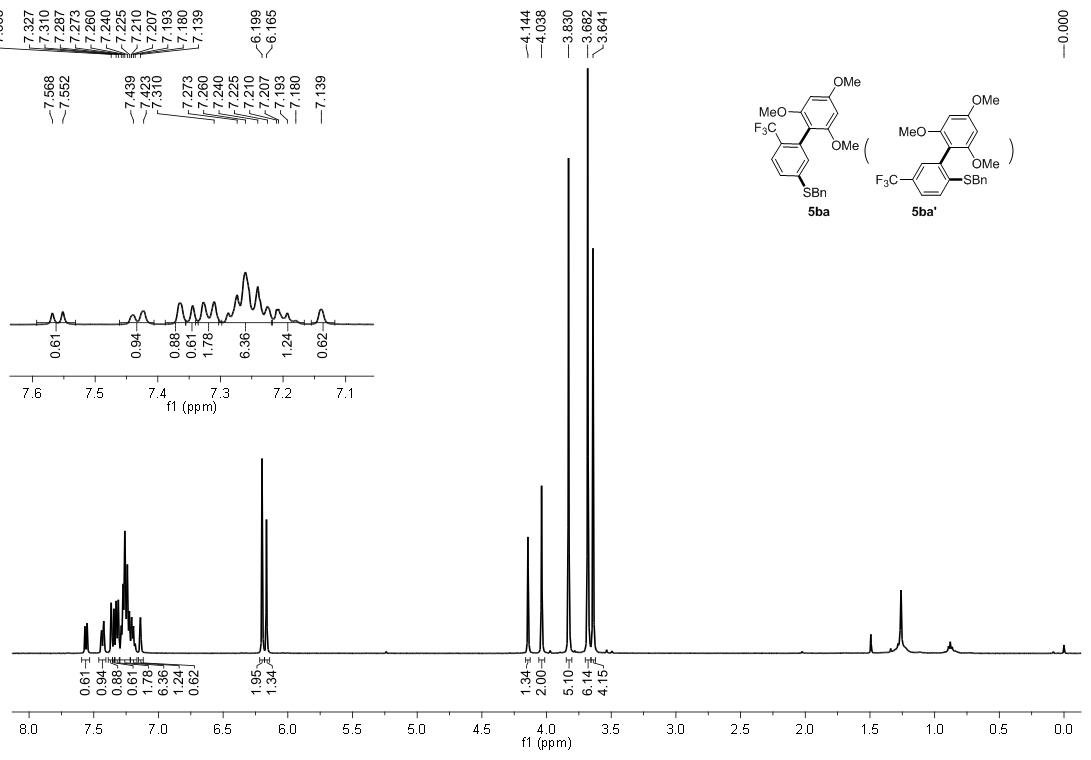
**

**^13^C NMR** (125 MHz, CDCl_3_) for **5ba/5ba’**

**^19^F NMR** (470 MHz, CDCl_3_) for **5ba/5ba’**

**^1^H NMR** (500 MHz, CDCl_3_) for **5ba’**

**^13^C NMR** (125 MHz, CDCl_3_) for **5ba’**

**^1^H NMR** (500 MHz, CDCl_3_) for **5ab/5ab’**

**^13^C NMR** (125 MHz, CDCl_3_) for **5ab/5ab’**

**^19^F NMR** (470 MHz, CDCl_3_) for **5ab/5ab’**

**^1^H NMR** (500 MHz, CDCl_3_) for **5ac/5ac’**

**^13^C NMR** (125 MHz, CDCl_3_) for **5ac/5ac’**

**^19^F NMR** (470 MHz, CDCl_3_) for **5ac/5ac’**

**^1^H NMR** (500 MHz, CDCl_3_) for **5ad/5ad’**

**^13^C NMR** (125 MHz, CDCl_3_) for **5ad/5ad’**

**^19^F NMR** (470 MHz, CDCl_3_) for **5ad/5ad’**

**^1^H NMR** (500 MHz, CDCl_3_) for **5ae/5ae’**

**^13^C NMR** (125 MHz, CDCl_3_) for **5ae/5ae’**

**^19^F NMR** (470 MHz, CDCl_3_) for **5ae/5ae’**

**^1^H NMR** (500 MHz, CDCl_3_) for **5bf/5bf’**

**^13^C NMR** (125 MHz, CDCl_3_) for **5bf/5bf’**

**^19^F NMR** (470 MHz, CDCl_3_) for **5bf/5bf’**

**^1^H NMR** (500 MHz, CDCl_3_) for **5ag**

**^13^C NMR** (125 MHz, CDCl_3_) for **5ag**

**^19^F NMR** (470 MHz, CDCl_3_) for **5ag**

**^1^H NMR** (500 MHz, CDCl_3_) for **6**

**^13^C NMR** (125 MHz, CDCl_3_) for **6**

**^1^H NMR** (500 MHz, CDCl_3_) for **6’**

**^13^C NMR** (125 MHz, CDCl_3_) for **6’**

**^1^H NMR** (500 MHz, CDCl_3_) for **8aa**

**^13^C NMR** (125 MHz, CDCl_3_) for **8aa**

**^1^H NMR** (500 MHz, CDCl_3_) for **8ba**

**^13^C NMR** (125 MHz, CDCl_3_) for **8ba**

**^1^H NMR** (500 MHz, CDCl_3_) for **8ca**

**^13^C NMR** (125 MHz, CDCl_3_) for **8ca**

**^19^F NMR** (470 MHz, CDCl_3_) for **8ca**

**^1^H NMR** (500 MHz, CDCl_3_) for **8da**

**^13^C NMR** (125 MHz, CDCl_3_) for **8da**

**^1^H NMR** (500 MHz, CDCl_3_) for **8ab**

**^13^C NMR** (125 MHz, CDCl_3_) for **8ab**

**^1^H NMR** (500 MHz, CDCl_3_) for **8ab’**

**^13^C NMR** (125 MHz, CDCl_3_) for **8ab’**

**^1^H NMR** (500 MHz, CDCl_3_) for **8ac**

**^13^C NMR** (125 MHz, CDCl_3_) for **8ac**

**^1^H NMR** (500 MHz, CDCl_3_) for **8ad**

**^13^C NMR** (125 MHz, CDCl_3_) for **8ad**

**^1^H NMR** (500 MHz, CDCl_3_) for **9**

**^13^C NMR** (125 MHz, CDCl_3_) for **9**

1. 1 Camps P. *et al.* Synthesis of polysubstituted bicyclo[3.3.1]nonane-3,7-diones from cyclohexa-2,5-dienones and dimethyl 1,3-acetonedicarboxylate. *Tetrahedron* **56***,* 8141–8151 (2000). [↑](#footnote-ref-2)
2. ^2^ Sheldrick, G. M. *SHELXS-97*, *Programs for X-ray Crystal Structure Solution*, University of Göttingen, Göttingen, (Germany, 1997). [↑](#footnote-ref-3)
